# From Respiration to Secondary Metabolism: A Heme A Synthase-Mediated Regulatory Network Expands Indolizidine Chemical Space in Fungi

**DOI:** 10.34133/research.1236

**Published:** 2026-04-27

**Authors:** Wei Bai, Guangzhi Dai, Junyang Ji, Qi Du, Meiling Ding, Wenbo Han, Hao Zhu, Xincun Wang, Wenying Zhuang, Renxiang Tan

**Affiliations:** ^1^ Synthetic Biology Center for Chinese Medicine, School of Pharmacy, Nanjing University of Chinese Medicine, Nanjing 210023, P. R. China.; ^2^ State Key Laboratory of Pharmaceutical Biotechnology, Institute of Functional Biomolecules, Nanjing University, Nanjing 210023, P. R. China.; ^3^ State Key Laboratory of Mycology, Institute of Microbiology, Chinese Academy of Sciences, Beijing 100101, P. R. China.

## Abstract

In single-cell microbes, primary and secondary metabolism are synergistically integrated to support survival and environmental adaptation through the biosynthesis of structurally diverse and biologically active small molecules—known as secondary metabolites (SMs) or natural products, such as penicillin. However, the therapeutic potential of microbial SMs is often limited because their biosynthetic gene clusters remain silent or weakly expressed under laboratory conditions. In this study, we employed BioNavi-NP as a guide to identify heme A synthase (*Cl*HAS) as a novel metabolic link in *Curvularia lunata*, bridging primary and secondary metabolism. Surprisingly, inactivation of *Cl*HAS led to the diversification of its characteristic indolizidine alkaloids, yielding previously unreported molecular frameworks. Mechanistically, *Cl*HAS disruption impaired heme biosynthesis, thereby inducing oxidative stress that activated an uncharacterized basic-region leucine zipper transcription factor (A7370). A7370 binds specifically to a unique *cis*-element (ACGGCTGAC) in the promoter region of *cuaF*, a pathway-specific positive regulator of indolizidine alkaloid biosynthesis. This cascade regulation produces structurally unprecedented alkaloids, some of which show antibacterial activity equal or superior to the clinically prescribed drug, tinidazole, against a range of human pathogenic bacteria. Overall, this work reveals a conserved regulatory network that coordinates primary and secondary metabolism in fungi, providing new mechanistic insights into the complexity of fungal SM biosynthesis.

## Introduction

Like plants, microbes produce structurally diverse secondary metabolites (SMs), or natural products, derived from primary metabolic precursors (e.g., acetyl-coenzyme A [CoA]) and sometimes enzyme cofactors [1]. Pioneering fungal SMs like penicillin, which launched the antibiotic era, possess unique chemical scaffolds and optimized bioactive properties. This enduring dogma underscores why bioactive fungal SMs remain an indispensable source of lead compounds for new medicines (e.g., lovastatin) and agrochemicals (e.g., strobilurin) [2]. However, the past few decades have witnessed a remarkable deceleration in discovering druggable SMs [3]. This slowdown stems from the frequent rediscovery of known compounds and the scarcity (or low abundance) of novel ones, even though genomic data indicate that the biosynthetic potential of fungi is far greater than the SM diversity isolated in laboratories [4]. In response, innovative strategies are being developed to activate silent or poorly expressed biosynthetic pathways, leading to the discovery of previously overlooked chemical scaffolds such as bioactive peptides [5] and halogenated compounds [6]. The SM biosynthesis typically initiates with the polymerization or condensation of building blocks from primary metabolism. The nascent precursors are then modified through multiple redox reactions, which install core molecular frameworks and ultimately yield SMs of intriguing complexity [7,8]. In addition to enzyme-catalyzed reactions, the biosynthesis of many SMs involves nonenzymatic or (thermodynamically) spontaneous steps [9]. Although certain activators of primary metabolism can also serve as negative regulators for specific SMs [10], a fundamental question therefore persists: the identity and mechanistic role of the central regulator coordinating primary metabolic flux and SM biosynthesis. Unraveling this regulatory hub is crucial for bypassing the current bottleneck that impedes the efficient discovery and application of novel fungal SMs.

In nature, the key driver of SM diversification lies in a collective microbial response to environmental stimuli and physiological requirements [11]. A particularly important mediator in the SM biosynthesis is the stress-induced intracellular generation of reactive oxygen species (ROS). In plants, for example, ROS acts as key signaling molecules that trigger the production of distinct SM profiles in response to environmental cues [12]. ROS such as superoxide anion (O₂^**•**^‾), hydrogen peroxide (H₂O₂), hydroxyl radical (•OH), and singlet oxygen (^1^O₂) (Table S1) are incompletely reduced oxygen species generated through the 1-electron reduction of molecular oxygen (O_2_) within cell [13,14]. On one hand, as a central container in the process, mitochondria serve not only as a major site of oxygen reduction and ROS formation [15] but also as a source of primary metabolic building blocks for SM biosynthesis [8]. On the other hand, ROS-mediated redox signaling could dynamically regulate the activity and expression of some SM-biosynthesizing enzymes [16]. The integration of ROS-based signaling and starting unit supply by mitochondria encouraged us to hypothesize that an interplay exists between primary and secondary metabolism [17]. Central to this regulatory nexus is heme A synthase (HAS), an evolutionarily conserved enzyme that catalyzes the conversion of heme O into heme A. Reflecting its critical role in this process, HAS is also designated Cox15, as it is essential for cytochrome c (Cyt c) oxidase maturation and the formation of functional respiratory complexes [18]. These insights suggested that disruption of HAS could impair respiratory function, leading to incomplete oxygen reduction and elevated oxidative stress caused by ROS. Under oxidative ROS stimulation, basic-region leucine zipper (bZIP) proteins in fungi act as key regulators linking cellular stress to secondary metabolism. For instance, AtfA has been experimentally [19] and computationally [20] validated to modulate polyketide biosynthesis under oxidative stress, while RsmA and Yap1 are involved in the regulation of penicillin production [21]. These findings lead us to hypothesize that HAS-induced ROS generation may trigger oxidative stress responses mediated by bZIP proteins, thereby orchestrating the downstream biosynthesis of SMs in fungi.

**Fig. 1.**
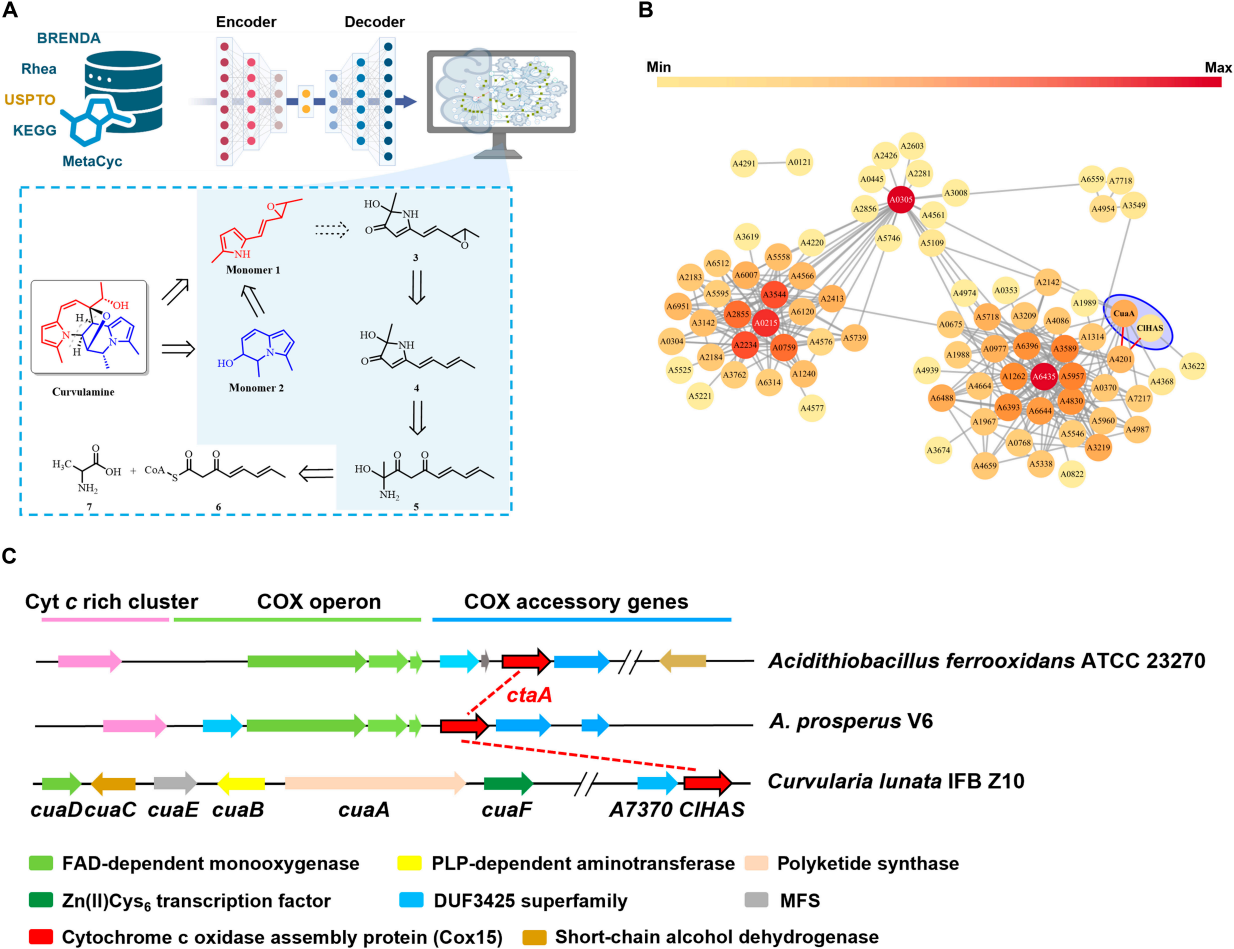
Bioretrosynthetic mapping of curvulamine biosynthesis and protein interactome analysis. *Cl*HAS was evidenced to be a redox hub linking mitochondrial heme maturation to curvulamine biosynthesis. (A) Schematic illustration for the BioNavi-NP protocol that explores nascent intermediates involved in curvulamine biosynthesis. The transformer neural networks were trained by combining the relevant biosynthetic and organic reactions, with 4 models trained with different hyperparameters forming the ensemble model that was used to make the single-step prediction. The bioretrosynthetic analysis of curvulamine integrated the expert-guided chemical logic, machine learning predictions, and experimentally validated biochemical reactions to reconstruct its biosynthetic pathway. (B) Protein–protein interaction (PPI) network of 31 core biosynthetic genes and 100 regulatory factors in the *C. lunata* genome. It was constructed using STRING network with a required interaction score of 0.4 and a PPI enrichment *P* value of 1.0e^−16^. The *Cl*HAS exhibited functional and genomic association with CuaA, encoded by a core (polyketide synthase) gene (circled in blue) responsible for curvulamine biosynthesis. (C) In some bacteria such as *Acidithiobacillus* strains, the cytochrome c oxidase (COX)-encoding genes are frequently clustered along with the primary metabolism genes responsible for the assembly of heme A and its cofactors. In contrast, the *C. lunata* genome harbors the encoding gene (*ClHAS*) close to the *cua* (curvulamine-biosynthesizing) cluster. BRENDA, Braunschweig Enzyme Database; USPTO, US Patent and Trademark Office; KEGG, Kyoto Encyclopedia of Genes and Genomes; MetaCyc, Metabolic Pathway Database. FAD, flavin adenosine dinucleotide; PLP, pyridoxal-5′-phosphate; MFS, major facilitator superfamily.

To test this hypothesis, we selected *Curvularia lunata* IFB-Z10, a fungus originally identified as *Curvularia* sp. IFB-Z10 that was found to produce a suite of antimicrobial indolizidine alkaloids, including curvulamine [22]. Indolizidine alkaloids feature structural novelty and promising bioactivities [23], yet those of microbial origin exhibit limited skeletal diversity. In bacteria, only cyclizidine and its congeners have been reported [24], while in fungi, swainsonine [25] has been extensively studied. They both share a monomeric 5- and 6-membered ring-fused system with a bridgehead nitrogen atom. In contrast, curvulamine features a dimeric skeleton, which is exceptionally rare in fungi. Although its structural uniqueness motivated the total synthesis of several derivatives, the synthetic routes were lengthy and suffered from low yields [26,27]. From a biosynthetic perspective, cyclizidine [28] and swainsonine [25] are assembled by polyketide synthase and nonribosomal peptide synthetase–polyketide synthase pathways, respectively, involving reductive release of the polyketide chain as an aldehyde, a reductive amination step followed by a series of downstream redox transformations. As a highly oxidized dimeric indolizidine, curvulamine from *C. lunata* thus provides an exceptional model for investigating redox-mediated regulation of primary and secondary metabolism. Our bioretrosynthetic analysis using BioNavi-NP confirmed that the biosynthesis of curvulamine in *C. lunata* involves multistep redox modifications (Fig. 1A), which is consistent with the proposed biosynthetic pathway [22]. Furthermore, genome-wide protein–protein interaction screening suggested that the *ClHAS* gene may interact with genes encoding other biosynthetic enzymes, especially the polyketide synthase CuaA [22]. Here, we present a redox-mediated link between mitochondrial respiration and secondary metabolism, orchestrated by fungal transcription factors like bZIP and Zn(II)₂Cys6, whose homologs are widespread in fungi [21]. The finding illuminates previously unrecognized (HAS involved) crosstalk between primary and secondary metabolism and thus provides a valuable addition to the available methodological arsenal for characterizing elusive bioactive indolizidine SMs from fungi.

## Results

### Bioretrosynthetic analysis highlights the *Cl*HAS involvement in the indolizidine alkaloid biosynthesis

The work was commenced by employing the bioretrosynthesis planning platform, BioNavi-NP, to propose a plausible biosynthetic route for curvulamine. Based on known chemical and biosynthetic reactions of indolizidine alkaloids from databases [29], supplemented by expert-guided manual curation, we first optimized a machine learning algorithm to predict multiple plausible biosynthetic routes to curvulamine (Fig. S1). Among these, one predicted pathway (Fig. 1A) aligned closely with the route we had previously established and experimentally validated [22]. Using this optimized framework, we integrated principles of chemical synthesis with the structural patterns of characterized indolizidine alkaloids, which led to the identification of 2 nitrogen-containing monomers as putative curvulamine precursors, (*E*)-2-methyl-5-(2-(3-methyloxiran-2-yl)vinyl)-1*H*-pyrrole (**1**) and 3,5-dimethyl-5,6-dihydroindolizin-6-ol (**2**) (Fig. 1A). Therefore, **1** and **2** were prioritized to be analyzed further, suggesting that the latter was redox-derived from the former by our transformer neural networks optimized through data augmentation and ensemble learning [30]. Our model further suggests that **1** is derived from (*E*)-2-hydroxy-2-methyl-5-(2-(3-methyloxiran-2-yl)vinyl)-1,2-dihydro-3*H*-pyrrol-3-one (**3**), which is triggered by an epoxidation of 2-hydroxy2-methyl-5-((1*E*,3*E*)-penta-1,3-dien-1-yl)-1,2-dihydro-3*H*-pyrrol-3-one (**4**). Computational analysis indicated that **4** is biosynthesized through the oxidative condensation of acyl-CoA (**6**) and alanine (**7**) via a hypothetical intermediate (**5**) (Fig. 1A and Fig. S1B). This proposed route was corroborated by our earlier experimental findings reported in *Proceedings of the National Academy of Sciences* [22], wherein CuaA elongates acetyl-CoA to produce **6**, and CuaB catalyzes the oxidative condensation of **6** with **7** to form the putative intermediate **5**. Following pyridoxal-5′-phosphate release, **5** yields **4**, which is subsequently oxidized by CuaD to give **3**. This inference aligns with the multiple redox nature of the pathway, which proceeds sequentially from the early building blocks to the final intermediate. With the predictive capacity of this approach validated, we were prompted to search for the key redox-regulatory node. Thus, the genome-wide protein–protein interaction analysis was performed in *C. lunata*, focusing on 31 core biosynthetic genes involved in SM production. This screen identified 100 regulatory proteins for further evaluation. In particular, *Cl*HAS (“*Cl*” signifying its origination from *C. lunata*) exhibited a substantial association with CuaA (Fig. 1B), a polyketide synthase essential for curvulamine biosynthesis [22]. Based on these findings, we were encouraged to propose that *Cl*HAS acts as a redox-sensitive gatekeeper capable of modulating curvulamine biosynthesis through a respiratory-metabolic orchestration mechanism.

**Fig. 2.**
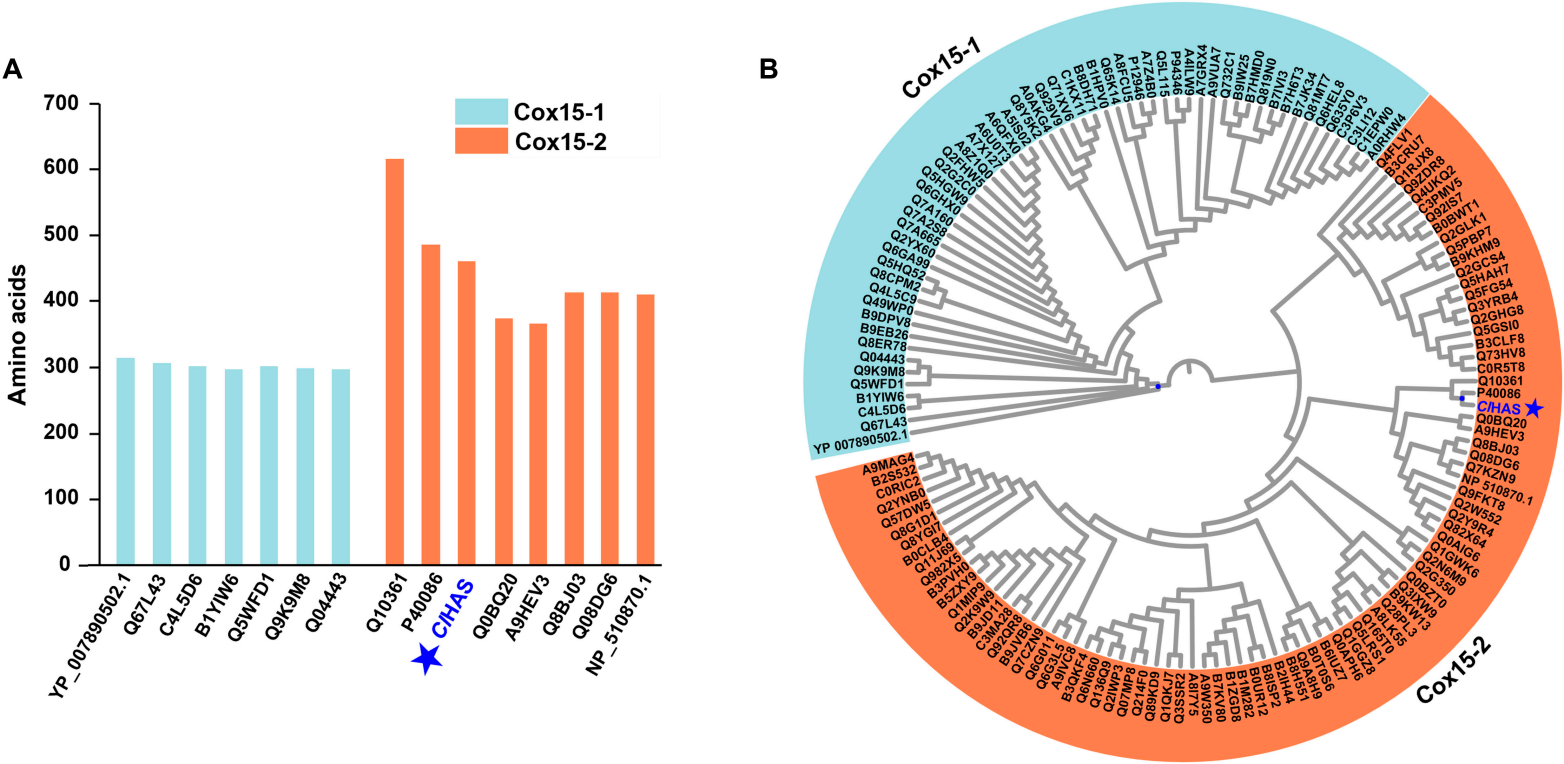
Phylogenetic analysis of *Cl*HAS and described Cox15 (heme A synthase [HAS]) proteins. (A) Cox15-1 proteins like CtaA have a shorter amino acid sequence than Cox15-2 enzymes such as *Cl*HAS and its described homologs. (B) The evolutionary tree analysis of the Cox15 proteins. Utilizing the Interpro website, a collection of 146 representative Cox15 amino acid sequences was assembled and aligned with *Cl*HAS using Blosum62 with the Jukes–Cantor model, showing that *Cl*HAS is a Cox15-2 protein. For clarity, the Cox15-1 and Cox15-2 amino acid sequences were in blue and orange, respectively.

Our genomic analysis showed that *ClHAS* was close to the *cua* (curvulamine biosynthetic) cluster (Fig. 1C). In general, HAS or Cox15 enzymes fall into 2 distinct groups, Cox15-1 and Cox15-2, each possessing a unique sequence signature. In eukaryotes, genes encoding the predominant Cox15-2 proteins were largely acquired via endosymbiotic gene transfer from the mitochondrial genome to the nucleus, whereas an ancestral Cox15-1 form is uniquely retained in the mitochondrial DNA of *Andalucia godoyi* [31]. Based on this evolutionary context, we hypothesized that *Cl*HAS might be a Cox15-2 rather than Cox15-1 protein, and by extrapolation, most if not all fungi express HAS enzymes also fall within the Cox15-2 category, too. This hypothesis was confirmed by our phylogenetic analysis, demonstrating that *Cl*HAS encodes a protein with a characteristic extended length relative to Cox15-1 orthologs (Fig. 2A) and clusters within a well-defined clade alongside known Cox15-2 homologs (Fig. 2B).

Next, we felt it necessary to ascertain whether *Cl*HAS could promote the transformation of heme O into heme A (Fig. S2). The *ClHAS* gene was inactivated to obtain the mutant (Table S2 and Fig. S3) for heme profiling. However, direct quantification of hemes A and O proved unfeasible due to technical limitations [32]. This frustration was overcome by a comparative LC-HR/MS (liquid chromatography hyphened with high-resolution mass spectrometry) profiling utilizing heme B as an internal standard. This approach revealed a marked accumulation of hemes B and O in the ∆*ClHAS* mutant relative to the wild-type (WT) strain (Fig. S4), indicating impaired conversion of heme O to heme A. The observation signified that *Cl*HAS catalyzes the conversion of heme O to heme A in *C. lunata* as does CtaA, a bacterial Cox15 (ref [18]). Our findings further support the view that *Cl*HAS and probably other Cox15-2 enzymes play an indispensable role in fungal respiratory adaptation required for primary metabolism.

### Inactivation of *Cl*HAS reshapes indolizidine alkaloid biosynthesis

**Fig. 3.**
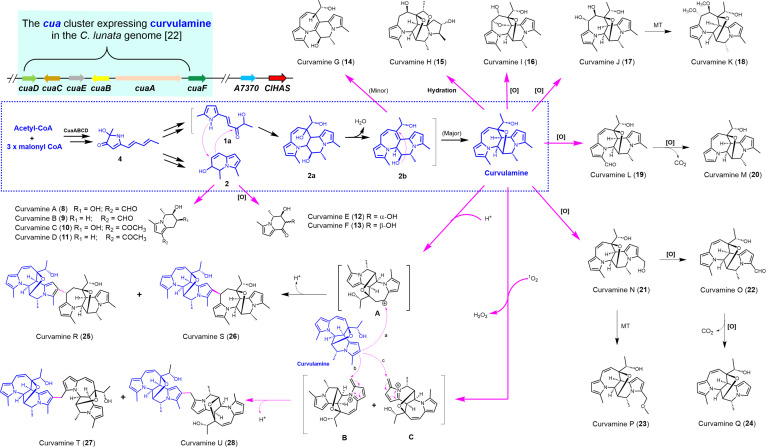
Deletion of *ClHAS* bifurcates and extends the curvulamine biosynthetic pathway in *C. lunata*. CoA, coenzyme A.

To gain insight into how *Cl*HAS is specifically involved in the biosynthesis of indolizidine alkaloids such as curvulamine, we compared the SM diversity of the ∆*ClHAS* and WT cultures, as the mutant’s heavier pigmentation suggested improved SM production (Fig. S5A). Subsequent high-performance liquid chromatography and LC-HR/MS analysis of ethyl acetate extracts from those cultures in 8 different media showed that more indolizidine alkaloids appeared in the mutant rather than WT cultures (Fig. S5B and C). The ∆*ClHAS* fermentation was therefore scaled up (Fig. S5C) to characterize a total of 21 undescribed indolizidine alkaloids, which we have named curvamines A-U (**8**–**28**) (Fig. 3). To our knowledge, this provides the first evidence linking HAS to fungal indolizidine alkaloid biosynthesis.

The structures and absolute stereochemistry of **8**–**28** were assigned by interpreting their LC-HR/MS, 1-dimensional (^1^H and ^13^C) and 2-dimensional nuclear magnetic resonance spectra (^1^H-^1^H correlation spectroscopy, heteronuclear single quantum coherence, heteronuclear multiple bond correlation, and nuclear overhauser effect spectroscopy) (Figs. S25 to S150 and Tables S7 to S27), by matching recorded and computed electronic circular dichroism spectra (Figs. S151 to S165), and by single crystal x-ray diffraction if necessary (Fig. S166). Evidenced from the molecular framework and substitution pattern of **8**–**28**, the *ClHAS* deletion appears to intensify the *cua* cluster expression leading to arrays of new indolizidine alkaloids (Fig. 3). Our metabolic analysis of the ∆*ClHAS* culture identified shunt products curvamines A–G (**8**–**14**) and downstream alkaloids curvamines H–U (**15**–**28**). The structures of **8**–**28** reveal an unexpected bifurcation and subsequent extension of the curvulamine biosynthetic pathway (Fig. 3). A tremendous amount of work is required to detail biosynthetic pathways toward these new alkaloids, falling beyond the scope of the present investigation. Given that gut bacterial infections [33,34] are frequently linked to intestinal toxicity and certain cancers [35], addressing these infections is an urgent concern. Therefore, all new alkaloids were subjected to bioassay against a panel of human pathogenic anaerobic bacteria. Notably, curvamine B (**9**) was demonstrated to be substantially active against *Streptococcus pneumoniae*, *Bacteroides vulgatus*, and *Peptostreptococcus micros* with minimum inhibitory concentrations (MICs) of 0.62, 1.30, and 0.62 μM, respectively. Moreover, curvamine P (**23**) exhibited potent activity against *Veillonella parvula* with its MIC value of 0.34 μM. The bioassay results reflected that **9** and **23**, 2 representatives of newly characterized alkaloids from the ∆*ClHAS* mutant, were more antibacterial than tinidazole, an antibacterial medicine coassayed as a positive control in the study (Table 1).

**Table 1.** MICs of curvamines against human pathogenic anaerobic bacteria *^a^*

	MIC (μM)				
Compounds	*Veillonella parvula*	*Actinomyces israelii*	*Streptococcus pneumoniae*	*Bacteroides vulgatus*	*Peptostreptococcus micros*
Curvamine A (**8**)	2.39	>10	>10	9.57	9.57
Curvamine B (**9**)	0.62	>10	0.62	1.30	0.62
Curvamine F (**13**)	0.62	>10	>10	>10	5.13
Curvamine O (**22**)	2.51	>10	5.02	5.02	5.02
Curvamine P (**23**)	0.34	>10	5.65	2.82	5.65
Curvamine Q (**24**)	1.61	>10	>10	6.45	>10
Curvamine T (**27**)	4.46	>10	>10	8.92	8.92
Tinidazole *^b^*	0.49	32.39	1.01	2.02	2.02

MIC, minimum inhibitory concentration

^a^ Cultured under anaerobic conditions.

^b^ Coassayed as a positive control.

### Insights into the *Cl*HAS regulation of indolizidine alkaloid biosynthesis

**Fig. 4.**
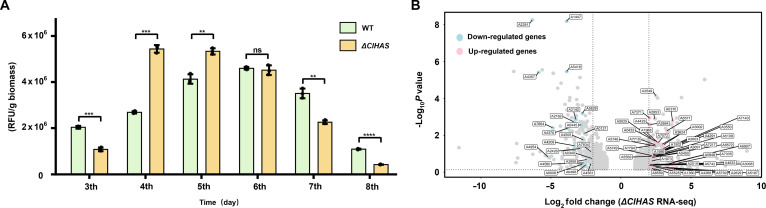
The difference in oxidative stress responses between Δ*ClHAS* and wild-type (WT) strains. (A) Quantitative assessment of the intracellular reactive oxygen species (ROS) levels after a 3- to 8-d cultivation. Data represent the mean of 3 independent experiments, **P < 0.01, ***P < 0.001, and ****P < 0.0001. ns, not significant; RFU, relative fluorescence units. (B) Volcano plot depicting transcriptomic changes after a 4-d cultivation. Transcriptome analysis pinpointed a total of 44 and 22 genes whose expressions were respectively up- and down-regulated in response to the oxidative stress (|log_2_fold change| > 2). RNA-seq, RNA sequencing.

Dysfunction in the mitochondrial respiration chain generates an increased amount of ROS [36]. Given the dual involvement of the *Cl*HAS in both mitochondrial respiration and SM biosynthesis, we hypothesized that the diversification of indolizidine alkaloids might arise from disruption of intracellular oxidative homeostasis in response to gene deletion. The hypothesis was initially confirmed by comparing the intracellular ROS levels of ∆*ClHAS* and WT strains with the method modified from the described protocol [37] (see Materials and Methods). As predicted, on the fourth day of cultivation, the intracellular ROS level of the mutant was twice that of WT strain (Fig. 4A). With prolonged fermentation, the intracellular ROS in the mutant helped to biosynthesize a new array of indolizidine alkaloids (Fig. 4A and Fig. 3). We then performed the transcriptome profiling of mutant and WT strains on the fourth day of cultivation, showing that up-regulation of 44 oxidative stress-dependent proteins (log_2_fold change > 2) and the down-regulation of 22 others (Fig. 4B and Fig. S6). The findings highlighted that the deletion of *ClHAS* disrupted the intracellular redox equilibrium, alters oxidative gene expression patterns, and elicits varied oxidative stress responses, ultimately leading to pronounced differences in the SM profiles of ∆*ClHAS* and WT strains.

It is established that Cyt c plays an indispensable role in the electron transport in the mitochondrial respiratory chain. Considering that its complexation with cardiolipin can induce the generation of ^1^O₂ [38], we speculated that the knockout of *ClHAS* might result in the intracellular accumulation of Cyt c, leading subsequently to an increased endogenous ^1^O_2_ level. Through a bimolecular transition, 2 ^1^O_2_ molecules simultaneously decay to the triplet (ground state) oxygen (^3^O_2_) by emitting the residual energy as visible light with a characteristic band at 634 nm [39]. Utilizing 9,10-diphenylanthracene as a trapping agent (Fig. S7A and B), we conducted fluorescence assays to compare the internal ^1^O_2_ levels in the mutant and WT strains. On the fourth day of cultivation, the ^1^O_2_ concentration in the mutant approximated twice that of WT strain (Fig. S7C). In the exposure to high(er) level of ^1^O_2_, more amounts of **27** and **28** were produced from curvulamine (Fig. 3). If escalated above the reaction threshold, ^1^O_2_ likely engages in a Diels–Alder-like reaction with 2 pyrrole diene motifs of curvulamine to afford the corresponding endoperoxide intermediates (Fig. S8). Subsequently, they undergo endoperoxide ring opening, releasing H_2_O_2_ and forming quaternary ammonium salts (**B** and **C**) (Figs. S8 and S9). The ammonium-polarized exomethylene group in **B** and **C** allows for the **27** and **28** generation by bonding covalently with the most nucleophilic carbon in the curvulamine molecule (Fig. 3). To verify this mechanism, we performed an in vitro assay in which curvulamine was irradiated in the presence of *β*-mercaptoethanol. This experiment successfully trapped the labile intermediates as corresponding adducts. Our LC-HR/MS analysis of the adducts confirmed the proposed structures (Fig. S10). These lines of evidence demonstrate that the deletion of *ClHAS* disrupts intracellular redox homeostasis by elevating ROS levels, particularly ^1^O₂, which in turn drives the oxidative tailoring of curvulamine and its precursors, ultimately leading to the generation of diverse arrays of highly oxidized alkaloids in the ∆*ClHAS* mutant of *C. lunata*.

**Fig. 5.**
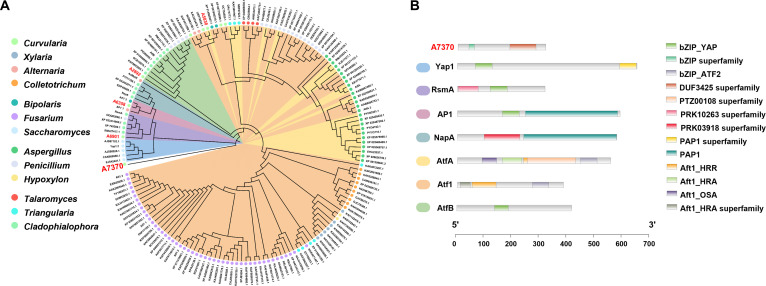
Bioinformatic analysis of fungal basic-region leucine zipper (bZIP) proteins. (A) Evolutionary analysis of 161 fungal bZIPs. The evolutionary tree was constructed from 156 bZIP proteins (amino acid sequences from National Center for Biotechnology Information (NCBI) database, representing 12 genera) along with the 5 counterparts (A7370, A5838, A2892, A6358, and A6901) from *C. lunata*, by aligning the sequences using Mega and iTOL bioinformatics tools [56]. A7370 fell outside all characterized bZIP clades and thus believed to represent an independent fungal bZIP lineage. (B) Sequence-based characterization of conserved motifs of bZIPs using TBtools-II [57]. A7370 protein was indicated to possess a conserved motif named as DUF3425, a domain with unknown function.

Given the established role of fungal bZIP transcription factors (e.g., Yap1 [40] and RsmA [21]) in regulating SM biosynthesis in response to oxidative stress, we reasoned that the disruption of intracellular oxidative homeostasis resulting from *ClHAS* deletion in *C. lunata* might involve bZIP-mediated regulation. To identify potential regulators, we were motivated to reanalyze the genome of *C. lunata* and identified 5 putative bZIP-encoding genes (*A7370*, *A2892*, *A5838*, *A6358*, and *A6901*) located outside the *cua* cluster. To assess their phylogenetic relationships, we aligned the 5 candidate sequences with 156 National Center for Biotechnology Information-deposited bZIP proteins characterized from 12 fungal genera, constructing a phylogenetic tree using HMMER 3.2.1. program [41] at an e-value cutoff of 1e^−3^. Intriguingly, A7370 occupies a position outside all identified bZIP families, placing it in a distinct clade (Fig. 5A). Furthermore, A7370 was indicated to harbor a conserved region designated as DUF3425, which was not possessed by described bZIP proteins and thus remained functionally unknown (Fig. 5B). This unique domain architecture led us to assume that A7370 might have a regulatory function distinct from those of known bZIP transcription factors. To address the assumption, we initially compared the expression levels of all 5 bZIP genes between the ∆*ClHAS* mutant and WT strains using reverse transcription quantitative polymerase chain reaction (RT-qPCR) experiments (Table S3, see Materials and Methods). As illustrated in Fig. S11A, the deletion of *ClHAS* facilitated the up-regulated expression of the 5 bZIP genes (vide supra) relative to those in WT strain. Notably, A7370 exhibited the most pronounced transcriptional activation, suggesting a specific correlation between *ClHAS* deletion and A7370 induction. The observation was then reinforced by the transcriptome data, being indicative of the up-regulated *A7370* transcripts in the mutant (Fig. S11B). Collectively, these results implicate A7370 as a unique transcriptional regulator potentially responsive to genomic or oxidative perturbations.

The bZIP proteins typically bind to promoter regions via hairpin structural motifs, thereby modulating specific transcription factor expressions under stress conditions [42,43]. This form of regulation is prominent for transcription factors specific to a particular biosynthetic gene cluster (BGC) [42], and can substantially influence the diversification of corresponding SMs [21,44]. The aforementioned indolizidine alkaloids overproduction by the *ClHAS* deletion prompted us to hypothesize that intracellular ROS accumulation affect the SM diversity in *C. lunata*. Agreeably, our RT-qPCR and transcriptome analysis revealed a pronounced up-regulation of all indolizidine-expressing (*cuaA*-*F*) genes in the mutant compared to WT strain, with *cuaBC* expressions being induced more dramatically (Fig. S11B and C). These transcriptional changes support a model linking the elevated ROS level to indolizidine alkaloid accumulation, wherein oxidative stress potentiates specific activation of *cuaF* by A7370, which in turn enhances the coordinated up-regulation of *cua* cluster genes and ultimately leads to alkaloid production. This model gained its corroboration from our *cuaF* overexpression (OE::*cuaF*) (Fig. S12), which allowed for curvulamine’s and its analogs’ hyperproduction as done by the mutant (Fig. S13), mirroring the metabolite profile of the ∆*ClHAS* mutant (Fig. S13). Conversely, the fungus was deprived of producing indolizidine alkaloids (Fig. S13) by our *cuaF* knockout (Fig. S14), confirming its essential role in this pathway. To elucidate the function of A7370, we scrutinized the difference in the indolizidine alkaloids’ productivity between overexpressing (Fig. S15) and silencing the *A7370* gene (Table S4). As expected, A7370 overexpression enhanced the production of curvulamine and its derivatives, whereas A7370 silencing substantially suppressed the indolizidine analogs’ abundance (Fig. S13). This set of experiments established A7370 as a transcriptional activator that stimulates indolizidine alkaloid biosynthesis by specifically inducing the cluster-specific transcription factor CuaF in *C. lunata*.

**Fig. 6.**
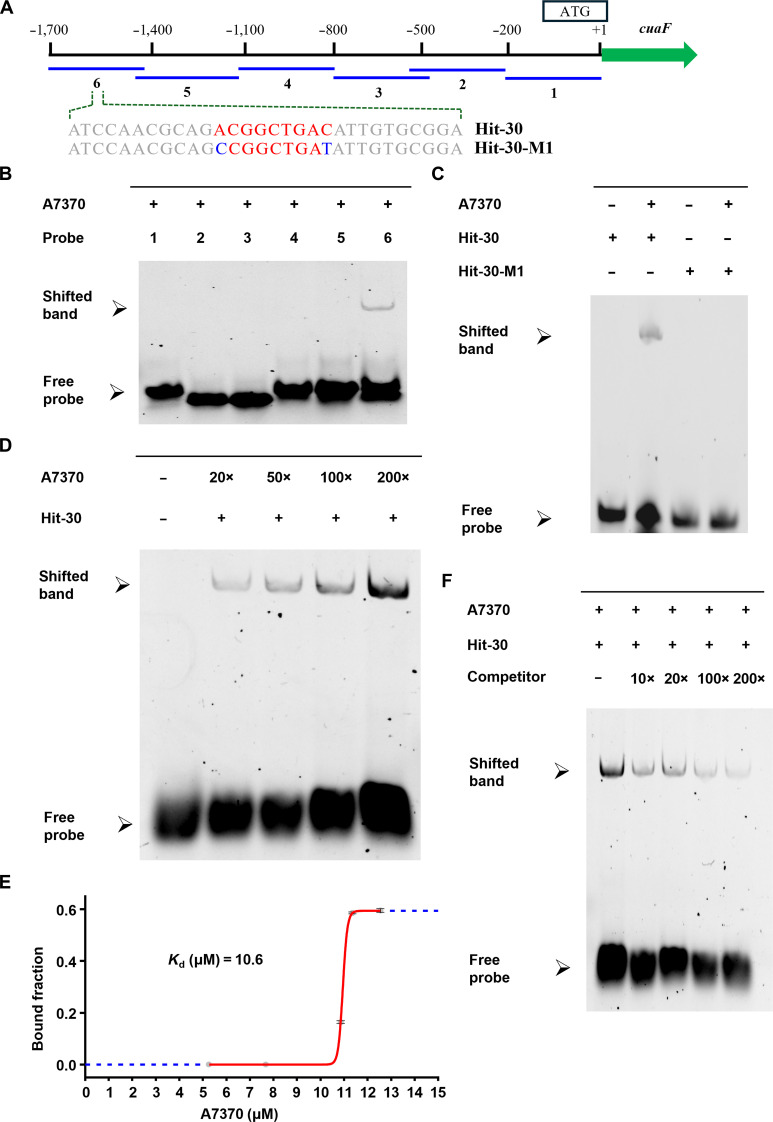
Specific binding of A7370 to the ACGGCTGAC motif in the promotor region of *cuaF*. (A) Schematic diagram of the *cuaF* promoter region showing the 6 partially overlapped fragments (probes 1 to 6) used in this study. Probe 6 encompasses hit-30. (B) Electrophoretic mobility shift assay (EMSA) screening for the A7370 interaction with 6 5'-carboxyfluorescein (5'-FAM) marked probes (lanes 1 to 6). Probe 6 exhibited a specific binding to A7370, whereas others displayed no detectable affinity, which aligns well with chromatin immunoprecipitation sequencing (ChIP-seq) assay. (C) Validation for the binding of A7370 to hit-30 (lane 1, hit-30; lane 2, hit-30 bound to A7370; lane 3, hit-30 M1; and lane 4, hit-30 M1 unable to bind to A7370). (D) The dose-dependent binding visualization of hit-30 to A7370 (from 20× to 200×). (E) Binding curve of A7370 to hit-30 of 10 pmol, derived from a linear regression of 3 independent replicates. (F) The affinity assay for the A7370 to hit-30 binding. The fluorescent intensity indicative of the binding intensity decreased as the concentration of unlabeled (5'-FAM-free) hit-30 increased gradually (from 10× to 200×).

**Fig. 7.**
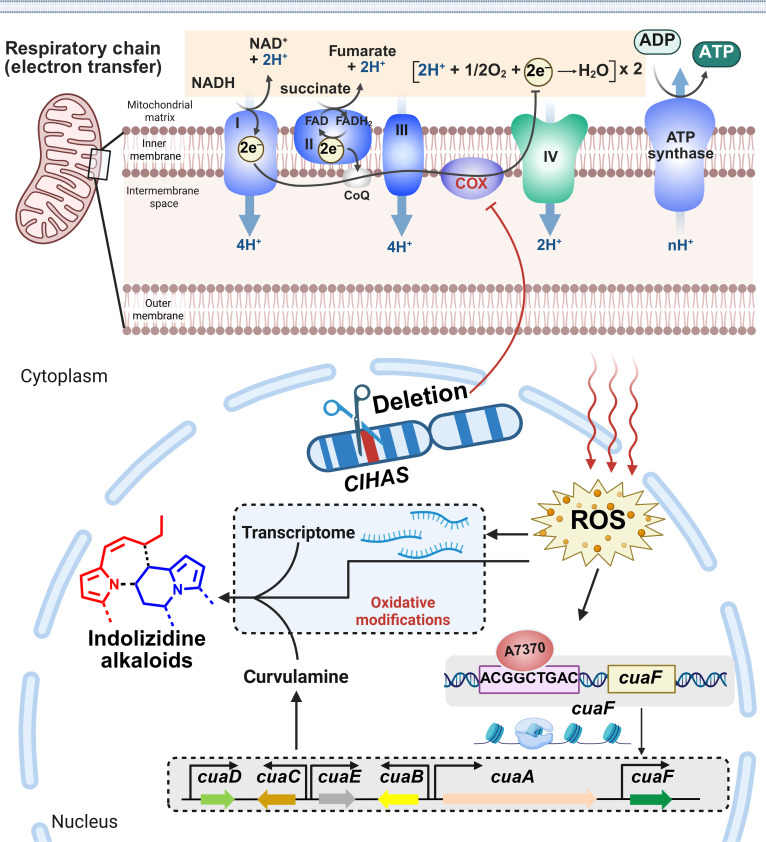
Proposed model for the oxidative stress-triggered up-regulation of indolizidine alkaloid biosynthesis in *C. lunata*. The *ClHAS* deletion escalates the intracellular reactive oxygen species (ROS) level to facilitate the binding of A7370’s hairpin substructure with the ACGGCTGAC motif in the promotor region of *cuaF*, an indolizidine pathway-specific regulator. Such an ROS-promoted binding up-regulates the *cuaF* expression to increase the transcriptional activity of the *cua* cluster and enhances the expression level of oxidative enzymes (Fig. 4B). These 2 factors synergize the biosynthesis of structurally diversified indolizidine alkaloids characterized from the mutant culture. This figure was created with the website http://www.biorender.com.

To elucidate the regulatory mechanism of A7370, we performed chromatin immunoprecipitation sequencing (ChIP-seq) analysis on strains expressing 3×Flag-tagged A7370. A total of 5,305 high-confidence binding peaks were identified by comparing the significant enrichment peaks with the input control. Approximately 79.3% of the peaks corresponded to promoter transcription start site (Fig. S18A and C), confirming the robustness of the ChIP-seq assay. Notably, a strong A7370 binding peak was observed in the region spanning −1,375 to −3,233 base pairs (bp) upstream of *cuaF* (Fig. S18D). To further validate this interaction, we performed electrophoretic mobility shift assays (EMSAs) to assess its DNA-binding specificity. Initial screening involved 6 partially overlapped 300-bp fragments sliced from the *cuaF* promotor region, which were used subsequently as probes 1 to 6 after labeled with 5'-carboxyfluorescein (5'-FAM) (Table S6 and Fig. 6A). Among them, only probe 6 showed specific binding (Fig. 6B) to the recombinant maltose-binding protein (MBP)-tagged A7370 (Fig. S19). Notably, the sequence of probe 6 fell exactly within the strongest binding region identified by our ChIP-seq analysis (Fig. S18D). To pinpoint the exact binding site, we further dissected probe 6 into shorter fragments, leading ultimately to the identification of a hit, a 30-bp critical segment called “hit-30”, which exhibited robust binding activity (Fig. 6C). Functional refinement through iterative probe truncation and site-directed mutagenesis under optimized reaction conditions (Fig. S20) further narrowed the recognition sequence to a 9-bp conserved motif (ACGGCTGAC). The specificity of this interaction was validated by dose-dependent binding assays with increasing concentrations of A7370 (Fig. 6D), from which the dissociation constant (*K*_d_) of 10.6 μM was determined (Fig. 6E). Further validation came from competitive inhibition assays using an unlabeled (5'-FAM-free) probe, which decreased the binding intensity under optimal conditions (Fig. 6F). Gratifyingly, mutation of the ACGGCTGAC motif to CCGGCTGAT (hit-30 M1) completely eliminated the A7370 binding, as shown by the absence of a band shift in EMSA (Fig. 6B). These results established ACGGCTGAC as a *cis*-acting element within the *cuaF* promoter region that is directly responsive to A7370. Collectively, the oxidative stress resulting from the *ClHAS* deletion elevates the intracellular ROS level, leading to up-regulation of A7370. This transcription factor in turn binds to the ACGGCTGAC motif in the *cuaF* promoter region, enhancing expression of the *cua* gene cluster and resulting in overproduction of indolizidine alkaloids. The increased abundance of these alkaloids above a reaction threshold further enables their diversification through enzymatic and/or spontaneous chemical modifications (Fig. 7 and Fig. 3).

### Genomic mining for simultaneous existence of A7370 and *Cl*HAS homologs

**Fig. 8.**
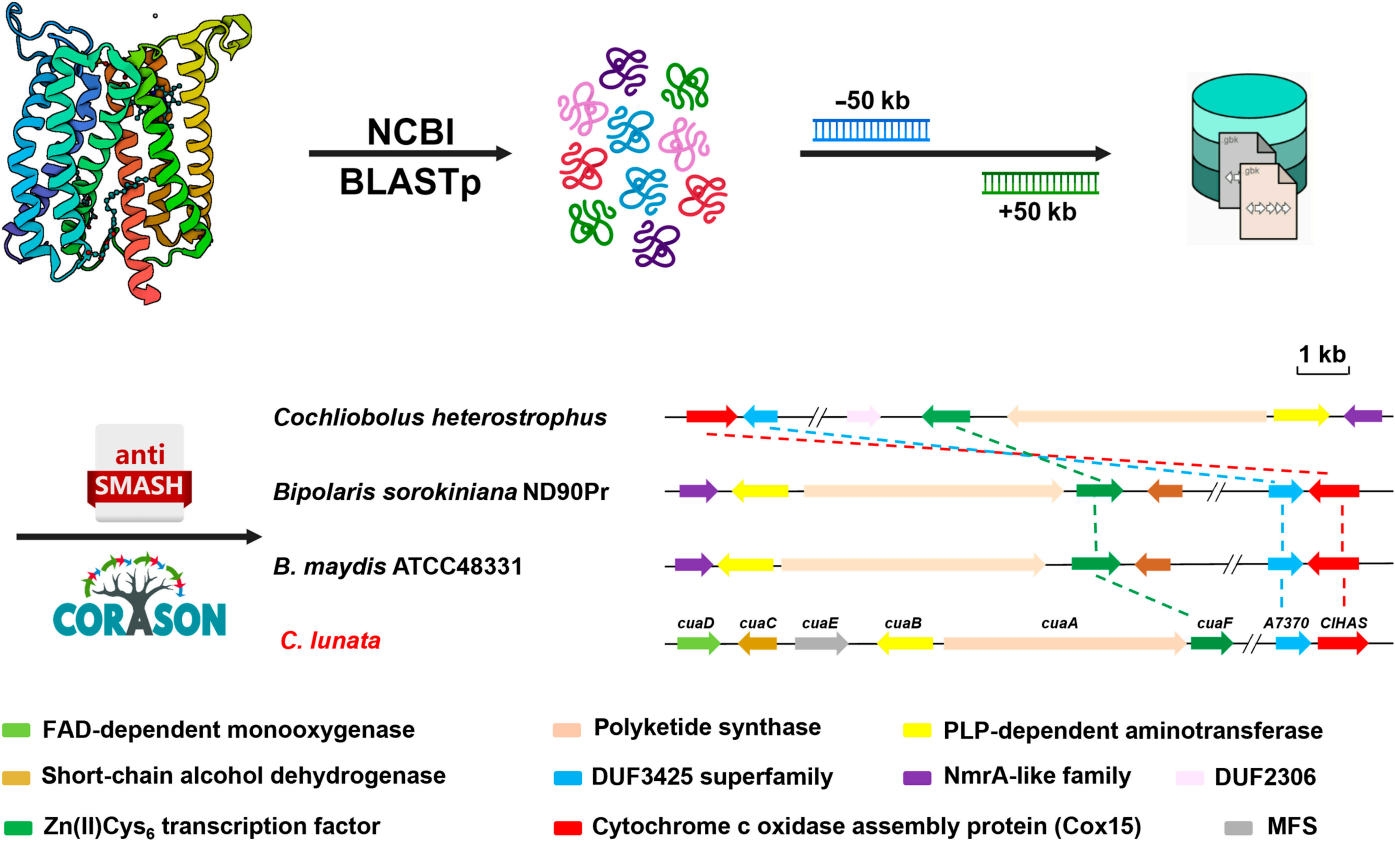
Genome mining workflow for the *ClHAS*, *A7370*, and *cuaF* homologs. The National Center for Biotechnology Information (NCBI) database was first mined to hit (>)5,000 *Cl*HAS homologs, which were further extended by 50 kb in the up- and downstream regions to capture all potential biosynthetic gene clusters (BGCs). Such hits were narrowed down by other 2 prerequisites of bearing an A7370 and CuaF homologous sequences. Subsequently, the antiSMASH database was mined using CORASON (CORe Analysis of Syntenic Orthologs to prioritize Natural Product-Biosynthetic Gene Clusters) platform to identify 3 BGCs homologous to the *cua* cluster from *Cochliobolus heterostrophus*, *Bipolaris sorokiniana*, and *B. maydis*. BLASTp, basic local alignment search tool protein; FAD, flavin adenosine dinucleotide; PLP, pyridoxal-5′-phosphate; MFS, major facilitator superfamily.

Beyond its canonical function in primary energy metabolism (Fig. 1C), *Cl*HAS, a Cox15-2 enzyme from *C. lunata*, functioned as a bridge between primary and secondary metabolism. We uncovered a substantial link between *Cl*HAS with A7370, a novel oxidative stress-responsive bZIP transcription factor. A7370 specifically regulates indolizidine alkaloid biosynthesis by binding to the promoter region of *cuaF*. To determine whether this regulatory relationship is species-specific or phylogenetically widespread, we performed genomic mining in the National Center for Biotechnology Information database using these protein sequences as probes. As expected, a search using *Cl*HAS alone as a probe returned a high abundance of homologs (>5,000 hits), reflecting the conserved role of Cox15 in respiration and revealing its presence across some BGCs (Fig. S21). Strikingly, when *Cl*HAS and A7370 were used together as dual probes, the search space narrowed considerably, only those BGCs containing both homologous proteins were identified, and these were exclusively associated with indolizidine alkaloid biosynthesis (Fig. S21). Consistent with this narrowing effect, a separate query using A7370 alone returned 1,972 homologs, all of which are functionally uncharacterized proteins. This prevalence of uncharacterized homologs indicates that A7370 represents a previously unrecognized family of fungal bZIP transcription factors (Fig. 5). We have thus defined a core regulatory triad composed of *Cl*HAS, A7370, and CuaF that governs indolizidine alkaloid biosynthesis in *C. lunata*. Importantly, this regulatory module is not unique to this species. When CuaF homologs were included as a phylogenetic filter, we observed that the 3 components co-occur in 3 other fungal genomes (Fig. 8), indicating the potential conservation of this regulatory architecture beyond *C. lunata*. These findings support a model in which fungal HAS may frequently contribute to secondary metabolism of indolizidine alkaloids, acting through a conserved mechanism that integrates upstream oxidative stress signaling via bZIP transcription factors with pathway-specific transcriptional activation in some fungi.

## Discussion

As noted earlier, HAS plays an essential role in primary metabolism. Its deficiency is known to cause hypertrophic cardiomyopathy in mammals [45] and disrupts aerobic respiration in yeast (*Saccharomyces cerevisiae*) [46]. In *C. lunata*, however, the ∆*ClHAS* mutant exhibited no markable difference in viability compared to the WT strain, presumably owing to compensatory mechanisms. Importantly, the disruption of the respiratory chain in the mutant led to elevated intracellular ROS levels. This increase in ROS consequently up-regulated the expression of bZIP-type transcription factors and various oxidoreductases. The resulting oxidative cellular environment drove the diversification of SMs, steering the curvulamine pathway toward the production of highly oxidized indolizidine analogs (Fig. 3). This mechanism not only aligns with the increased accumulation of pigment-like SMs in the mutant (Fig. S5A) but also considerably expands the repertoire of fungal indolizidine alkaloids.

As previously mentioned, compounds **1a**, **2**, and **4** represent early-stage indolizidine precursors with close biosynthetic relationships. Their elevated accumulations driven by the up-regulated expression of the *cua* gene cluster in the ∆*ClHAS* mutant enabled several shunt pathways to generate detectable amounts of new derivatives. The routes for the diversification of **2** include: acylation (i.e., formylation to yield curvamines A [**8**] and B [**9**], and acetylation to supply curvamines C [**10**] and D [**11**]); oxidation (to afford curvamines E [**12**] and F [**13**]); and heterodimerization with monomer **1a** (derived from **4** [Fig. S22]) into curvamine G (**14**). The observation reveals a new landscape for chemical convertibility of **2**, a possibility initially hinted at by the earlier identification of procurvamine (3,5-dimethylindolizin-8(5H)-one) from culture**s** of *C. lunata* [47]. Furthermore, the present characterization of **14** provides insight into the curvulamine biosynthetic pathway. Evidenced from the structural feature, we propose that the oxidation of **4** to **1a** is followed by the [2+5]-cycloaddition of **1a** with **2** to form intermediate **2a**. Subsequent dehydration of **2a** yields **2b**, a transformation presumably driven by the resulting extended conjugation. An intramolecular addition of **2b** then affords curvulamine. In this work, however, a very small fraction of **2b** became curvamine G (**14**) presumably via a double-bond migration process as suggested in other contexts [48]. Again, the *ClHAS* deletion strategy proved crucial, allowing us to characterize **14**, a minor curvulamine analog.

Curvulamine features 4 polarized double bonds within its pyrrole systems, with the most nucleophilic methine group capable of participating in Michael additions to *α,β*-unsaturated ketones [47]. Inactivation of *ClHAS* led to intracellular accumulation of curvulamine, consequently promoting the formation of arrays of new alkaloids. The structure of curvamine H (**15**) implies that curvulamine undergoes multistep hydration in the fungal culture (Fig. S23). The methine groups adjacent to the pyrrole ring are susceptible to oxidation, generating curvamines I (**16**) and J (**17**) (Fig. S23). Subsequent dimethylation of **17** yields curvamine K (**18**) (Fig. S23). The methyl groups on the pyrrole ring are also susceptible to oxidation, forming an alcohol (curvamine N [**21**]), aldehydes (curvamines L [**19**] and O [**22**]), or carboxylic acids. But the acid intermediates readily undergo decarboxylation, as indicated by the demethylated structures of curvamines M (**20**) and Q (**24**). Furthermore, alcohol **21** can be methylated to form the methyl ether, curvamine P (**23**). Unexpectedly, we characterized 4 dimers, curvamines R–U (**25**–**28**), from the ∆*ClHAS* culture. Structurally, curvamines R (**25**) and S (**26**) are diastereomers that result from the 2-way (pro-*S* and pro-*R*) addition of curvulamine to a carbocation (**A**). As reinforced by our acid-promotion test (Fig. S24), this carbocation A forms from the protonation of curvulamine and is obviously stabilized by conjugation with a pyrrole nucleus. Curvamines T (**27**) and U (**28**) are a pair of regioisomers formed respectively from the curvulamine attacks on exomethylene carbons of regioisomeric ammonium salts **B** and **C** (Fig. S8). These electrophilic intermediates, **B** and **C**, are generated through Diels–Alder addition of ^1^O₂ to each of the 2 pyrrole diene systems in curvulamine, followed by endoperoxide opening and release of H₂O₂ (Fig. S9). Their formations were further confirmed by trapping with *β*-mercaptoethanol (Fig. S10). Notably, the *ClHAS* inactivation induced overproduction of ^1^O₂, raising its concentration above the threshold required for the Diels–Alder reaction with curvulamine (Figs. S6 to S10). To our knowledge, the driving force formation for such cation-promoted dimerizations is distinct from reported counterparts from bacterial [49], fungal [50], and plant systems [51]. Finally, antibacterial assay allowed us to detect previously overlooked new antibacterial molecules—curvamines B (**9**) and P (**23**), and crucially they feature novel antibacterial pharmacophores with no precedent in existing or developmental antibacterial agents [3].

In summary, our machine learning analyses suggested that redox-driven couplings of nitrogen-containing monomers play a role in the biosynthesis of indolizidine alkaloids. We also identified an interaction between *Cl*HAS (a Cox15-2 homolog) and CuaA, a polyketide synthase that catalyzes the first key step in the production of these SMs in *C. lunata*. The findings allowed us to establish *Cl*HAS as a modulator of indolizidine alkaloid biosynthesis, thereby establishing a previously uncharacterized link between mitochondrial respiration and an indolizidine secondary metabolic regulatory axis involving 2 transcription factors: A7370, which responds to *ClHAS* inactivation-induced oxidative stress, and CuaF, which is specific to the indolizidine alkaloid biosynthetic pathway. We further characterized A7370 by demonstrating its selective binding to a new *cis*-element (ACGGCTGAC) in the *cuaF* promoter region. bZIP transcription factors are widely distributed in fungi [52] and plants [53] and have been reported to regulate secondary metabolism under oxidative stress conditions. However, previous studies have primarily focused on responses to exogenous oxidative stimuli. Here, we demonstrate for the first time that an internally triggered shift in the cellular redox microenvironment, resulting from respiratory chain disruption caused by *ClHAS* deletion, can activate a complete regulatory network bridging primary and secondary metabolism. This network features A7370 as a bZIP factor that appears to represent a broader class of functionally unannotated bZIP regulators inherent to, or evolutionarily acquired by, fungi as a mechanism to counteract oxidative insults. Through targeted gene mining, we found that this regulatory network specifically governs the production of indolizidine alkaloids. We anticipate that, over the course of evolution, such a network may have the capacity to orchestrate the biosynthesis of a broader array of SMs. In aggregation, the work presents a profound crosstalk between primary and secondary metabolism in fungi, triggered by HAS deletion, which led to enhanced indolizidine alkaloid precursor supply and oxidative stress. It thus provides a strategy for pushing the boundaries of secondary metabolic research and delivers 2 new bioactive indolizidine alkaloids that may be valuable for developing antibacterial agents.

## Materials and Methods

### Strains and cultivation conditions

*Curvularia lunata* IFB Z10 was isolated from the intestinal tract of the white croaker fish. The reidentification of strain was performed by Professor Wen Ying Zhuang from the Institute of Microbiology, Chinese Academy of Sciences. The WT and mutant strains were maintained on PDA (potato dextrose broth containing 2% agar) at 30 °C and stored on PDA slopes at 4 °C. All strains were cultivated at 30 °C and grown in liquid Czapek–Dox medium (CDM) (1 l: 2 g of soluble starch, 30 g of sucrose, 3 g of NaNO_3_, 1 g of K_2_HPO_4_, 1 g of yeast extract, 1 g of polypeptone, 20 g of maltose, 0.55 g of CaCl_2_, 0.5 g of MgSO_4_·7H_2_O, and 0.01 g of FeSO_4_·7H_2_O) for further related experiments. All *Escherichia coli* strains were cultured in lysogeny broth (LB) media with ampicillin (amp) antibiotic at 220 rpm, 37 °C.

### Fungal genomic DNA extraction

The WT and mutant strains of *C. lunata* were cultured at 30 °C for 3 d in yeast extract peptone dextrose medium (1 l: 20 g of sucrose, 10 g of yeast extract, 10 g of polypeptone, and 10 g of casein hydrolyzate). The mycelia collected by filtration were frozen in liquid nitrogen and crushed using a refiner. The subsequent operations were carried out in accordance with the procedure of Rapid Fungal Genomic DNA Isolation Kit (Sangon Biotech (Shanghai) Co., Ltd., China). The genomic DNA concentration was determined by Thermo Fisher Scientific U-3000 NanaDrop 2000C.

### DNA manipulation and plasmid construction

The primers used for constructing plasmids are listed in Table S2. Then, PCR amplifications were then performed on a Bio-Rad S1000 Thermal Cycler using Phanta Max Super-Fidelity DNA Polymerase (Nanjing Vazyme Biotech Co., Ltd., China). The concentrations of DNA fragments were measured using the NanoDrop 2000C. The gene fragments utilized for gene knockout and overexpression were amplified from genomic DNA and cloned into the plasmid puc19 between the BamHI and HindIII sites. The ClonExpress MultiS One Step Cloning Kit (Nanjing Vazyme Biotech Co., Ltd., China) was used for DNA cloning into plasmids. The plasmids were extracted from the corresponding *E. coli* (DH5α) strains using the SanPrep Column Plasmid MiniPreps Kit (Sangon Biotech (Shanghai) Co., Ltd., China), with sequence identity confirmed by General Biosystems (Anhui) Co., Ltd., China.

### Gene deletion and overexpression

Briefly, the *C. lunata* mycelia were initially cultured on PDA for 3 d, which were transferred to yeast extract peptone dextrose medium and incubated at 30 °C with shaking at 140 rpm for 10 h to induce germination. The germlings were collected by centrifugation and digested with 60 mg/ml lysing enzyme (Sigma-Aldrich, L1412) and 30 mg/ml driselase enzyme (Sigma-Aldrich, D9515) for 2 h at 30 °C to obtain protoplasts. The protoplasts were collected by centrifugation at 4,000 rpm for 15 min, washed with 0.6 M KCl solution, and then treated with STC buffer (1 l: 182.2 g of sorbitol, 10 ml of tris-HCl [1 M], and 5.5 g of CaCl_2_). The protoplasts were then transformed using polyethylene glycol (PEG)-mediated strategy [54] with the 2 split-marker knockout cassettes, generating by fusion PCR and *hph* phosphotransferase gene used as selectable marker (Figs. S3, S12, S14, and S15). The primers in Table S2 were utilized to construct the upstream, downstream and *hph* fragments used for fusion PCR. The transformed protoplasts were grown in regeneration medium (REG) (1 l: 14.6 g of mannitol, 0.4 g of yeast extract, and 0.1 g of soluble starch) for overnight culture at 30 °C and poured onto the PDA plate supplemented with 100 μg/ml of *hph*. Subsequently, the mutant strains were diagnosed by PCR using primer pairs diagnostic F/R listed in Table S2.

### Total RNA extraction and RT-qPCRanalysis

After a 4-d cultivation in CDM, the mycelia of WT and *ΔClHAS* strains were collected. The total RNA was extracted from the crushed mycelia following the manufacturer’s instruction of the TRIzol Reagent (Ambion). DNA contamination was eliminated by ribonuclease-free deoxyribonuclease I (Thermo Fisher Scientific). The total RNA concentration was determined by NanaDrop 2000C. Then, the real-time quantitative PCR for complementary DNA (cDNA) synthesis was performed using 5 μg of total RNA as the template with Oligo-dT primers according to the protocol of Hifair III 1st Strand cDNA Synthesis SuperMix for qPCR (cat. no. 11141ES60; Yeasen, Shanghai, China). The mRNA expression levels of genes were tested on the StepOnePlus Real-Time PCR System (Applied Biosystems) and calculated using the 2*^−ΔΔCt^* method. The gene of actin was used as a reference. Primers used for amplifying target genes are listed in Table S3. Each set of data was repeated 3 times.

### RNA interference-mediated silencing of *A7370* gene

The strategy implemented for constructing si-*A7370* mutant of *C. lunata* involved introducing double-stranded RNA to trigger A7370-specific degradation as described by Hitoshi Nakayashiki [55]. Primers 7370-pS-F/R (Table S4) were used for PCR amplification of *A7370* fragment that was inserted into the vector pSilent-Dual1-RNAi at the HindIII and EcoRI sites, located between the 2 opposite constitutive promoters (*gpdA* and *trpC*). The plasmid pSilent-Dual1 was obtained from HonorGene (Changsha, China). Subsequently, the recombinant plasmid was introduced into *C. lunata* protoplasts using the PEG-mediated transformation method [54]. The obtained silencing mutants were utilized for fermentation validation.

### RNA sequencing and transcriptomic data analysis

The mycelia of WT and *ΔClHAS* strains collected after a 4-d cultivation in CDM were utilized for transcriptome sequencing. Total RNA was isolated and subjected to RNA sequencing with a global transcriptomic analysis. The cDNA libraries were sequenced on the Illumina NovaSeq 6000 sequencing platform at Benagen Technology (Wuhan, China). A total of 22 to 30 million reads were obtained per replicate, each read with an average length of 75 bp. At the chosen time point, 41% of the genome was found to be expressed in WT strain. Three biological replicates of each WT and *ΔClHAS* strains were cultured to early stationary phase. The data were analyzed by determining gene expression levels for all observed transcripts that were mapped onto the sequenced *C. lunata* genome. Relative to WT strain, the top 177 up-regulated and top 179 down-regulated genes in the *ΔClHAS* mutant are subjected to Gene Ontology and Kyoto Encyclopedia of Genes and Genomes classification, followed by clustering analysis in Fig. S6, respectively. In agreement with the metabolomic data, we observed major inductions of up-regulated expression of 44 genes and down-regulated expression of 22 genes encoding oxidoreductases (|log_2_fold change| > 2) in the *ΔClHAS* mutant, respectively (Fig. 4B).

### Quantification of ROS

The method described by Miranda et al. [37] was used with some modifications for the ROS quantification in mycelia from WT and *ΔClHAS* strains. After 3 to 7 d of cultivations in CDM, 100 μg of mycelia of WT and *ΔClHAS* strains was collected. Each sample was homogenized and added to 10 μM H_2_DCF-DA (2′,7′-dichlorodihydrofuorescein diacetate) in 1 ml of phosphate-buffered saline (PBS) cold buffer. The reactions were incubated in the dark for 40 min on an ice bath, with the blank controls treated in 1 ml of PBS cold buffer without H_2_DCF-DA. The samples were vortexed for 20 s and centrifuged at 12,000 × g at 4 °C for 15 min. For each sample, 200 μl of supernatant was put in a 96-well microplate, and the absorbance reading was adjusted to 485-nm excitation and 530-nm emission in a DTX 880 multimode plate reader (Beckman Coulter). The obtained signal was corrected by subtracting the background signal and normalized with the biomass (dry weight). All data were obtained from 3 replicates.

### Construction of A7370-3×Flag tag strains

The protoplasts were collected by the method described in the “Gene deletion and overexpression” section. They were then transformed using PEG-mediated strategy [54] with the 2 split-marker cassettes. The fragments were constructed by deleting the stop codon of A7370, followed by the addition of a 3×Flag tag and AmyB terminator (Fig. S16). For the upstream and downstream cassettes, the fragments were cloned into pcDNA3.1 and pUC19, respectively, with *hph* phosphotransferase gene serving as the selection marker. Subsequently, the mutant strains were diagnosed by PCR using primer pairs diagnostic F/R listed in Table S5.

### ChIP-seq assay, library preparation, and sequencing

Fungal conidia of mutants with 3×Flag tag were inoculated into CDM at 28 °C for 4 d. Then, the filtered mycelia (a total of 2 × 10^7^ cells) were washed with PBS and cross-linked with a 1% formaldehyde solution for 10 min at room temperature. Cross-linking was quenched with 0.125 M glycine for 5 min, after which the cells were pelleted and washed twice with PBS. The cell pellets were lysed on ice for 5 min using a cell lysis buffer containing 10 mM Hepes (pH 7.5), 0.1 mM EDTA, 0.5% NP-40, and a protease inhibitor cocktail. Nuclei were collected by centrifugation at 2,000 × g for 10 min at 4 °C, and chromatin was sheared to an average size of 100 to 500 bp using sonication. Of the sonicated chromatin, 10% was reserved and labeled as “input”, while 80% was utilized for immunoprecipitation reactions with a specific anti-flag antibody (Sigma f1804). An additional 10% was incubated with rabbit immunoglobulin G (Abcam, ab171870) as a negative control and labeled as “IgG”. The immunoprecipitated complexes were subjected to a series of washes, eluted, and de-crosslinked. DNA from both input and IP fractions (Fig. S17A) was extracted using the phenol–chloroform method, quantified using Qubit 3 fluorometer (Thermo Fisher). Then, the degree of chromosomal fragmentation was assessed by detecting input DNA via agarose gel electrophoresis. The success of the immunoprecipitation was further evaluated by Western blot analysis (Fig. S17B). For high-throughput DNA sequencing, libraries were prepared using the VAHTS Universal DNA Library Prep Kit for MGI (Vazyme, China, cat. no. NDM607). Library products corresponding to fragments ranging from 200 to 500 bp were enriched and quantified. Sequencing was performed on a DNBSEQ-T7 (MGI) using the PE150 sequencing mode to generate paired-end reads. Immunoprecipitated DNA was sequenced on an Illumina NovaSeq 6000 platform in Seqhealth Technology Co. Ltd. (Wuhan, China).

### Expression and purification of A7370

Recombinant A7370 (marked with an MBP tag) was expressed in *E. coli* BL21(DE3) transformed with plasmid pMCSG9-MBP-A7370. Cells were grown at 37 °C in LB medium containing 100 μg/ml amp until the optical density at 600 nm value reached 0.6. Then, the expression of *A7370* was induced by adding 0.2 mM isopropyl *β*-D-1-thiogalactopyranoside, and cells were cultured at 25 °C for 3 h. Cells were harvested by centrifugation (4,000 × g for 20 min) and resuspended in lysis buffer (1 l: 11.7 g of NaCl, 0.34 g of imidazole, and 12.5 ml of tris-HCl [2 M]), followed by ultrasonic homogenizer. After centrifugation to remove cell debris, the supernatant was filtered and loaded onto a nickel-nitrilotriacetic acid column (Qiagen, Valencia, CA) for affinity purification. The recombinant MBP-A7370 protein was eluted using a linear gradient of imidazole solution (from 50 to 800 mM), and fractions containing the protein were analyzed by sodium dodecyl sulfate-polyacrylamide gel electrophoresis to collect highly pure samples (Fig. S16).

### EMSA

Probes for EMSA were obtained either by PCR amplification when the length exceeded 70 bp or by annealing of 2 complementary oligos when the length was below 60 bp. Labeling was performed with 5'-FAM. For annealing, the oligos were mixed and denatured for 3 min at 90 °C, followed by gradual cooling at 1 °C/min to their *T*_m_, held for 30 min and finally cooled to room temperature. The reactions were prepared by gently mixing the components in this order: PBS buffer (100 mM), 1 μl; labeled probe (10 pmol), 1.3 μl; purified MBP-A7370 (348 μg), 3.4 μl; KCl (100 mM), 1 μl; salmon sperm (12 μg), 1 μl. The reactions were incubated at 28 °C for 25 min, then mixed with 1 μl of loading buffer (30% glycerin with 30 mM of NaCl, 10×), and loaded onto the gel. A 5% native polyacrylamide gel was made as follows: mixing of H_2_O, 29.5 ml; tris-borate-EDTA buffer 10×, 5 ml; acrylamide-bisacrylamide (29:1) at 45%, 5.5 ml; stirred for 1 min, and addition of ammonium persulfate substitute 5% (400 μl) and tetramethylethylenediamine, 36 μl. Finally, the gel was casted in a Mini-Protean Tetra Cell (Bio-Rad) and left to polymerize and run for 40 to 60 min at 110 V with cooled tris-borate-EDTA buffer 0.5×.

### Antibacterial assay

A classical but believable disc diffusion assay was used to determine antibacterial activities of the obtained alkaloids, against *Veillonella parvula*, *Actinomyces israelii*, *Bacteroides vulgatus*, *Streptococcus pneumoniae*, and *Peptostreptococcus micros*. These target bacterial pathogens were cultivated in LB medium at 37 °C (220 rpm) until optical density at 600 nm value reached 0.5, followed by mixing 1 ml of culture with 100 ml of LB agar medium for solid LB plates. All test samples were dissolved in dimethyl sulfoxide at 10 mM. Tinidazole was used as the positive control. The prepared solutions (5 μl) were diluted gradiently and applied to 6-mm paper disks. The disks were then placed onto solid LB agar mixed with bacterial culture, and these plates were incubated overnight at 37 °C. The MIC of compounds was identified by the lowest concentration that prevents visible bacterial growth.

## Data Availability

The datasets used and/or analyzed in the current study are available from the corresponding author on reasonable request. The nucleic acid sequences of *ClHAS* and *A7370* have been submitted to the NCBI database, and their corresponding GenBank accession numbers are PZ085902 and PZ085903, respectively.
