# Supplementary material for: From Respiration to Secondary Metabolism: A Heme A Synthase-Mediated Regulatory Network Expands Indolizidine Chemical Space in Fungi
Source: Research (Wash D C). 2026 Apr 27;9:1236. doi: 10.34133/research.1236 (PMC13113314; doi:10.34133/research.1236)
Supplement: Supplementary 1 — Supplementary Text Tables S1 to S27 Figs. S1 to S166 [file research.1236.f1.zip › SI_2026 02 26.pdf]

Supplementary Materials

Wei Bai[1], Guangzhi Dai[2], Junyang Ji[1], Qi Du[1], Meiling Ding[1], Wenbo Han[2], Hao Zhu[1], Xincun Wang[3], Wenying Zhuang[3], and Renxiang Tan[1,2]*

[1]Synthetic Biology Center for Chinese Medicine, School of Pharmacy, Nanjing University of Chinese Medicine, Nanjing 210023, P. R. China
[2]State Key Laboratory of Pharmaceutical Biotechnology, Institute of Functional Biomolecules, Nanjing University, Nanjing 210023, P. R. China
[3]State Key Laboratory of Mycology, Institute of Microbiology, Chinese Academy of Sciences, Beijing 100101, P. R. China

*Address correspondence to: Renxiang Tan; rxtan@nju.edu.cn

Experimental Procedures 7
General experimental procedures 7
Fermentation, extraction, and HPLC analysis 7
Whole-genome sequencing and analysis 7
Physicochemical data of compounds 8
Structural determination of new compounds 10
Supplementary tables 14
Table S1. ROS and their production inside cells 14
Table S2. Primers used in the study for gene deletion and overexpression. 15
Table S3. Primers used in this study for RT-qPCR analysis. 16
Table S4. Primers used in this study for RNAi-mediated silencing. 17
Table S5. Primers used in this study for Chip-seq assay. 18
Table S6. Oligonucleotides utilized for gel shift assay. 19
Table S7. ^{1}H and ^{13}C NMR data of curvamine A (**8**). 20
Table S8. ^{1}H and ^{13}C NMR data of curvamine B (**9**). 21
Table S9. ^{1}H and ^{13}C NMR data of curvamine C (**10**). 22
Table S10. ^{1}H and ^{13}C NMR data of curvamine D (**11**). 23
Table S11. ^{1}H and ^{13}C NMR data of curvamine E (**12**) ($CDCl_3$). 24
Table S12. ^{1}H and ^{13}C NMR data of curvamine F (**13**) ($CDCl_3$). 25
Table S13. ^{1}H and ^{13}C NMR data of curvamine G (**14**). 26
Table S14. ^{1}H and ^{13}C NMR data of curvamine H (**15**). 27
Table S15. ^{1}H and ^{13}C NMR data of curvamine I (**16**). 28
Table S16. ^{1}H and ^{13}C NMR data of curvamine J (**17**). 29
Table S17. ^{1}H and ^{13}C NMR data of curvamine K (**18**). 30
Table S18. ^{1}H and ^{13}C NMR data of curvamine L (**19**) ($CDCl_3$). 31
Table S19. ^{1}H and ^{13}C NMR data of curvamine M (**20**) ($CDCl_3$). 32
Table S20. ^{1}H and ^{13}C NMR data of curvamine N (**21**). 33
Table S21. ^{1}H and ^{13}C NMR data of curvamine O (**22**). 34
Table S22. ^{1}H and ^{13}C NMR data of curvamine P (**23**). 35
Table S23. ^{1}H and ^{13}C NMR data of curvamine Q (**24**). 36
Table S24. ^{1}H and ^{13}C NMR data of curvamine R (**25**) ($CDCl_3$). 37
Table S25. ^{1}H and ^{13}C NMR data of curvamine S (**26**) ($CDCl_3$). 39
Table S26. ^{1}H and ^{13}C NMR data of curvamine T (**27**) ($CDCl_3$). 41
Table S27. ^{1}H and ^{13}C NMR data of curvamine U (**28**) ($CDCl_3$). 43
Supplementary Figures 45
Fig. S1. The bioretrosynthesis prediction of nitrogenated monomers **1** (A) and **2** (B) by BioNavi-NP.. 45
Fig. S2. The Cox15 proteins catalyze the heme O conversion into heme A. 45
Fig. S3. Construction of *ΔClHAS* mutant of *C. lunata*. 46
Fig. S4. The analysis of hemes B and O components in *ΔClHAS* and WT strains. 46
Fig. S5. The fermentation broth and HPLC profiles of *ΔClHAS* and WT strains in different media.. 47
Fig. S6. The analysis of RNA-seq data from the Δ*ClHAS* and WT strains. 47
Fig. S7. The difference in the $^{1}O_2$ content between *ΔClHAS* and WT strains cultured for 4 days. 48
Fig. S8. The proposed formation process of curvamine T (**27**) and curvamine U (**28**). 48

Fig. S9. Measurement of the H_2O_2 content. 49
Fig. S10. LC-HR/MS detection of adducts **1** and **2** resulting from the addition of curvulamine with *β*-mercaptoethanol. 49
Fig. S11. RT-qPCR analysis of gene expressions associated with indolizidine alkaloid biosynthesis in WT and *ΔClHAS* strains after a 4-day cultivation. 50
Fig. S12. Construction of the OE::*cuaF* strain of *C. lunata*. 50
Fig. S13. HPLC profiles of the WT, OE::*A7370*, si-*A7370*, OE::*cuaF*, Δ*cuaF*, and Δ*ClHAS* strains after an 11-day fermentation in CDM. 51
Fig. S14. Construction of the *ΔcuaF* mutant of *C. lunata*. 51
Fig. S15. Construction of the OE::*A7370* strain of *C. lunata*. 52
Fig. S16. Construction of the *A7370*-3×*Flag* tag strain of *C. lunata*. 52
Fig. S17. ChIP-seq sample verification. 52
Fig. S18. ChIP-seq assay showing the genes regulated by A7370. 53
Fig. S19. The SDS-PAGE of recombinant A7370 (marked with a mycelin basic protein (MBP) tag). 54
Fig. S20. Optimization of the binding of A7370 with ACGGCTGAC. 54
Fig. S21. Genome mining of *Cl*HAS homologs-containing BGCs. 55
Fig. S22. Proposed **4**-to-**1a** transformation. 55
Fig. S23. The proposed biosynthetic pathways of curvamines H–K (**15**–**18**). 55
Fig. S24. The acid-promoted formation of curvamines R (**25**) and S (**26**) from curvulamine. 56
Fig. S25. ^{1}H NMR spectrum of curvamine A (**8**) (500 MHz). 56
Fig. S26. ^{13}C NMR spectrum of curvamine A (**8**) (125 MHz). 57
Fig. S27. ^{1}H-^{1}H COSY spectrum of curvamine A (**8**). 57
Fig. S28. HSQC spectrum of curvamine A (**8**). 58
Fig. S29. HMBC spectrum of curvamine A (**8**). 58
Fig. S30. NOESY spectrum of curvamine A (**8**). 59
Fig. S31. ^{1}H NMR spectrum of curvamine B (**9**) (500 MHz). 59
Fig. S32. ^{13}C NMR spectrum of curvamine B (**9**) (125 MHz). 60
Fig. S33. ^{1}H-^{1}H COSY spectrum of curvamine B (**9**). 60
Fig. S34. HSQC spectrum of curvamine B (**9**). 61
Fig. S35. HMBC spectrum of curvamine B (**9**). 61
Fig. S36. NOESY spectrum of curvamine B (**9**). 62
Fig. S37. ^{1}H NMR spectrum of curvamine C (**10**) (600 MHz). 62
Fig. S38. ^{13}C NMR spectrum of curvamine C (**10**) (150 MHz). 63
Fig. S39. ^{1}H-^{1}H COSY spectrum of curvamine C (**10**). 63
Fig. S40. HSQC spectrum of curvamine C (**10**). 64
Fig. S41. HMBC spectrum of curvamine C (**10**). 64
Fig. S42. NOESY spectrum of curvamine C (**10**). 65
Fig. S43. ^{1}H NMR spectrum of curvamine D (**11**) (400 MHz). 65
Fig. S44. ^{13}C NMR spectrum of curvamine D (**11**) (100 MHz). 66
Fig. S45. ^{1}H-^{1}H COSY spectrum of curvamine D (**11**). 66
Fig. S46. HSQC spectrum of curvamine D (**11**). 67
Fig. S47. HMBC spectrum of curvamine D (**11**). 67
Fig. S48. NOESY spectrum of curvamine D (**11**). 68
Fig. S49. ^{1}H NMR spectrum of curvamine E (**12**) (500 MHz, $CDCl_3$). 68

Fig. S50. ^{13}C NMR spectrum of curvamine E (**12**) (125 MHz, $CDCl_3$). 69
Fig. S51. ^1H-^1H COSY spectrum of curvamine E (**12**) in $CDCl_3$. 69
Fig. S52. HSQC spectrum of curvamine E (**12**) in $CDCl_3$. 70
Fig. S53. HMBC spectrum of curvamine E (**12**) in $CDCl_3$. 70
Fig. S54. NOESY spectrum of curvamine E (**12**) in $CDCl_3$. 71
Fig. S55. ^1H NMR spectrum of curvamine F (**13**) (500 MHz, $CDCl_3$). 71
Fig. S56. ^{13}C NMR spectrum of curvamine F (**13**) (125 MHz, $CDCl_3$). 72
Fig. S57. ^1H-^1H COSY spectrum of curvamine F (**13**) in $CDCl_3$. 72
Fig. S58. HSQC spectrum of curvamine F (**13**) in $CDCl_3$. 73
Fig. S59. HMBC spectrum of curvamine F (**13**) in $CDCl_3$. 73
Fig. S60. NOESY spectrum of curvamine F (**13**) in $CDCl_3$. 74
Fig. S61. ^1H NMR spectrum of curvamine G (**14**) (500 MHz). 74
Fig. S62. ^{13}C NMR spectrum of curvamine G (**14**) (125 MHz). 75
Fig. S63. ^1H-^1H COSY spectrum of curvamine G (**14**). 75
Fig. S64. HSQC spectrum of curvamine G (**14**). 76
Fig. S65. HMBC spectrum of curvamine G (**14**). 76
Fig. S66. NOESY spectrum of curvamine G (**14**). 77
Fig. S67. ^1H NMR spectrum of curvamine H (**15**) (500 MHz). 77
Fig. S68. ^{13}C NMR spectrum of curvamine H (**15**) (125 MHz). 78
Fig. S69. ^1H-^1H COSY spectrum of curvamine H (**15**). 78
Fig. S70. HSQC spectrum of curvamine H (**15**). 79
Fig. S71. HMBC spectrum of curvamine H (**15**). 79
Fig. S72. NOESY spectrum of curvamine H (**15**). 80
Fig. S73. ^1H NMR spectrum of curvamine I (**16**) (500 MHz). 80
Fig. S74. ^{13}C NMR spectrum of curvamine I (**16**) (125 MHz). 81
Fig. S75. ^1H-^1H COSY spectrum of curvamine I (**16**). 81
Fig. S76. HSQC spectrum of curvamine I (**16**). 82
Fig. S77. HMBC spectrum of curvamine I (**16**). 82
Fig. S78. NOESY spectrum of curvamine I (**16**). 83
Fig. S79. ^1H NMR spectrum of curvamine J (**17**) (500 MHz). 83
Fig. S80. ^{13}C NMR spectrum of curvamine J (**17**) (125 MHz). 84
Fig. S81. ^1H-^1H COSY spectrum of curvamine J (**17**). 84
Fig. S82. HSQC spectrum of curvamine J (**17**). 85
Fig. S83. HMBC spectrum of curvamine J (**17**). 85
Fig. S84. NOESY spectrum of curvamine J (**17**). 86
Fig. S85. ^1H NMR spectrum of curvamine K (**18**) (500 MHz). 86
Fig. S86. ^{13}C NMR spectrum of curvamine K (**18**) (125 MHz). 87
Fig. S87. ^1H-^1H COSY spectrum of curvamine K (**18**). 87
Fig. S88. HSQC spectrum of curvamine K (**18**). 88
Fig. S89. HMBC spectrum of curvamine K (**18**). 88
Fig. S90. NOESY spectrum of curvamine K (**18**). 89
Fig. S91. ^1H NMR spectrum of curvamine L (**19**) (500 MHz, $CDCl_3$). 89
Fig. S92. ^{13}C NMR spectrum of curvamine L (**19**) (125 MHz, $CDCl_3$). 90
Fig. S93. ^1H-^1H COSY spectrum of curvamine L (**19**) in $CDCl_3$. 90

Fig. S94. HSQC spectrum of curvamine L (**19**) in $CDCl_3$. 91
Fig. S95. HMBC spectrum of curvamine L (**19**) in $CDCl_3$. 91
Fig. S96. NOESY spectrum of curvamine L (**19**) in $CDCl_3$. 92
Fig. S97. ^1H NMR spectrum of curvamine M (**20**) (500 MHz, $CDCl_3$). 92
Fig. S98. ^{13}C NMR spectrum of curvamine M (**20**) (125 MHz, $CDCl_3$). 93
Fig. S99. ^1H-^1H COSY spectrum of curvamine M (**20**) in $CDCl_3$. 93
Fig. S100. HSQC spectrum of curvamine M (**20**) in $CDCl_3$. 94
Fig. S101. HMBC spectrum of curvamine M (**20**) in $CDCl_3$. 94
Fig. S102. NOESY spectrum of curvamine M (**20**) in $CDCl_3$. 95
Fig. S103. ^1H NMR spectrum of curvamine N (**21**) (500 MHz). 95
Fig. S104. ^{13}C NMR spectrum of curvamine N (**21**) (125 MHz). 96
Fig. S105. ^1H-^1H COSY spectrum of curvamine N (**21**). 96
Fig. S106. HSQC spectrum of curvamine N (**21**). 97
Fig. S107. HMBC spectrum of curvamine N (**21**). 97
Fig. S108. NOESY spectrum of curvamine N (**21**). 98
Fig. S109. ^1H NMR spectrum of curvamine O (**22**) (500 MHz). 98
Fig. S110. ^{13}C NMR spectrum of curvamine O (**22**) (125 MHz). 99
Fig. S111. ^1H-^1H COSY spectrum of curvamine O (**22**). 99
Fig. S112. HSQC spectrum of curvamine O (**22**). 100
Fig. S113. HMBC spectrum of curvamine O (**22**). 100
Fig. S114. NOESY spectrum of curvamine O (**22**). 101
Fig. S115. ^1H NMR spectrum of curvamine P (**23**) (500 MHz). 101
Fig. S116. ^{13}C NMR spectrum of curvamine P (**23**) (125 MHz). 102
Fig. S117. ^1H-^1H COSY spectrum of curvamine P (**23**). 102
Fig. S118. HSQC spectrum of curvamine P (**23**). 103
Fig. S119. HMBC spectrum of curvamine P (**23**). 103
Fig. S120. NOESY spectrum of curvamine P (**23**). 104
Fig. S121. ^1H NMR spectrum of curvamine Q (**24**) (400 MHz). 104
Fig. S122. ^{13}C NMR spectrum of curvamine Q (**24**) (100 MHz). 105
Fig. S123. ^1H-^1H COSY spectrum of curvamine Q (**24**). 105
Fig. S124. HSQC spectrum of curvamine Q (**24**). 106
Fig. S125. HMBC spectrum of curvamine Q (**24**). 106
Fig. S126. NOESY spectrum of curvamine Q (**24**). 107
Fig. S127. ^1H NMR spectrum of curvamine R (**25**) (500 MHz, $CDCl_3$). 107
Fig. S128. ^{13}C NMR spectrum of curvamine R (**25**) (125 MHz, $CDCl_3$). 108
Fig. S129. ^1H-^1H COSY spectrum of curvamine R (**25**) in $CDCl_3$. 108
Fig. S130. HSQC spectrum of curvamine R (**25**) in $CDCl_3$. 109
Fig. S131. HMBC spectrum of curvamine R (**25**) in $CDCl_3$. 109
Fig. S132. NOESY spectrum of curvamine R (**25**) in $CDCl_3$. 110
Fig. S133. ^1H NMR spectrum of curvamine S (**26**) (500 MHz, $CDCl_3$). 110
Fig. S134. ^{13}C NMR spectrum of curvamine S (**26**) (125 MHz, $CDCl_3$). 111
Fig. S135. ^1H-^1H COSY spectrum of curvamine S (**26**) in $CDCl_3$. 111
Fig. S136. HSQC spectrum of curvamine S (**26**) in $CDCl_3$. 112
Fig. S137. HMBC spectrum of curvamine S (**26**) in $CDCl_3$. 112

Fig. S138. NOESY spectrum of curvamine S (**26**) in $CDCl_3$. 113
Fig. S139. ^{1}H NMR spectrum of curvamine T (**27**) (500 MHz, $CDCl_3$). 113
Fig. S140. ^{13}C NMR spectrum of curvamine T (**27**) (125 MHz, $CDCl_3$). 114
Fig. S141. ^{1}H-^{1}H COSY spectrum of curvamine T (**27**) in $CDCl_3$. 114
Fig. S142. HSQC spectrum of curvamine T (**27**) in $CDCl_3$. 115
Fig. S143. HMBC spectrum of curvamine T (**27**) in $CDCl_3$. 115
Fig. S144. NOESY spectrum of curvamine T (**27**) in $CDCl_3$. 116
Fig. S145. ^{1}H NMR spectrum of curvamine U (**28**) (500 MHz, $CDCl_3$). 116
Fig. S146. ^{13}C NMR spectrum of curvamine U (**28**) (125 MHz, $CDCl_3$). 117
Fig. S147. ^{1}H-^{1}H COSY spectrum of curvamine U (**28**) in $CDCl_3$. 117
Fig. S148. HSQC spectrum of curvamine U (**28**) in $CDCl_3$. 118
Fig. S149. HMBC spectrum of curvamine U (**28**) in $CDCl_3$. 118
Fig. S150. NOESY spectrum of curvamine U (**28**) in $CDCl_3$. 119
Fig. S151. ECD spectra recorded and calculated for curvamine A (**8**). 119
Fig. S152. ECD spectra recorded and calculated for curvamine E (**12**). 120
Fig. S153. ECD spectra recorded and calculated for curvamine F (**13**). 120
Fig. S154. ECD spectra recorded and calculated for curvamine G (**14**). 121
Fig. S155. ECD spectra recorded and calculated for curvamine H (**15**). 121
Fig. S156. ECD spectra recorded and calculated for curvamine I (**16**). 122
Fig. S157. ECD spectral analysis of curvamine J (**17**). 122
Fig. S158. ECD spectral comparison between curvamine L (**19**) and curvulamine. 123
Fig. S159. ECD spectral comparison between curvamine M (**20**) and curvulamine. 123
Fig. S160. ECD spectral comparison between curvamine N (**21**) and curvulamine. 123
Fig. S161. ECD spectral comparison between curvamine P (**23**) and curvulamine. 124
Fig. S162. ECD spectra recorded and calculated for curvamine R (**25**). 124
Fig. S163. ECD spectra recorded and calculated for curvamine S (**26**). 125
Fig. S164. ECD spectral comparison between curvamine T (**27**) and curvulamine. 125
Fig. S165. ECD spectral comparison between curvamine U (**28**) and curvulamine. 125
Fig. S166. Single crystal X-ray diffractions of curvamine B (**9**), curvamine C (**10**), curvamine D (**11**), and curvamine Q (**24**). 126

Experimental Procedures

General experimental procedures

The NMR spectra were acquired in acetone-d_6 (unless indicated otherwise) on a Bruker Avance 400 spectrometer operating at 400 MHz for ^{1}H and 100 MHz for ^{13}C nuclei, a Bruker Avance 500 spectrometer at 500 MHz for ^{1}H and 125 MHz for ^{13}C nuclei and a Bruker Avance 600 spectrometer at 600 MHz for ^{1}H and 150 MHz for ^{13}C nuclei. HRESIMS analysis was performed on an Agilent 6530 TOF LC/MS mass spectrometer equipped with a Poroshell 120 EC-C_{18} column (4.6×50 mm, 2.7 μm). Medium-pressure liquid chromatography (MPLC) was conducted on a Biotage Isolera One MPLC system using a Biotage SNAP Cartridge Ultra C_{18} 60 g column, and semipreparative RP-HPLC on an Agilent 1200 HPLC system with an Agilent Eclipse XDB-C18 column (5 μm, 250×9.4 mm). The HPLC analysis was accomplished on an Agilent 1200 Infinity HPLC system equipped with a Poroshell 120 EC-C_{18} column (4.6×50 mm, 2.7 μm).

Fermentation, extraction, and HPLC analysis

Fresh spores of mutant and WT strains of *C. lunata* were inoculated into CDM for fermentation. The transformant strain (OE::*cuaF* and OE::*A7370*) was cultivated for 6–11 days in CDM with extra 2% maltose CDM to induce the expression of desired gene *cuaF* under *amyB* promoter. The mutants subjected to genes knockout (Δ*ClHAS* and Δ*cuaF*) and knockdown (si-*A7370*) were cultivated in CDM for 6–11 days. After fermentation, the mycelia were filtered and ethyl acetate was then added to the cultures and shaken thoroughly. The ethyl acetate extract layer was collected and evaporated *in vacuo*. The obtained extract was directly used for HPLC analysis (10 μL injected). The column was initially eluted with a linear gradient of 10% to 90% acetonitrile (MeCN) in water within 20 min, followed by 100% MeCN for 3 min at 0.5 mL/min.

Whole-genome sequencing and analysis

The whole-genome sequencing of *C. lunata* was performed by BGI (Shenzhen, China) with an Illumina HiSeq 2000 platform. The SOAP de novo version 1.05 (http://soap.genomics.org.cn/soapdenovo.html) sequence assembly system was used to produce 196 contigs, covering 35.6 Mb. The open reading frames were predicted by Augustus (http://bioinf.uni-greifswald.de/webaugustus/). The protein functions was further analyzed by 2ndFind (http://biosyn.nih.go.jp/2ndfind/), antiSMASH (fungal version) (https://fungismash.secondarymetabolites.org) [58] and Basic Local Alignment Search Tool (BLAST) (https://blast.ncbi.nlm.nih.gov/Blast.cgi).

Physicochemical data of compounds

Curvamine A (**8**): Yellow oil; $[\alpha]^{28}_{D}$ −10.6 (c 0.01 MeOH); UV (MeOH) λ_{max} (logε) 210 (3.9), 251 (3.9), 290 (3.8) nm; IR (KBr) ν_{max}: 3397, 2927, 1636, 1521, 1467, 1396, 1317, 1090 cm^{-1}; HRESIMS: *m/z*: 232.0946 $[M + Na]^+$ (calcd for $C_{11}H_{15}NO_3Na$, 232.0944); ^1H and ^{13}C NMR data assigned and listed in table S7.

Curvamine B (**9**): Brown needle; $[\alpha]^{28}_{D}$ +3.3 (c 0.06 MeOH); UV (MeOH) λ_{max} (logε) 210 (4.0), 252 (3.9), 291 (3.9) nm; IR (KBr) ν_{max}: 3376, 2930, 1721, 1639, 1470, 1395, 1208, 1065 cm^{-1}; HR-ESI-MS: *m/z*: 216.0998 $[M + Na]^+$ (calcd for $C_{11}H_{15}NO_2Na$, 216.0995); ^1H and ^{13}C NMR data assigned and listed in table S8.

Curvamine C (**10**): White solid. $[\alpha]^{25}_{D}$ −14.7 (c = 0.01, MeOH); UV (MeOH) λ_{max} (logε) 210 (3.9), 250 (3.9), 291 (3.8) nm; IR (KBr) ν_{max}: 3394, 2921, 1628, 1517, 1444, 1380, 1188, 1076 cm^{-1}; HR-ESI-MS: *m/z*: 246.1114 $[M + Na]^+$ (calcd for $C_{12}H_{17}NO_3Na$, 246.1106); ^1H and ^{13}C NMR data assigned and listed in table S9.

Curvamine D (**11**): White solid. $[\alpha]^{25}_{D}$ +5.8 (c = 0.02, MeOH); UV (MeOH) λ_{max} (logε) 210 (4.0), 251 (3.9), 291 (3.8) nm; IR (KBr) ν_{max}: 3406, 2940, 1626, 1516, 1445, 1319, 1228, 1188 cm^{-1}; HR-ESI-MS: *m/z*: 230.1157 $[M + Na]^+$ (calcd for $C_{12}H_{17}NO_2Na$, 230.1160); ^1H and ^{13}C NMR data assigned and listed in table S10.

Curvamine E (**12**): Yellow oil; $[\alpha]^{28}_{D}$ −38.0 (c 0.06 MeOH); UV (MeOH) λ_{max} (logε) 306 (4.0) nm; IR (KBr) ν_{max}: 3398, 1636, 1543, 1491, 1462, 1342, 1186, 1051 cm^{-1}; HR-ESI-MS: *m/z*: 218.0786 $[M + Na]^+$ (calcd for $C_{10}H_{13}NO_3Na$, 218.0788); ^1H and ^{13}C NMR data assigned and listed in table S11.

Curvamine F (**13**): Yellow oil; $[\alpha]^{28}_{D}$ +12.1 (c 0.02 MeOH); UV (MeOH) λ_{max} (logε) 302 (3.9) nm; IR (KBr) ν_{max}: 3423, 2979, 1649, 1542, 1491, 1336, 1187, 1052 cm^{-1}; HR-ESI-MS: *m/z*: 218.0790 $[M + Na]^+$ (calcd for $C_{10}H_{13}NO_3Na$, 218.0788); ^1H and ^{13}C NMR data assigned and listed in table S12.

Curvamine G (**14**): Brown solid; $[\alpha]^{20}_{D}$ +149.1 (c 0.06, MeOH); UV (MeOH) λ_{max} (logε) 229 (4.0), 298 (4.1) nm; IR (KBr) ν_{max}: 3441, 2970, 1538, 1450, 1376, 1205, 1051, 769 cm^{-1}; HRESIMS: *m/z*: 347.1751 $[M + Na]^+$ (calcd for $C_{20}H_{24}N_2O_2Na$, 347.1730); ^1H and ^{13}C NMR data assigned and listed in table S13.

Curvamine H (**15**): Yellow oil; $[\alpha]^{25}_{D}$ −210.0 (c 0.02 MeOH); UV (MeOH) λ_{max} (logε) 218 (4.0) nm; HR-ESI-MS: *m/z*: 361.2109 $[M + H]^+$ (calcd for $C_{20}H_{29}N_2O_4$ 361.2122). ^1H and ^{13}C NMR data assigned and listed in table S14.

Curvamine I (**16**): Yellow solid; $[\alpha]^{25}_{D}$ −26.7 (c 0.06 MeCN); UV (MeCN) λ_{max} (logε) 222 (3.9) nm; HR-ESI-MS: *m/z*: 357.1819 $[M + H]^+$ (calcd for $C_{20}H_{25}N_2O_4$ 357.1809); ^1H and ^{13}C NMR data assigned and listed in table S15.

Curvamine J (**17**): Yellow solid; $[\alpha]^{25}_{D}$ +32.2 (c 0.062 MeCN); UV (MeCN) λ_{max} (logε) 296 (3.0) nm; HR-ESI-MS: *m/z*: 359.1954 $[M + H]^+$ (calcd for $C_{20}H_{27}N_2O_4$ 359.1965); ^1H and ^{13}C NMR data assigned and listed in table S16.

Curvamine K (**18**): Yellow solid; $[\alpha]^{28}_{D}$ −131.7 (c 0.09 MeOH); UV(MeOH) λ_{max} 220 nm; IR (KBr) ν_{max} 3490, 2915, 1417, 1023, 749 cm^{-1}; HR-ESI-MS: *m/z*: 409.2110 $[M + Na]^+$ (calcd for $C_{22}H_{30}N_2O_4Na$, 409.2103); ^1H and ^{13}C NMR data assigned and listed in table S17.

Curvamine L (**19**): Yellow solid; $[\alpha]^{25}_{D}$ −240.8 (c 0.06 MeOH); UV (MeOH) λ_{max} (log ε) 290 (3.8) nm; IR (KBr) ν_{max}: 3425, 2974, 2935, 1651, 1499, 1446, 1387, 1301, 1060 cm^{-1}; HR-ESI-MS: *m/z*: 361.1524 $[M + Na]^+$ (calcd for $C_{20}H_{22}N_2O_3Na$, 361.1523); ^1H and ^{13}C NMR data assigned and listed in table S18.

Curvamine M (**20**): Yellow solid; $[\alpha]^{28}_{D}$ −17.9 (c 0.03 MeOH); UV (MeOH) λ_{max} (log ε) 294 (3.7) nm; IR (KBr) ν_{max}: 3411, 2977, 2938, 1689, 1488, 1398, 1236, 1020 cm^{-1}; HR-ESI-MS: *m/z*: 333.1566 $[M + Na]^+$ (calcd for $C_{19}H_{22}N_2O_2Na$, 333.1574); ^1H and ^{13}C NMR data assigned and listed in table S19.

Curvamine N (**21**): Yellow solid;$[\alpha]^{25}_{D}$ +50.4 (c 0.19 MeCN); UV (MeCN) λ_{max} (logε) 296 (3.7) nm; HR-ESI-MS: *m/z*: 341.1856 $[M + H]^+$ (calcd for $C_{20}H_{25}N_2O_3$ 341.1860); ^1H and ^{13}C NMR data assigned and listed in table S20.

Curvamine O (**22**): Yellow solid; $[\alpha]^{25}_{D}$ −250.9 (c 0.06 MeOH); UV(MeOH) λ_{max} (log ε) 297 (4.0) nm; IR (KBr) ν_{max}: 3419, 2974, 2935, 1651, 1478, 1448, 1385, 1299, 1045 cm^{-1}; HR-ESI-MS: *m/z*: 361.1524 $[M + Na]^+$ (calcd for $C_{20}H_{22}N_2O_3Na$, 361.1523); ^1H and ^{13}C NMR data assigned and listed in table S21.

Curvamine P (**23**): Yellow solid; $[\alpha]^{28}_{D}$ −111.7 (c 0.09 MeOH); UV(MeOH) λ_{max} (log ε) 237 (3.3), 294 (3.5) nm; IR (KBr) ν_{max}: 3501, 2930, 1411, 1023, 759 cm^{-1}; HR-ESI-MS: *m/z*: 377.1849 $[M + Na]^+$ (calcd for $C_{21}H_{26}N_2O_3Na$, 377.1841); ^1H and ^{13}C NMR data assigned and listed in table S22.

Curvamine Q (**24**): Yellow solid; $[\alpha]^{28}_{D}$ −13.4 (c 0.03 MeOH); UV (MeOH) λ_{max} (log *ε*) 294 (3.2) nm; IR (KBr) ν_{max}: 3431, 2968, 2927, 1689, 1420, 1379, 1263, 1035 cm^{-1}; HR-ESI-MS: *m/z*: 333.1566 $[M + Na]^+$ (calcd for $C_{19}H_{22}N_2O_2Na$, 333.1574); ^1H and ^{13}C NMR data assigned and listed in table S23.

Curvamine R (**25**): Yellow oil; $[\alpha]^{25}_{D}$ −40.0 (c 0.02 MeOH); UV (MeOH) λ_{max} (log *ε*) 210 (3.9), 230 (4.1), 290 (3.4) nm; HR-ESI-MS: *m/z*: 649.3780 $[M + H]^+$ (calcd for $C_{40}H_{49}N_4O_4$, 649.3748). ^1H and ^{13}C NMR data assigned and listed in table S24.

Curvamines S (**26**): Brown block; $[\alpha]^{25}_{D}$ −10.0 (c 0.02, MeOH); UV (MeOH) λ_{max} (log *ε*) 206 (3.5), 230 (3.6), 290 (3.2) nm; HR-ESI-MS: *m/z*: 649.3786 $[M + H]^+$ (calcd for $C_{40}H_{49}N_4O_4$, 649.3748). ^1H and ^{13}C NMR data assigned and listed in table S25.

Curvamine T (**27**): Brownish solid; $[\alpha]^{25}_{D}$ −305.7 (c 0.03, MeOH); UV (MeOH) λ_{max} (log *ε*) 230 (4.1), 296 (3.6) nm; IR (KBr) ν_{max}: 3420, 2926, 1706, 1578, 1447, 809 cm^{-1}; HR-ESI-MS: *m/z*: 647.3573 $[M + H]^+$ (calcd for $C_{40}H_{47}N_4O_4$, 647.3597). ^1H and ^{13}C NMR data assigned and listed in table S26.

Curvamines U (**28**): Brownish solid; $[\alpha]^{28}_{D}$ −298.6 (c 0.03 MeOH); UV (MeOH) λ_{max} (log *ε*) 230 (3.9), 296 (3.5) nm; IR (KBr) ν_{max}: 3398, 2969, 1689, 1415, 912 cm^{-1}; HR-ESI-MS: *m/z*: 647.3578 $[M + H]^+$ (calcd for $C_{40}H_{47}N_4O_4$, 647.3597). ^1H and ^{13}C NMR data assigned and listed in table S27.

Structural determination of new compounds

Curvamine A (**8**) was indicated to possess a molecular formula of $C_{11}H_{15}NO_3$ by the Na^+-liganded molecular ion at *m/z* 232.0946 in its HR-ESI-MS spectrum. The ^1H and ^{13}C NMR spectra of **8** were comparable to that of bipolamine B [22]. However, the two pyrrole proton doublets (J = 3.0 Hz) of bipolamine B at δ_H 5.77 and 5.87 were missing in the ^1H NMR spectrum of **8**, and replaced by two singlets at δ_H 9.71 and 6.22, the latter being broadened by its allylic coupling with H_3-10 (table S7). This observation, along with its 2D experiments (^1H-^1H COSY, HSQC, HMBC and NOESY), underscored that **8** was a 7-aldehyde derivative of bipolamine B. The (2*R*, 3*R*, 4*S*)-configuration of **8** was established by comparing its ECD spectrum with the ECD curves calculated for the possible stereoisomers (fig. S151).

Curvamine B (**9**) was analyzed to have a molecular formula of $C_{11}H_{15}NO_2$ by its HR-ESI-MS spectrum displaying the Na^+-liganded molecular ion at *m/z* 216.0998. The ^1H and ^{13}C NMR spectra of **9** revealed that it was a 4-deoxy derivative of **8**, in conjunction with its 2D NMR experiments (^1H-^1H COSY, HSQC, HMBC, and NOESY) (table S8). The conclusion was reinforced by the single-crystal X-ray diffraction analysis of **9** (CCDC No.994362) with monochromatic CuKα radiation (fig. S166), which clarified its (2*R*, 3*S*)-configuration as well.

Curvamine C (**10**) was found to have a molecular formula of $C_{12}H_{17}NO_3$ corresponding to its Na^+-liganded molecular ion at *m/z* 246.1114 in its HR-ESI-MS spectrum. The ^1H and ^{13}C NMR spectra of **10** were comparable to that of bipolamine B [22]. Similarly, the two pyrrole proton doublets (J = 3.0 Hz) of bipolamine B at δ_H 5.77 and 5.87 were missing in the ^1H NMR spectrum of **10**, and replaced by two singlets at δ_H 2.24 and 6.26 (table S9). This observation, along with its 2D experiments (^1H-^1H COSY, HSQC, HMBC, and NOESY), suggested that **10** was a 7-acetyl derivative of bipolamine B. The (2*R*, 3*R*, 4*S*)-configuration of **10** was established by the single-crystal X-ray diffraction analysis (CCDC No.1420072) with monochromatic CuKα radiation (fig. S166).

Curvamine D (**11**) was figured out to have a molecular formula of $C_{12}H_{17}NO_2$ from the Na^+-liganded molecular ion at *m/z* 230.1157 in its HR-ESI-MS spectrum. The ^1H and ^{13}C NMR data of **11** suggested that it was a 4-deoxy derivative of **10**. This is substantiated by its 2D NMR experiments (^1H-^1H COSY, HSQC, HMBC, and NOESY) (table S10) and by the single-crystal X-ray diffraction analysis (CCDC No.1420071) of **11** with monochromatic CuKα radiation, which demonstrated its (2*R*, 3*S*)-configurations (fig. S166).

Curvamine E (**12**) possessed a molecular formula $C_{10}H_{13}NO_3$ giving rise to the Na^+-liganded molecular ion at *m/z* 218.0786 in its HR-ESI-MS spectrum. The ^1H and ^{13}C NMR data of **12** were similar to those of bipolamine A [22]. However, in the ^1H NMR spectrum of **12**, the H-4 signal was displayed as a doublet (J = 9.5 Hz), and the two pyrrole protons resonated as doublets (J = 4.0 Hz) at δ_H 6.15 and 7.03, being moved downfield from those of bipolamine A (δ_H 5.73 and 5.83). The observation highlighted that **12** was most likely a 5-ketone derivation of bipolamine A. This hypothesis was confirmed by its 2D NMR experiments (^1H-^1H COSY, HSQC, HMBC, and NOESY) which led to the unambiguous assignment of all ^1H and ^{13}C NMR signals (table S11). The (2*R*, 3*R*, 4*S*)-configuration of **12** was assigned by comparing its experimental ECD curve to the theoretical ECD spectra calculated for all possible stereoisomers (fig. S152).

Curvamine F (**13**) possessed a molecular formula of $C_{10}H_{13}NO_3$ corresponding to the Na^+-liganded molecular ion at *m/z* 218.0790 in its HR-ESI-MS spectrum. The ^1H and ^{13}C NMR data of **13** (table S12) were assigned by the 2D NMR experiments including ^1H-^1H COSY, HSQC, HMBC, and ROESY, indicating that it was a 4-epimer of **12**. The (2*R*, 3*R*, 4*R*)-configuration of **13** was determined by comparing its ECD spectrum with its computational ECD curves (fig. S153) as done with **12**.

Curvamine G (**14**) was found to share the same molecular formula ($C_{20}H_{24}N_2O_2$) with curvulamine [59] by its Na^+-liganded molecular ion at *m/z* 347.1751 in its HR-ESI-MS spectrum. The ^1H and ^{13}C NMR data of **14** (table S13) were assigned unambiguously through a combination of ^1H-^1H COSY, HSQC, and HMBC experiments. But the H-14 doublet (J = 12.0 Hz) at δ_H 5.72 in the ^1H NMR spectrum of curvulamine moved downfield to δ_H 5.93 in that of

14 as a double doublet (J = 10.5, 9.0 Hz) coupling to the H-13 triplet at δ_H 3.23 (J = 9.0 Hz). Furthermore, the proton signals ascribable for 4- and 5-methine groups of curvulamine were missing in case of **14**. This observation, along with the two quaternary carbons at δ_C 127.8 and 123.9, indicated the presence of a 4,5-double bond of **14**. The formulated stereochemistry of **14** agreed with its NOESY spectrum (table S12) and the biogenetic relationship with curvulamine. The (2*R*, 3*R*, 12*S*, 13*S*)-configurations of **14** was assigned by comparing its recorded CD spectrum with all ECD curves calculated for the alkaloid (fig. S154).

Curvamine H (**15**) was determined to have a molecular formula of $C_{20}H_{28}N_2O_4$ by the H^+-liganded molecular ion at *m/z* 361.2109 in its HR-ESI-MS spectrum. This molecular formula underscored that it is a double nitrogenated alkaloid, too. However, the ^{1}H NMR spectrum of **15** displays only a single set of doublets at δ_H 5.63 (d, J = 3.5) and 5.71 (d, J = 3.5) ascribable for a 1,2,5-trisubstituted pyrrole nucleus (table S14). The ^{1}H and ^{13}C NMR data of **15** was comparable to those of bipolamine E [22]. The ^{1}H-^{1}H COSY correlation of H-8 (δ_H 4.14) with H-7 (δ_H 2.01 and 2.27), H-9 (δ_H 3.31), and H-10 (δ_H 1.15), along with HMBC correlations from H-8 to C-6 (δ_C 106.5) and H-7 to C-5 (δ_C 54.1) indicated the presence of a 1,2,4,5-tetrasubstituted tetrahydropyrrole motif. The HMBC correlation from H-12 (δ_H 4.25) to C-6 demonstrated another formation of ether linkage between C-6 and C-12, which explained the downfield shift of H-12 in tetrahydrofuran group. The *J*-coupling constant of H-8 and H-9 was 3.0 Hz, which indicated the opposite orientation of H-8 and H-9. The (2*R*, 3*R*, 4*S*, 5*S*, 6*R*, 8*S*, 9*R*, 12*S*, 13*S*, 14*R*)-configuration of **15** was assigned according to the comparison between calculated and experimental ECD spectra (fig. S155).

Curvamine I (**16**) was demonstrated to have a molecular $C_{20}H_{24}N_2O_4$ from its H^+-liganded molecular ion at *m/z* 357.1819 in its HR-ESI-MS spectrum, possessing an extra oxygen atom of bipolamine F. Comparison of their ^{1}H NMR data revealed that **16** contained an oxygenated methine (δ_H 4.09 and δ_C 86.6), whereas bipolamine F has a methylene (δ_H 1.83 and 2.78, δ_C 39.0) (table S15) [22]. This suggested the presence of a hydroxyl group at C-14 in **16**, which was supported by the ^{1}H-^{1}H COSY correlations of H-14 (δ_H 4.09) and H-15 (δ_H 5.05). The singlet of H-15 revealed an α-orientation for H-14. Subsequently, the absolute configuration (2*R*, 3*R*, 4*R*, 5*S*, 12*S*, 13*R*, 14*S*, 15*S*) of **16** was established by matching its recorded CD spectrum with those calculated for all options (fig. S156).

Curvamine J (**17**) was demonstrated to have a molecular formula of $C_{20}H_{26}N_2O_4$ by the H^+-liganded molecular ion at *m/z* 359.1954 in its HR-ESI-MS spectrum. A pair of 1,2,5-trisubstituted pyrrole nuclei are indicated by the four doublets (J = 3.3 or 3.5 Hz) at δ_H 5.94, 5.92 and 5.80 in the ^{1}H NMR spectrum of **17**. The ^{1}H and ^{13}C NMR data (table S16) of **17** were comparable to those of bipolamine G [22]. However, in the ^{1}H NMR spectrum of **17**, the H-15 signal was moved downfield to δ_H 4.69, suggesting that **17** was a 15-demethoxy derivative of bipolamine G. This assumption was confirmed by its 2D NMR spectra including the ^{1}H-^{1}H COSY, HSQC, HMBC, and NOESY experiments, which allowed the ascription of all ^{1}H and ^{13}C NMR signals. It was determined that the 14,15-vicinal diol adopts a *trans* configuration based on the coupling constants of H-14 and H-15. The absolute configuration was then established via the $Mo_2(OAc)_4$ method [60], which provided an induced circular dichroism (ICD) spectrum for the ligand–metal complex. The "net" ICD (i.e., subtracting the ECD spectrum of **17**) exhibited a positive Cotton effect at approximately 310 nm (fig. S157), thereby confirming the absolute configuration (14*R*, 15*S*). The remaining stereocenters are consistent with those of bipolamine G (see below).

Curvamine K (**18**) possessed a molecular formula of $C_{22}H_{30}N_2O_4$ by its HR-ESI-MS spectrum exhibiting the Na^+-liganded molecular ion at *m/z* 409.2110. Its ^{1}H and ^{13}C NMR data were similar to those of **17**, with the differences being the presences of two extra methoxy groups (δ_H 3.15 and 3.34) in **18**. The HSQC and HMBC spectra of **18** confirmed the hypothesis and corroborated that it was a methylated product of bipolamine G. **17** and **18** were shown to share (2*R*, 3*R*, 4*S*, 5*R*, 12*S*, 13*R*, 14*R*, 15*S*)-configuration by the close similarity in ^{1}H and ^{13}C NMR signals (table S17).

Curvamine L (**19**) possessed a molecular formula of $C_{20}H_{22}N_2O_3$ as demonstrated by the Na^+-liganded molecular ion at *m/z* 361.1524 in its HR-ESI-MS spectrum. The assignment of all ^{1}H and ^{13}C signals was accomplished by its

^{1}H-^{1}H COSY, HSQC, and HMBC spectra. Comparing between the ^{1}H NMR spectra of curvulamine [59] and **19** (table S18), a pyrrole methyl singlet δ_H 2.30 discerned in the former was substituted by an aldehyde singlet at δ_H 9.50. Correlating to the downfield movement of pyrrole proton doublets (J = 4.1 Hz) at δ_H 6.95 and 6.27, we inferred that **17** was 20-aldehyded derivative of curvulamine. Alkaloid **19** was shown to share (2*R*, 3*R*, 4*S*, 5*R*, 12*S*, 13*R*)-configuration with curvulamine by the close similarity in the ECD spectrum with curvulamine (fig. S158).

Curvamine M (**20**) was analyzed to have a molecular formula of $C_{19}H_{22}N_2O_2$ corresponding to the Na^+-liganded molecular ion at *m/z* 333.1566 in its HR-ESI-MS spectrum. The ^{1}H and ^{13}C NMR data of **20** (table S18) were close to those of curvulamine [59]. But one pyrrolic nucleus in **20** became 1,2-disubstituted as suggested by the three pyrrolic proton signals at δ_H 6.67, 6.17, and 6.15 in the ^{1}H NMR data of **20**, which displayed only one pyrrolic methyl singlet at δ_H 2.27. The observation highlighted that **20** could be a 19-demethylated analogue of curvulamine. The assignment was confirmed by the 2D NMR experiments, which collectively led to the exact assignment of all ^{1}H and ^{13}C NMR data of **20**. The absolute stereochemistry (2*R*, 3*R*, 4*S*, 5*R*, 12*S*, 13*R*) of **20** was clarified by its NOESY and ECD spectra, which are closely similar to those of curvulamine (fig. S159).

Curvamine N (**21**) was analyzed to have a molecular formula of $C_{20}H_{24}N_2O_3$ by its HR-ESI-MS spectrum displaying the H^+-liganded molecular ion at *m/z* 341.1856. The ^{1}H and ^{13}C NMR data of **21** were comparable to those of curvulamine [59]. However, the broadened pyrrole methyl singlet at δ_H 2.30 in the ^{1}H NMR spectrum of curvulamine was replaced by a pair of hydroxymethyl doublets (J = 13.2 Hz) at δ_H 4.65 and 4.54 (table S20). The observation indicated that **21** was a 10-hydroxylated derivative of curvulamine. This assumption was confirmed by its 2D NMR spectra including the ^{1}H-^{1}H COSY, HSQC, HMBC, and NOESY experiments, which allowed the ascription of all ^{1}H and ^{13}C NMR signals. Alkaloid **21** was shown to share (2*R*, 3*R*, 4*S*, 5*R*, 12*S*, 13*R*)-configuration with curvulamine by the close similarity between their ECD spectra (fig. S160).

Curvamine O (**22**) possessed a molecular formula of $C_{20}H_{22}N_2O_3$ as demonstrated by the Na^+-liganded molecular ion at *m/z* 361.1524 in its HR-ESI-MS spectrum. The assignment of all ^{1}H and ^{13}C signals was accomplished by its ^{1}H-^{1}H COSY, HSQC, HMBC, and NOESY spectra. Comparing the ^{1}H NMR spectra of curvulamine [59] and **22** (table S21), a pyrrole methyl singlet at δ_H 2.30 discerned in the former was substituted by an aldehyde singlet at δ_H 9.50. Correlating to the downfield movement of pyrrole proton doublets (J = 3.6 Hz) at δ_H 7.08 and 6.27, we inferred that **22** was 10-aldehyded derivative of curvulamine. **22** was shown to share (2*R*, 3*R*, 4*S*, 5*R*, 12*S*, 13*R*)-configuration with curvulamine by the close similarity in their ^{1}H and ^{13}C NMR data and optical rotations.

Curvamine P (**23**) was evidenced to have a molecular formula of $C_{21}H_{26}N_2O_3$ from the Na^+-liganded molecular ion at *m/z* 377.1849 in its HR-ESI-MS spectrum. The ^{1}H and ^{13}C NMR data of **23** were comparable to those of **21**. However, the hydroxymethyl signal in the ^{1}H NMR spectrum of **21** was replaced by a combination of a methoxy singlet at δ_H 3.23 and a hydroxymethyl doublets (J = 12.5 Hz) at δ_H 4.51 and 4.34 in that of **23** (table S22). The observation indicated that **23** was a 10-methoxylated derivative of **21**. This assumption was confirmed by its 2D NMR spectra including the ^{1}H-^{1}H COSY, HSQC, HMBC, and NOESY experiments, which allowed the ascription of all ^{1}H and ^{13}C NMR signals. The similarity in ECD spectrum (fig. S161) between **23** and curvulamine established their identical absolute configuration (2*R*, 3*R*, 4*S*, 5*R*, 12*S*, 13*R*).

Curvamine Q (**24**) was analyzed to have a molecular formula of $C_{19}H_{22}N_2O_2$ by its HR-ESI-MS spectrum displaying the Na^+-liganded molecular ion at *m/z* 333.1566. The ^{1}H and ^{13}C NMR data of **24** (table S23) were close to those of curvulamine [59]. But one pyrrolic nucleus in **24** became 1,2-disubstiuted as suggested by the three pyrrolic proton signals at δ_H 6.93 (dd, J = 2.5, 2.0 Hz), 6.10 (dd, J = 3.5, 2.0 Hz) and 6.07 (dd, J = 3.5, 2.5 Hz) in the ^{1}H NMR spectrum of **24**, which displayed only one pyrrolic methyl singlet at δ_H 2.24. The observation highlighted that **24** could be a 9-demethylated analog of curvulamine. The assignment was confirmed by the 2D NMR experiments, which collectively led to the exact assignment of all ^{1}H and ^{13}C NMR data of **24**. The absolute

stereochemistry of **24** was clarified by its NOESY spectrum and its single crystal X-ray diffraction (CuKα) (CCDC No.995223), establishing its (2*R*, 3*R*, 4*S*, 5*R*, 12*S*, 13*R*)-configuration (fig. S166).

Curvamines R (**25**) and S (**26**) were identical in the molecular formula ($C_{40}H_{48}N_4O_4$) corresponding to the H^+-liganded molecular ions at *m/z* 649.3780 and 649.3786, respectively, in their HR-ESI-MS spectra. This molecular formula is exactly twice that of curvulamine. The similarity in their ^1H and ^{13}C NMR data implies that they are isomers. Furthermore, they display similar sets of 1,2-disubstituted *cis*-double bond resonances [δ_H 5.74 (d, J = 11.8 Hz) and 6.43 (d, J = 11.8 Hz), and δ_C 120.0 and124.3 in **25** (table S24); as well as at δ_H 5.71 (d, J = 11.7 Hz) and 6.40 (d, J = 11.7 Hz), and at δ_C 120.0 and 124.2 in **26** (table S25)]. Obviously, each of the two sets is closely similar to those arising from the counterpart of curvamine Q (**24**) (table S23). Furthermore, one of the four pyrrole nuclei, which a 'curvulamine dimer' must possess, became 1,2,3,5-tetrasubstited. The observation allowed us to propose a dimerization pattern building on the covalent bond formation of C-8 with C15', in conjunction with the CH-CH_2 proton signals at δ_H 2.12 (d, J = 14.0 Hz), 2.46 (dd, J = 14.0, 4.5 Hz), and 4.20 (d, J = 4.5 Hz) for **25**; as well as at δ_H 2.10 (d, J = 11.5 Hz), and 4.25 (dd, J = 11.5, 1.5 Hz) for **26**. This assumption was confirmed by our 2D NMR experiments (^1H-^1H COSY, HSQC, HMBC, and NOESY). The absolute configuration of **25** and **26** was assigned by matching their recorded CD spectra with all calculated options (figs. S162 and S163). Co-characterization of **25** and **26** suggests that curvulamine is protonated to form carbocation (**A**). Though stabilized by its conjugation with a pyrrole nucleus, carbocation **A** remains active enough to form a covalent bond with the nucleophilic carbon of curvulamine (Scheme 1) [38], from *Si* or *Re* face to give **25** and **26**, respectively.

Curvamines T (**27**) and U (**28**) were evidenced to share a molecular formula of $C_{40}H_{46}N_4O_4$ from their HR-ESI-MS spectra, which exhibit the Na^+-liganded molecular ion at *m/z* 647.3573 and 647.3578, respectively. This molecular formula indicates a loss of two protons relative to **25** and **26** (vide supra), suggesting that they are oxidative dimers of curvulamine. As indicated by their ^1H and ^{13}C NMR data, each **27** or **28** molecule contains three rather than four pyrrole methyl groups, suggesting a pyrrole methyl-involved dimerization. With the rationalization in mind, we found that two sets of ^1H and ^{13}C NMR signals arising from the two 2-methyl-5-vinyl-pyrrole motifs of **27** are differentiated substantially (table S26), whereas counterparts of **28** are similar (table S27) and resemble those of curvamines N (**21**) and O (**22**) (tables S20 and S21). This observation, along with the most nucleophilic carbon noted for curvulamine, encouraged us to hypothesize the coupling of curvulamine with its oxidated (dehydrogenated) derivative. The formulated dimerization was corroborated by the 2D NMR experiments (^1H-^1H COSY, HSQC, HMBC, and NOESY), which allowed the ascription of all ^1H and ^{13}C NMR signals. Compounds **27** and **28** were stereochemically identical to curvulamine (figs. S164 and S165), respectively. In view of the singlet oxygen ($^1O_2$) upon the *ClHAS* inactivation, the production of **27** and **28** by the Δ*ClHAS* mutant is rationalized in Scheme 1.

Supplementary tables

Table S1. ROS and their production inside cells

Organelle	Primary ROS Generated	Key Enzymes/Processes Involved
Mitochondria	Superoxide ($O_2^{\bullet-}$), H_2O_2	Electron Transport Chain (Complex I & III), SOD2
Chloroplasts	Singlet Oxygen ($^1O_2$), Superoxide ($O_2^{\bullet-}$), H_2O_2	Photosystems I & II (Mehler reaction)
Peroxisomes	H_2O_2, Superoxide ($O_2^{\bullet-}$)	Fatty Acid *β*-Oxidation (AOX, XOX), Metabolic Oxidases
Endoplasmic Reticulum	H_2O_2, Superoxide ($O_2^{\bullet-}$)	Cytochrome P450 enzymes (EROX1), Protein Folding
Plasma Membrane	Superoxide ($O_2^{\bullet-}$)	NADPH Oxidases (NOX/DUOX)
Cytosol	Superoxide ($O_2^{\bullet-}$), H_2O_2	Xanthine Oxidase, Metalloenzymes, SOD1 (conversion)
Lysosomes	Hydroxyl Radical (•OH)	Fenton Reaction (H_2O_2 + Fe^{2+})
Nucleus	H_2O_2, •OH (minor)	DNA Damage, Specific Oxidases
Cell Wall (Plants)	Superoxide ($O_2^{\bullet-}$), H_2O_2	NADPH Oxidases, Peroxidases

Table S2. Primers used in the study for gene deletion and overexpression.

Primer name	Sequence (5' to 3')	Purpose
U-cuaF-F	CGGGGATCCTCTAGAGTCGACTGCGTTGATCTGTTTGACGTC	*cuaF* deletion
U-cuaF-R	CTCCTTCAATATCATCTTCTGGGATGCGTGGTTCGAGGCT	
D-cuaF-F	ATCCTTCTTTCTAGAGGATCCCGGGTTTCAATGGTATCTA	
D-cuaF-R	CTTGCATGCCTGCAGGTCGACGAGCGGTCTCAAGGACGATC	
cuaF-A-F	TGCGTTGATCTGTTTGACGTC	
A-R	AGCGGATTCCTCAGTCTC	
B-F	CTCCTTCAATATCATCTTCTGATGCGCTGCAACTCCAC	
cuaF-B-R	GAGCGGTCTCAAGGACGATC	
U-*Cl*HAS-F	GCTTTCTGGACGACCGCTCTG	*Cl*HAS deletion
U- *Cl*HAS -R	CTCCTTCAATATCATCTTCTGGCCCTGAATCTTCTTCATG	
D- *Cl*HAS -F	ATCCTTCTTTCTAGAGGATCCTGCCTGCATGCGAAGAGCC	
D- *Cl*HAS -R	CCTGAGGCTGTCCGAGTTAGT	
*Cl*HAS -A-F	CATGAAGAAGATTCAGGGCCAGAAGATGATATTGAAGGAG	
*Cl*HAS -B-R	GGCTCTTCGCATGCAGGCAGGATCCTCTAGAAAGAAGGAT	
U-OE-A7370-F	AGCTCGGTACCCGGGGATCCTACAGGCGGGACAAGTAAG	*A7370* overexpression
U-OE-A7370-R	TCCTTCAATATCATCTTCTGCAGATGTGAAACCATGTCTAA	
hph2-AmyB-F	TCCTTCTTTCTAGAGGATCCCGACTCCAATCTTCAAGAG	
AmyB-R	GGTACCGAGCTCGAATTC	
D-OE-A7370-F	CCGAATTCGAGCTCGGTACCCCTTAGCATCACCCACC	
D-OE-A7370-R	ACCATGATTACGCCAAGCTTACCCGAAAGTCCTTTCG	
OE-A7370-A-F	AGGCGGGACAAGTAAGT	
OE-A7370-B-R	ACCCGAAAGTCCTTTCG	
U-OE-CuaF-F	CGGGGATCCTCTAGAGTCGACTGACAATTTCAACGCCTTTG	*cuaF* overexpression
U-OE-CuaF-R	CTCCTTCAATATCATCTTCTGCAAGATGGATGCCTCTGGA	
D-OE-CuaF-F	GAATTCGAGCTCGGTACCATGCCACCACCAACCGGCAT	
D-OE-CuaF-R	CTTGCATGCCTGCAGGTCGACACTGATTCAGCGTCTCTGC	
OE-CuaF-A-F	TGACAATTTCAACGCCTTTG	
OE-CuaF-B-R	ACTGATTCAGCGTCTCTGC	
hph-1F	CAGAAGATGATATTGAAGGAG	hph1 construction
hph-1R	GACCATGATTACGCCAAGCTTAGCGGATTCCTCAGTCTCGTA	
hph-2F	CGAGCTCGGTACCCGGGGATCCAGACCTGCCTGAAACCGAAC	hph2 construction
hph-2R	GGATCCTCTAGAAAGAAGGAT	
cuaF-Diag-F	ATCTTACACCCCAAGCGT	verification of Δ*cuaF*
cuaF-Diag-R	GCTTCTCACGGGTTTCAG	
*Cl*HAS -Diag-F	AGAACTGTTGATAGGCGACT	verification of Δ*Cl*HAS
*Cl*HAS -Diag-R	TATGCGTTGCGTCTGGAG	
OE-A7370-Diag-F	GTAAAACCCCGGAGTCAAC	verification of OE::*A7370*
OE-A7370-Diag-R	TACAACACCGTGTCGGGA	
OE-cuaF-Diag-F	TCAGTAACGGCGAAGATGGC	verification of OE::*cuaF*
OE-cuaF-Diag-R	TCAAGGTATTAATCAAGTGC	

Table S3. Primers used in this study for RT-qPCR analysis.

Primer name	Sequence (5' to 3')
actA-F	GAAGTCCTACGAACTGCCTGATG
actA-R	AAGAACGCTGGGCTGGAA
A7370-q-F	CAACGCTTCGCCGAGCA
A7370-q-R	CGGGTGATGTCTGGTCGT
A5838-q-F	TCCATGAACGCTGGCCAC
A5838-q-R	GGCGTTGCTCTTCTTCCG
A2892-q-F	CGCCGCACAAGCAGACAAA
A2892-q-R	GGCGGTGTAGGCCTCCA
A6358-q-F	CCCAAGATTCGGTGCACTT
A6358-q-R	GGTGTTGCTGCGAGTGGC
A6901-q-F	GGAGCTCGAGACCAAGCTC
A6901-q-R	GCGGCTGGACGTGATACTC
CuaA-q-F	CAACGTGTCGGCCTACC
CuaA-q-R	TTGCTGCGGGTCCGCAA
CuaB-q-F	CGGCATGAGCCACACTG
CuaB-q-R	GCCCATGAGCTCTGCTAG
CuaC-q-F	GGGGGTCAAATCGTTCCC
CuaC-q-R	GGTCCATCCTCGGGTCC
CuaD-q-F	CCACTGGATCAGCTTGGC
CuaD-q-R	CTCGAGGCACCATCTGC
CuaE-q-F	AAAAGTTCCCATGGCTCGC
CuaE-q-R	CTGCCGACCTCAAAGCAG
CuaF-q-F	CGCCCAAGATAGCGAGCA
CuaF-q-R	TCGTCTGGGGCAAAAGGC

Table S4. Primers used in this study for RNAi-mediated silencing.

Primer name	Sequence (5' to 3')
7370-pS-R	GGCTGCAGGAATTCAAGCTTTGTGGATCCCACGGCTCGCC
7370-pS-F	CCCAAGCATCGATAAGCTTCCATTGCTCCGAAGAAGATG
ps-hph-F	GCGTAATACGACTCACTATAGGCAGAAGATGATATTGAAGGAG
ps-hph-R	CTCGAGAACCCAGGGCTGGATCCTCTAGAAAGAAGGAT
ps-dual-F	CCTATAGTGAGTCGTATTACGC
ps-dual-R	AGCCCTGGGTTCTCGAG

Table S5. Primers used in this study for Chip-seq assay.

**Primer name**	**Sequence (5' to 3')**
7370-Down-F	AATCCTTCTTTCTAGAGGATCCCTATCGTTGTTTTCTCTTTGGAAG
7370-Down-R	GACCATGATTACGCCAAGCTTTTCCGCATTACCCGTCC
puc19-hph-F	CGAGCTCGGTACCCGGGGATCCCAAGGTAAGTGAACGACCC
hph2-R	GGATCCTCTAGAAAGAAGGAT
hph2-7370-B-F	GGTAAGTGAACGACCCGG
hph2-7370-B-R	CATTACCCGTCCGCTTCG
pcDNA3.1-F	CTCGAGTCTAGAGGGCCC
pcDNA3.1-R	GAATTCCACCACACTGGACTA
pcDNA3.1-7370-F	TAGTCCAGTGTGGTGGAATTCTCATCGATCGACATCGGTG
pcDNA-3.1-7370-R	GGGCCCTCTAGACTCGAGCCAGCTACTTGCATCGTACT
pcDNA3.1-A7370-line-F	GTTTAAACCCGCTGATCAGC
pcDNA3.1-A7370-line-R	TCACTTGTCATCGTCATCCT
3Flag-trpC-F	CAAGGATGACGATGACAAGTGAATCCACTTAACGTTACTGAAATCAT
3Flag-trpC-R	GCTCCTTCAATATCATCTTCTGGGATCCTCTAGAAAGAAGGATTAC
pcDNA3.1-7370-TrpC-hph-F	AATCCTTCTTTCTAGAGGATCCCAGAAGATGATATTGAAGGAGCA
pcDNA3.1-7370-TrpC-hph-R	AGGCTGATCAGCGGGTTTAAACTTTGCCCTCGGACGAGT
pcDNA-amyB-line-F	CAGAAGATGATATTGAAGGAGCAT
pcDNA3.1-A7370-line-R	TCACTTGTCATCGTCATCCT
PamyB-F2	GATCATGACATCGACTACAAGGATGACGATGACAAGTGAATGTCCCT TGTCGATGCG
PamyB-R2	CAGCCAAGCCCAAAAATGCTCCTTCAATATCATCTTCTGTGGTGGGG TAACCAAGGT
7370-flag-TrpC-hph1-A-F	ATCGATCGACATCGGTGC
7370-flag-TrpC-hph1-A-R	TTGCCCTCGGACGAGTG
A7370-3×Flag tag-Diag-F	ACAGAGCTGCAGCTCAC
A7370-3×Flag tag-Diag-R	GACAAACGCACAAGTTATCGT

Table S6. Oligonucleotides utilized for gel shift assay.

Primer name	Sequence (5' to 3')
probe-1-F (5'-Fam)	ATTACTCGCTTGGCAGGCC
probe-1-R	GGTGGCATGGATGCGTGG
probe-2-F (5'-Fam)	GTGGTGCATGAGGCTTGC
probe-2-R	TAGAGCCAAGTCGGCCTG
probe-3-F (5'-Fam)	TTACACAGCAGCTTGAGCAGG
probe-3-R	TCACGGCATGCAAGCCTC
probe-4-F (5'-Fam)	GTGGCGTGCTGTAAGGC
probe-4-R	AGTCCCCTCATCTTACCTGC
probe-5-F (5'-Fam)	ACCCCACCATCACAGCC
probe-5-R	GTGTCGCTACCATTCCCTTT
probe-6-F (5'-Fam)	TGCAAGTGGACGACGCT
probe-6-R	GGCCCATGTTAACGTACGA
probe-6-3F (5'-Fam)	ACGTCTCCCCATCCAACG
probe-6-60-R	GAGTGGTGGGGGGTCCTG
probe-6-70-R	CTAGTGTGGGGAGTGGTG
probe-6-80-R	TGAAATGGCACTAGTGTGGG
probe-6-91-R	GCATGTACATGTGAAATGGC
probe-6-30-50-F (5'-Fam)	ATTGTGCGGAACCAGGACCCC
probe-6-2-R	TTCCGCACAATGTCAGCC
hit-30-F (5'-Fam)	ATCCAACGCAGACGGCTGACATTGTGCGGA
hit-30-R	TCCGCACAATGTCAGCCGTCTGCGTTGGAT
hit-30-mut-F (5'-Fam)	ATCCAACGCAGCCGGCTGATATTGTGCGGA
hit-30-R	TCCGCACAATATCAGCCGGCTGCGTTGGAT

Table S7. ^{1}H and ^{13}C NMR data of curvamine A (**8**).

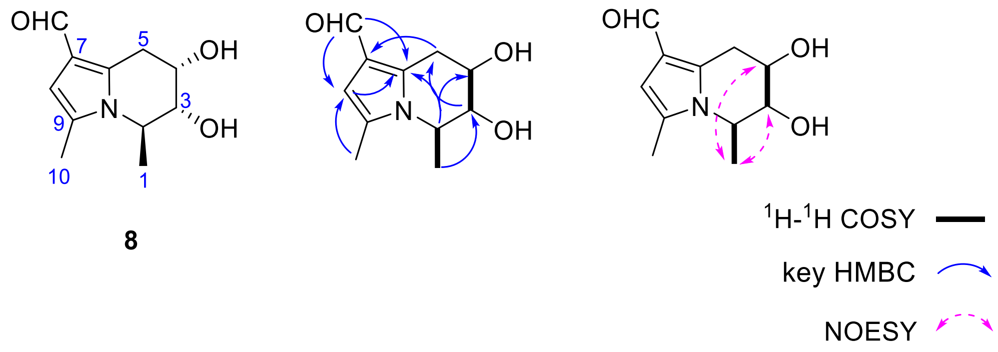

Position	δ_H (mult, *J* in Hz)	δ_C	HMBC	NOESY
1	1.37 (d, *J* = 6.5)	20.1	C3	H3, H4
2	4.32 (qd, *J* = 6.5, 2.0)	55.8	C4, C6	H1
3	4.05 (t, *J* = 2.0)	73.3	C5	H1, H4
4	4.26 (ddd, *J* = 10.5, 6.5, 2.0)	63.9	C2	H5
5a	3.43 (dd, *J* = 12.0, 6.5)	27.4	C7	H4
5b	2.98 (dd, *J* = 12.0, 10.5)			
6		137.2		
7		120.9		
8	6.22 (br s)	108.2	C6	
9		129.5		
10	2.21 (br s)	11.7	C8	
11	9.71 (s)	184.5	C6, C8	

Table S8. ^{1}H and ^{13}C NMR data of curvamine B (**9**).

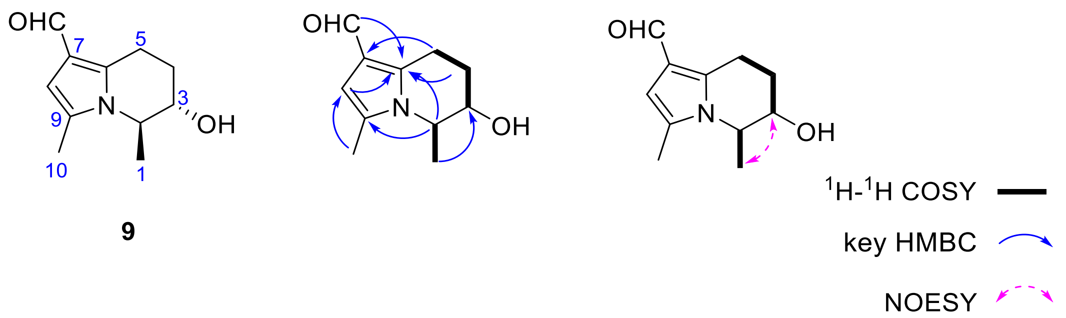

Position	δ_H (mult, *J* in Hz)	δ_C	HMBC	NOESY
1	1.30 (d, *J* = 6.8)	20.5	C3	H2, H3
2	4.19 (qd, *J* = 6.8, 2.0)	55.0	C6, C9	H3
3	4.14 (dt, *J* = 4.0, 2.0)	68.6		
4a	2.03 (ddd, *J* = 11.2, 7.2, 2.0)	21.9	C2, C6	H1, H5
4b	1.97 (ddd, *J* = 11.2, 4.0, 2.0)			
5a	3.16 (ddd, *J* = 17.6, 7.2, 2.0)	17.9	C3, C7	
5b	3.13 (dd, *J* = 17.6, 7.2)			
6		138.1		
7		120.9		
8	6.20 (br d, *J* = 0.8)	107.4	C6	
9		129.4		
10	2.20 (br d, *J* = 0.8)	11.6	C8	
11	9.72 (s)	184.4	C6	

Table S9. ^{1}H and ^{13}C NMR data of curvamine C (**10**).

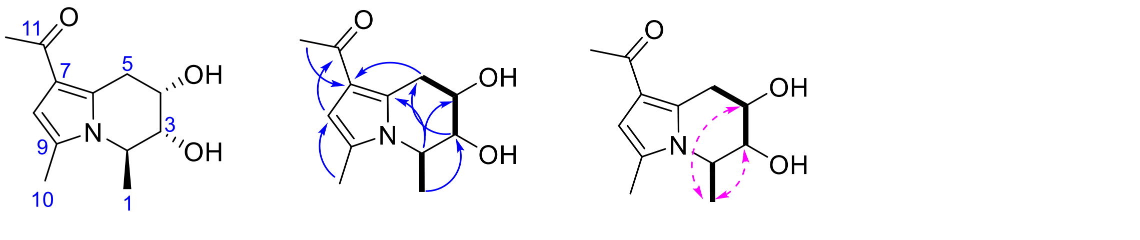

10

Position	δ_H (mult, *J*, Hz)	δ_C	HMBC	NOESY
1	1.34 (d, *J* = 7.2)	20.1	C3	H3, H4
2	4.30 (qd, *J* = 7.2, 2.4)	55.6	C4, C6	
3	4.02 (t, *J* = 2.4)	73.0	C5	H4
4	4.19 (ddd, *J* = 10.2, 6.6, 2.4)	64.1		H5
5a	3.49 (dd, *J* = 17.4, 6.6)	29.5	C7	H4
5b	2.87 (dd, *J* = 17.4, 10.2)			
6		134.4		
7		119.5		
8	6.26 (br s)	110.2	C10, C11	H10
9		127.4		
10	2.21 (br s)	11.8	C8	
11		193.9		
12	2.24 (s)	28.1	C7	

Table S10. ^{1}H and ^{13}C NMR data of curvamine D (**11**).

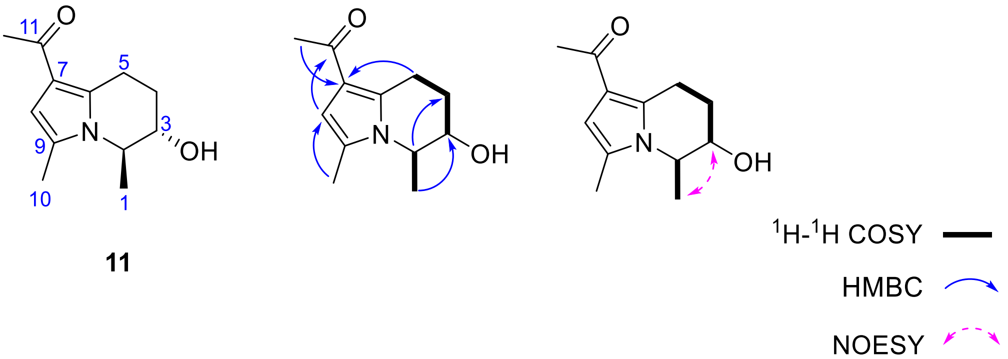

11

Position	δ_H (mult, *J* in Hz)	δ_C	HMBC	NOESY
1	1.28 (d, *J* = 6.8)	20.6	C3	H3
2	4.17 (qd, *J* = 6.8, 2.1)	54.9	C3, C4	H1
3	4.10 (m)	68.7		H1, H4
4a	1.95 (m)	22.2		
4b	1.91 (m)			
5a	3.17 (ddd, *J* =18.6, 6.6, 2.2)	20.0	C7	H4
5b	2.99 (dd, *J* =18.6, 6.6)			
6		134.9		
7		119.6		
8	6.23 (br s)	109.3	C10, C11	H10
9		127.1		
10	2.19 (br s)	11.6	C8	
11		193.0		
12	2.22 (s)	28.4	C7	

Table S11. ^{1}H and ^{13}C NMR data of curvamine E (**12**) (CDCl$_3$).

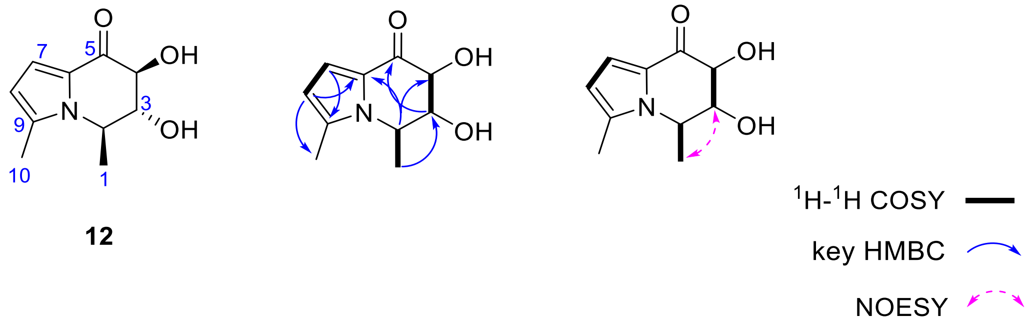

12

Position	δ_H (mult, J in Hz)	δ_C	HMBC	NOESY
1	1.69 (d, J = 7.5)	19.6	C3	H3
2	4.24 (dq, J = 8.0, 7.5)	55.3	C4	
3	3.83 (dd, J = 9.5, 8.0)	76.2	C5	H1
4	4.22 (d, J = 9.5)	75.1	C2	
5		183.9		
6		127.8		
7	7.03 (d, J = 4.0)	116.0	C5	
8	6.15 (d, J = 4.0)	113.6	C6, C10	H10
9		138.1		
10	2.39 (br s)	14.8	C8	

Table S12. ^{1}H and ^{13}C NMR data of curvamine F (**13**) ($CDCl_3$).

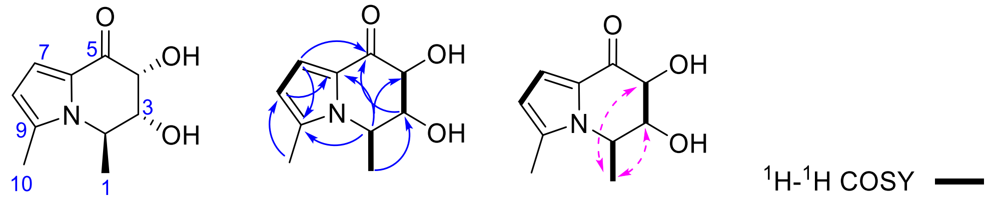

13

Position	δ_H (mult, J in Hz)	δ_C	HMBC	ROESY
1	1.50 (d, J = 7.0)	18.6	C3	H3, H4
2	4.57 (qd, J = 7.0, 2.0)	53.0	C4, C6, C9	
3	4.42 (dd, J = 3.0, 2.0)	73.4	C5	H1
4	4.56 (d, J = 3.0)	70.4	C2	
5		183.9		
6		126.3		
7	7.07 (d, J = 4.0)	116.1	C5, C9	
8	6.13 (d, J = 4.0)	112.4	C6	H10
9		136.8		
10	2.33 (br s)	12.0	C8	

Table S13. ^{1}H and ^{13}C NMR data of curvamine G (**14**).

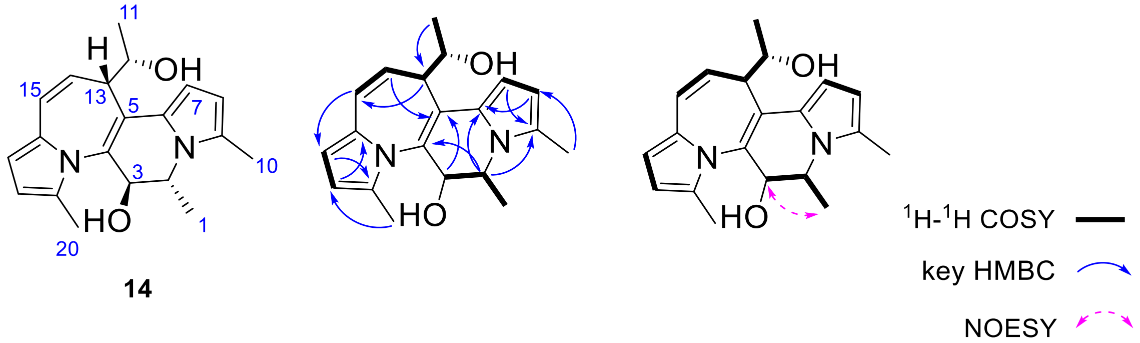

Position	δ_H (mult, *J* in Hz)	δ_C	HMBC	NOESY
1	1.35 (d, *J* = 7.0)	19.0	C3	H2, H3
2	4.44 (qd, *J* = 7.0, 2.0)	55.4	C4, C6	
3	4.60 (d, *J* = 2.0)	72.2	C5	H1
4		123.9		
5		127.8		
6		128.2		
7	6.30 (d, *J* = 3.5)	107.6	C9	
8	5.87 (d, *J* = 3.5)	108.5	C6	
9		131.1		
10	2.29 (s)	11.5	C8	
11	1.03 (d, *J* = 6.0)	12.5	C13	H12
12	3.48 (m)	66.7		
13	3.23 (t, *J* = 9.0)	48.1	C15	
14	5.93 (dd, *J* = 10.5, 9.0)	129.3	C5	H13, H15
15	6.46 (d, *J* = 10.5)	123.2	C17	H14
16		134.7		
17	6.02 (d, *J* = 3.5)	108.4	C19	
18	5.93 (d, *J* = 3.5)	109.7	C16	H20
19		130.9		
20	2.40 (s)	15.1	C18	

Table S14. ^{1}H and ^{13}C NMR data of curvamine H (**15**).

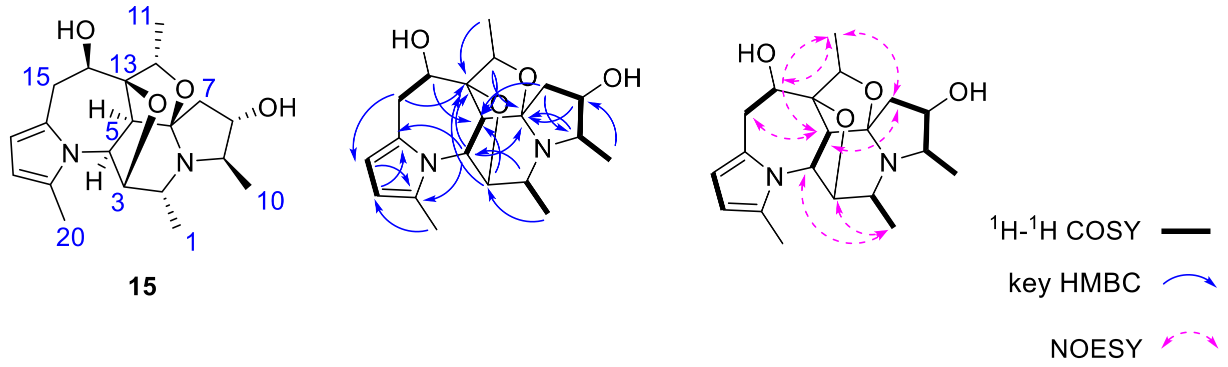

15

Position	δ_H (mult, J in Hz)	δ_C	HMBC	NOESY
1	1.09 (d, J = 6.5)	16.9	C3	H3, H4, H9
2	3.21 (q, J = 6.5)	54.6	C4	
3	4.04 (br s)	87.2	C5, C13	H1
4	4.64 (br s)	56.7	C6, C13, C16	H1
5	2.57 (br s)	54.1		H7, H15, H14
6		106.5		
7a	2.27 (dd, J = 12.0, 7.0)	49.4	C5, C9	
7b	2.01 (dd, J = 12.0, 7.0)			
8	4.14 (td, J = 7.0, 3.0)	77.8	C6	
9	3.31 (qd, J = 7.0, 3.0)	61.3		
10	1.15 (d, J = 7.0)	17.9	C8	
11	1.22 (d, J = 6.5)	19.8	C13	H5, H7a, H14
12	4.25 (q, J = 6.5)	80.3	C5, C6	
13		98.9		
14	3.80 (dd, J = 10.5, 4.4)	70.8	C5	H5, H11
15a	3.06 (dd, J = 14.0, 10.5)	34.2	C13, C17	H5
15b	2.87 (dd, J = 14.0, 4.4)			
16		129.7		
17	5.71 (d, J = 3.5)	108.7	C19	
18	5.63 (d, J = 3.5)	106.3	C16	
19		128.0		
20	2.21 (s)	13.5	C18	

Table S15. ^{1}H and ^{13}C NMR data of curvamine I (**16**).

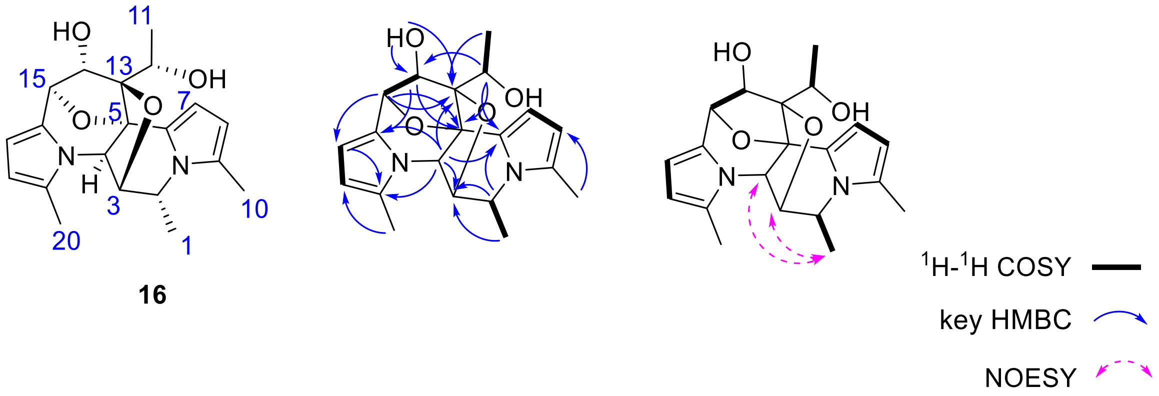

Position	δ_H (mult, J in Hz)	δ_C	HMBC	NOESY
1	1.63 (d, J = 7.0)	19.8	C2, C3	H3, H4
2	4.41 (q, J = 7.0)	57.9	C3, C6	
3	4.56 (s)	84.3	C13	H1
4	4.65 (s)	60.8	C3, C6, C13	H1
5		88.5		
6		131.1		
7	5.87 (d, J = 3.4)	102.3		
8	5.81 (d, J = 3.4)	107.7	C7	
9		128.1		
10	2.28 (s)	11.8	C8, C9	
11	1.33 (d, J = 6.6)	18.9	C13	
12	3.01 (m)	72.4	C14	
13		94.1		
14	4.09 (d, J = 9.4)	86.6	C16, C5	H11, H15
15	5.05 (s)	84.6	C5, C13, C17	H14
16		129.3		
17	6.05 (d, J = 2.9)	101.2	C19	
18	5.87 (d, J = 2.9)	108.7		
19		130.1		
20	2.31 (s)	13.3	C18, C19	
12-OH	3.54 (d, J = 5.1)		C11, C12	
14-OH	5.42 (d, J = 9.4)		C13, C14	

Table S16. ^{1}H and ^{13}C NMR data of curvamine J (**17**).

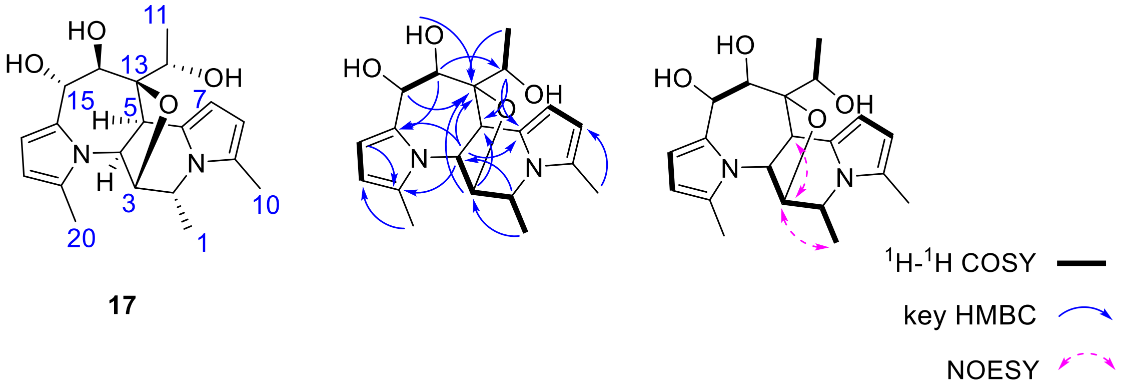

Position	δ_H (mult, J in Hz)	δ_C	HMBC	NOESY
1	1.56 (d, J = 6.8)	19.5	C2, C3	H3, H5
2	4.32 (m)	57.6	C4	
3	4.29 (overlap)	86.8	C5	H5
4	5.16 (s)	42.2	C6, C13, C19	
5	4.89 (s)	60.5	C4, C6	H3
6		131.6		
7	5.92 (d, J = 3.5)	111.9	C8	
8	5.80 (d, J = 3.5)	108.2		
9		128.4		
10	2.29 (s)	13.6	C8, C9	
11	1.07 (d, J = 6.5)	18.7	C12, C13	
12	2.51 (m)	70.4		
13		91.7		
14	4.29 (overlap)	86.8	C16	
15	4.69 (d, J = 3.3)	71.7	C13	
16		130.8		
17	5.94 (d, J = 3.3)	105.1	C19	
18	5.80 (d, J = 3.3)	108.1		
19		128.3		
20	2.26 (s)	12.8	C18, C19	
14-OH	2.94 (d, J = 5.2)		C13, C14	

Table S17. ^{1}H and ^{13}C NMR data of curvamine K (**18**).

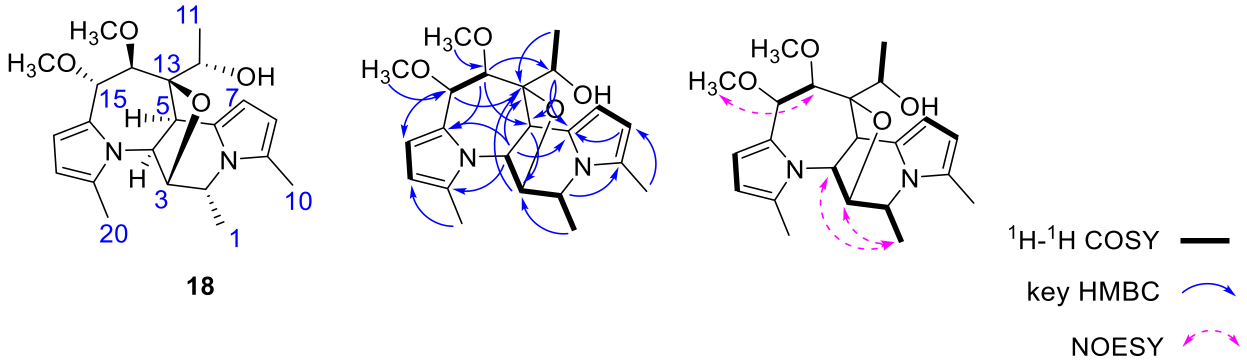

Position	δ_H (mult, J in Hz)	δ_C	HMBC	NOESY
1	1.51 (d, $J = 6.5$)	19.7	C3	H3, H4
2	4.20 (q, $J = 6.5$)	57.8	C9	
3	4.12 (br s)	87.0		H1
4	4.79 (br s)	60.8	C13, C16, C19	H1
5	4.76 (br s)	42.8	C3	
6		132.9		
7	5.86 (d, $J = 3.0$)	104.7		
8	5.76 (d, $J = 3.0$)	108.1		
9		129.0		
10	2.25 (br s)	12.8	C8	H1
11	1.03 (d, $J = 6.5$)	18.2	C13	H12
12	2.43 (q, $J = 6.5$)	70.0		H11
13		91.3		
14	4.33 (d, $J = 2.5$)	75.7	C16	H15
15	3.99 (d, $J = 2.5$)	80.3	C13, C17	H14
16		131.3		
17	6.04 (d, $J = 3.0$)	113.5		H18
18	5.81 (d, $J = 3.0$)	107.7		
19		133.5		
20	2.28 (br s)	13.6	C18	
14-OCH_3	3.15 (s)	55.5	C14	H15
15-OCH_3	3.34 (s)	56.4	C15	H14, H15

Table S18. ^{1}H and ^{13}C NMR data of curvamine L (**19**) ($CDCl_3$).

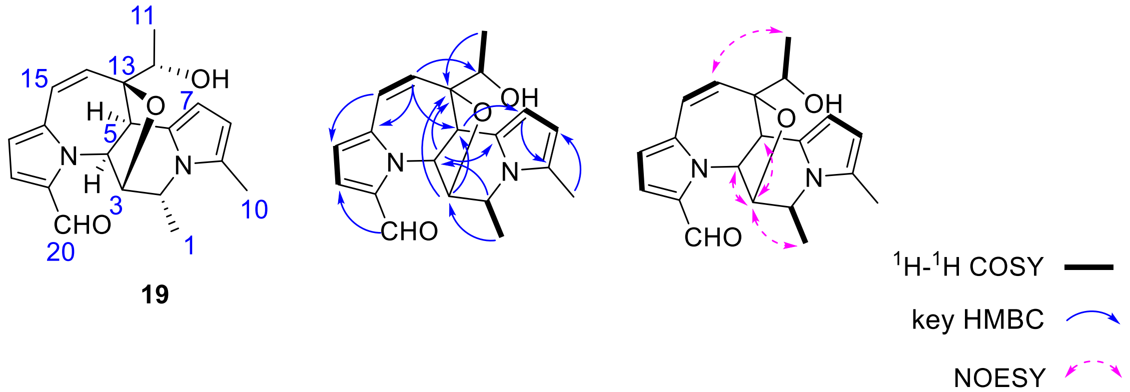

19

Position	δ_H (mult, *J* in Hz)	δ_C	HMBC	NOESY
1	1.65 (d, *J* = 7.0)	19.1	C2, C3	H3, H4, H5
2	4.14 (q, *J* = 7.0)	58.2	C3, C4	
3	4.58 (br s)	100.0	C13	H4, H5
4	6.33 (br s)	61.8	C4, C13, C16	H20
5	3.88 (br s)	44.2	C3	
6		128.8		
7	5.86 (d, *J* = 3.1)	103.6	C6, C9	
8	5.89 (d, *J* = 3.1)	108.0		
9		129.0		
10	2.28 (s)	13.1	C8, C9	
11	1.20 (d, *J* = 6.8)	17.4	C12, C13	H14
12	2.62 (d, *J* = 6.8)	69.9		
13		88.9		
14	6.20 (d, *J* = 11.8)	130.3	C5, C16	
15	6.50 (d, *J* = 11.8)	123.3	C13, C16, C17	
16		140.0		
17	6.27 (d, *J* = 4.1)	115.3	C16, C19	
18	6.95 (d, *J* = 4.1)	126.8	C17, C19	H20
19		133.7		
20	9.50 (s)	179.4		

Table S19. ^{1}H and ^{13}C NMR data of curvamine M (**20**) ($CDCl_3$).

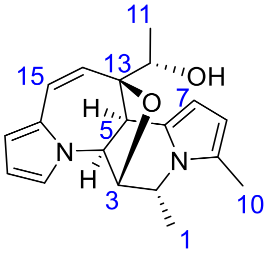

20

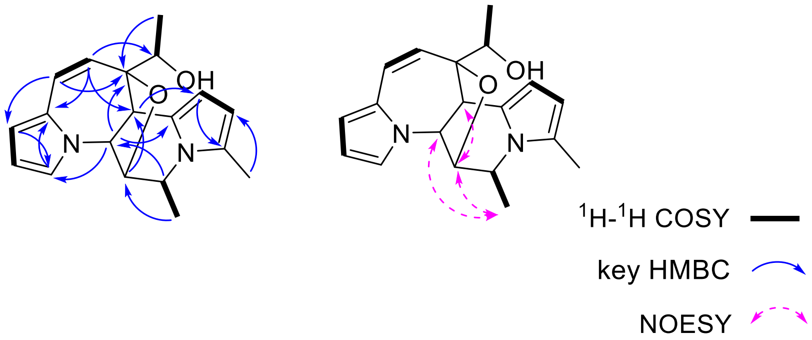

Position	δ_H (mult, J in Hz)	δ_C	HMBC	NOESY
1	1.46 (d, J = 6.6)	19.0	C2, C3	H3, H4
2	4.17 (q, J = 6.6)	57.2	C3, C4	
3	4.54 (br s)	89.1	C4, C5, C13	H5
4	4.91 (br s)	64.3	C6, C13, C19	H19, H8
5	3.97 (br s)	44.7	C3, C7	
6		129.4		
7	5.89 (d, J = 3.4)	103.8	C9	
8	5.91 (d, J = 3.4)	107.8	C9	
9		128.7		
10	2.27 (s)	12.8	C8, C9	H8
11	1.21 (d, J = 6.4)	17.4	C12, C13	
12	2.68 (m)	70.3		
13		89.3		
14	5.76 (d, J = 11.8)	121.6	C5, C12, C16	H15
15	6.46 (d, J = 11.8)	123.9	C13, C16, C17	H14
16		130.6		
17	6.17 (m)	114.5	C15, C16, C19	H15
18	6.15 (m)	109.5	C16	H19
19	6.67 (m)	125.1	C18, C17, C16	

Table S20. ^{1}H and ^{13}C NMR data of curvamine N (**21**).

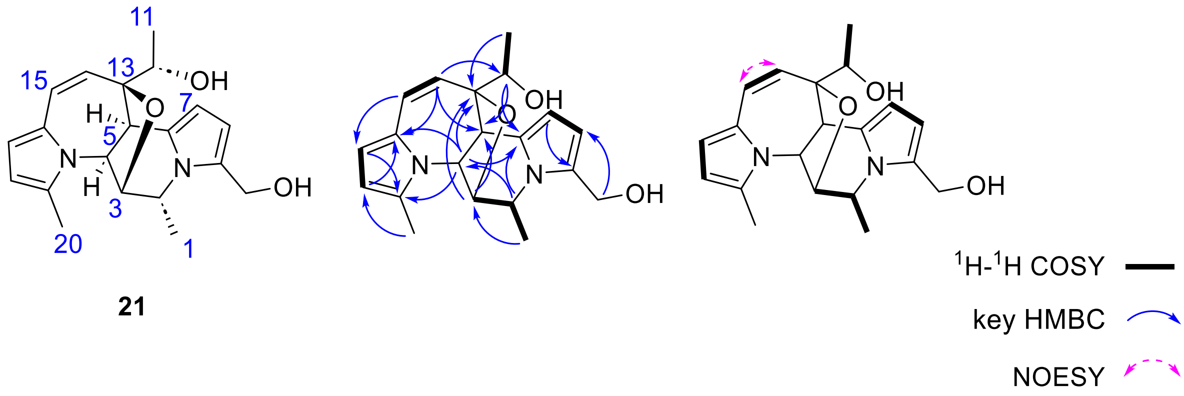

21

Position	δ_H (mult, J in Hz)	δ_C	HMBC	NOESY
1	1.61 (d, J = 6.8)	19.9	C3	
2	4.41 (q, J = 6.8)	58.9	C4, C6	
3	4.47 (br s)	89.7	C5, C13	
4	4.98 (br s)	61.2	C6, C13, C16	
5	3.84 (s)	46.2	C3, C4, C6	
6		132.2		
7	5.96 (d, J = 3.3)	105.3	C9	
8	6.00 (d, J = 3.3)	109.8	C10	
9		133.2		
10a	4.54 (d, J = 13.2)	57.1	C8, C9	
10b	4.65 (d, J = 13.2)			
11	1.08 (d, J = 6.4)	18.3	C13	
12	2.59 (q, J = 6.4)	70.1		
13		90.1		
14	5.70 (d, J = 11.9)	121.7	C5, C12, C16	H15
15	6.32 (d, J = 11.9)	124.0	C17	H14
16		131.3		
17	6.00 (d, J = 3.3)	113.4	C19	
18	5.86 (d, J = 3.3)	108.8	C16	
19		133.5		
20	2.32 (s)	13.7	C19	

Table S21. ^{1}H and ^{13}C NMR data of curvamine O (**22**).

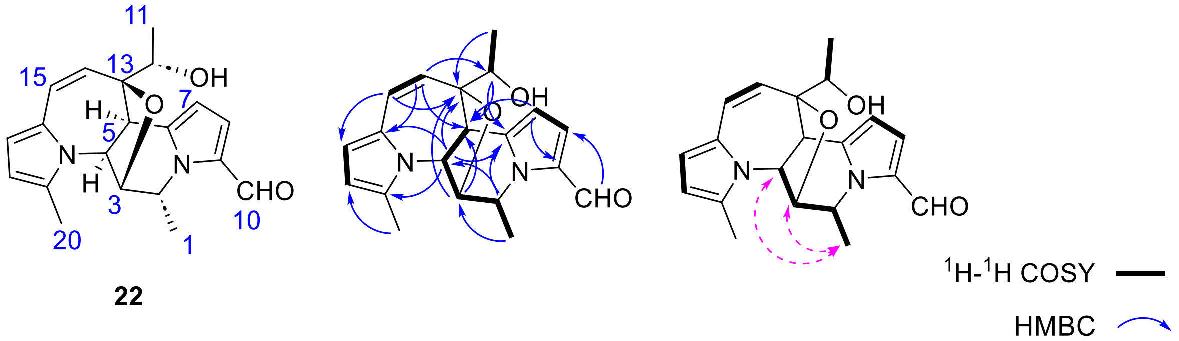

Position	δ_H (mult, J in Hz)	δ_C	HMBC	NOESY
1	1.60 (d, J = 6.6)	19.0	C3	H3, H4
2	4.63 (qd, J = 6.6, 1.8)	60.5	C4, C6	
3	4.60 (d, J = 1.8)	88.1	C5, C13	H1, H4
4	5.11 (br s)	59.2	C6, C13, C16, C19	H1
5	4.02 (br s)	45.7	C7, C13, C16	
6		141.5		
7	6.27 (d, J = 3.6)	108.8	C9	
8	7.08 (d, J = 3.6)	125.4		H10
9		131.7		
10	9.50 (s)	177.7	C8	
11	1.16 (d, J = 6.6)	17.7	C13	H8
12	2.68 (q, J = 6.6)	69.3	C5, C14	
13		89.6		
14	5.73 (d, J = 12.0)	120.2	C5, C12, C16	H11
15	6.38 (d, J = 12.0)	123.4	C13, C17	
16		130.2		
17	6.07 (d, J = 3.6)	112.9		
18	5.89 (d, J = 3.6)	108.1		H20
19		132.9		
20	2.34 (br s)	12.8	C18	

Table S22. ^{1}H and ^{13}C NMR data of curvamine P (**23**).

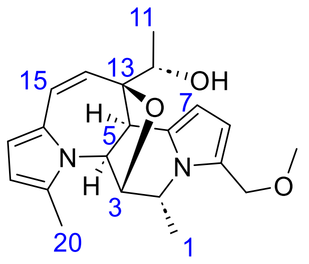

23

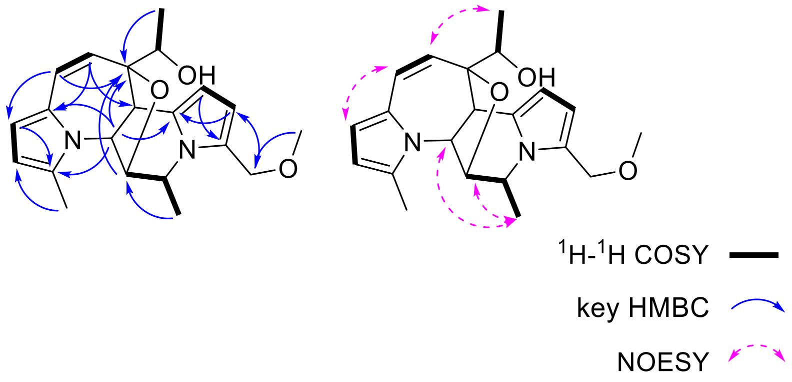

Position	δ_H (mult, *J* in Hz)	δ_C	HMBC	NOESY
1	1.55 (d, *J* = 6.5)	19.7	C3	H3, H4
2	4.31 (q, *J* = 6.5)	59.0		
3	4.45 (br s)	89.6	C13	H1, H4
4	4.98 (br s)	61.0	C6, C13, C19	
5	3.85 (br s)	46.1		
6		132.9		
7	5.98 (d, *J* = 3.0)	105.6	C9	
8	6.06 (d, *J* = 3.0)	111.9	C6	
9		129.0		
10a	4.51 (d, *J* = 12.5)	66.8	C8	
10b	4.34 (d, *J* = 12.5)			
11	1.08 (d, *J* = 6.0)	18.2	C13	H14
12	2.56 (q, *J* = 6.0)	70.0		
13		90.1		
14	5.68 (d, *J* = 11.5)	121.5	C5, C16	H11, H15
15	6.46 (d, *J* = 11.5)	124.0	C13, C17	H14, H17
16		131.3		
17	5.99 (d, *J* = 3.0)	113.4	C19	H15
18	5.84 (d, *J* = 3.0)	108.8		
19		133.5		
20	2.30 (br s)	13.6	C18	
10-OCH_3	3.23 (s)	56.6	C10	

Table S23. ^{1}H and ^{13}C NMR data of curvamine Q (**24**).

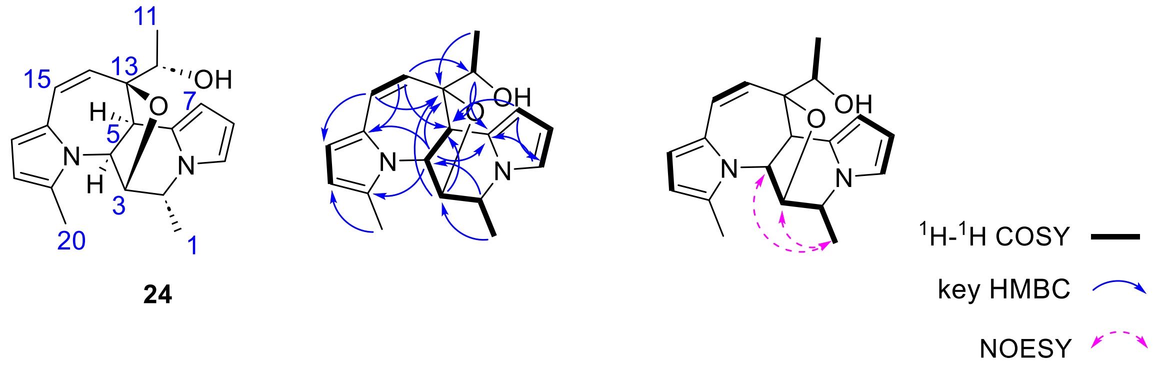

Position	δ_H (mult, J in Hz)	δ_C	HMBC	NOESY
1	1.47 (d, J = 6.5)	18.7	C3	H3, H4
2	4.15 (q, J = 6.5)	58.1	C4	
3	4.46 (br s)	89.7	C13	H1
4	5.06 (br s)	64.8	C6, C13, C16	H1, H5
5	3.86 (br s)	45.6	C3	
6		131.3		
7	6.10 (dd, J = 3.5, 2.0)	114.3	C9	H8
8	6.07 (dd, J = 3.5, 2.5)	108.0		H9
9	6.93 (dd, J = 2.5, 2.0)	126.3	C6	
11	1.09 (d, J = 6.5)	18.1	C13	H12, H14
12	2.63 (q, J = 6.5)	69.9	C14	
13		89.8		
14	6.36 (d, J = 12.0)	123.5	C5, C12, C16	H15
15	5.77 (d, J = 12.0)	123.3	C13, C17	
16		130.0		
17	6.10 (d, J = 3.0)	105.6		H18
18	5.79 (d, J = 3.0)	108.0	C16	
19		128.4		
20	2.24 (br s)	12.7	C18	

Table S24. ^{1}H and ^{13}C NMR data of curvamine R (**25**) ($CDCl_3$).

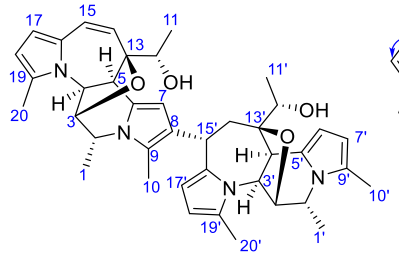

25

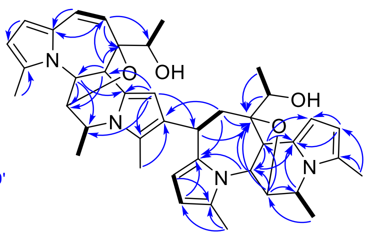

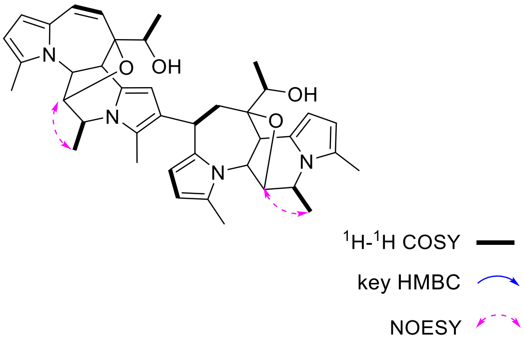

Position	δ_H (mult, *J* in Hz)	δ_C	HMBC	NOESY
1	1.47 (d, *J* = 6.8)	19.9	C3	H3
2	4.19 (d, *J* = 6.8)	57.5	C4	
3	4.49 (br s)	89.2	C5, C13	H1
4	4.89 (br s)	60.5	C5, C6, C13, C16, C19	
5	3.91 (br s)	45.4	C6, C4, C7, C13, C14	
6		130.6		
7	5.95 (br s)	103.6	C5, C6, C8, C9, C15'	
8		122.5		
9		128.4		
10	2.20 (s)	11.2	C8	
11	1.27 (d, *J* = 6.4)	17.5		
12	2.86 (m)	70.4		
13		89.6		
14	5.74 (d, *J* = 11.8)	120.0	C12, C13, C16	
15	6.43 (d, *J* = 11.8)	124.3	C16	
16		130.2		
17	6.09 (d, *J* = 3.5)	113.5	C18	
18	5.92 (overlap)	107.9	C17	
19		128.8		
20	2.25 (s)	13.9	C19	
1'	1.53 (d, *J* = 6.6)	19.5	C3'	H3'
2'	4.24 (m)	57.0	C6'	
3'	4.19 (br s)	85.0	C2', C4', C5'	H1'
4'	4.88 (br s)	61.2	C6', C13', C16'	
5'	4.35 (br s)	42.0	C4', C6', C7'	
6'		130.3		
7'	5.92 (d, *J* = 3.6)	103.1		
8'	5.91 (d, *J* = 3.6)	107.9	C6', C7'	
9'		132.6		
10'	2.26 (s)	13.0	C8', C9'	
11'	1.08 (d, *J* = 6.5)	16.8	C12', C13'	
12'	2.57 (q, *J* = 6.5)	71.2	C13', C14'	
13'		91.4		

14a'	2.12 (d, J = 14.0)	40.2	C8, C13', C16'
14b'	2.46 (dd, J = 14.0, 4.5)		
15'	4.20 (d, J = 4.5)	32.1	C9, C18'
16'		136.4	
17'	5.21 (m)	106.4	C19'
18'	5.80 (m)	106.8	C16'
19'		128.8	
20'	2.28 (s)	14.0	C18', C19'

Table S25. ^{1}H and ^{13}C NMR data of curvamine S (**26**) ($CDCl_3$).

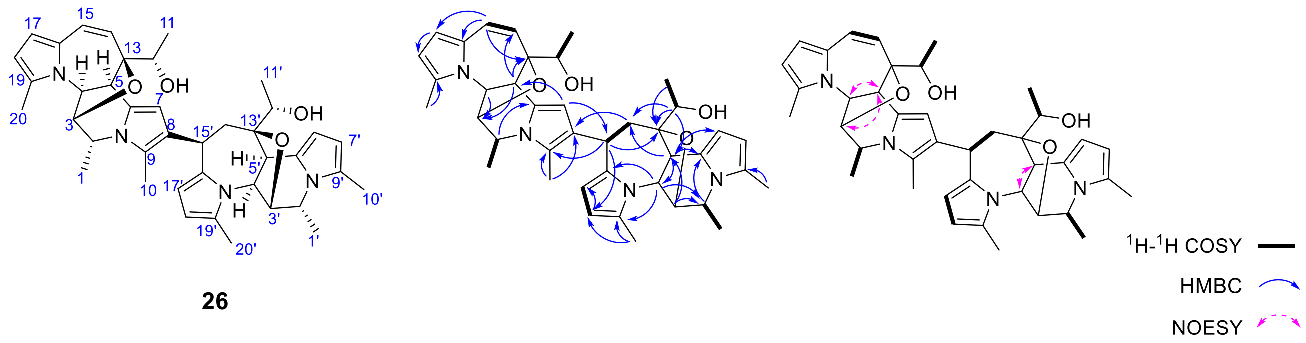

Position	δ_H (mult, *J* in Hz)	δ_C	HMBC	NOESY
1	1.50 (d, *J* = 6.6)	19.9	C6	
2	4.20 (d, *J* = 6.6)	57.4		
3	4.49 (br s)	89.6		H5
4	4.95 (br s)	60.5	C3	H5
5	3.90 (br s)	45.0	C3, C13, C14	H4
6		128.4		
7	5.87 (m)	103.8	C15’	
8		124.2		
9		136.1		
10	2.20 (s)	11.2	C8, C9	
11	1.23 (d, *J* = 6.4)	17.4		
12	2.72 (m)	70.4		
13		89.2		
14	5.71 (d, *J* = 11.7)	120.0		
15	6.40 (d, *J* = 11.7)	124.2	C13, C14, C15	
16		130.6		
17	6.10 (m)	113.4	C18	
18	5.93 (d, *J* = 3.8)	108.6		
19		132.6		
20	2.31 (s)	14.0	C18, C19	
1'	1.47 (d, *J* = 6.6)	19.0		
2'	4.35 (m)	58.3	C6'	
3'	4.52 (br s)	85.3	C2', C5'	
4'	4.92 (br s)	59.1	C2', C19'	H5'
5'	3.44 (br s)	49.2	C4', C6', C7'	H4'
6'		130.6		
7'	5.87 (d, *J* = 3.8)	103.8		
8'	5.93 (d, *J* = 3.8)	108.6		
9'		129.4		
10'	2.27 (s)	13.9	C9'	
11'	1.14 (d, *J* = 6.5)	17.8	C13'	
12'	2.97 (q, *J* = 6.5)	71.8	C13', C14'	
13'		91.1		

14'	2.10 (d, J = 11.5)	37.5	C8
15'	4.25 (d, J = 11.5, 1.5)	31.4	C9, C18'
16'		130.8	
17'	5.87 (m)	104.0	
18'	5.89 (m)	107.3	C17'
19'		128.4	
20'	2.29 (s)	12.9	C18', C19'

Table S26. ^{1}H and ^{13}C NMR data of curvamine T (**27**) ($CDCl_3$).

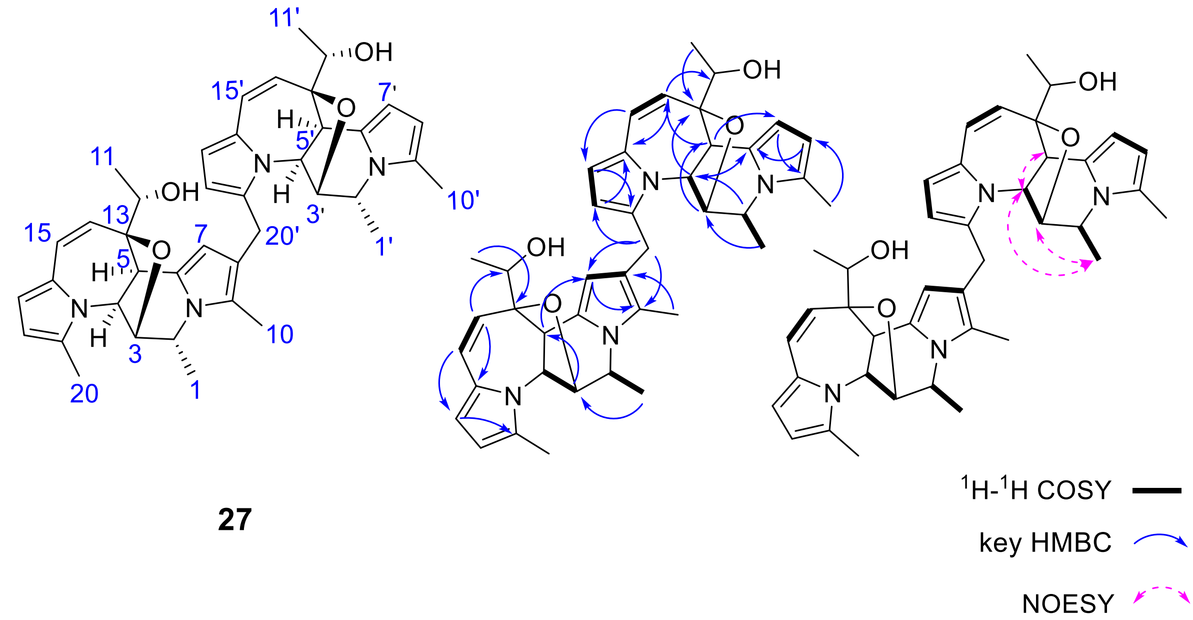

Position	δ_H (mult, *J* in Hz)	δ_C	HMBC	NOESY
1	1.50 (d, *J* = 6.8)	19.8	C2, C3	
2	4.19 (m)	57.6	C3, C4	
3	4.48 (br s)	89.2	C2, C4, C5, C13	
4	4.91 (br s)	60.6	C3, C5	
5	3.93 (br s)	45.2	C3, C4, C6, C7	
6		130.6		
7	5.95 (s)	114.6	C8, C9, C20	
8		118.0		
9		128.7		
10	2.16 (s)	11.3	C8, C9	
11	1.22 (d, *J* = 6.4)	17.4	C12′, C13′	
12	2.67 (m)	70.3		
13		89.5		
14	5.71 (d, *J* = 11.6)	119.8	C5, C12, C13, C16	
15	6.37 (d, *J* = 11.6)	124.2	C16, C17	
16		129.9		
17	6.10 (d, *J* = 3.6)	113.5	C15, C16, C19	
18	5.93 (m)	108.6		
19		118.0		
20	2.19 (s)	11.3	C18, C19	
1′	1.49 (d, *J* = 6.8)	19.9	C2′, C3′	H2′, H3′, H4′
2′	4.19 (m)	57.6	C3′, C4′	
3′	4.43 (br s)	89.4	C2′, C4′, C5′, C13′	
4′	4.93 (br s)	60.4	C5′, C6′, C13′	H5′
5′	3.93 (br s)	45.2	C3′, C4′, C6′, C7′, C14′	H4′
6′		130.4		

7′	5.89-5.93 (m)	103.8	C9′	
8′	5.82 (d, *J* = 3.4)	108.0	C6′	
9′		128.7		
10′	2.28 (s)	12.9	C8′, C9′	
11′	1.20 (d, *J* = 6.4)	17.4	C12′, C13′	
12′	2.67 (m)	70.3		
13′		89.5		
14′	5.69 (d, *J* = 11.6)	119.8	C5′, C12′, C13′, C16′	
15′	6.42 (d, *J* = 11.6)	124.3	C13′, C16′, C17′	
16′		129.4		
17′	5.90 (m)	119.8	C15′, C18′, C19′	
18′	5.92 (m)	108.6	C20′	
19′		132.4		
20′a	3.74 (d, *J* = 16.8)	24.6	C7, C9, C18′, C19′	H7
20′b	3.67 (d, *J* = 16.8)			

Table S27. ^{1}H and ^{13}C NMR data of curvamine U (**28**) ($CDCl_3$).

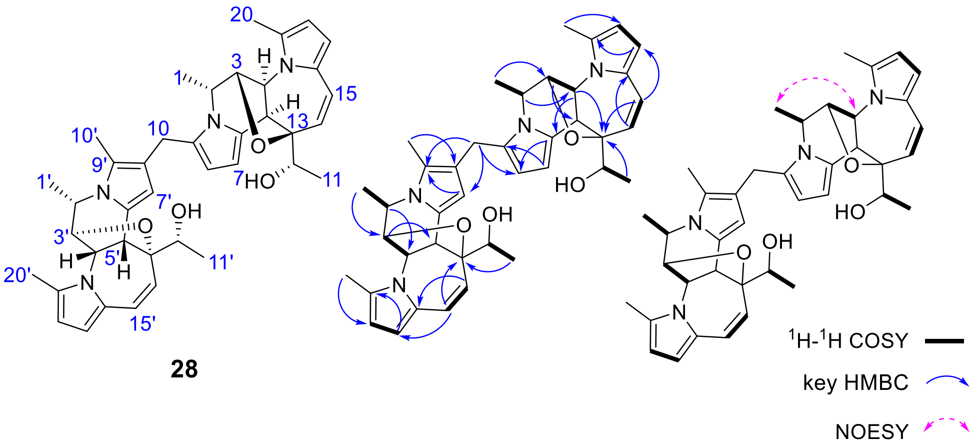

Position	δ_H (mult, J in Hz)	δ_C	HMBC	NOESY
1	1.49 (d, J = 6.8)	19.7	C2, C3	H4
2	4.14 (dd, J = 6.8, 1.9)	57.6	C3, C4	
3	4.46 (br s)	89.1	C2, C4, C5, C13	
4	4.90 (br s)	60.5	C6, C13	H20, H1
5	3.91 (br s)	45.2	C3, C4, C6	H4
6		130.6		
7	5.91 (m)	103.7	C6, C9	
8	5.92 (m)	108.7	C6, C9	
9		132.6		
10a	3.70 (d, J = 16.5)	25.1	C7′, C8, C9, C8′, C9′,	
10b	3.82 (d, J = 16.5)			
11	1.19 (d, J = 6.4)	17.4	C12, C13	
12	2.62 (m)	70.5		
13		89.7		
14	5.66 (d, J = 11.6)	119.7	C13, C16	
15	6.41 (d, J = 11.6)	124.3	C13, C16, C17	
16		130.6		
17	6.09 (m)	113.6	C16, C19, C18	
18	5.92 (d, J = 3.0)	108.5	C16, C17, C19	
19		132.6		
20	2.26 (s)	13.9	C19, C18	H7
1′	1.47 (d, J = 6.8)	19.7	C2′, C3′	
2′	4.14 (q, J = 6.8)	57.6	C4′, C3′	
3′	4.40 (br s)	89.0	C5′, C2′, C4′, C13′	
4′	4.90 (br s)	60.3	C3′	
5′	3.86 (br s)	45.0	C3′, C4′, C6′	
6′		128.7		
7′	5.81 (s)	105.1	C9′, C8′, C6′	
8′		117.6		
9′		124.9		

10′	2.13 (s)	10.5	C8′, C9′	
11′	1.16 (d, *J* = 6.4)	17.4	C12′, C13′	
12′	2.62 (m)	70.5		
13′		89.4		
14′	5.68 (d, *J* = 11.6)	118.6	C13′, C16′	H15′
15′	6.40 (d, *J* = 11.6)	124.4	C13′, C16′, C17′	H14′
16′		130.5		
17′	6.09 (m)	113.5	C18′, C16′, C19′	
18′	5.90 (d, *J* = 3.0)	108.6	C17′, C16′, C19′	
19′		132.6		
20′	2.26 (s)	13.9	C18′, C19′	

Supplementary Figures

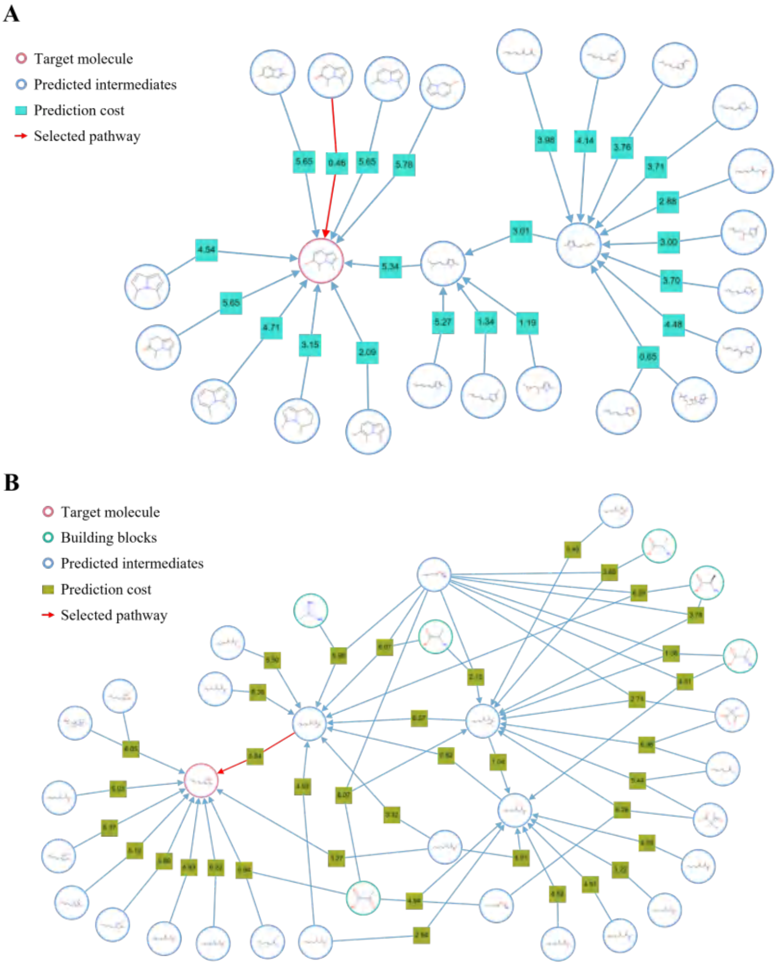

Fig. S1. The bioretrosynthesis prediction of nitrogenated monomers **1** (A) and **2** (B) by BioNavi-NP. The most possible (or top1) pathway was colored in red. The cost of each reaction step is reflected by the confidence score (smaller prediction cost means higher reaction probability).

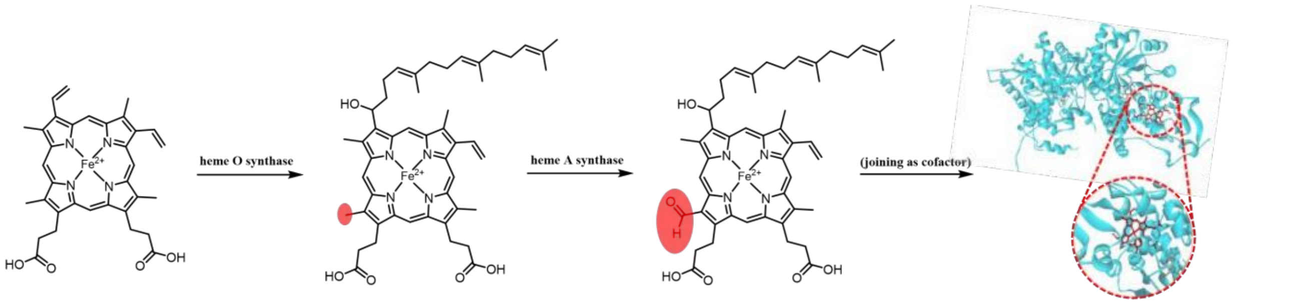

heme B **heme O** **heme A** **cytochrome *c* oxidase (COX)**

Fig. S2. The Cox15 proteins catalyze the heme O conversion into heme A. The methyl in heme O was oxidized to the aldehyde group in heme A. Heme A is an indispensable cofactor for cytochrome *c* oxidase (COX) that is vital for the respiration of aerobic organisms.

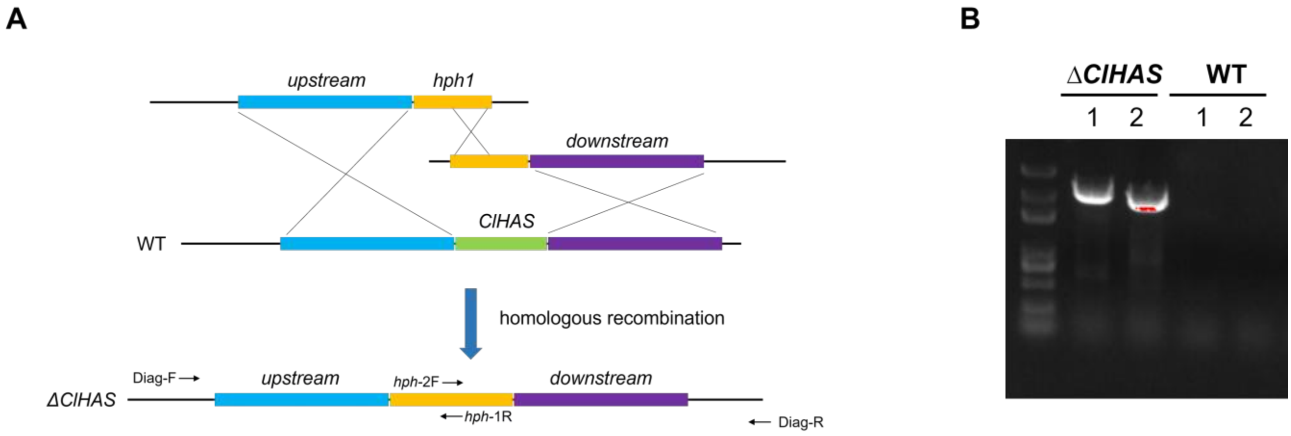

Fig. S3. Construction of *ΔClHAS* mutant of *C. lunata*. (A) The substitution of *ClHAS* using *hph* as the selective marker. (B) Diagnostic PCR for confirming the mutation. Fragment 1 was amplified using primers *Cl*HAS-Diag-F and hph-1R, and fragment 2 was done utilizing primers hph-2F and *Cl*HAS-Diag-R.

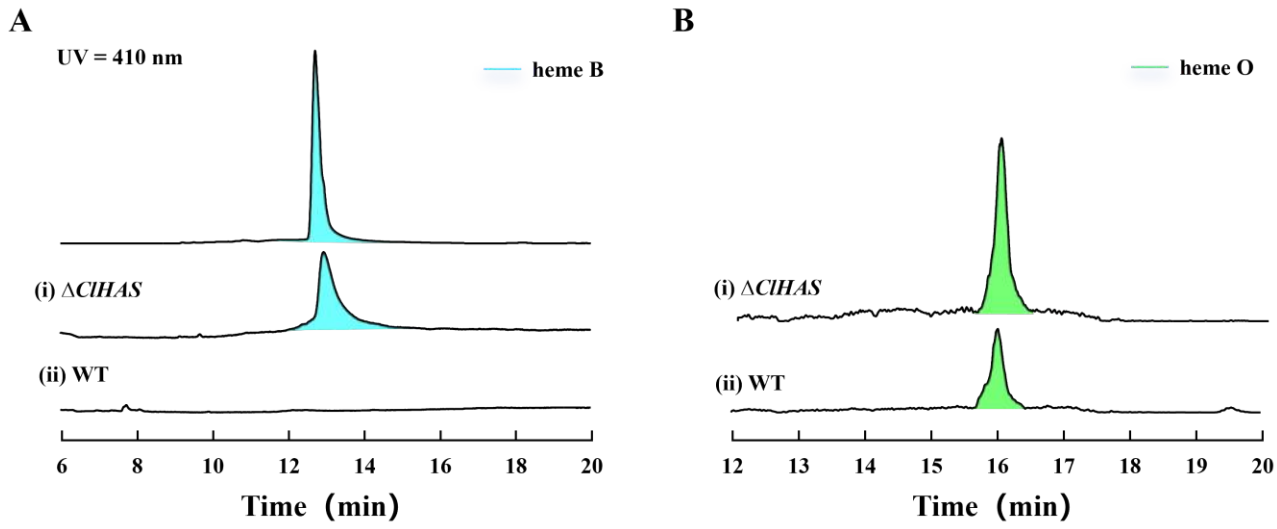

Fig. S4. The analysis of hemes B and O components in *ΔClHAS* and WT strains. (A) HPLC analysis of heme B in *ΔClHAS* and WT strains. (B) LC-HR/MS analysis of heme O in *ΔClHAS* and WT strains.

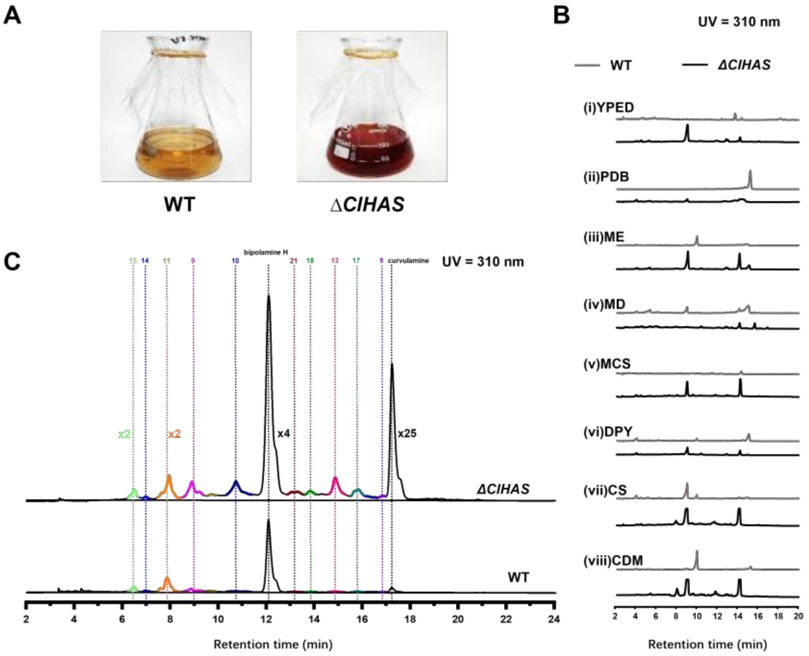

Fig. S5. The fermentation broth and HPLC profiles of *ΔClHAS* and WT strains in different media. (A) The flask growth tests showed enhanced SM production by the mutant relative to WT. (B) HPLC profiles of the two strains in different media. (C) HPLC profiles of the two strains cultured in CDM. Colors indicate *ΔClHAS*-derived compound numbers as in the text.

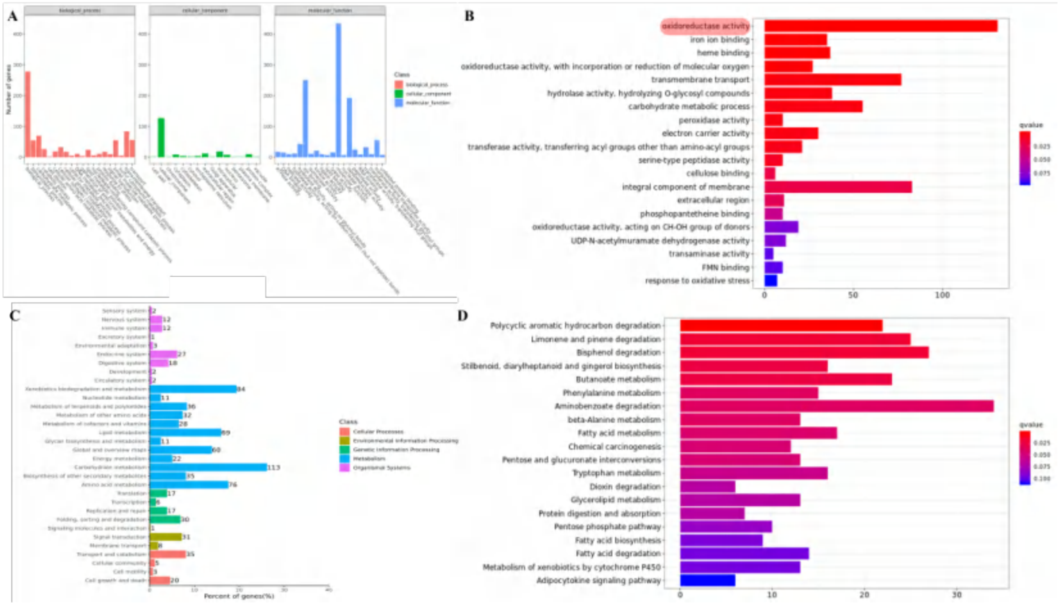

Fig. S6. The analysis of RNA-seq data from the Δ*ClHAS* and WT strains. (A-B) The GO analysis of the 356 genes with changed expression magnitude. The putative 66 oxidoreductases were highlighted in red. (C-D) The KEGG analysis of genes governing differentiated SM biosynthesis. All data are presented as the mean of three RNA-seq experiments.

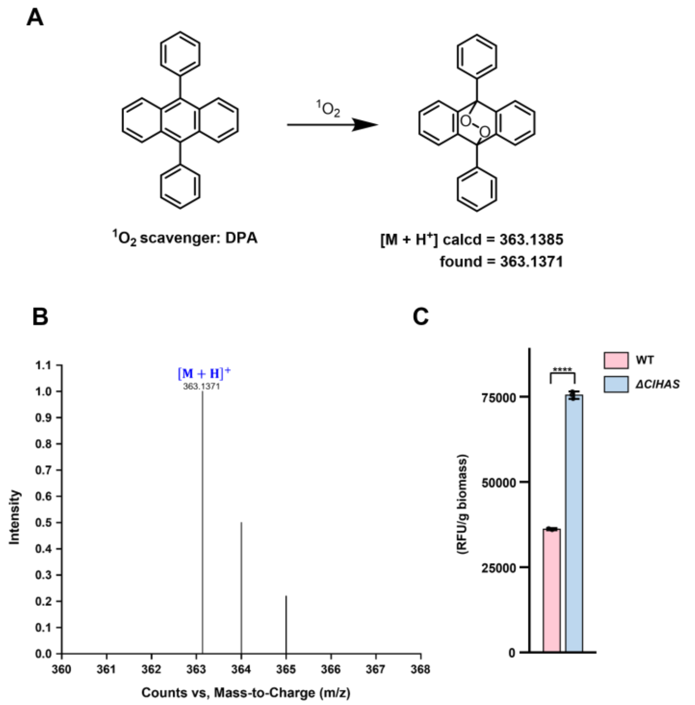

Fig. S7. The difference in the $^1O_2$ content between *ΔClHAS* and WT strains cultured for 4 days. 9,10-Diphenylanthracene (DPA) captures $^1O_2$ by forming a DPA derivative with a peroxy-bridge (A), which was detected by LC-HR/MS (B). (C) The relative fluorescence unit (RFU) for each gram of biomass was measured using an enzyme marker at 634 nm. $^1O_2 + {}^1O_2 \longrightarrow {}^3O_2 + h\nu$ (λ = 634 nm) [39]

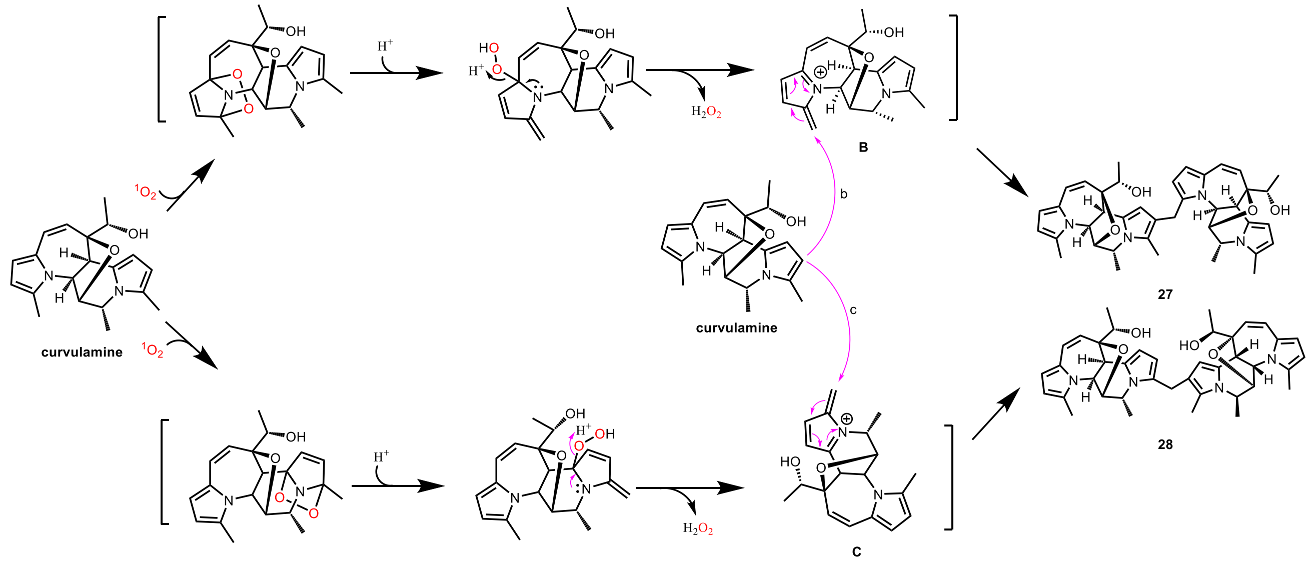

Fig. S8. The proposed formation process of curvamine T (**27**) and curvamine U (**28**).

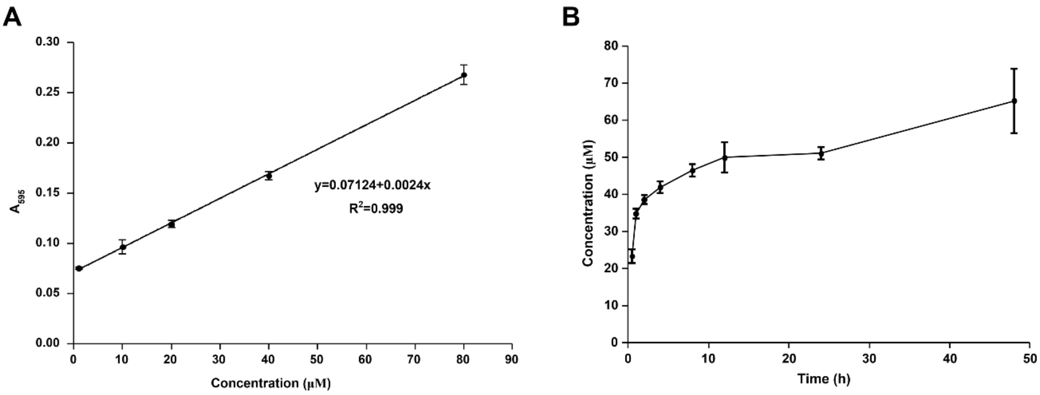

Fig. S9. Measurement of the H_2O_2 content. (A) Standard curve for the H_2O_2 analysis. (B) H_2O_2 measurements during the reaction process leading to curvamine T (**27**) and curvamine U (**28**).

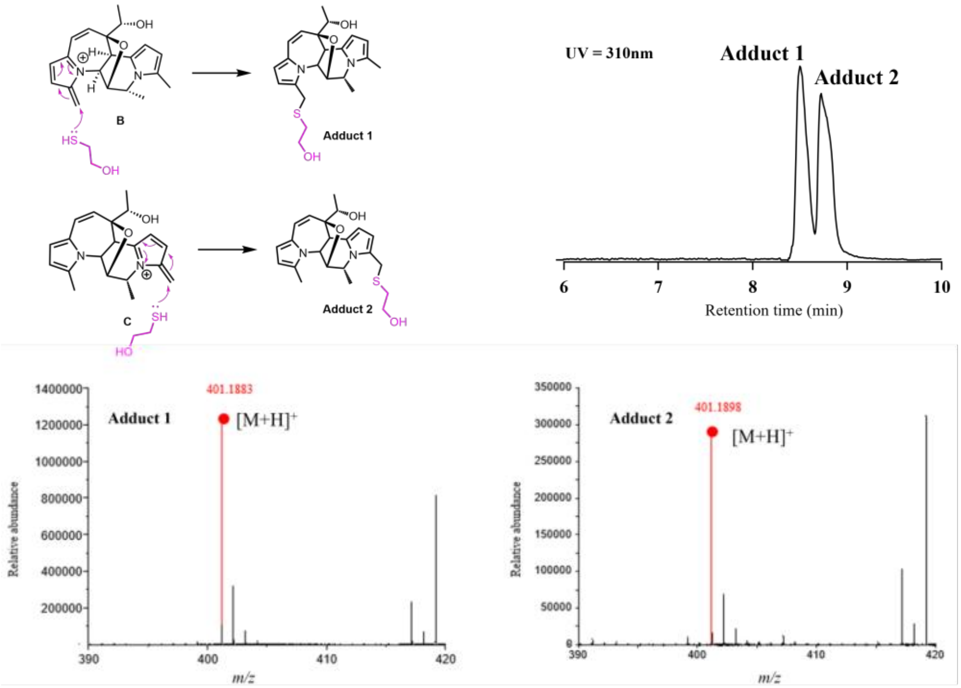

Fig. S10. LC-HR/MS detection of adducts **1** and **2** resulting from the addition of curvulamine with *β*-mercaptoethanol.

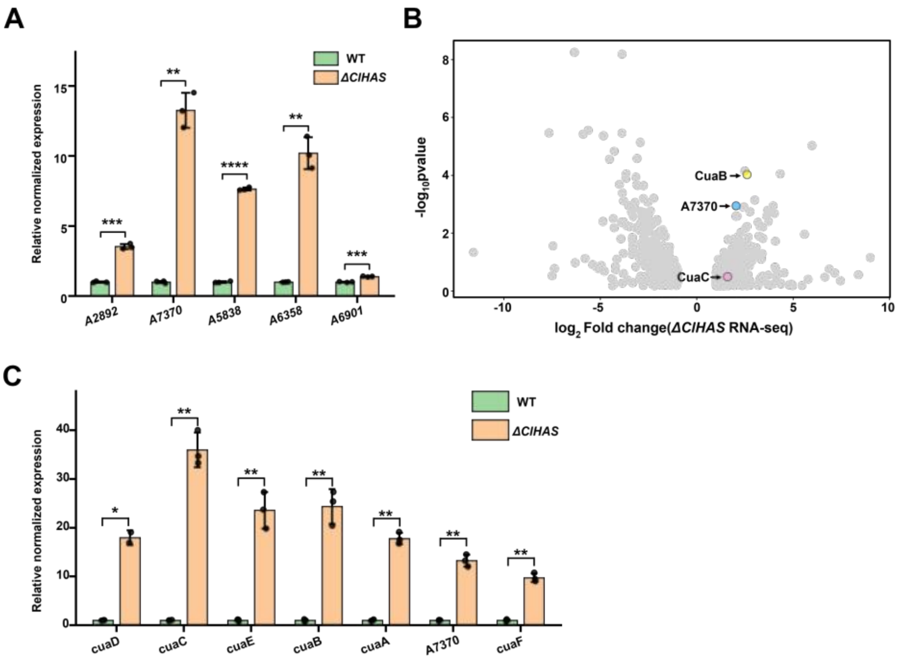

Fig. S11. RT-qPCR analysis of gene expressions associated with indolizidine alkaloid biosynthesis in WT and *ΔClHAS* strains after a 4-day cultivation. All data represent the mean of three biological replicates, with statistical significance indicated by *p< 0.1, **p< 0.01, ***p< 0.001, and ****p< 0.0001. (A) A7370 and other bZIP proteins were expressed differently between the two strains. (B) Transcriptomic data revealing that the mutation enhanced the expression of *A7370*, *cuaB*, and *cuaC* genes (log_2fold change>2). (C) Concerted upregulation of genes in the *cua* gene cluster.

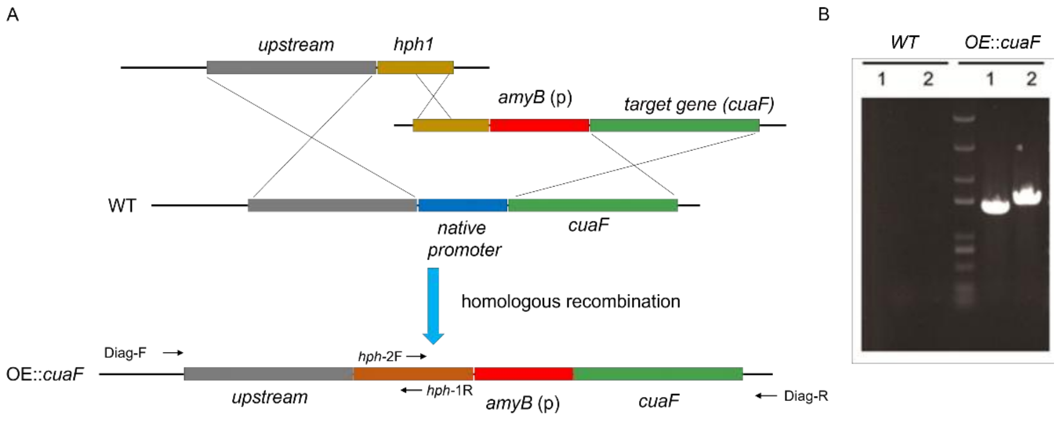

Fig. S12. Construction of the OE::*cuaF* strain of *C. lunata*. (A) The substitution of the native promoter of *cuaF* using *hph* as a selective marker. (B) Diagnostic PCR conducted to confirm the mutation. Fragment 1 was amplified using primers OE-cuaF-Diag-F and hph-1R, and fragment 2 was done utilizing primers hph-2F and OE-cuaF-Diag-R.

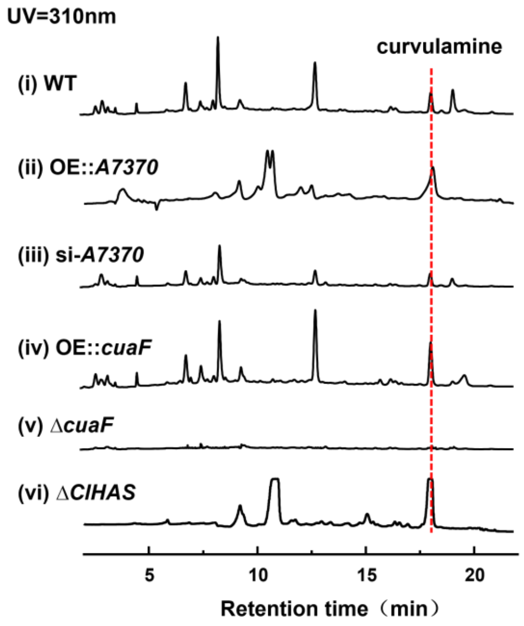

Fig. S13. HPLC profiles of the WT, OE::*A7370*, si-*A7370*, OE::*cuaF*, Δ*cuaF*, and Δ*ClHAS* strains after an 11-day fermentation in CDM.

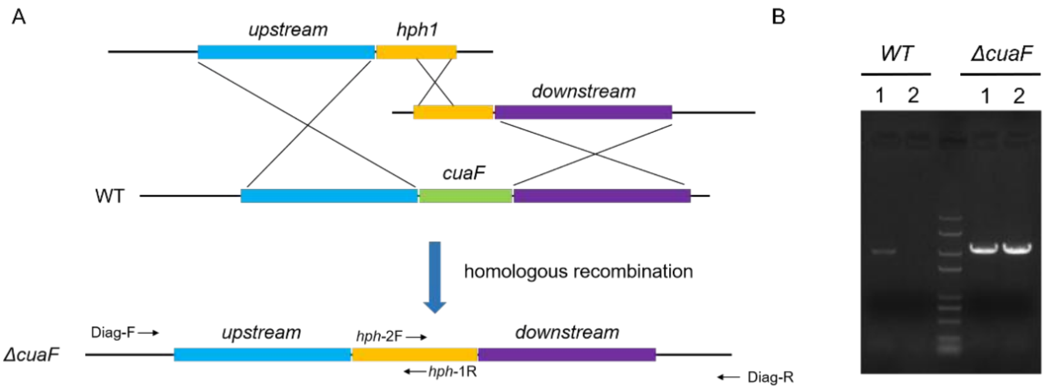

Fig. S14. Construction of the *ΔcuaF* mutant of *C. lunata*. (A) The substitution of *cuaF* using *hph* as a selective marker. (B) Diagnostic PCR for confirming the mutation. Fragment 1 was amplified using primers *cuaF*-Diag-F and hph-1R, and fragment 2 was done utilizing primers hph-2F and cuaF-Diag-R.

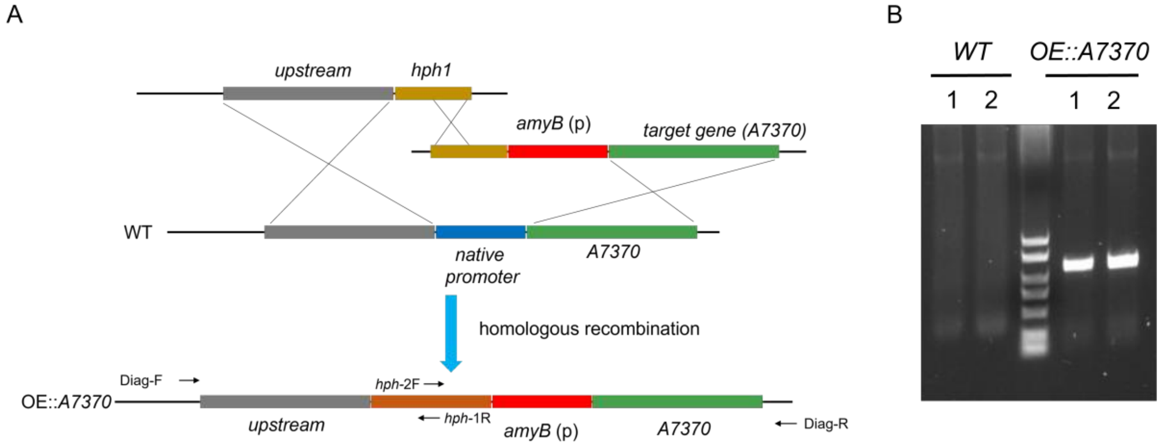

Fig. S15. Construction of the OE::*A7370* strain of *C. lunata*. (A) The substitution of the native promoter of *A7370* (bZIP transcription factor) using *hph* as a selective marker. (B) The mutation confirmed by diagnostic PCR. Fragment 1 was amplified using primers OE-A7370-Diag-F and hph-1R, and fragment 2 was done utilizing primers hph-2F and OE-A7370-Diag-R.

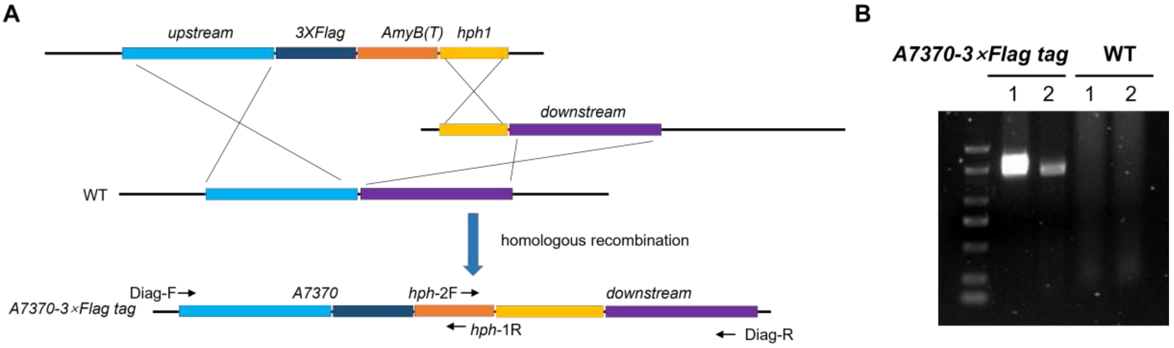

Fig. S16. Construction of the *A7370*-3×*Flag* tag strain of *C. lunata*. (A) The substitution of the stop codon (TGA) of *A7370* using 3×Flag tag, T_{AmyB}, and *hph* used as a selective marker. (B) The mutation confirmed by diagnostic PCR. Fragment 1 was amplified using primers A7370-3×Flag tag-Diag-F and hph-1R, and fragment 2 was done utilizing primers hph-2F and A7370-3×Flag tag-Diag-R.

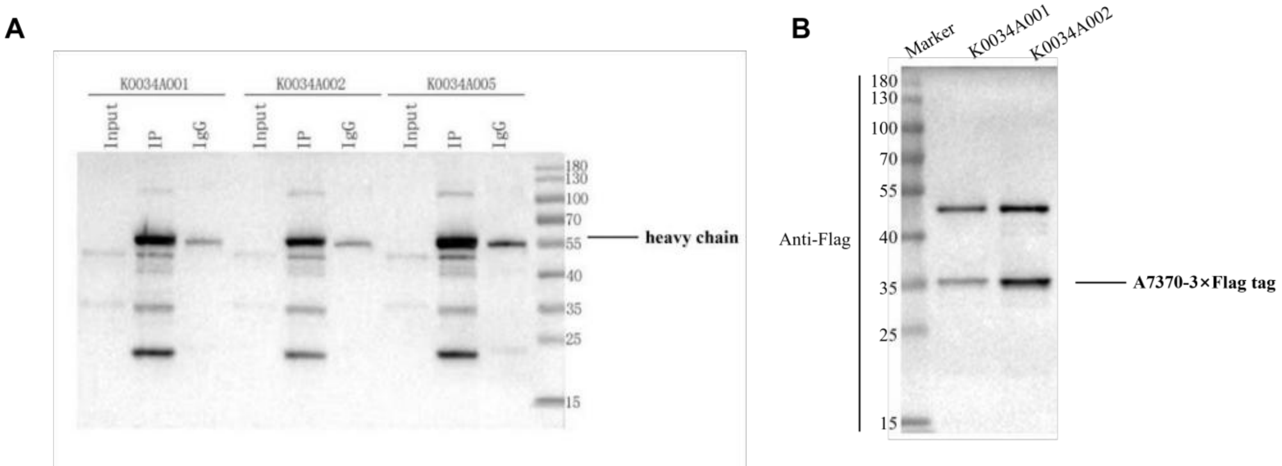

Fig. S17. ChIP-seq sample verification. (A) Heavy chain bands of IgG from three biological replicates. (B) Western blot analysis of A7370-3×Flag tag protein.

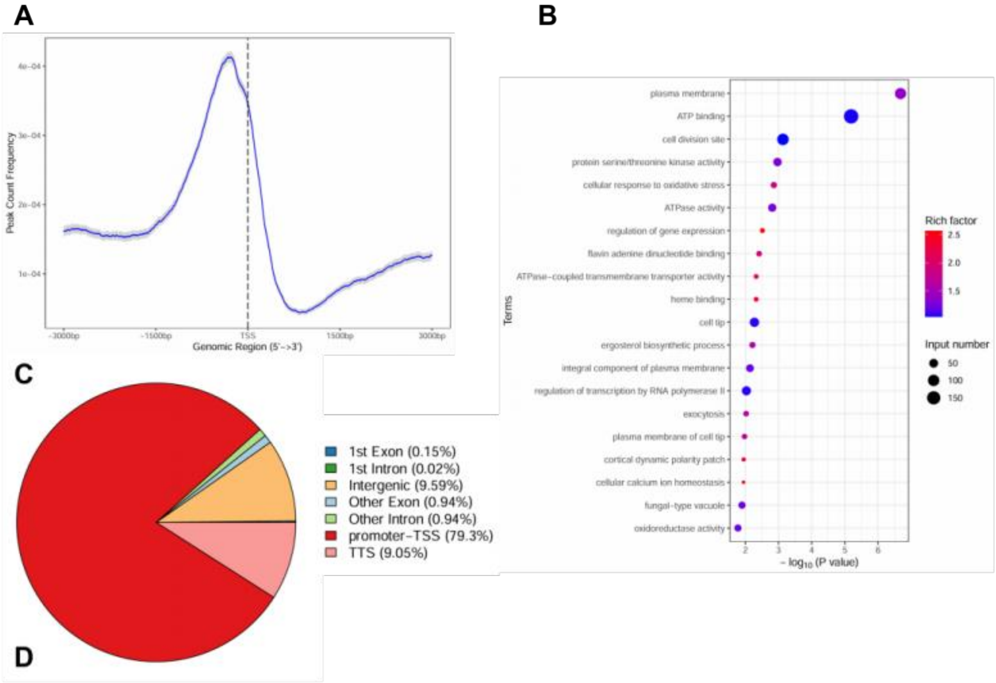

>Z10_peak_4982
GTTAGAGTTTACCACGAAGCATTCTCACGAGTGCTAGTAATTAGGGTTAATGCGATGGCTTGGAAATTTTCCAACCACAC
ACGACTCCAGCAGCGAACAAGAGAGGATTACATCTCTCCCTTCAAGACGGCACTCCAATCCTCACCGCGGAAGAATTC
CTCCAAAGAAGTCGTCTTGATGCCTAGCTCCGCCGGGGTCTTGACCTTGGGATTACCACCAGCATAGCCGTATTCTGAAA
AGAATTTGAGATCGTCTATCAAGGTGGCCTCGAAGCCTTCCGGGACATACTTCAAGAACTCTTCCTCGGGGACATCTCT
GCAAGTTGCCTTGACGCCCATGGTGCGACCCCAAAGAGCCGCGTAGTCTGGCTTAGTGATGATCTCGCTGGCGCCCAGG
ACGTCGGTGCCAGGTGGATGGTGAAGGACCAGCGCTTCAACAAAGGCACCCGTGTCGCGAGTGGCCACTACCTCAGG
GTTTGCCTTGTGTGGGCCGGGACAATCGAGACGCACGTATTCAAAAGTACCGTCGGGCAGTTTATGAGGCCGGAAAGC
GGGAATTTCCTTCCAAGTCTCCTGGAAGATACCCATGTTGACGGTGCTCAGGCGGTTCCGCAGCTCTGGAAATCGTTCT
CTCATGTACTGCTCCGCGCCAGCCTTGGAGTCAAACTCGTACGAGTTCTTGTACTTGCCGCCGCTGATCTGCTTGACTGG
GGCTAATGTAGAGAAGACGAATTTCTCTAGAGTCGAAAGGACTTTCGGGTCACTGGCGGCCGTTGCCACGTTGATGCCT
TGGTGCTTTTCCAGGTGTCCAGCATATTCCTCGAATGACATGCCAGCCTTGTTTGCTTCGTCTTGTAGATTTTTGTTTTCT
GAAACCCGTGAGAAGCAGGCGGCCCAATCAGTAACGGCGAAGATGGCATGTGCACCTTGGAAAGCAGCTTTGAGCGAT
TCCAGATCGTCGTGGTCGCCTTTGACAATTTCAACGCCTTTGCTCGCCCAATCCTTGGCTTTAGCGGAACCGGGGTTGCG
CGTCAGTCCACGTACGCGCCACTCAGAGTGGTAAAGAAATCGACGGGCAACAGAGCCACCCTAGAGCTTGCGTCAGTG
TGGGTCTGACGTGAGATGACTAGGAATCAACATACCTGCGTTCCAGTGATGCCCAGAACAGTGATGAGCTTCTTTGTCA
TGATGTGCTTGTTTGGTTGCGGCTGAAAGTGTGACTGACTAGGTTGACTTGTGAGCTGGGAAATTTCAAGAGGGTGCAT
TTATCTATGCTCGACCAGTGTCAGACCCCGTTTACGTGAGCGTGTGGCACGGTCCTTAGTGGATGTGTATAGTAGTCTCG
GCGCATTATCATGCGCTGGCTGACGGTGCCAGGAGTGACAGGATGAAGCCGTAGGCCAAGTACGAGGAGTTCATTGTTG
TGAGAAAGAGCCTCAAGAGCAGCTACTACTATCCAGGCTTAGGGTCGTGACACTAAGACAATTCGGGAAAGAGCTGTG
AGAAAGGCGATAATCAACGTCTTCCCATTGGCCCGTCAGGCGACACAAGATGCTGCAAGTGGACGACGTTTTGGCGGC
CCGTGTCAGGGGATGTTATGCAAGTGGACGACGCTGACCCGCTTTTTTCTCTACATGGAGCGCACATCACCTGACCCCG
TTGTATGCACATTGGTGACGTGACTTGCACTCTGCAACGTCTCCCCATCCAACGCAGACGGCTGACATTGTGCGGAACC
AGGACCCCCCACCACTCCCCACACTAGTGCCATTTCACATGTACATGCAACCGACAGTTACCTCGTACGTTAACATGGGC
CGACCCGGAGGGCGCTACGGGCCCGTACCGCCGTTGTAGAC

Fig. S18. ChIP-seq assay showing the genes regulated by A7370. (A) Peaks distribution diagram. Peaks represent regions of significant enrichment resulting from the alignment of DNA reads to the genome sequence. TSS: transcription start site. (B) GO enrichment analysis of putative A7370 target genes from ChIP-seq. (C) Distribution of A7370 binding sites across different genomic functional regions. TTS indicate transcription termination sites. Exon signifies the coding region, whereas Intron signifies the non-coding region. Intergenic refers to regions between different genes. The percentage numbers represent the ratios of binding sites in each region. (D) The region of DNA fragments with the strongest binding affinity to A7370 is located 1375 bp upstream of *cuaF*.

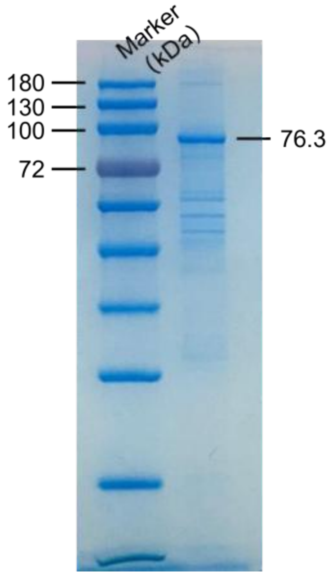

Fig. S19. The SDS-PAGE of recombinant A7370 (marked with a mycelin basic protein (MBP) tag).

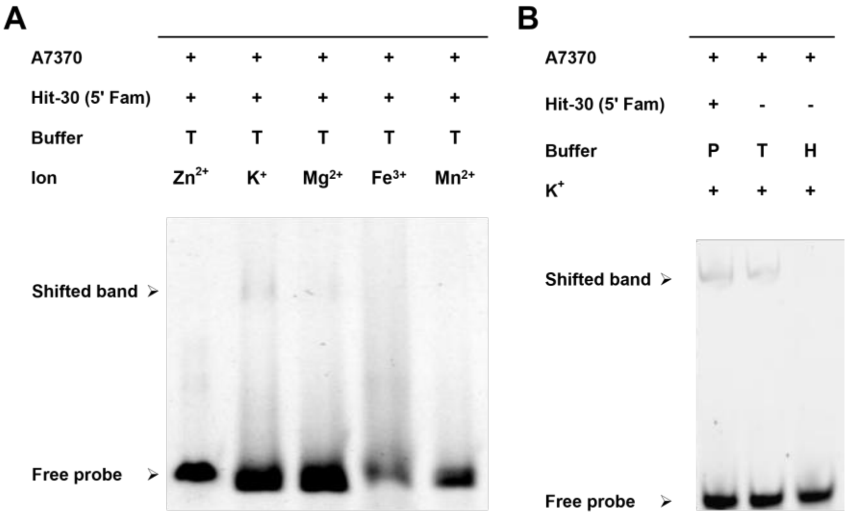

Fig. S20. Optimization of the binding of A7370 with ACGGCTGAC. (A) Screening of different metal ions in Tris-HCl (abbreviated as T) buffer. Zn^{2+}, K^{+}, Mg^{2+}, Fe^{3+}, and Mn^{2+} were assessed as given in lanes 1–5, respectively. (B) Screened buffers included PBS (abbreviated as P, lane 1), Tris-HCl (abbreviated as T, lane 2), and HEPES (abbreviated as H, lane 3).

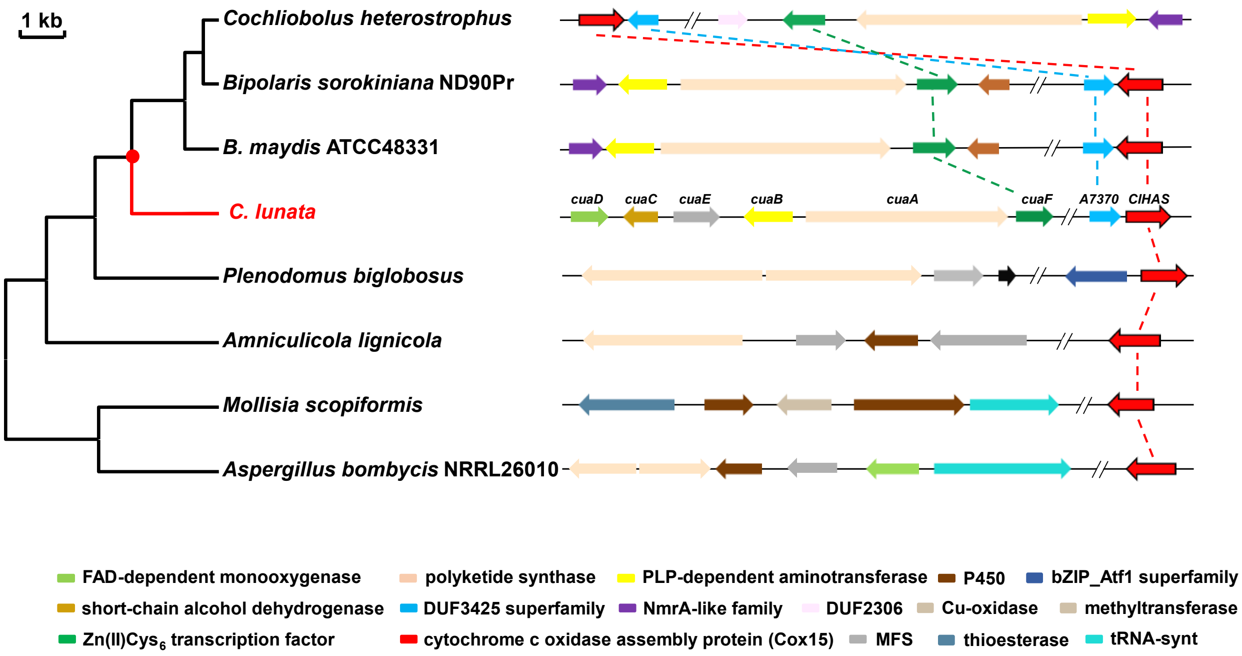

Fig. S21. Genome mining of *Cl*HAS homologs-containing BGCs.

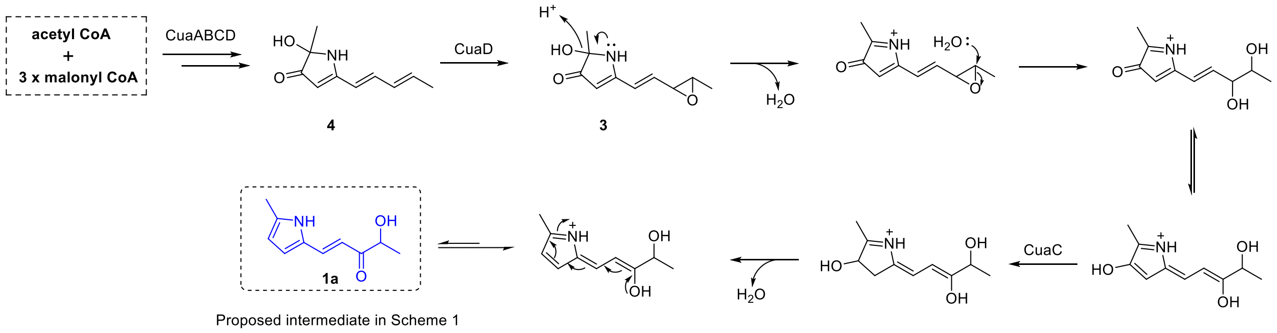

Fig. S22. Proposed **4**-to-**1a** transformation.

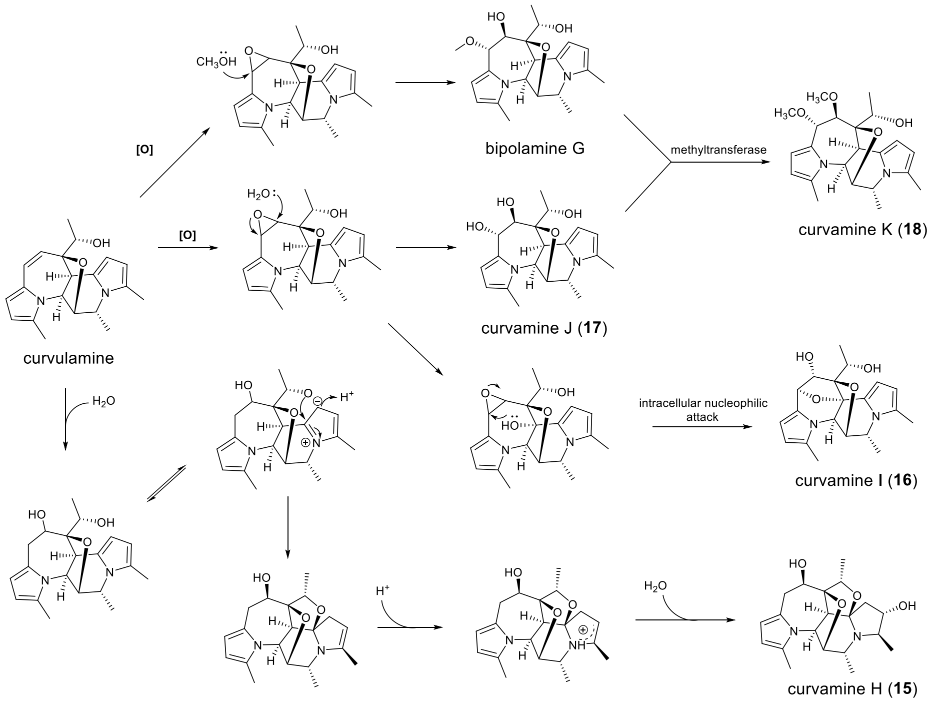

Fig. S23. The proposed biosynthetic pathways of curvamines H–K (**15–18**).

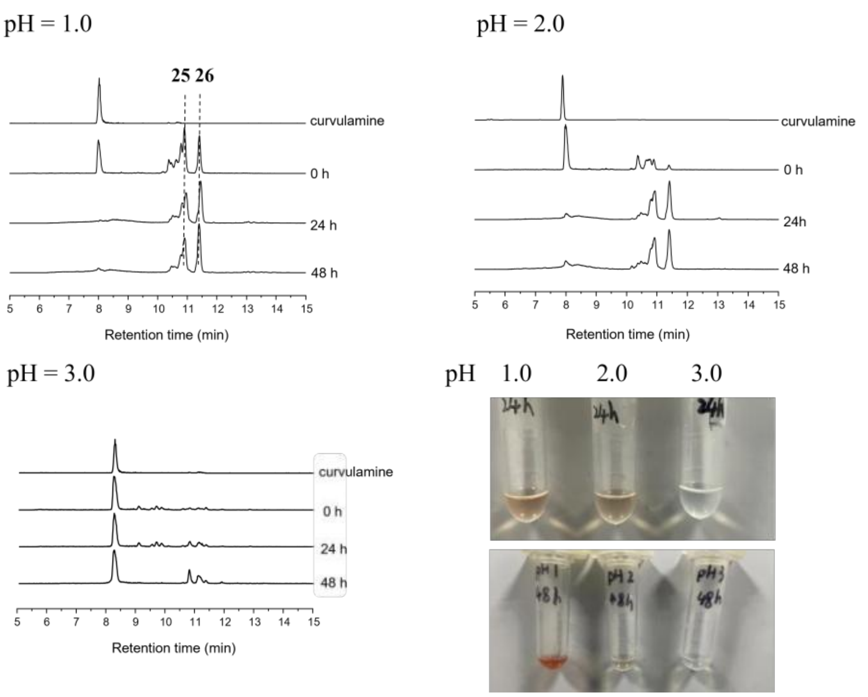

Fig. S24. The acid-promoted formation of curvamines R (**25**) and S (**26**) from curvulamine.

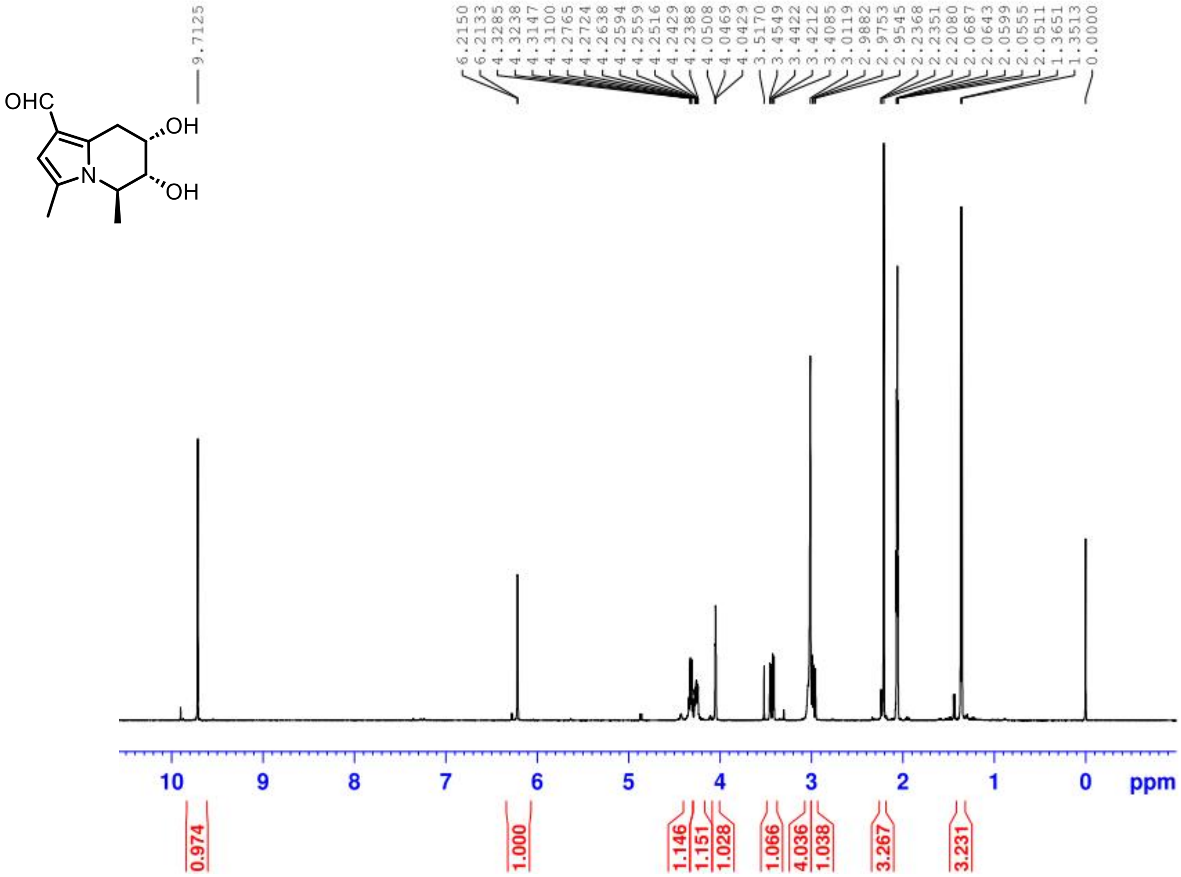

Fig. S25. ^{1}H NMR spectrum of curvamine A (**8**) (500 MHz).

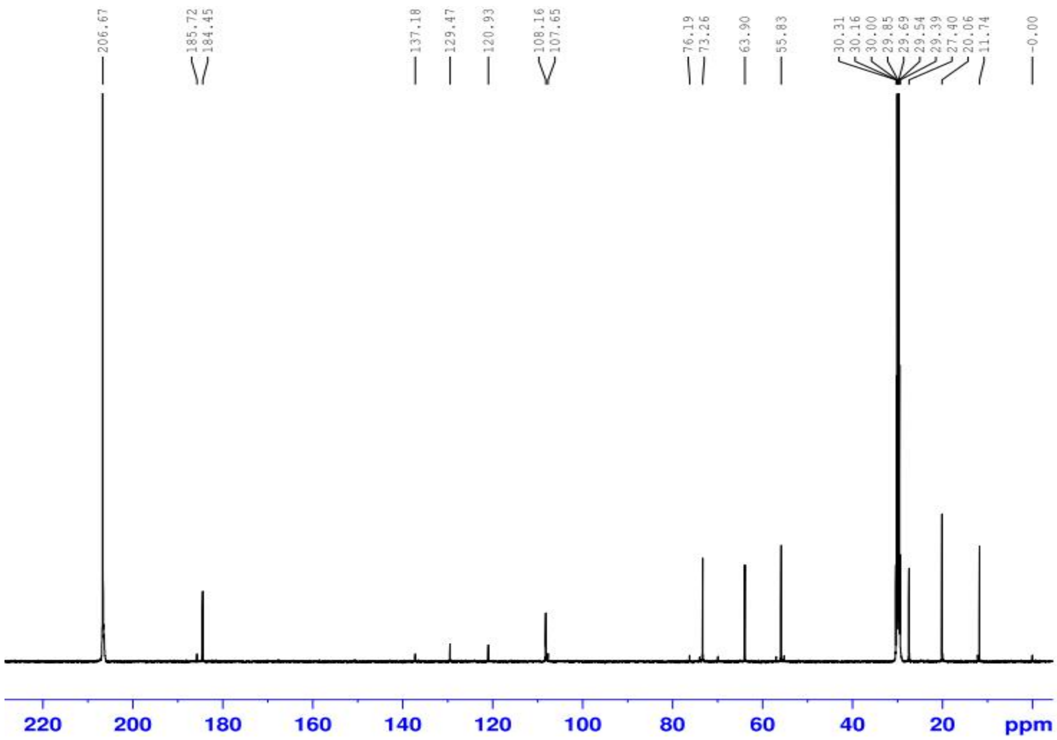

Fig. S26. ^{13}C NMR spectrum of curvamine A (**8**) (125 MHz).

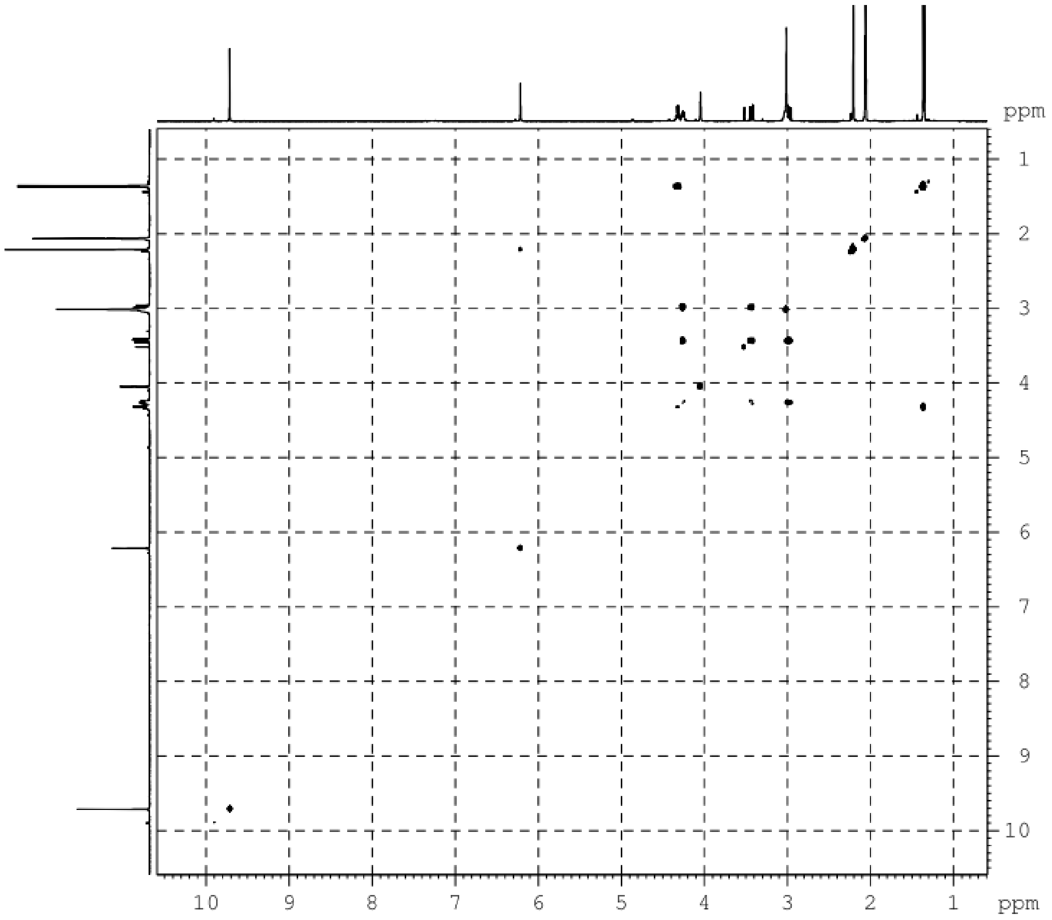

Fig. S27. ^{1}H-^{1}H COSY spectrum of curvamine A (**8**).

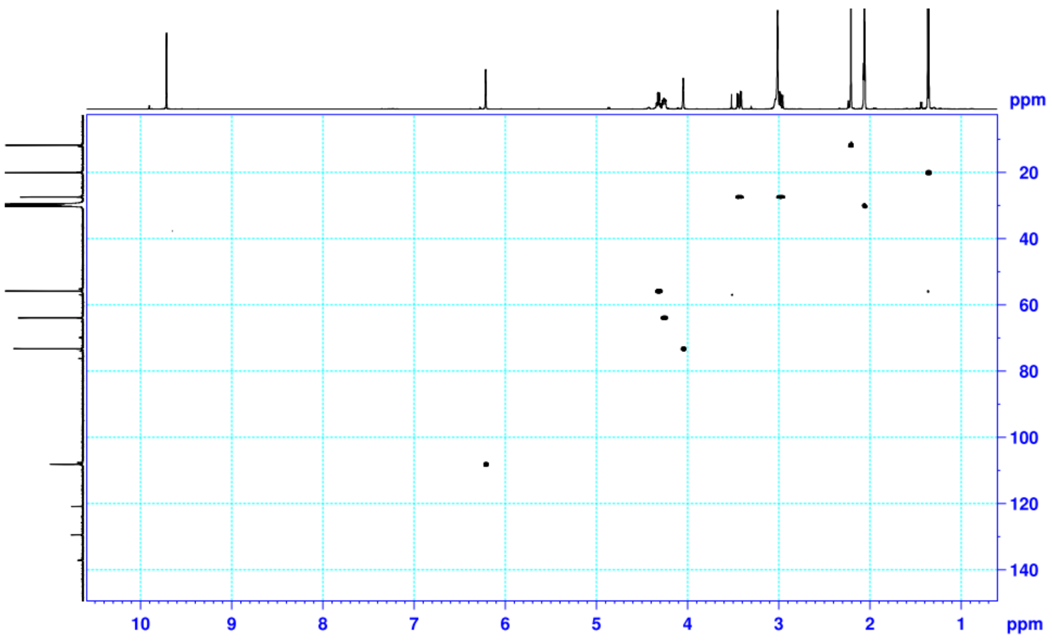

Fig. S28. HSQC spectrum of curvamine A (**8**).

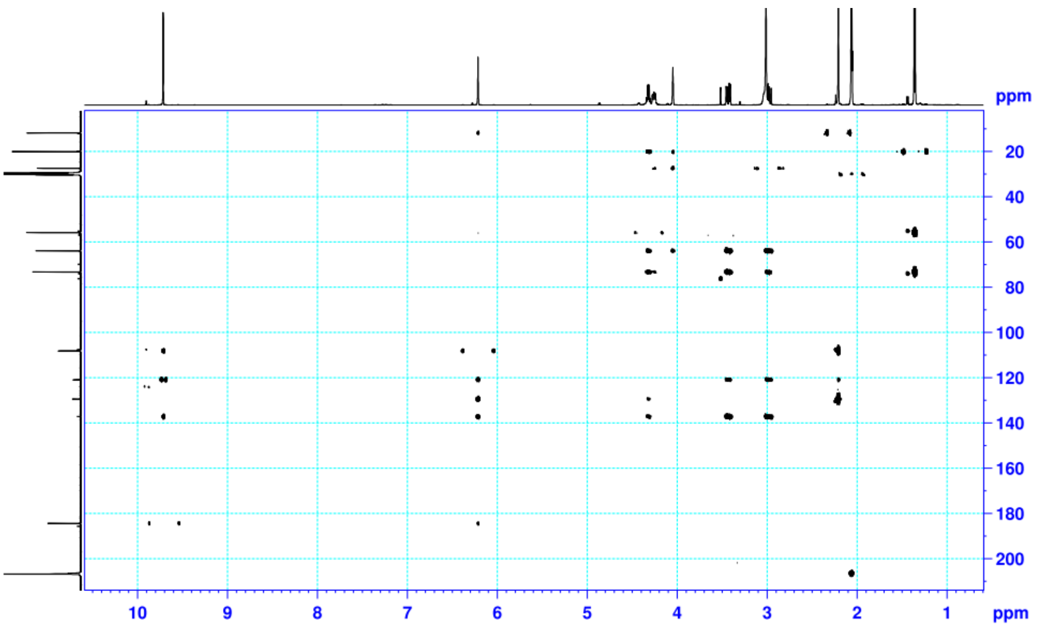

Fig. S29. HMBC spectrum of curvamine A (**8**).

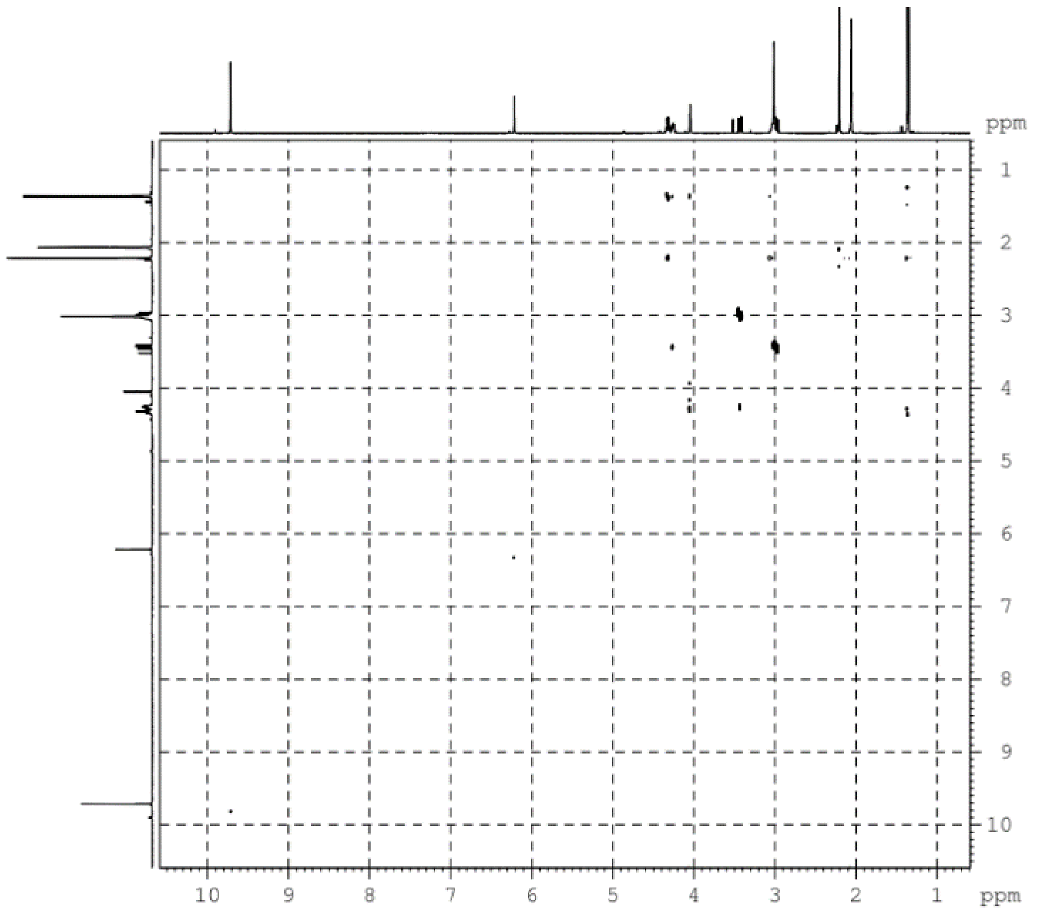

Fig. S30. NOESY spectrum of curvamine A (**8**).

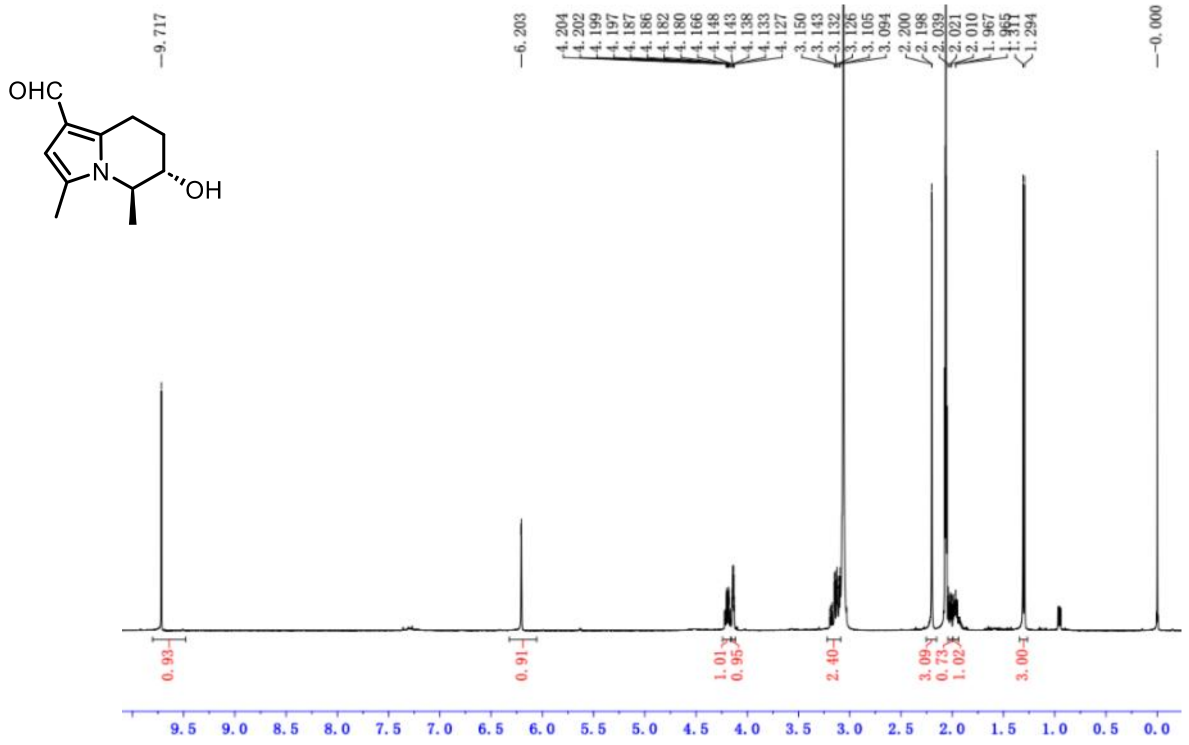

Fig. S31. ^{1}H NMR spectrum of curvamine B (**9**) (500 MHz).

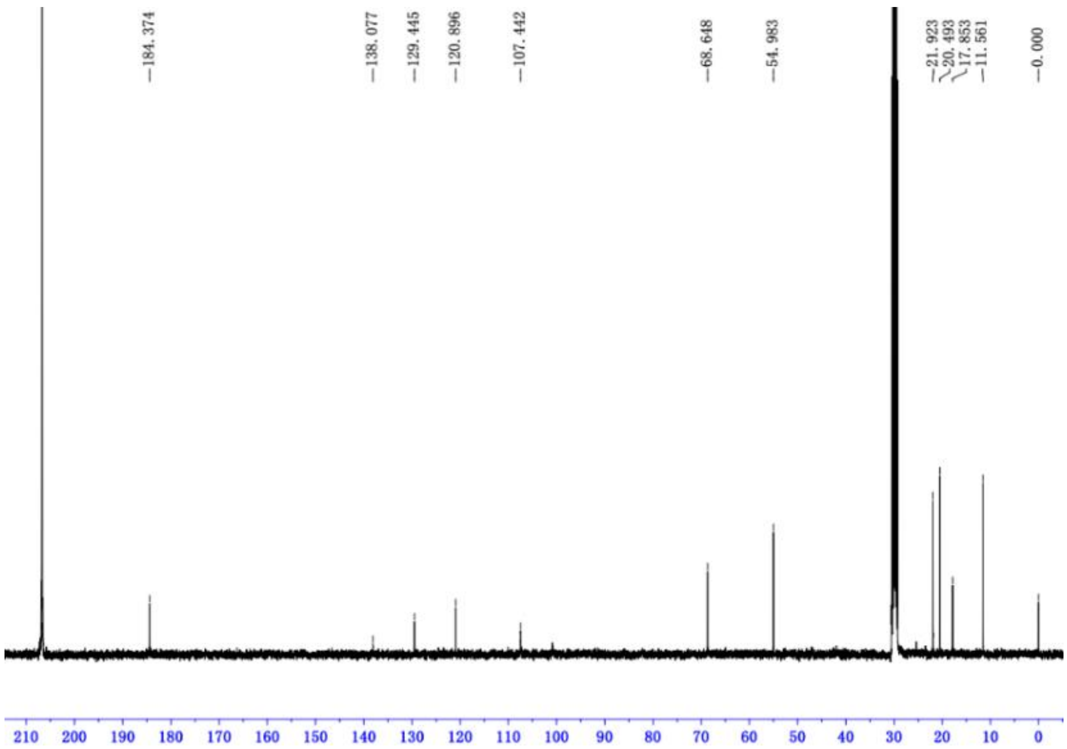

Fig. S32. ^{13}C NMR spectrum of curvamine B (**9**) (125 MHz).

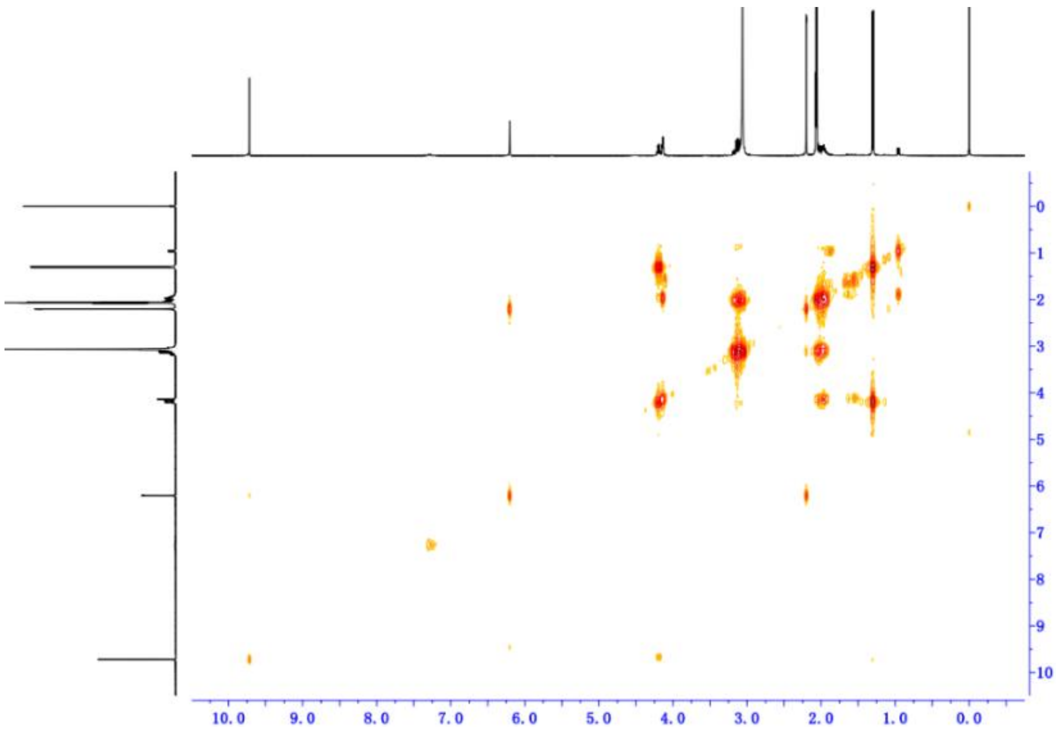

Fig. S33. ^{1}H-^{1}H COSY spectrum of curvamine B (**9**).

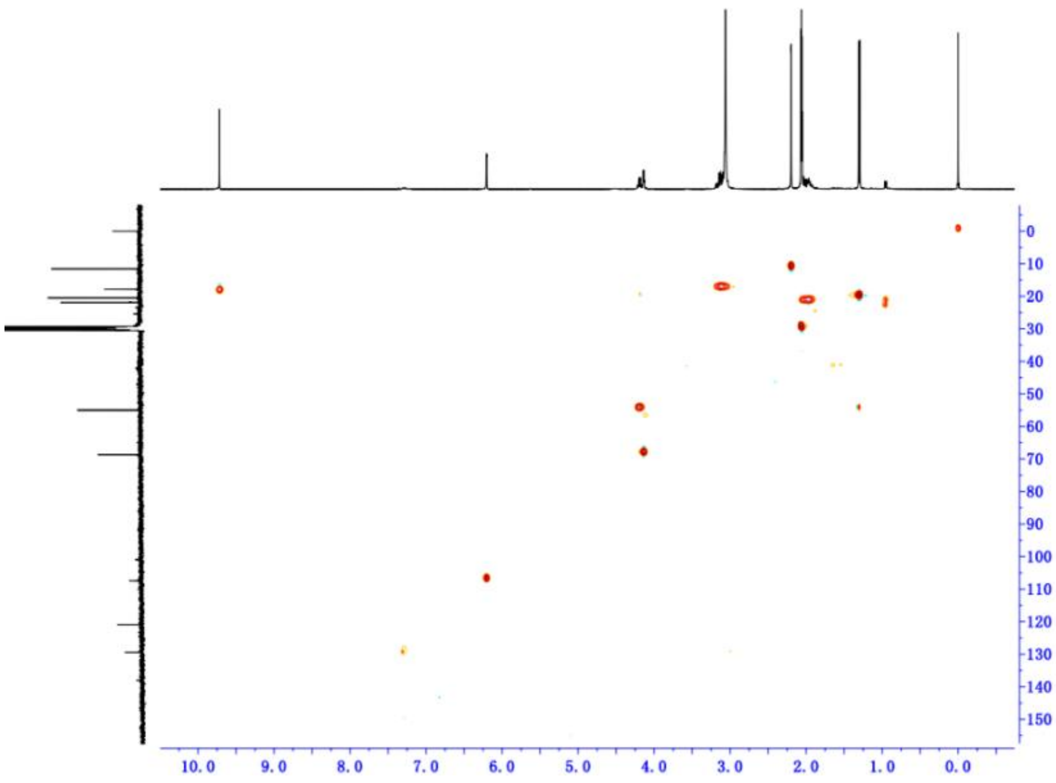

Fig. S34. HSQC spectrum of curvamine B (**9**).

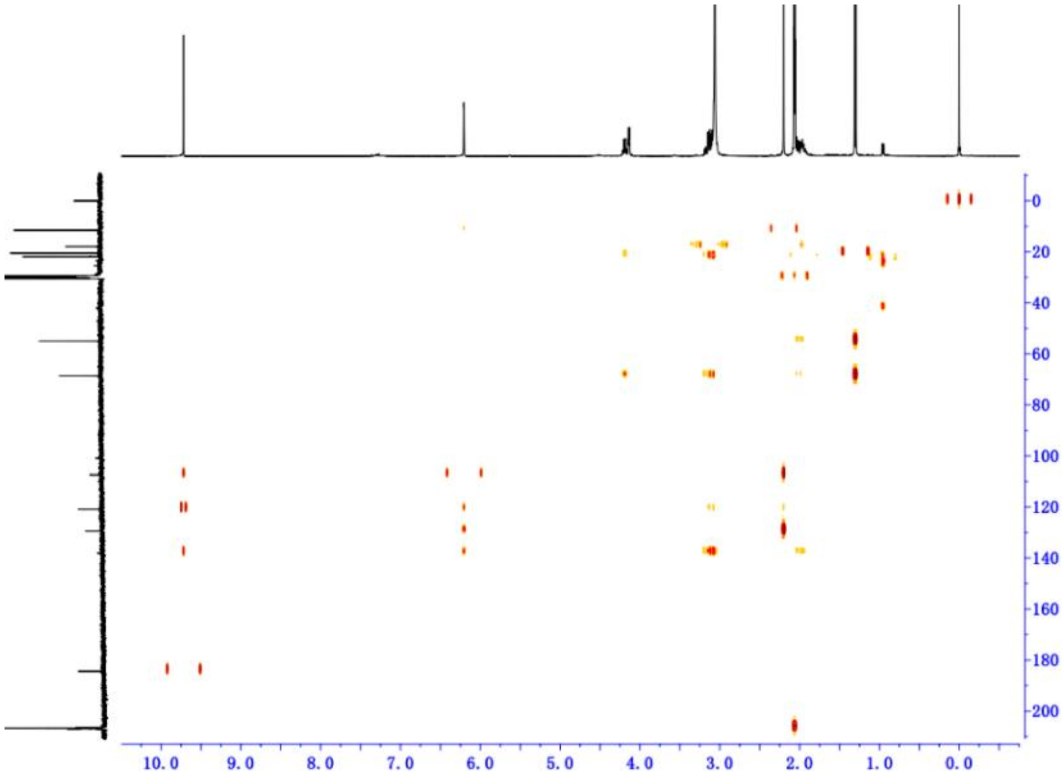

Fig. S35. HMBC spectrum of curvamine B (**9**).

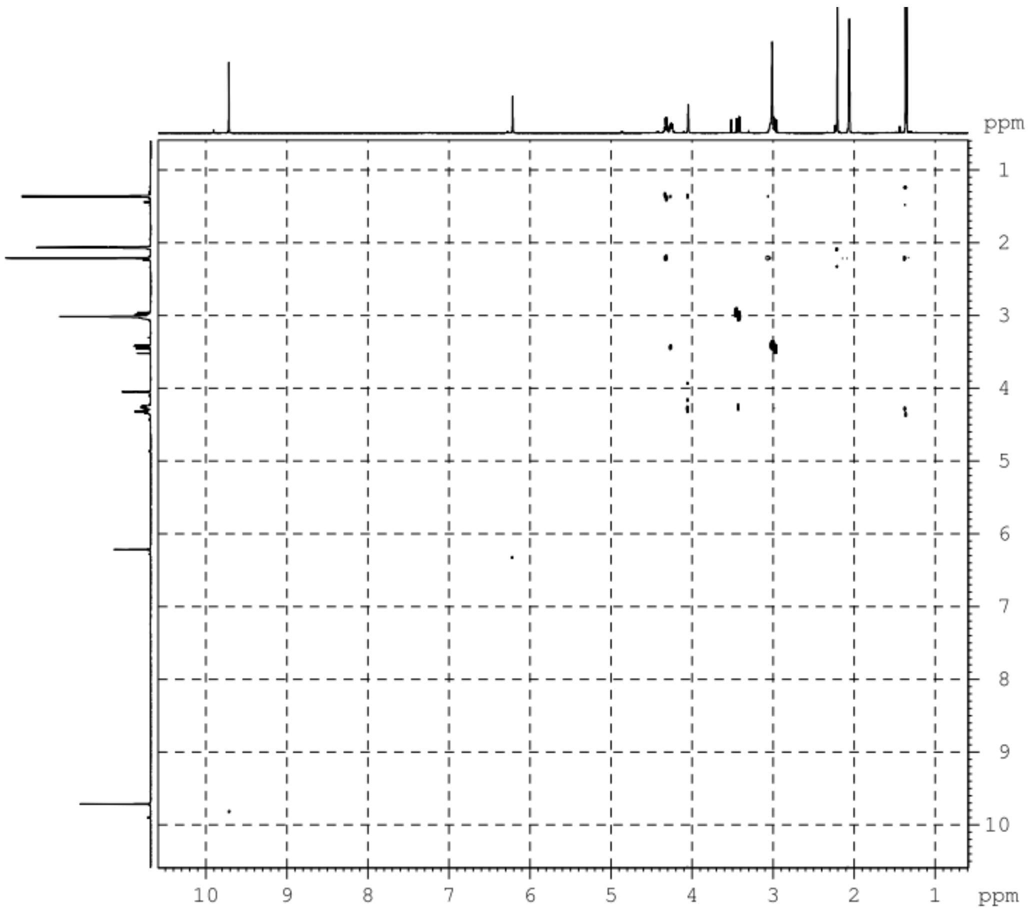

Fig. S36. NOESY spectrum of curvamine B (**9**).

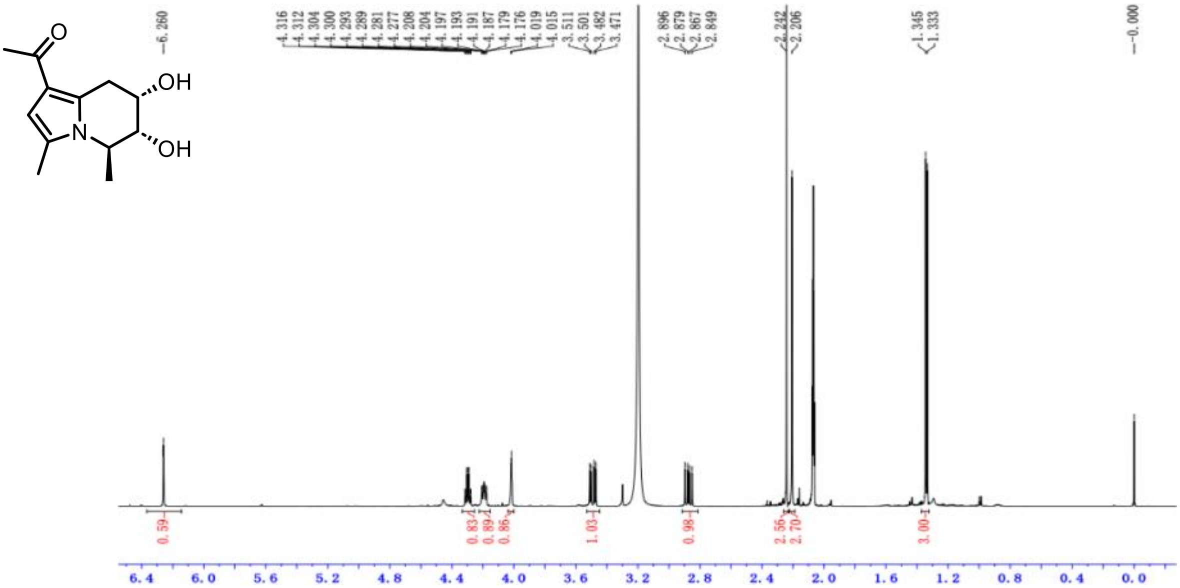

Fig. S37. ^{1}H NMR spectrum of curvamine C (**10**) (600 MHz).

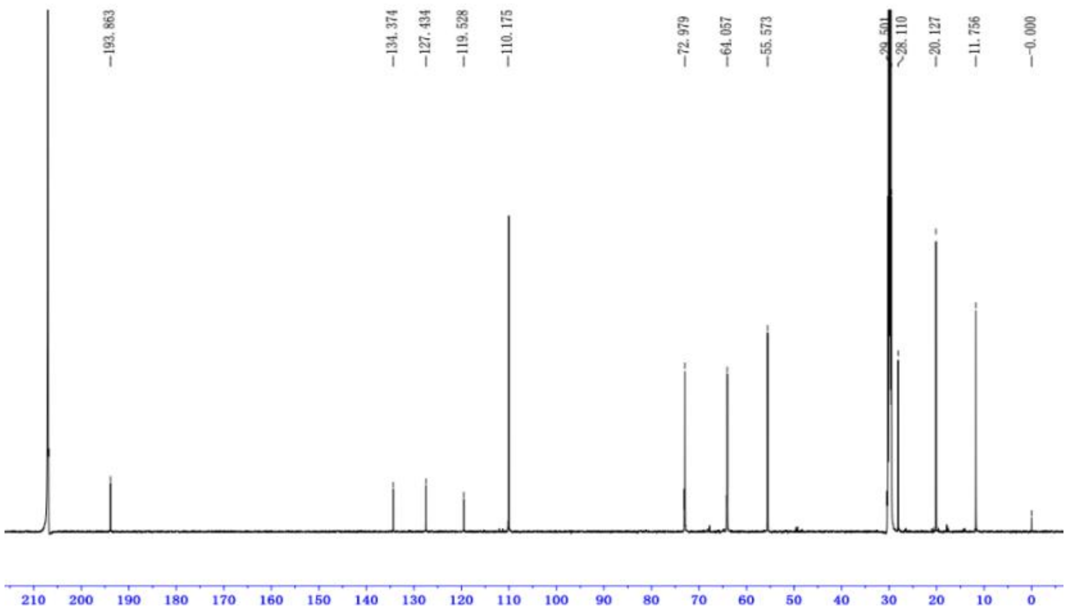

Fig. S38. ^{13}C NMR spectrum of curvamine C (**10**) (150 MHz).

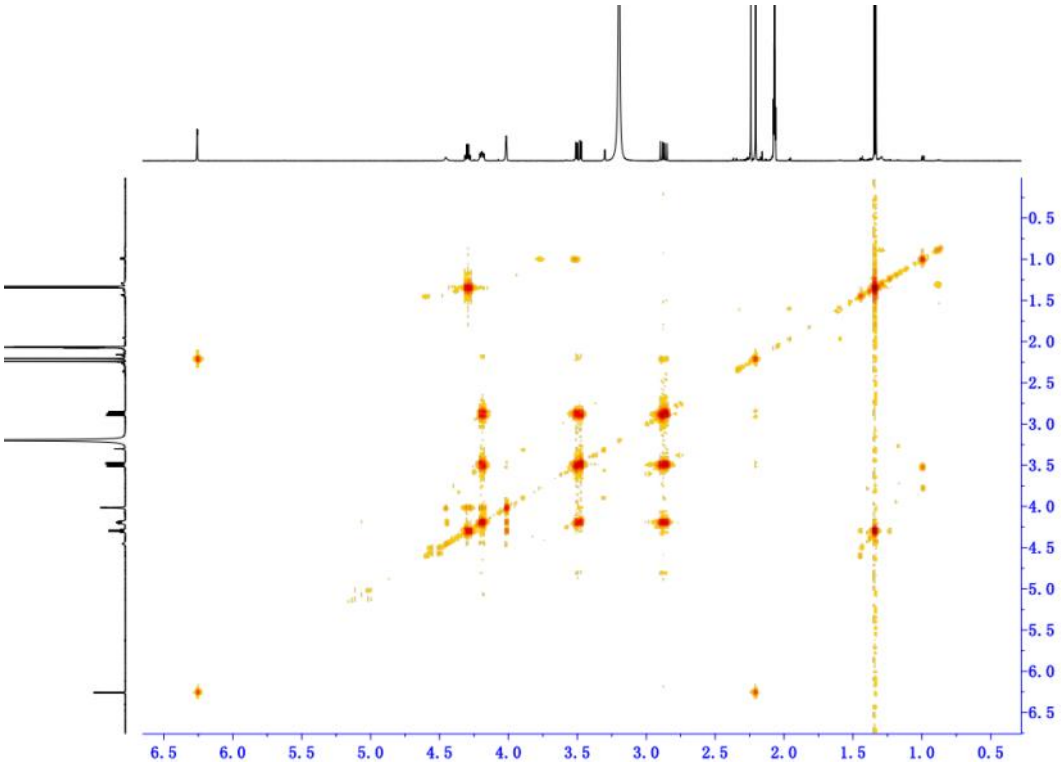

Fig. S39. ^{1}H-^{1}H COSY spectrum of curvamine C (**10**).

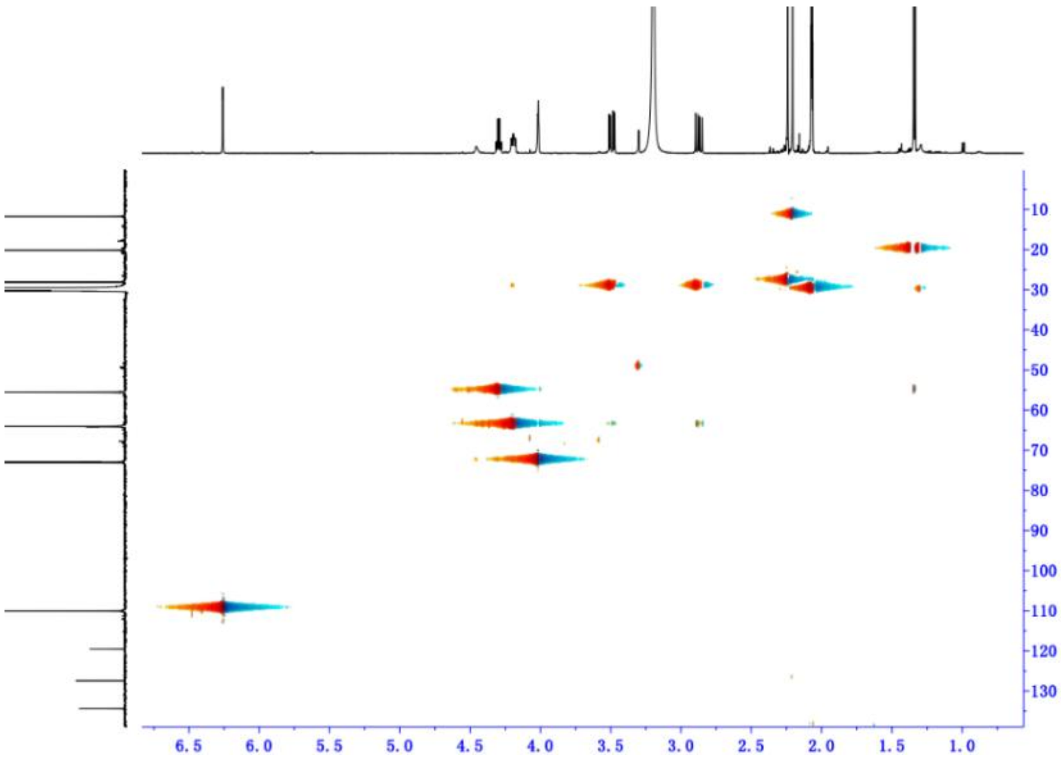

Fig. S40. HSQC spectrum of curvamine C (**10**).

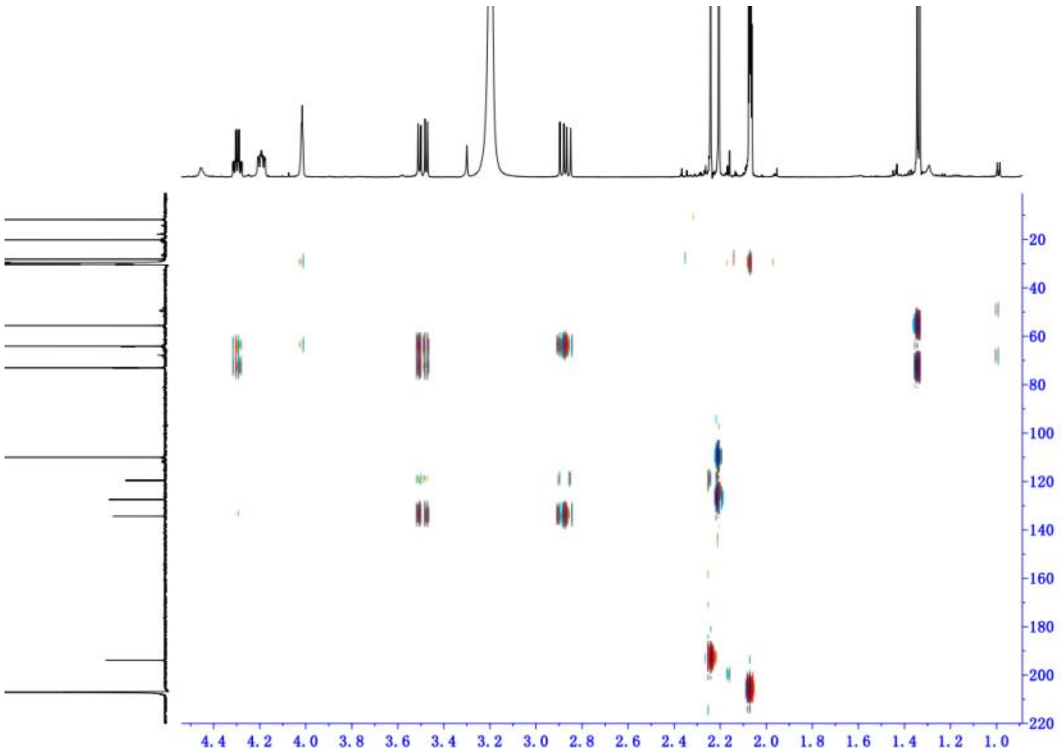

Fig. S41. HMBC spectrum of curvamine C (**10**).

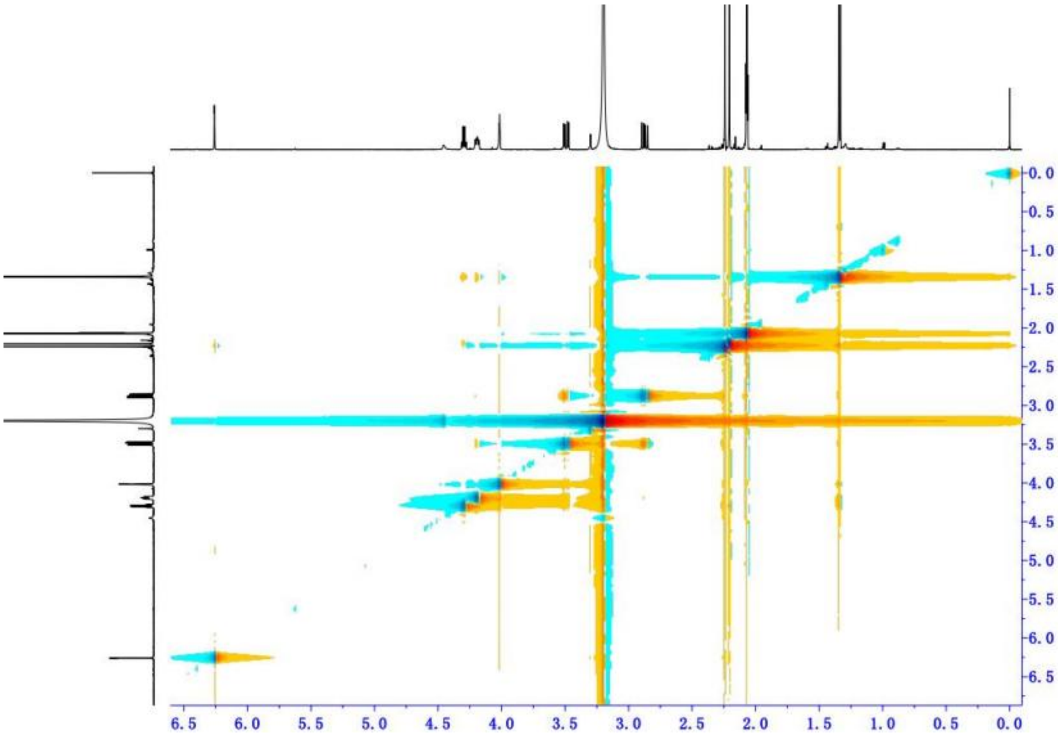

Fig. S42. NOESY spectrum of curvamine C (**10**).

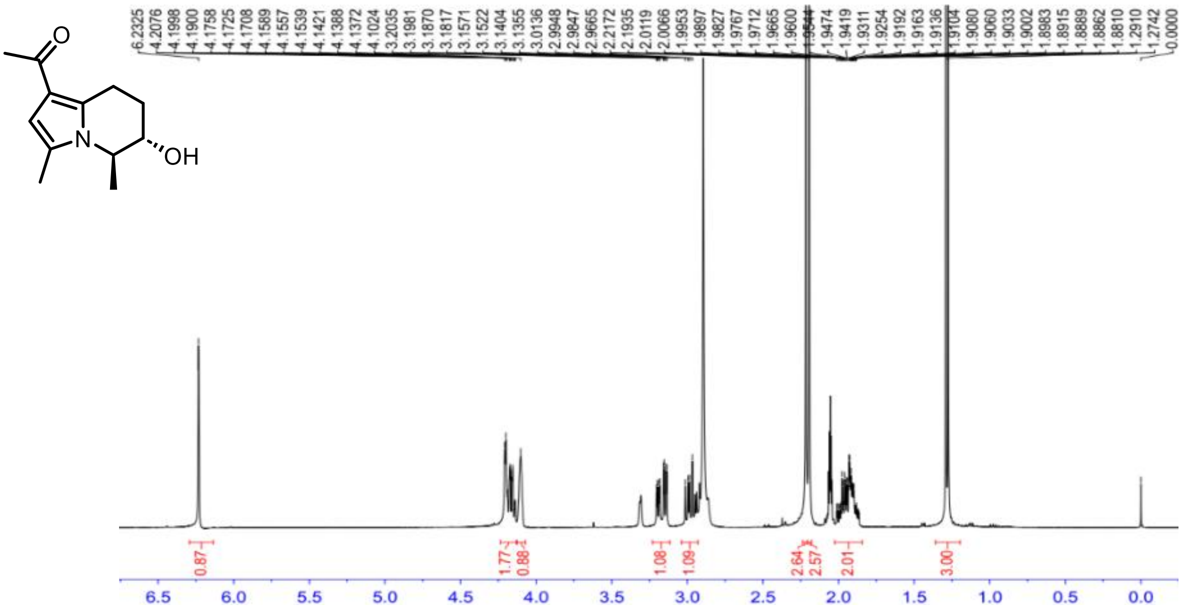

Fig. S43. ^{1}H NMR spectrum of curvamine D (**11**) (400 MHz).

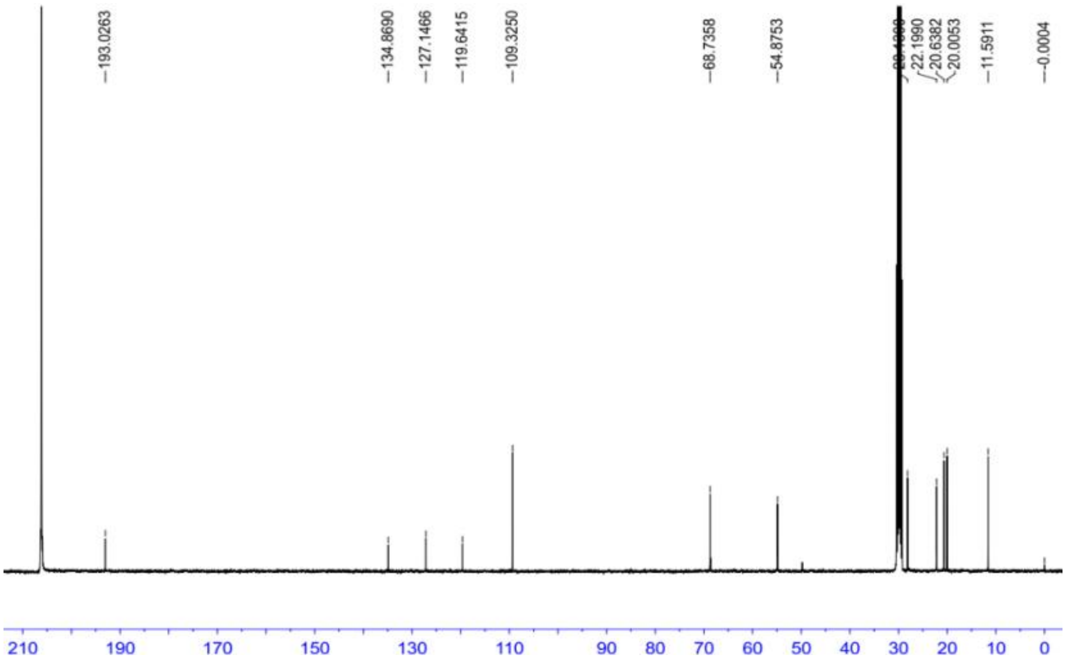

Fig. S44. ^{13}C NMR spectrum of curvamine D (**11**) (100 MHz).

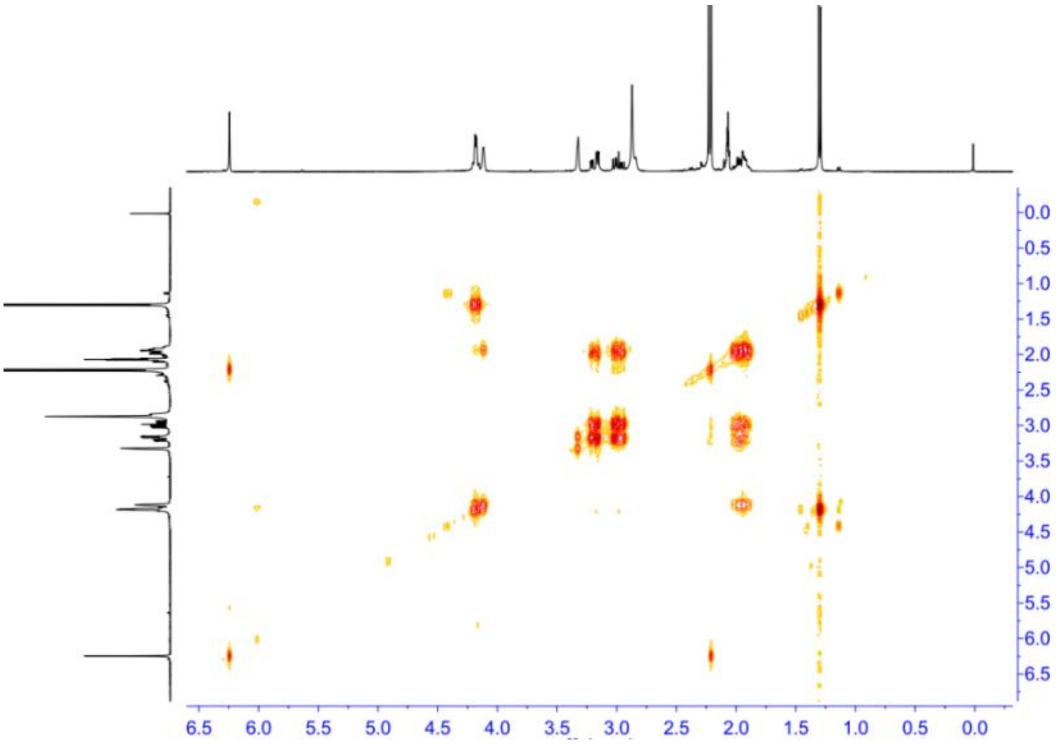

Fig. S45. ^{1}H-^{1}H COSY spectrum of curvamine D (**11**).

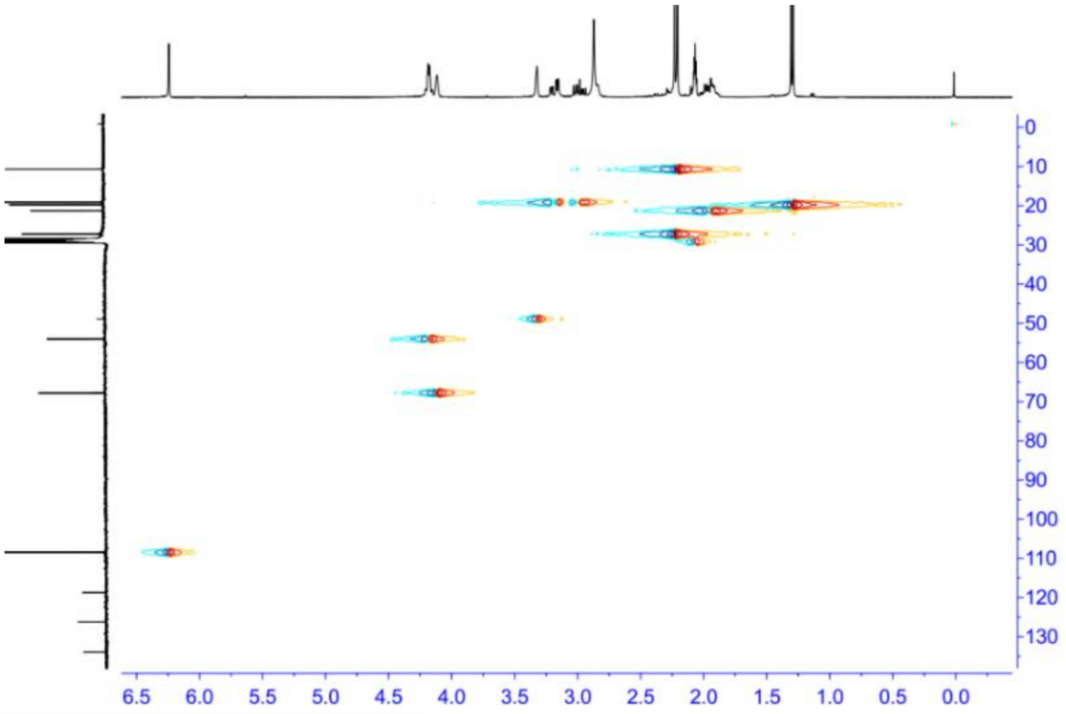

Fig. S46. HSQC spectrum of curvamine D (**11**).

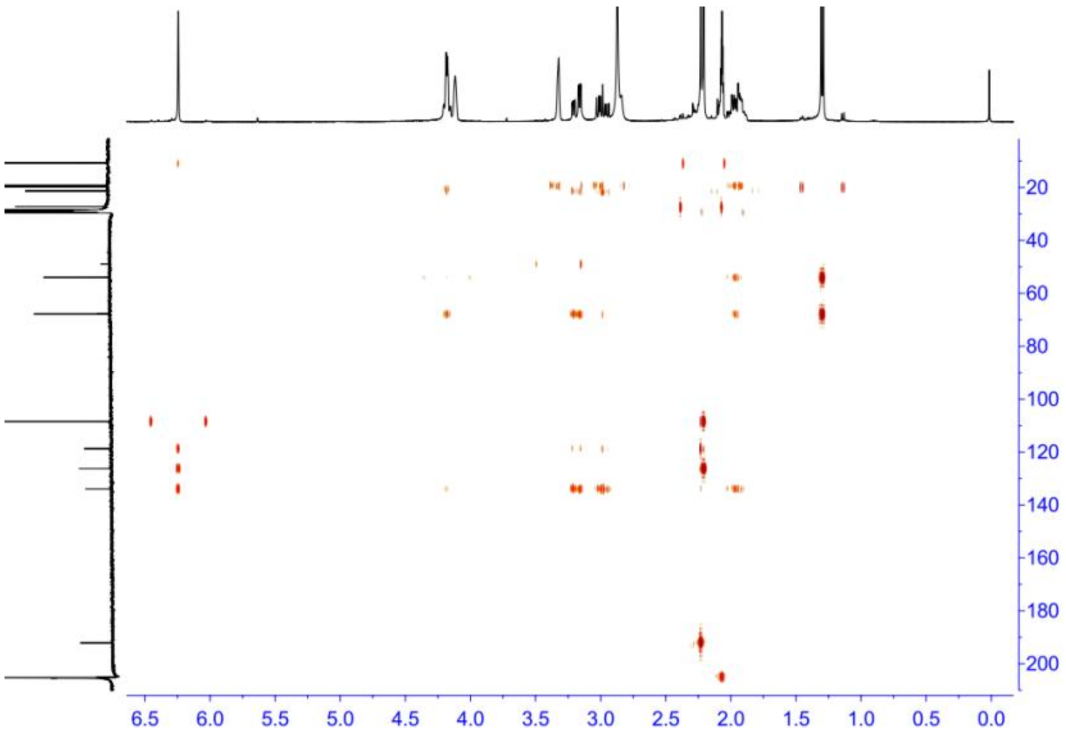

Fig. S47. HMBC spectrum of curvamine D (**11**).

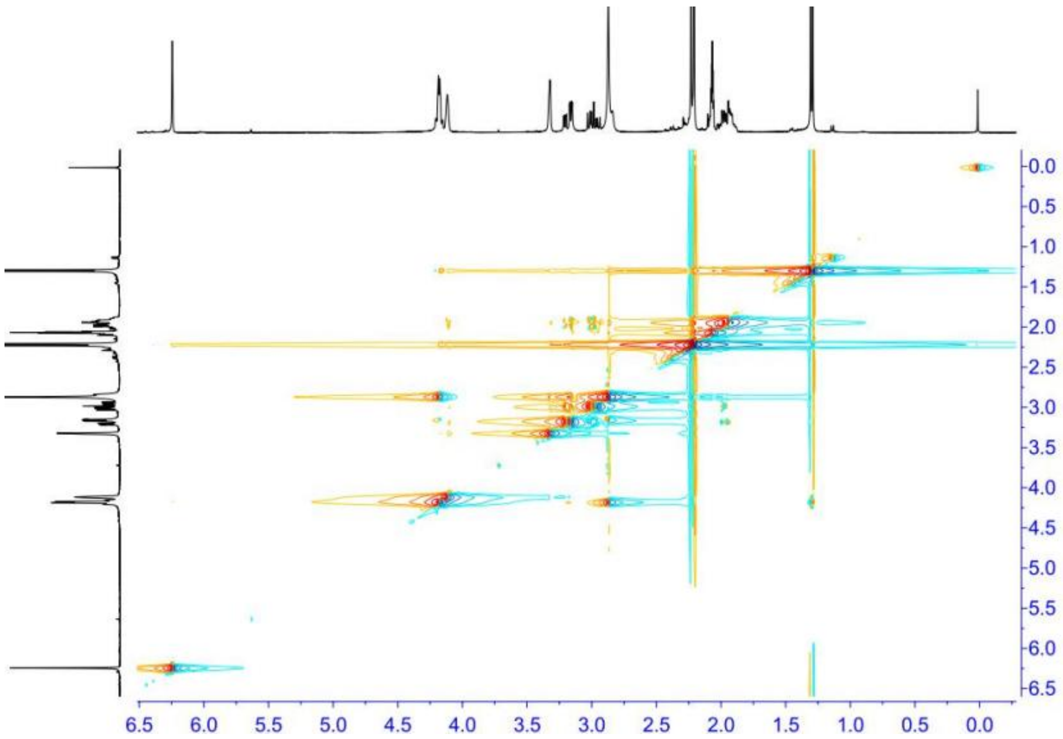

Fig. S48. NOESY spectrum of curvamine D (**11**).

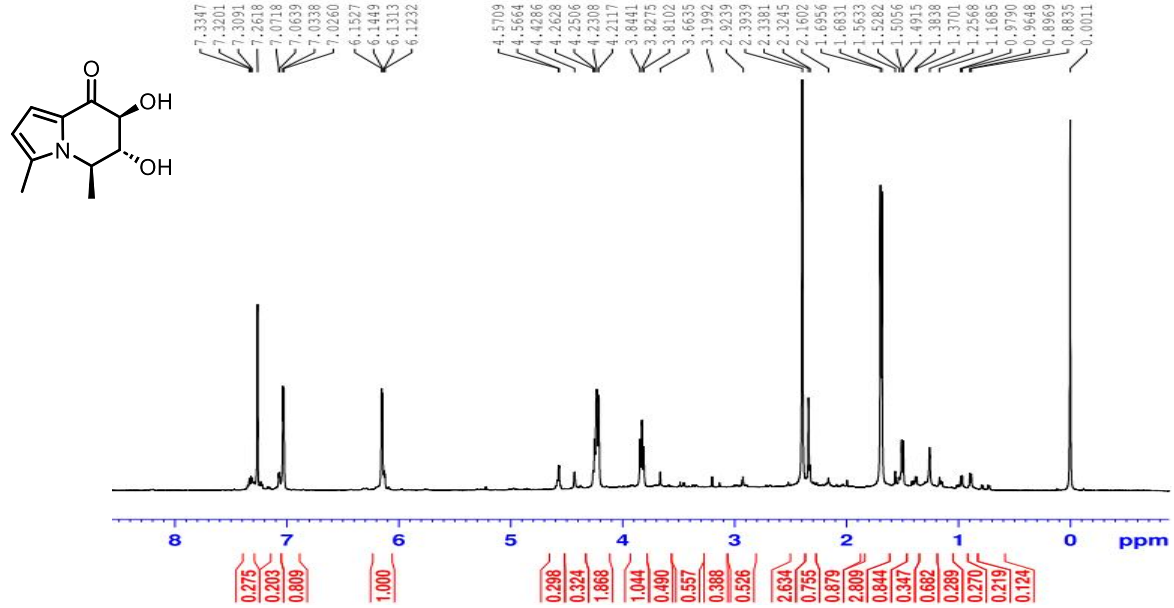

Fig. S49. ^{1}H NMR spectrum of curvamine E (**12**) (500 MHz, $CDCl_3$).

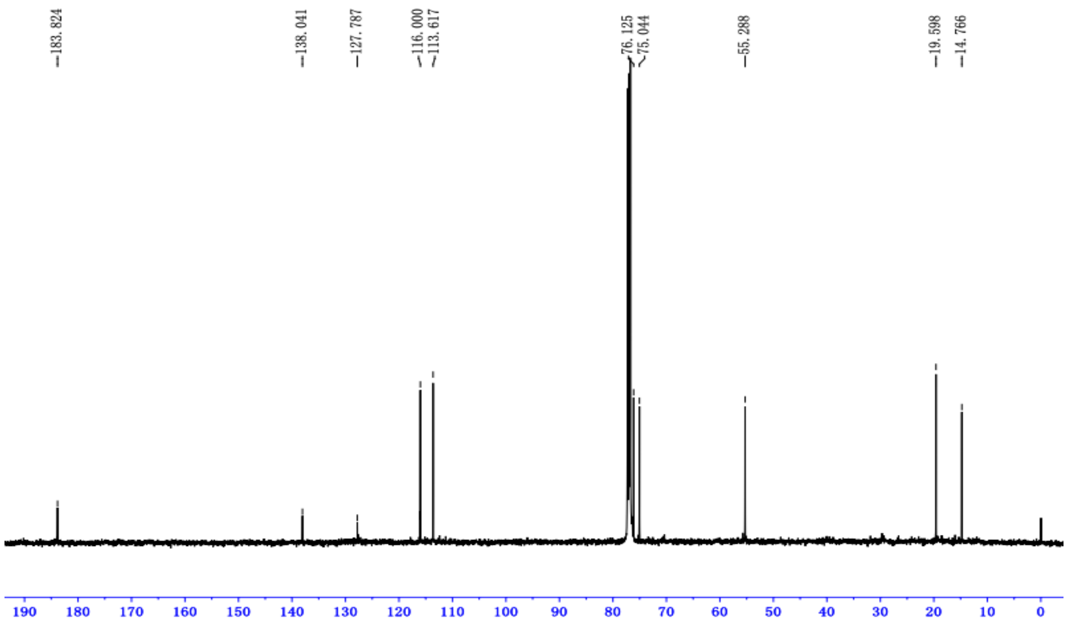

Fig. S50. ^{13}C NMR spectrum of curvamine E (**12**) (125 MHz, $CDCl_3$).

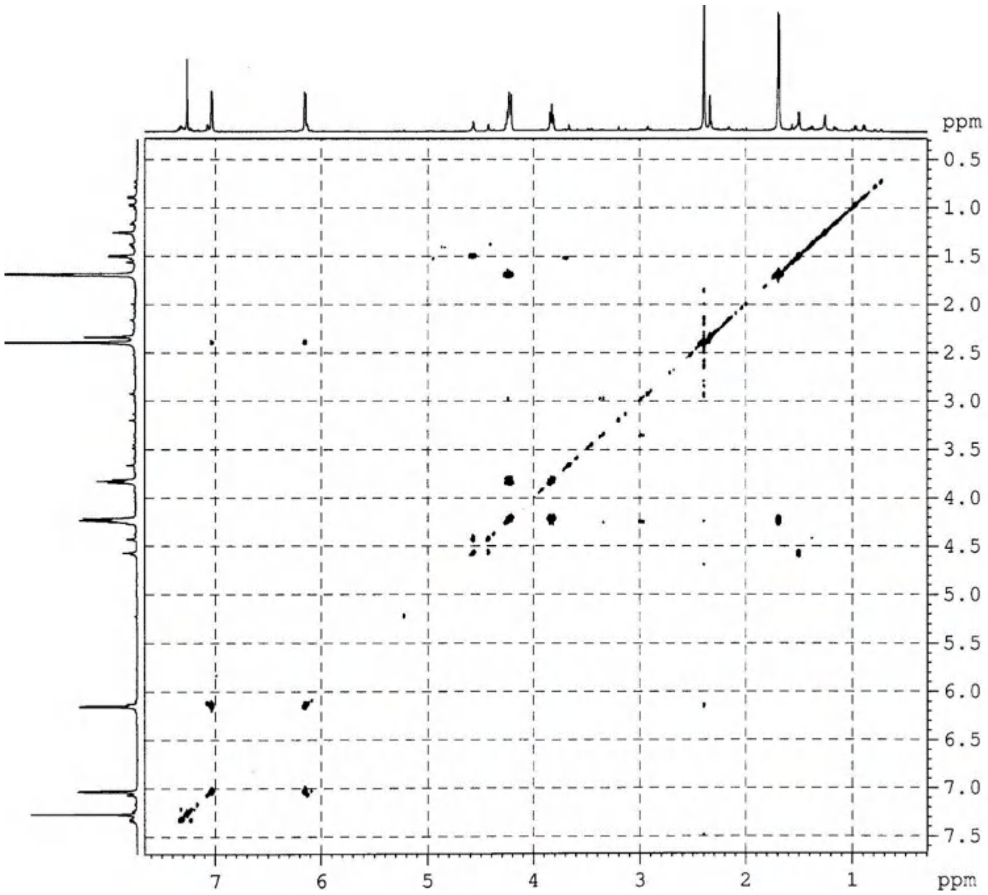

Fig. S51. ^{1}H-^{1}H COSY spectrum of curvamine E (**12**) in $CDCl_3$.

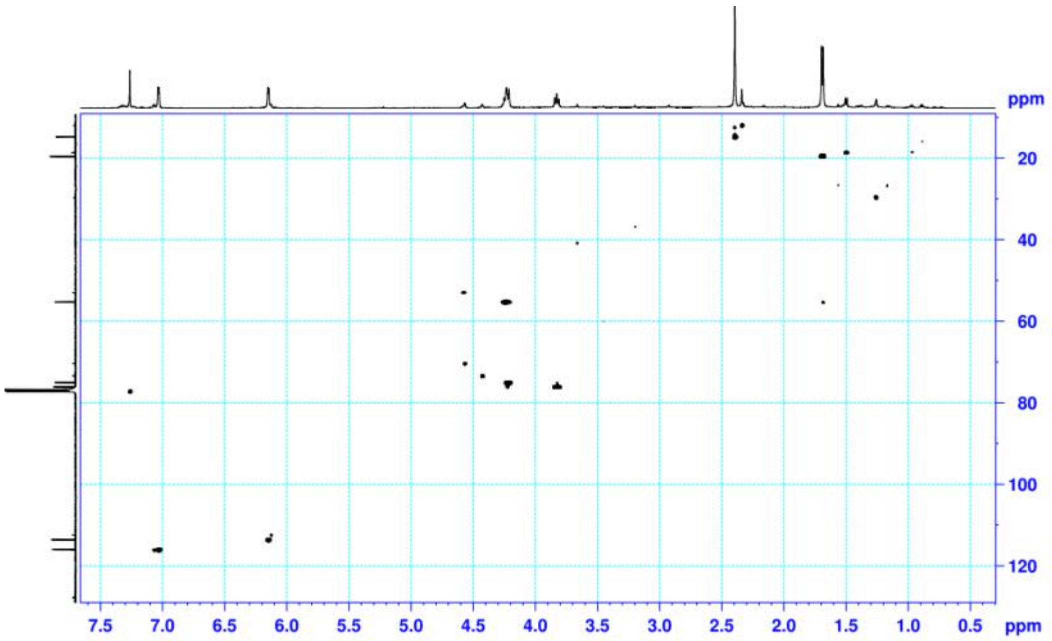

Fig. S52. HSQC spectrum of curvamine E (**12**) in $CDCl_3$.

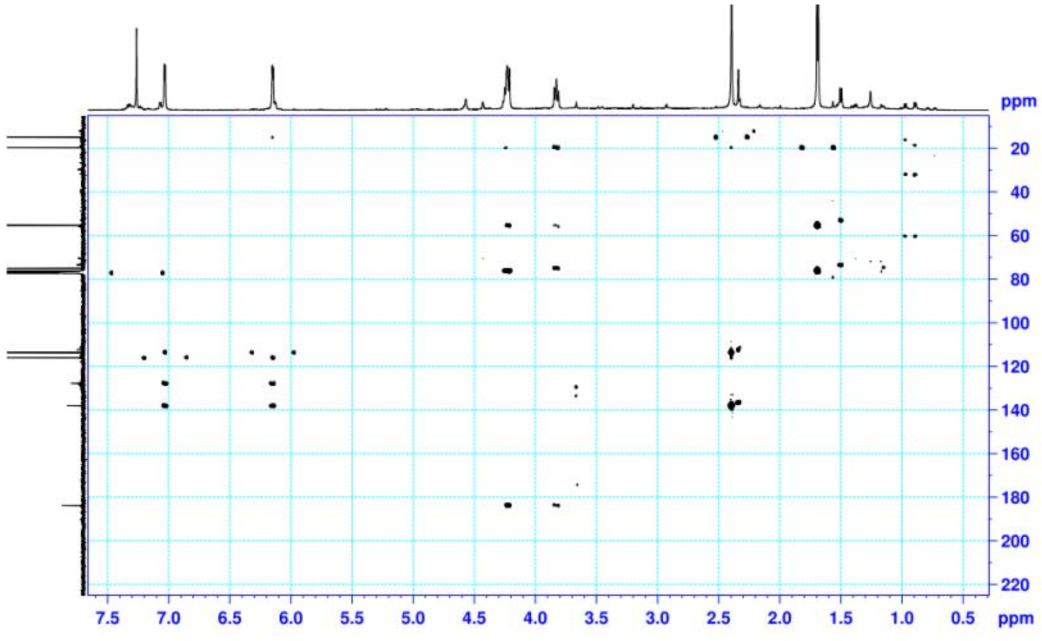

Fig. S53. HMBC spectrum of curvamine E (**12**) in $CDCl_3$.

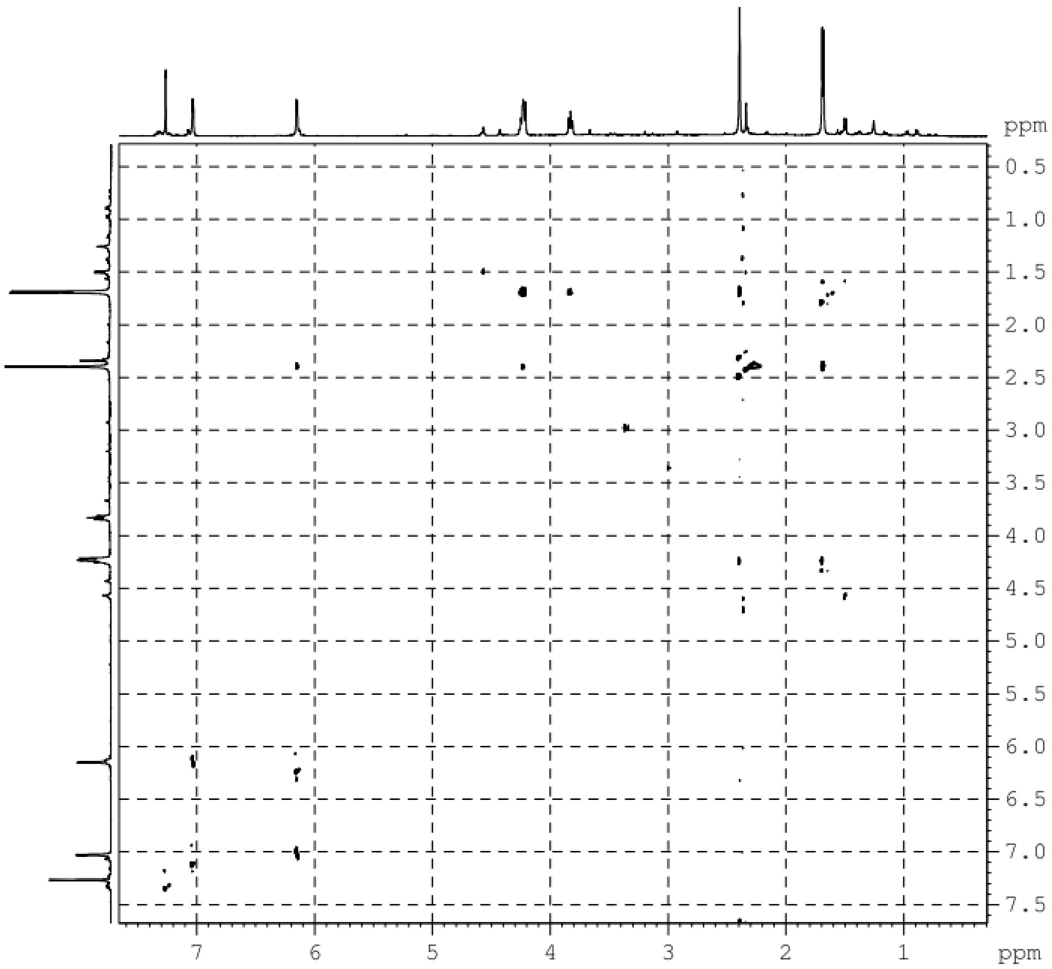

Fig. S54. NOESY spectrum of curvamine E (**12**) in $CDCl_3$.

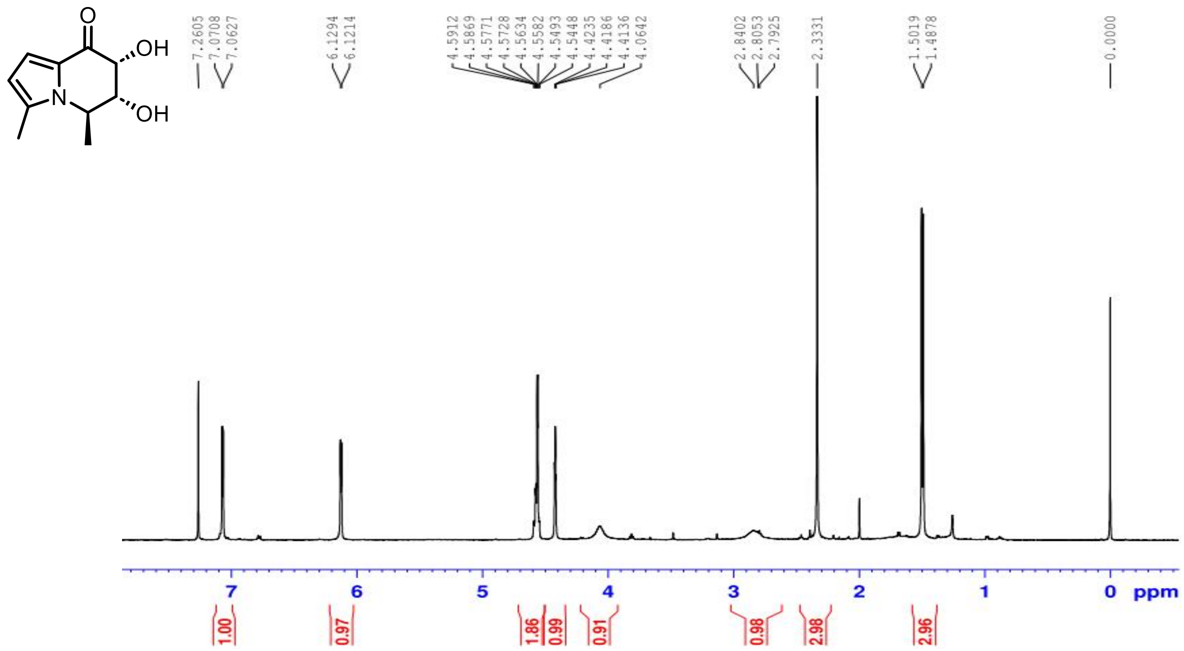

Fig. S55. ^{1}H NMR spectrum of curvamine F (**13**) (500 MHz, $CDCl_3$).

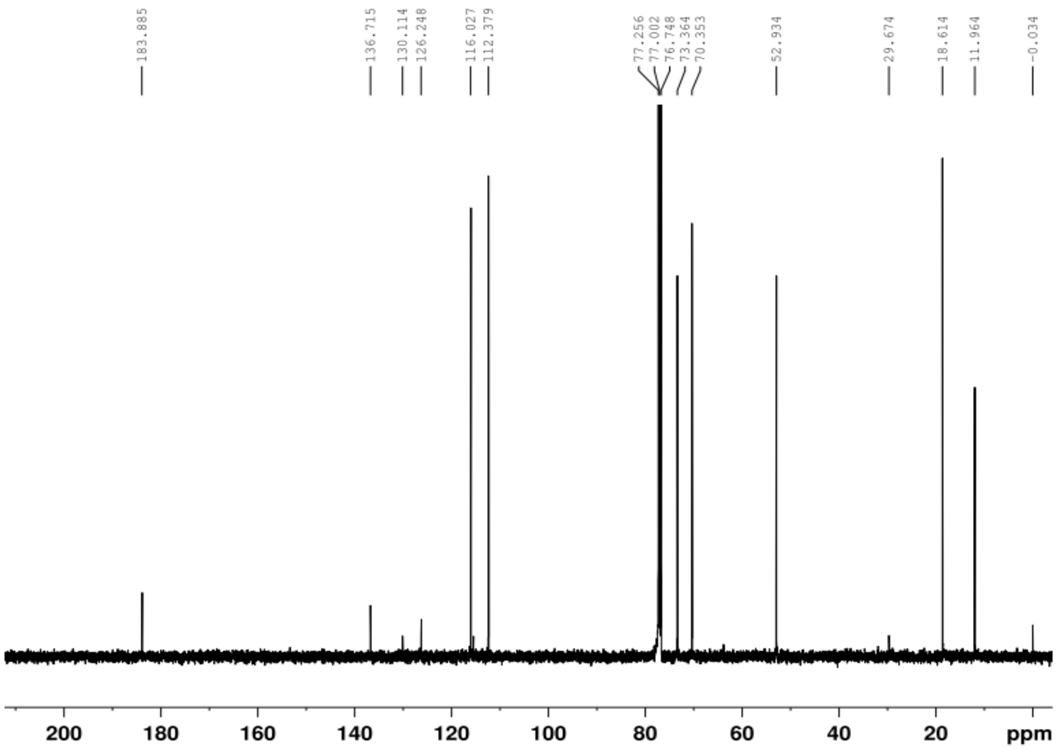

Fig. S56. ^{13}C NMR spectrum of curvamine F (**13**) (125 MHz, $CDCl_3$).

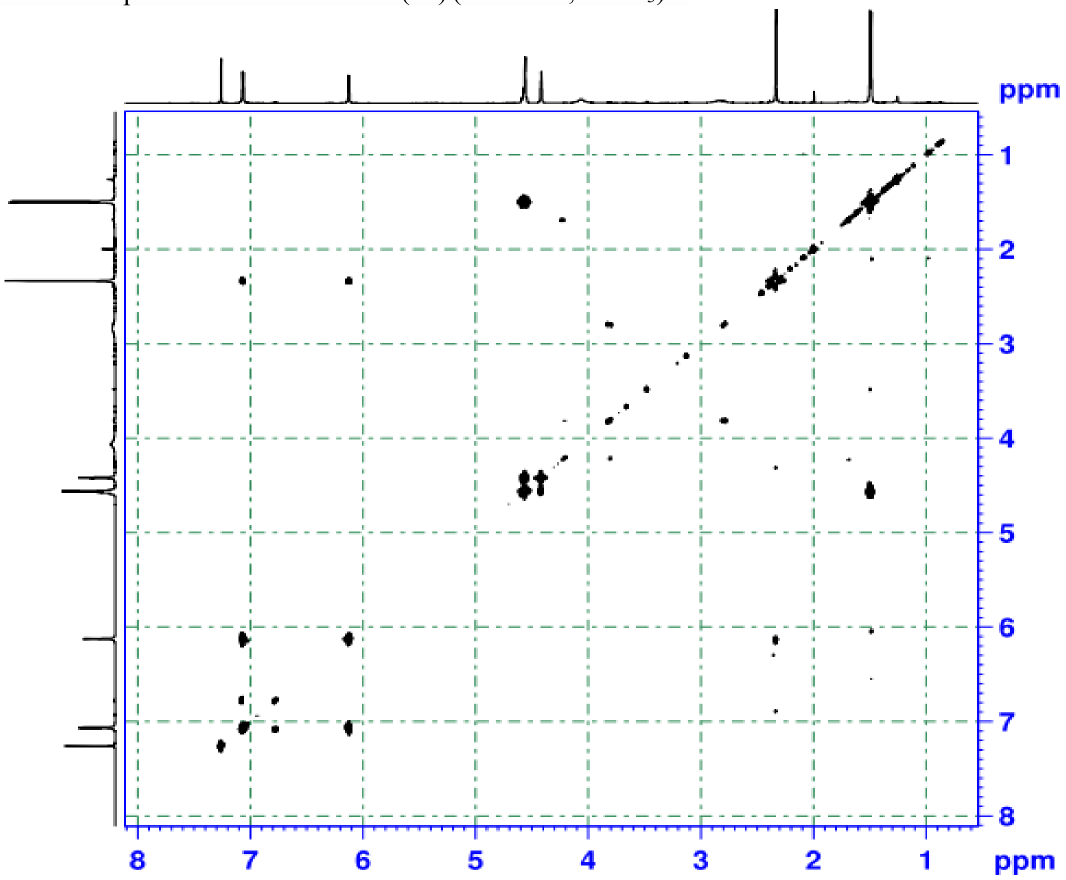

Fig. S57. ^{1}H-^{1}H COSY spectrum of curvamine F (**13**) in $CDCl_3$.

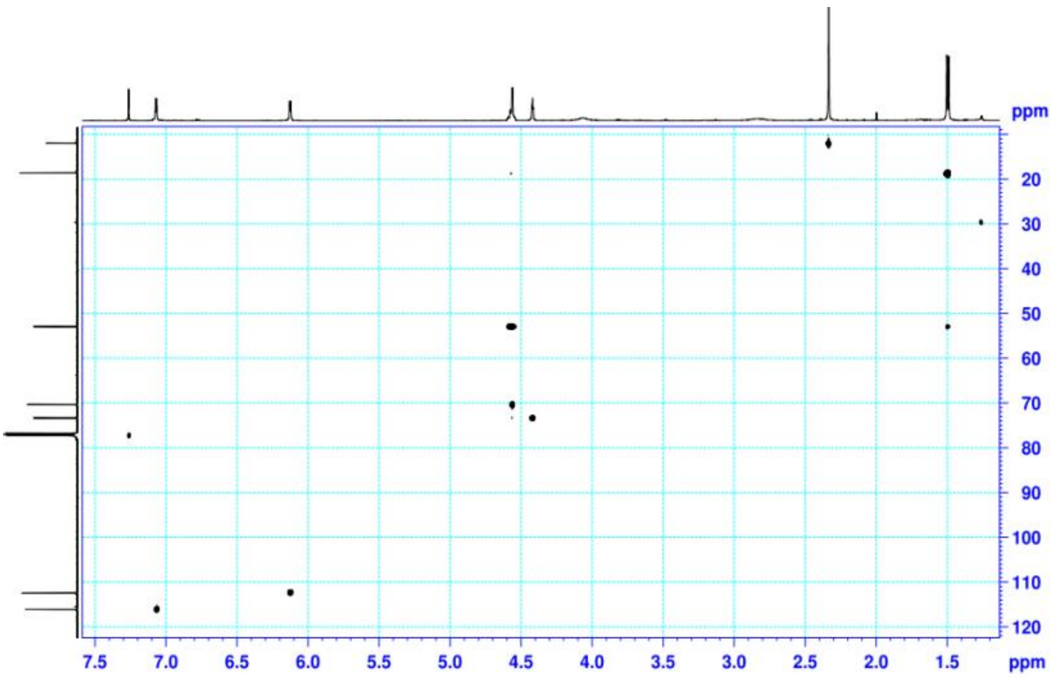

Fig. S58. HSQC spectrum of curvamine F (**13**) in $CDCl_3$.

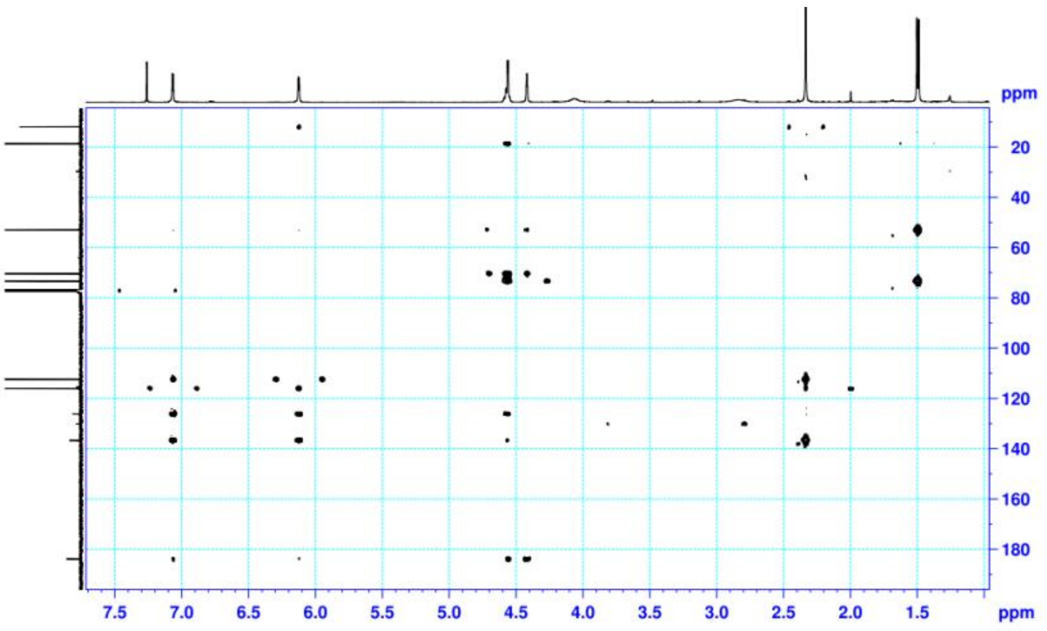

Fig. S59. HMBC spectrum of curvamine F (**13**) in $CDCl_3$.

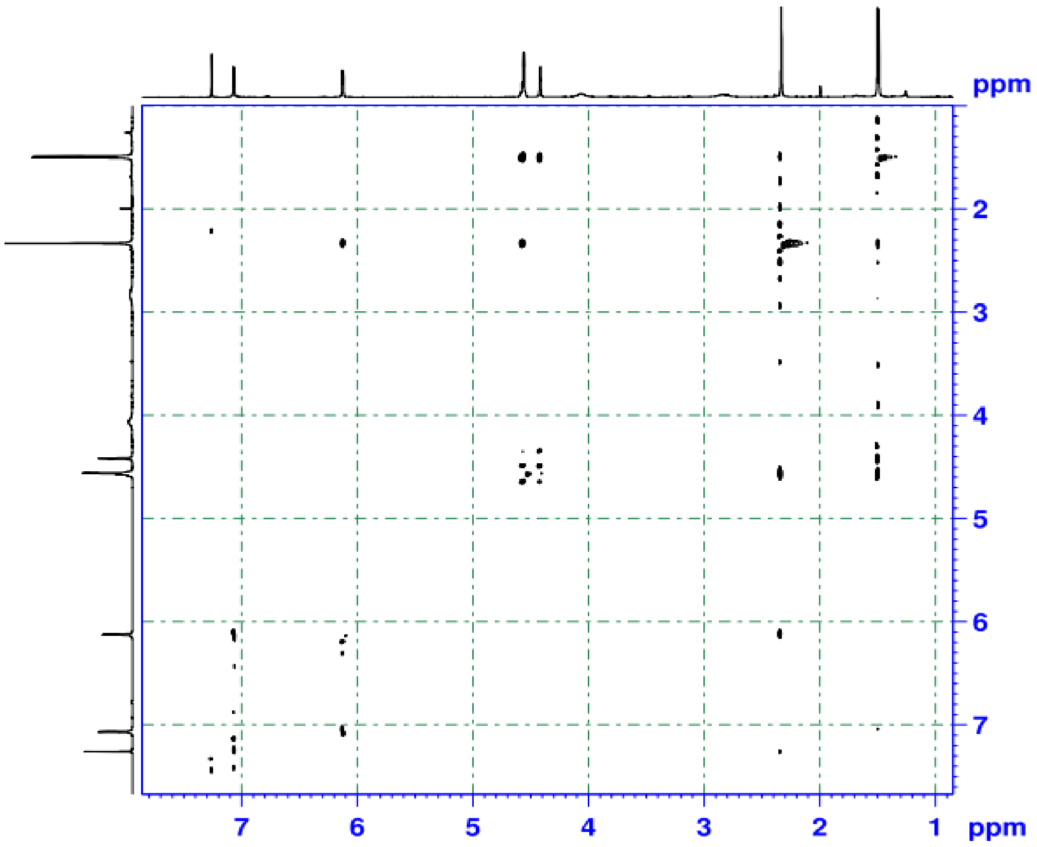

Fig. S60. NOESY spectrum of curvamine F (**13**) in $CDCl_3$.

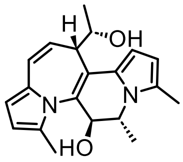

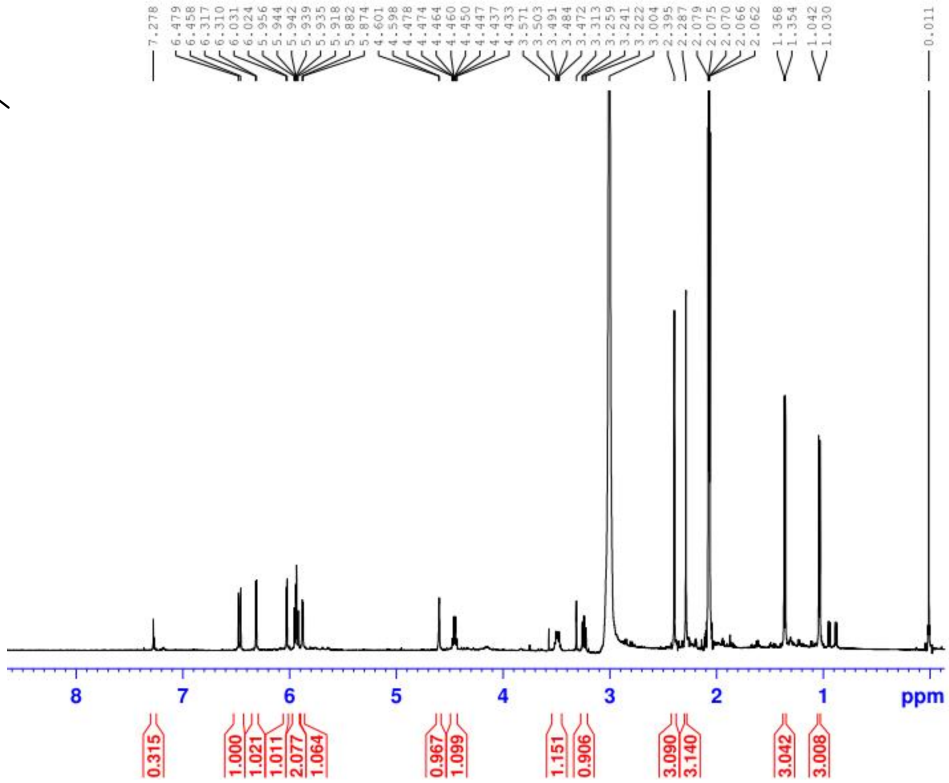

Fig. S61. ^{1}H NMR spectrum of curvamine G (**14**) (500 MHz).

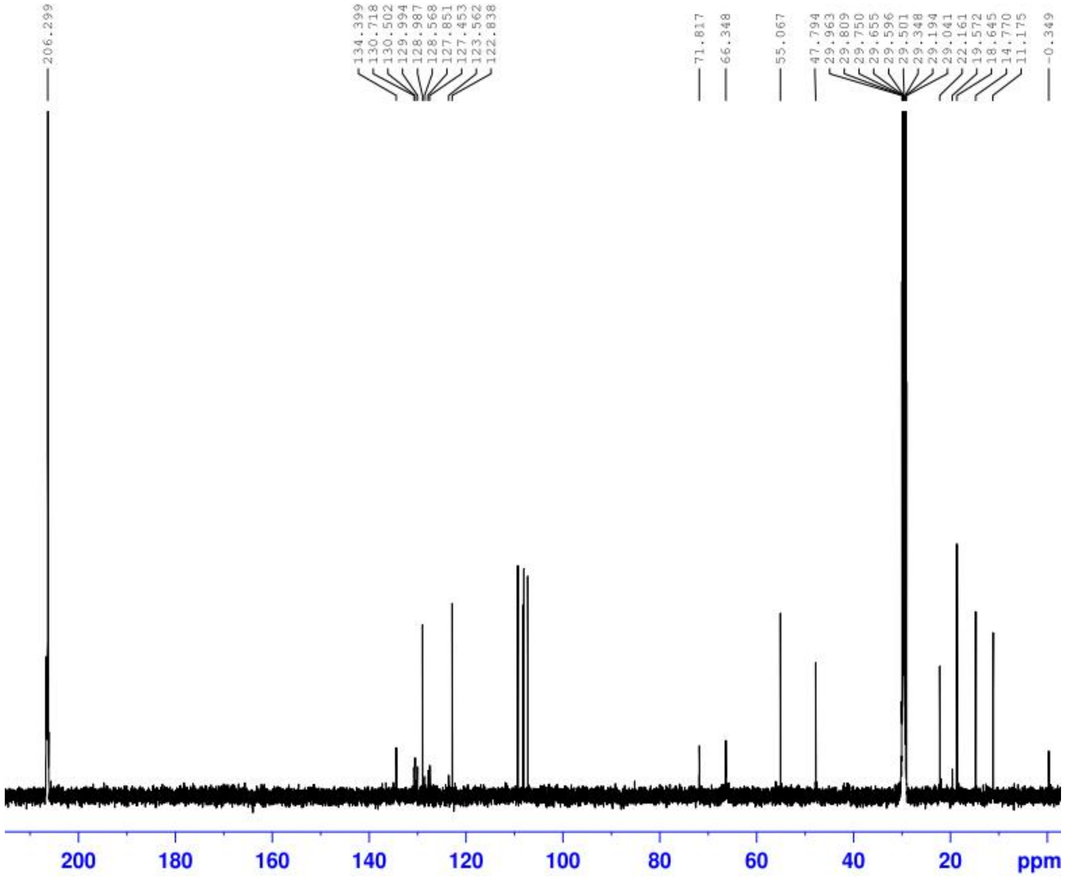

Fig. S62. ^{13}C NMR spectrum of curvamine G (**14**) (125 MHz).

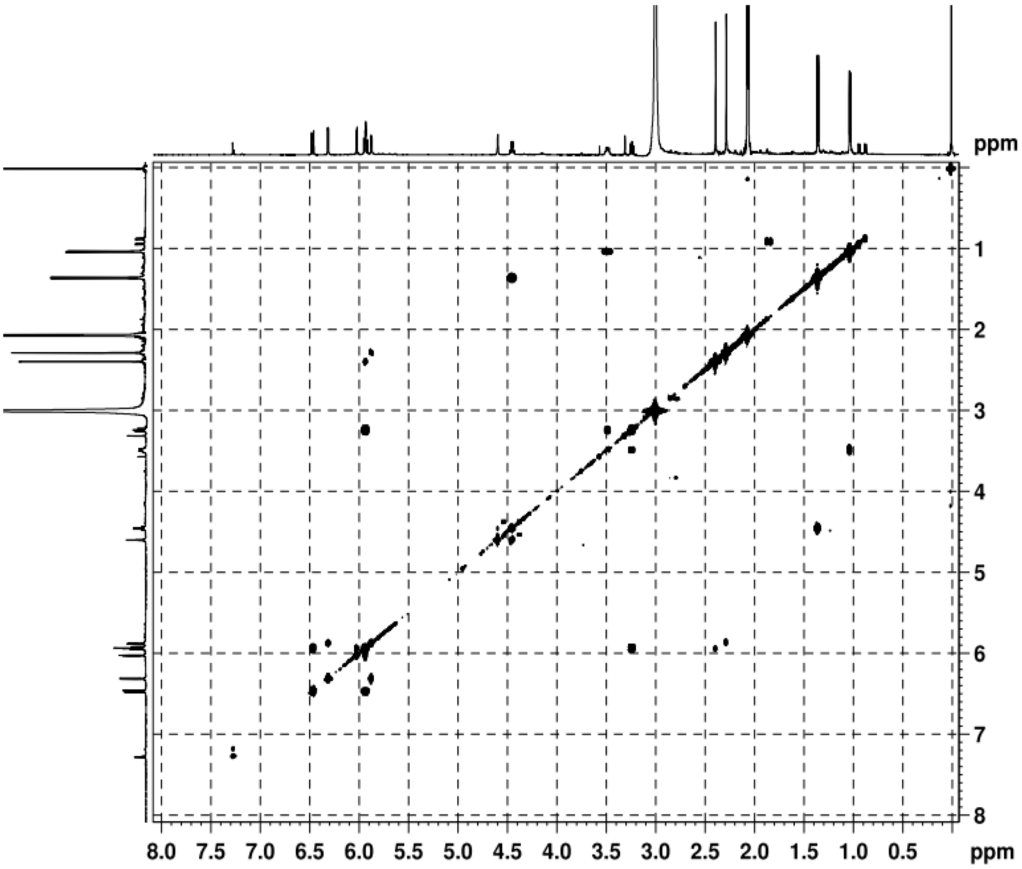

Fig. S63. ^{1}H-^{1}H COSY spectrum of curvamine G (**14**).

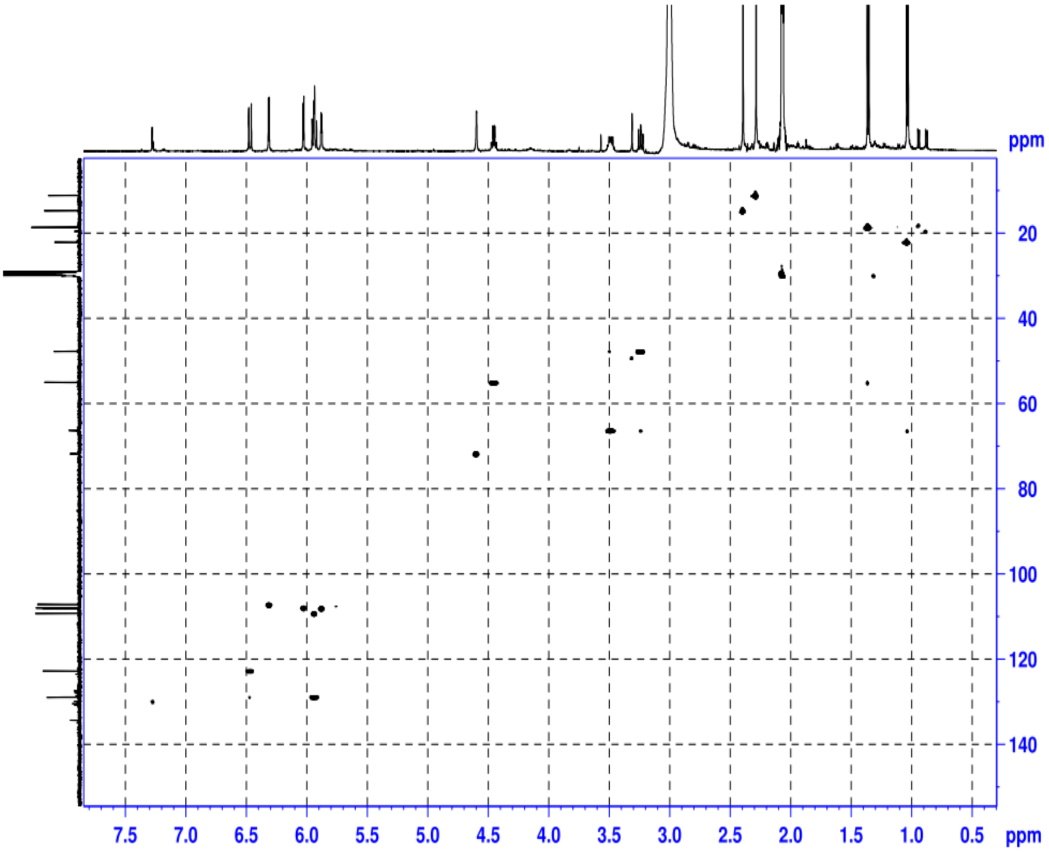

Fig. S64. HSQC spectrum of curvamine G (**14**).

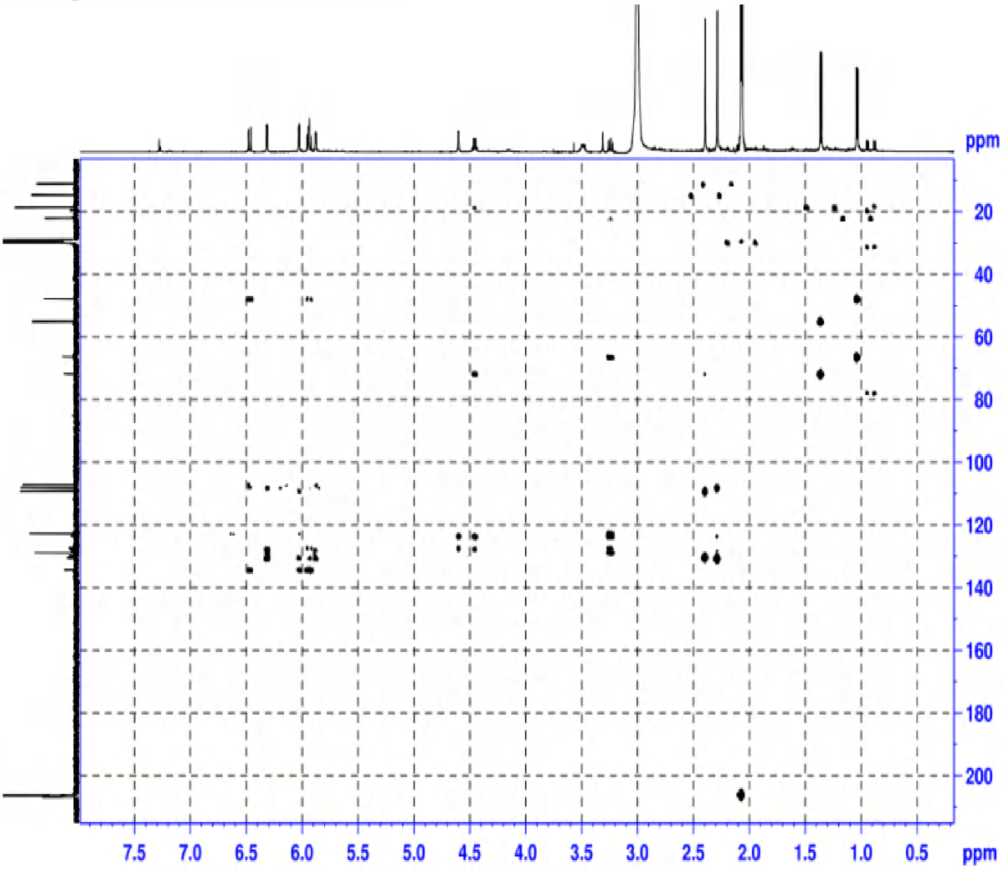

Fig. S65. HMBC spectrum of curvamine G (**14**).

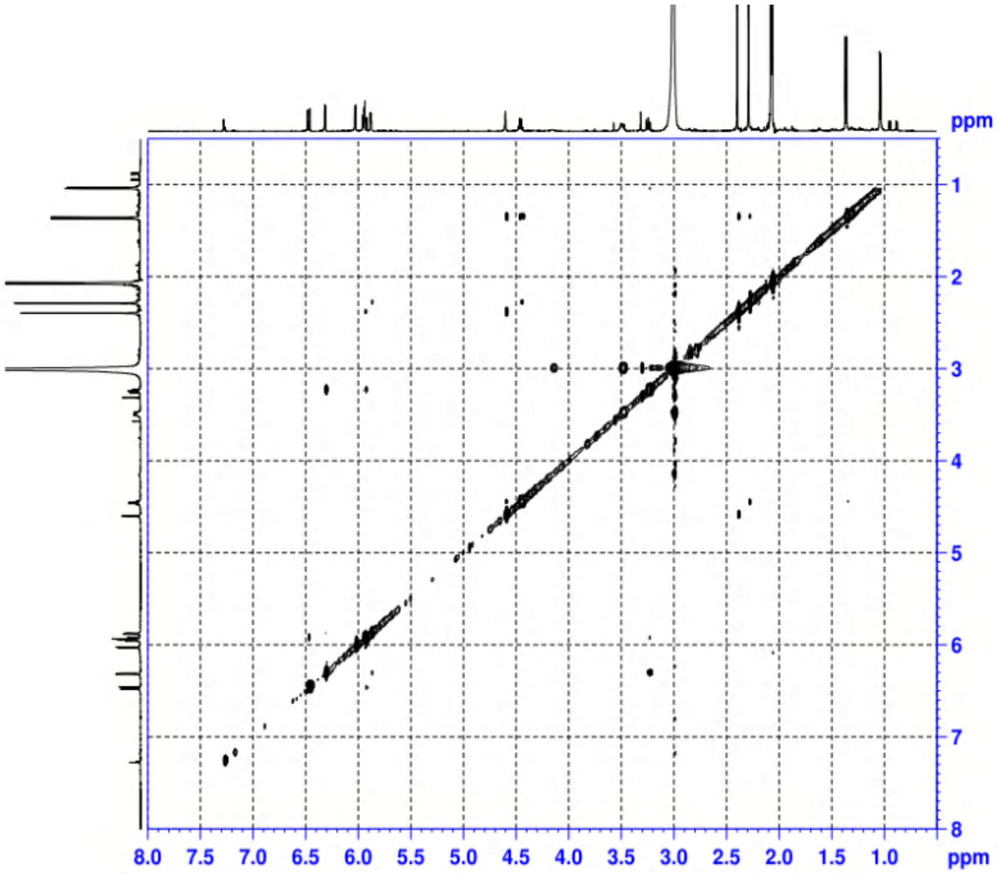

Fig. S66. NOESY spectrum of curvamine G (**14**).

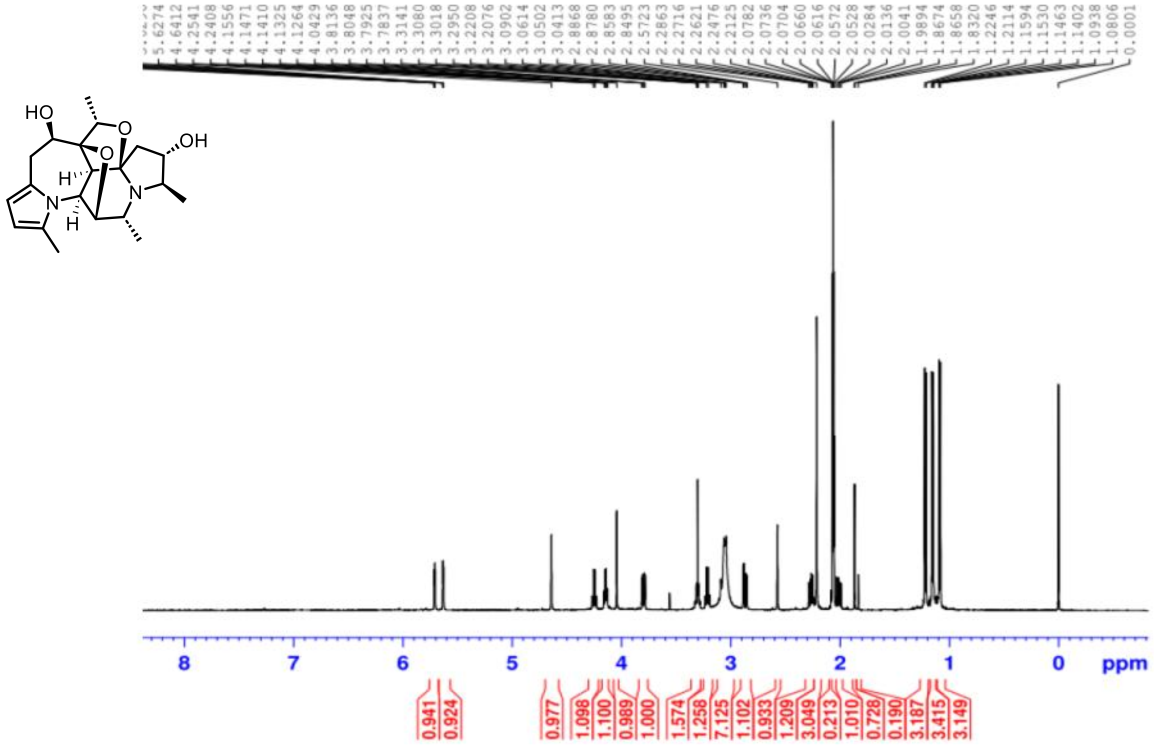

Fig. S67. ^{1}H NMR spectrum of curvamine H (**15**) (500 MHz).

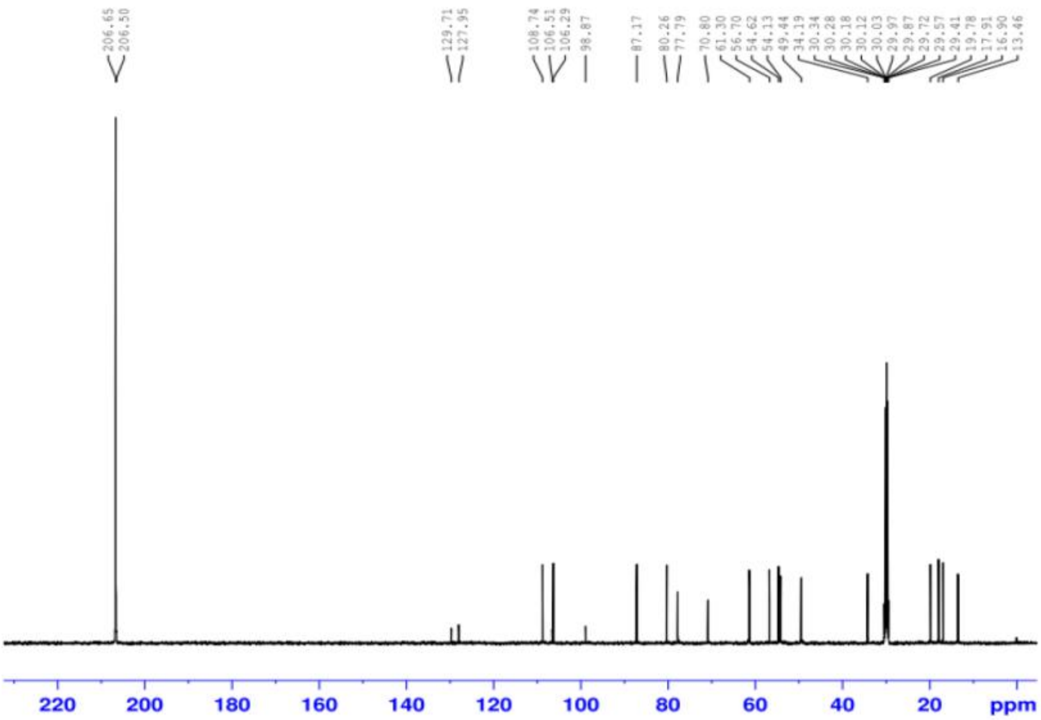

Fig. S68. ^{13}C NMR spectrum of curvamine H (**15**) (125 MHz).

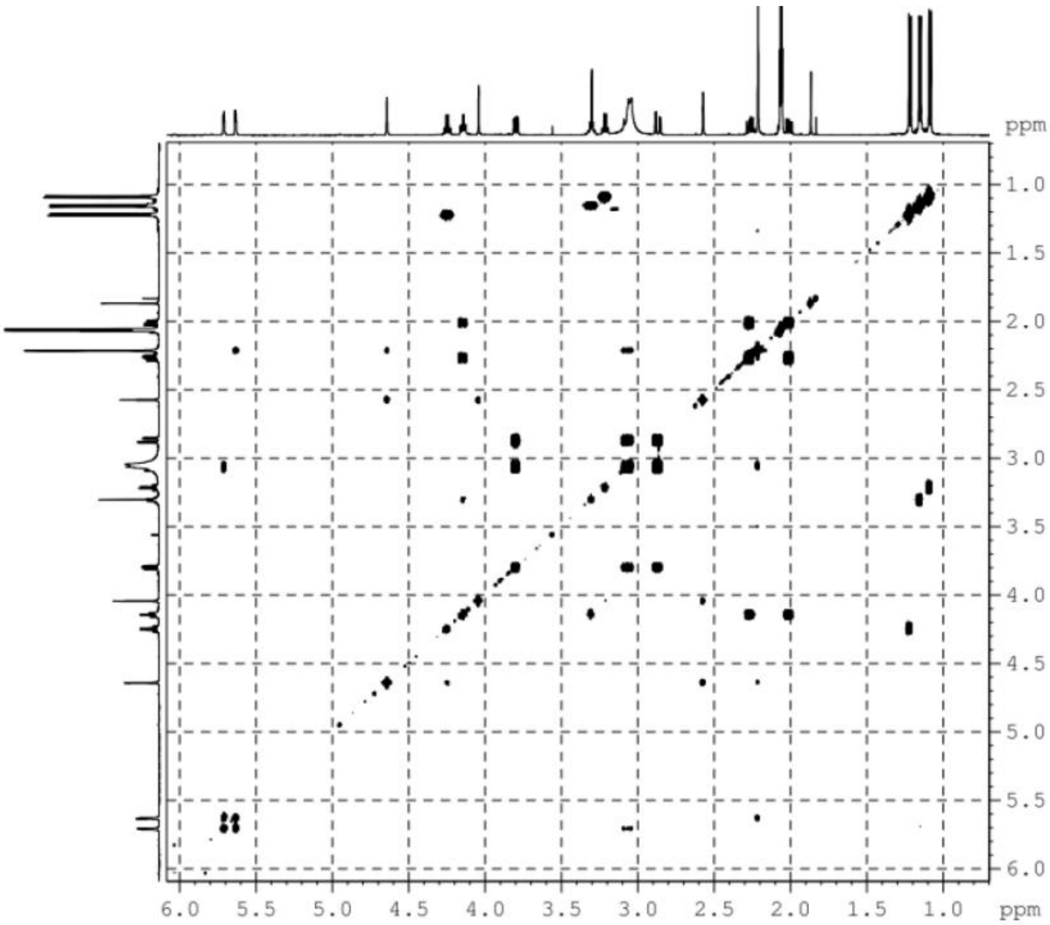

Fig. S69. ^{1}H-^{1}H COSY spectrum of curvamine H (**15**).

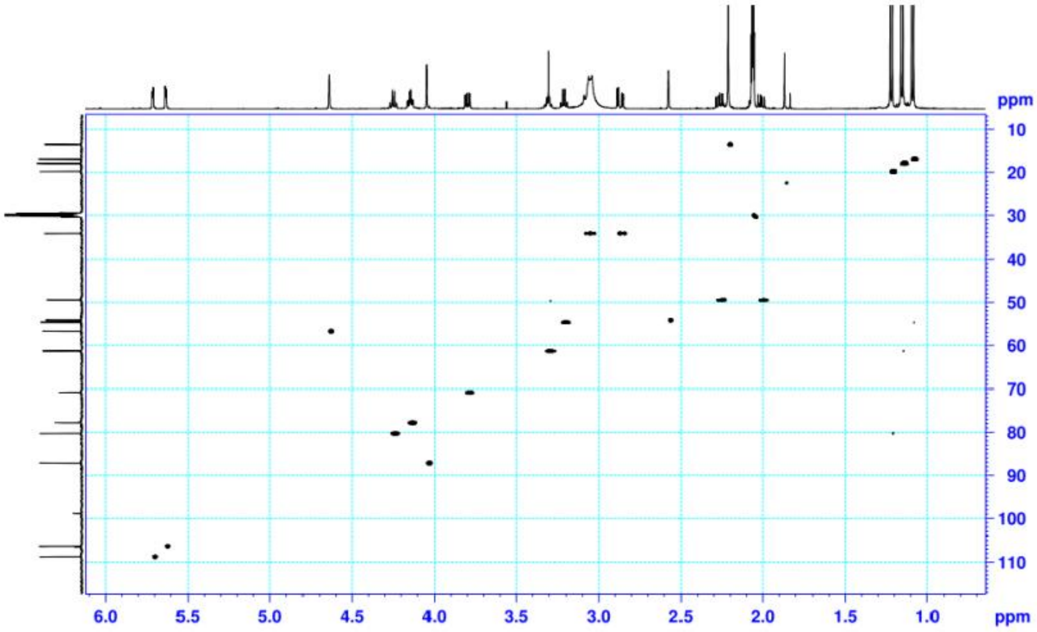

Fig. S70. HSQC spectrum of curvamine H (**15**).

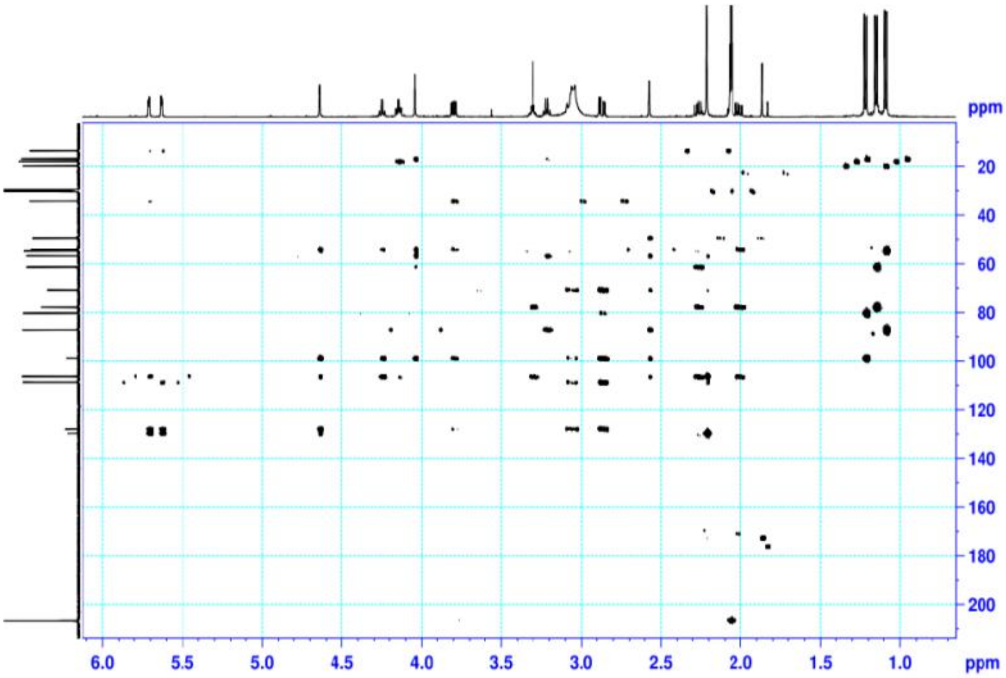

Fig. S71. HMBC spectrum of curvamine H (**15**).

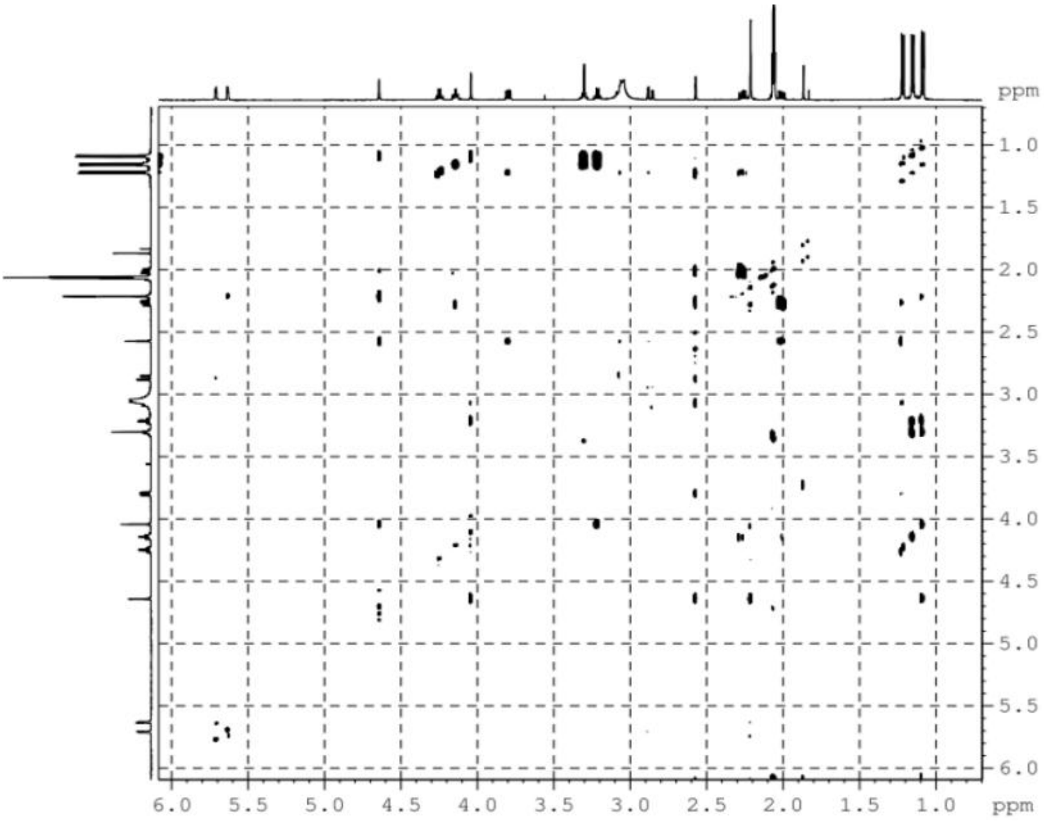

Fig. S72. NOESY spectrum of curvamine H (**15**).

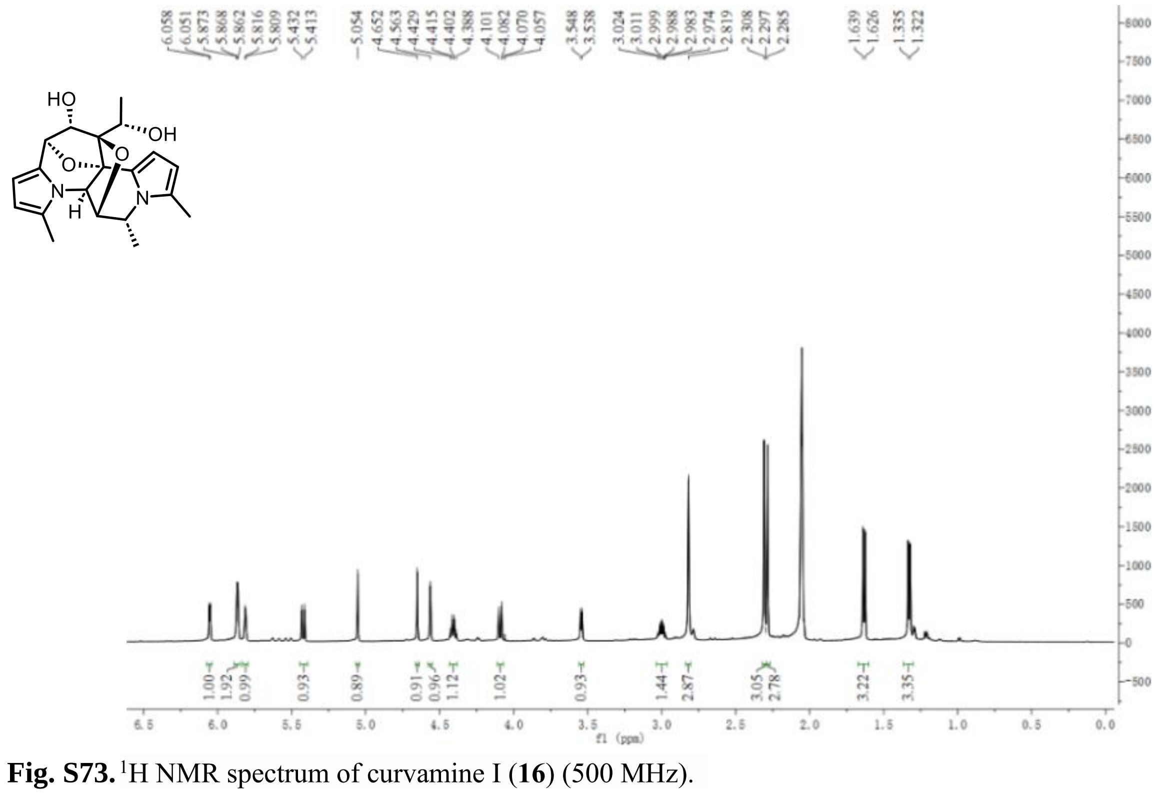

Fig. S73. ^{1}H NMR spectrum of curvamine I (**16**) (500 MHz).

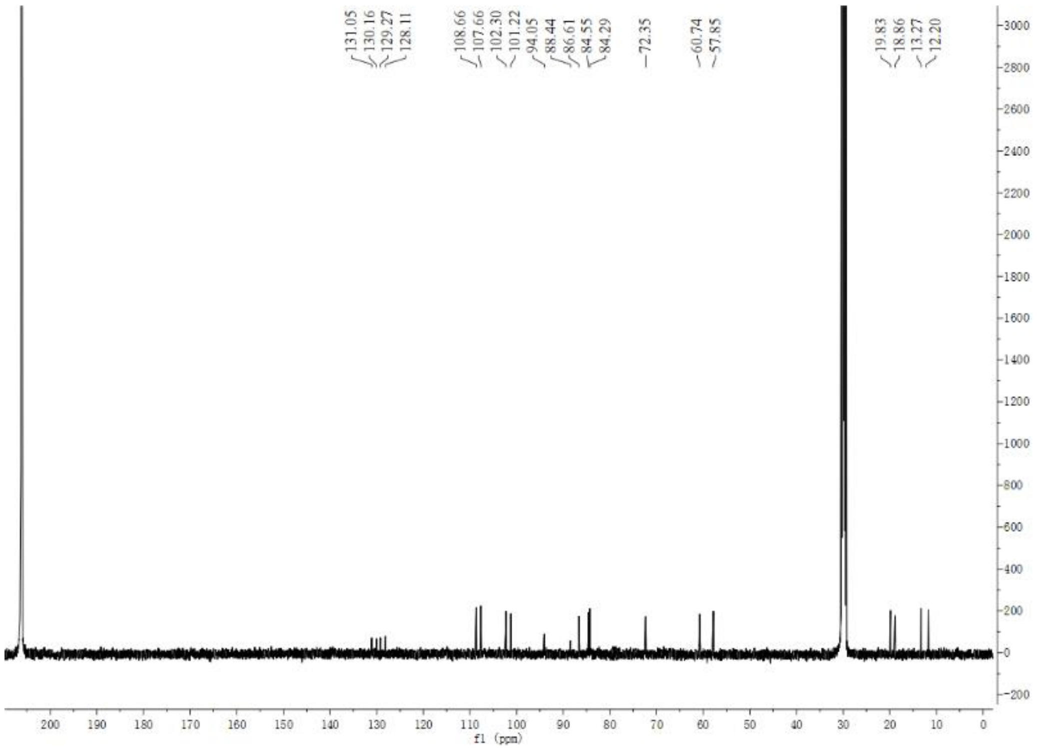

Fig. S74. ^{13}C NMR spectrum of curvamine I (**16**) (125 MHz).

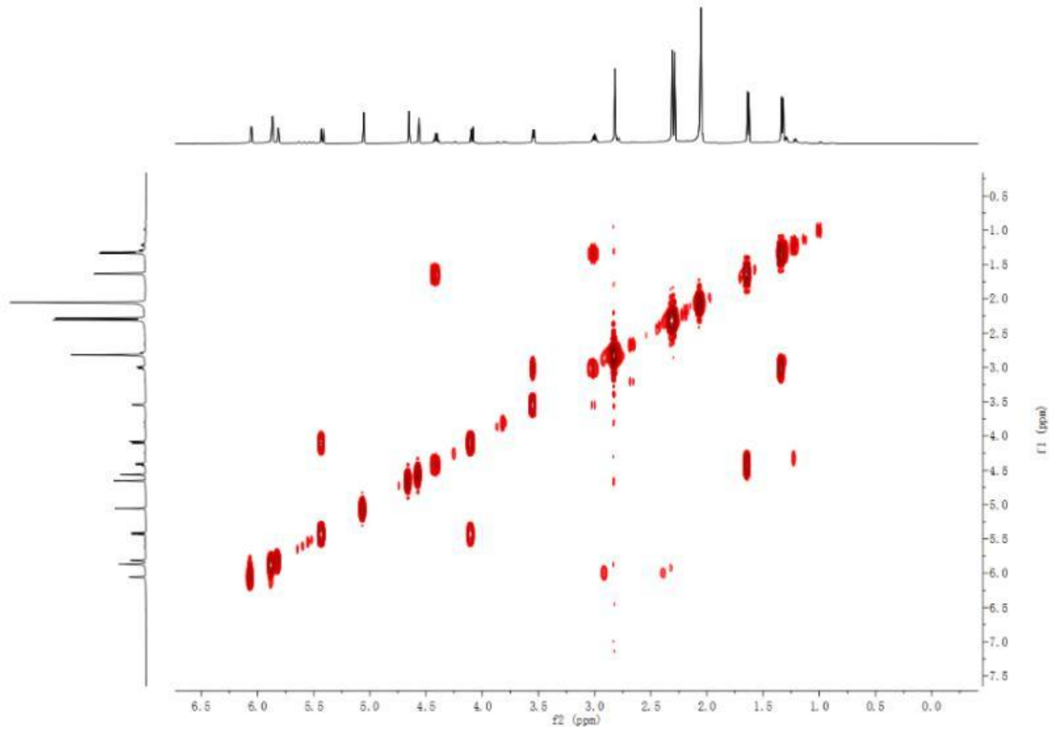

Fig. S75. ^{1}H-^{1}H COSY spectrum of curvamine I (**16**).

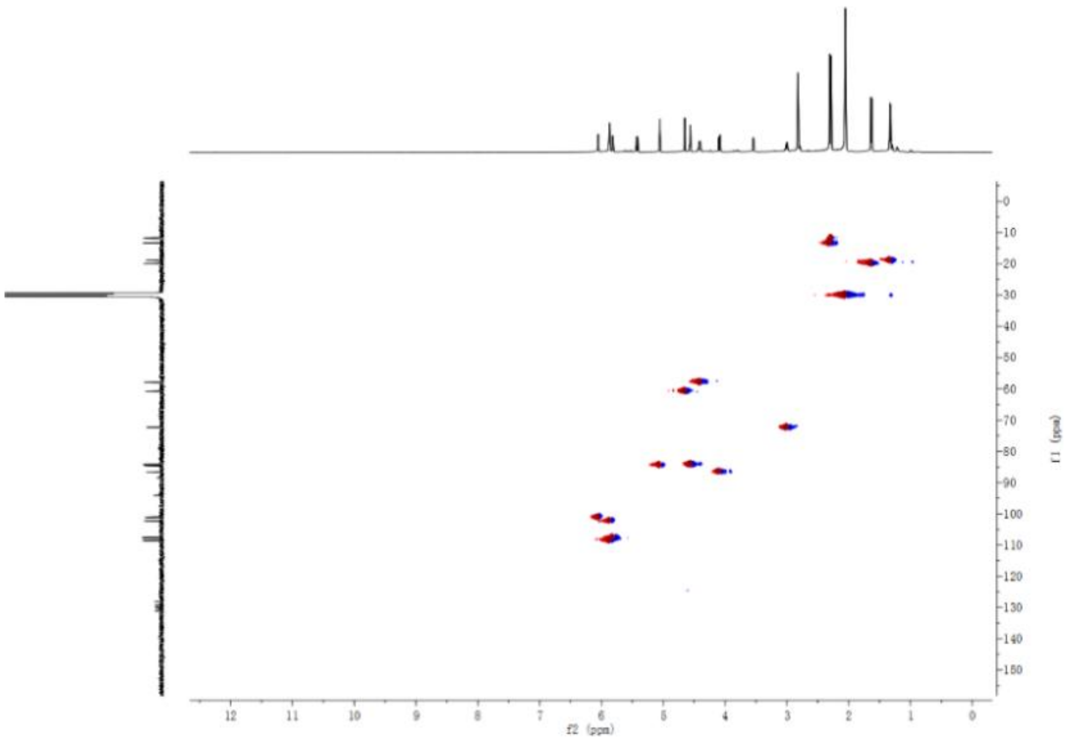

Fig. S76. HSQC spectrum of curvamine I (**16**).

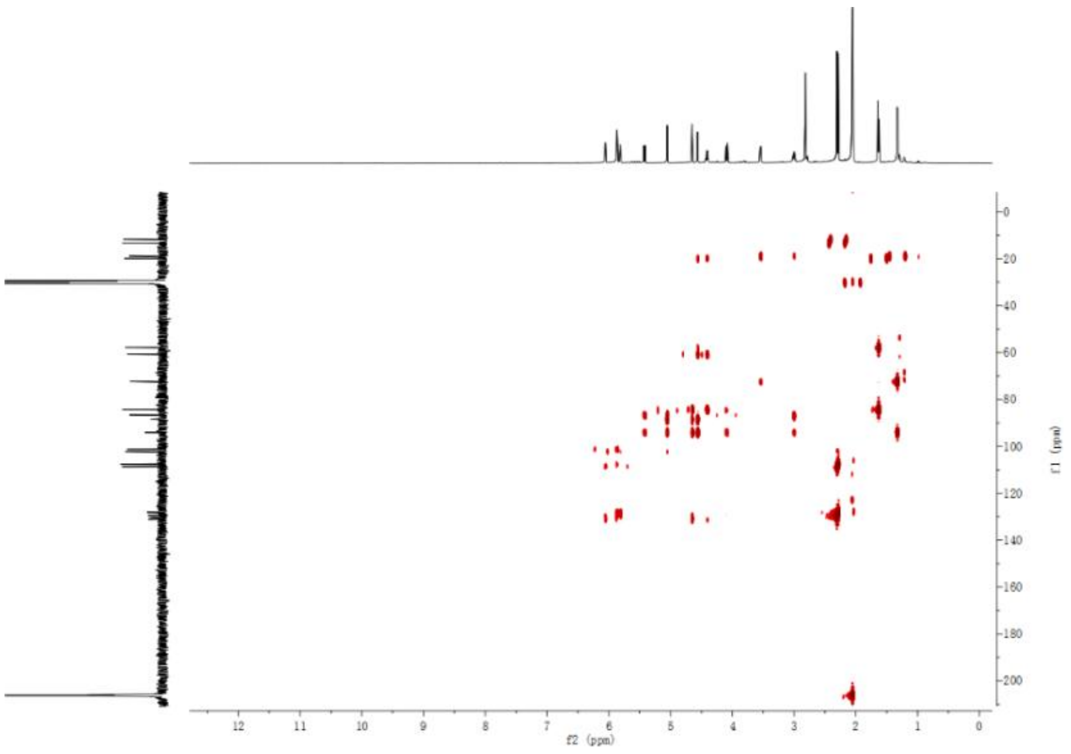

Fig. S77. HMBC spectrum of curvamine I (**16**).

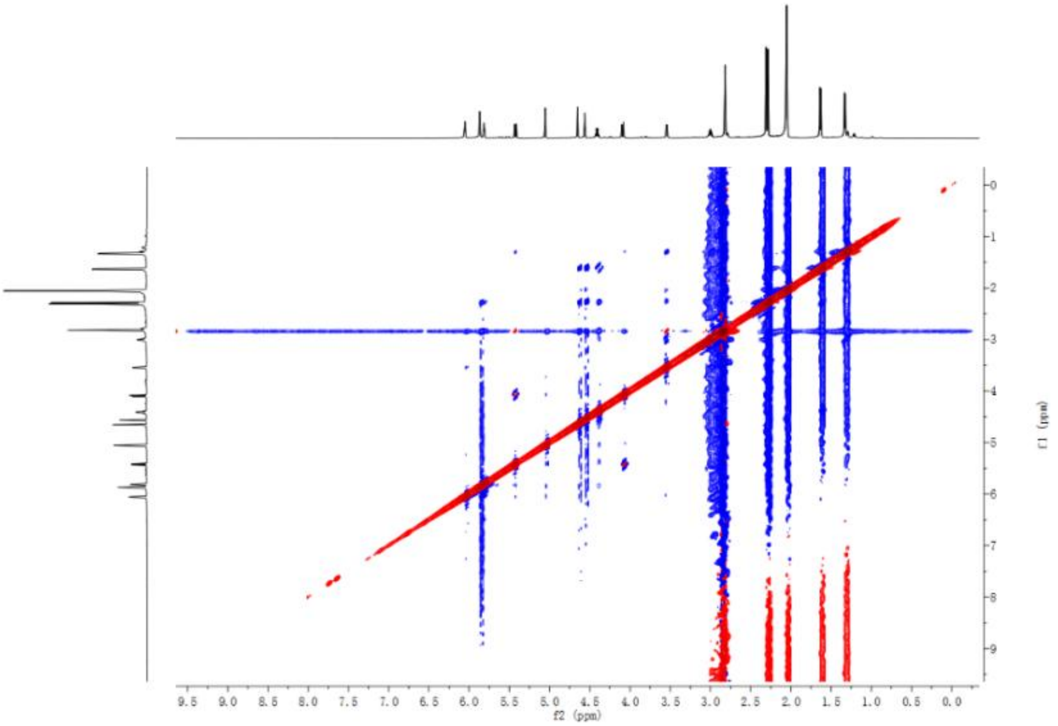

Fig. S78. NOESY spectrum of curvamine I (**16**).

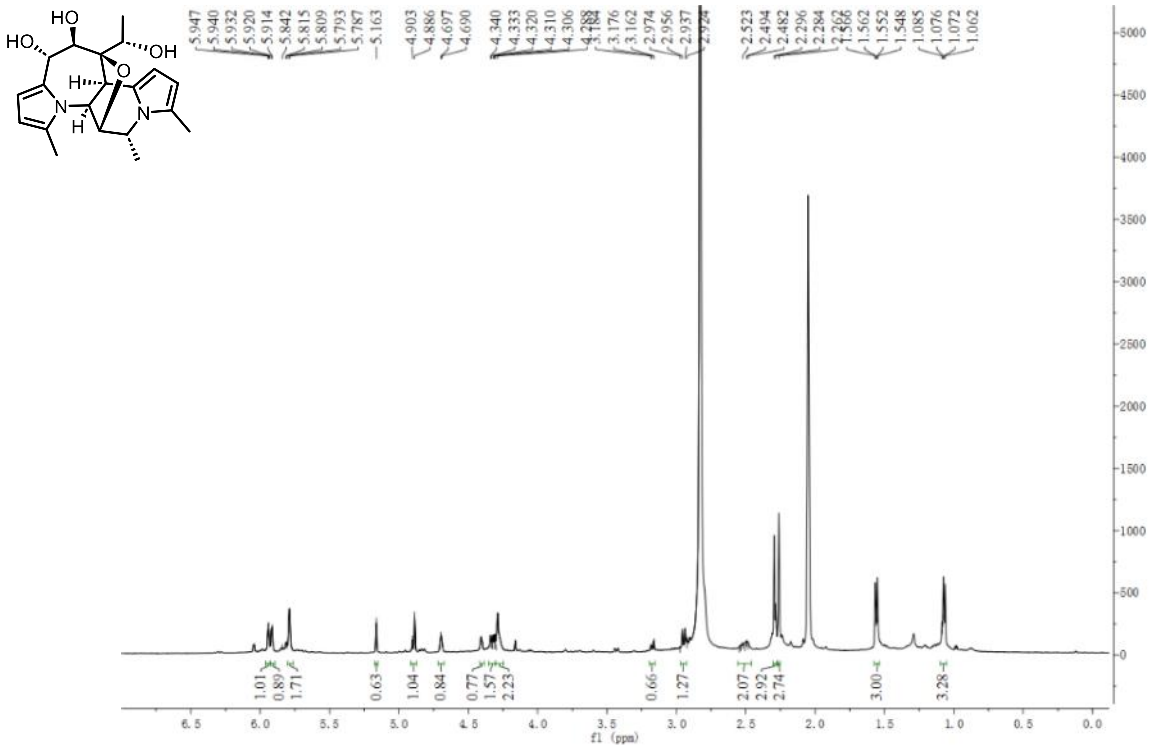

Fig. S79. ^{1}H NMR spectrum of curvamine J (**17**) (500 MHz).

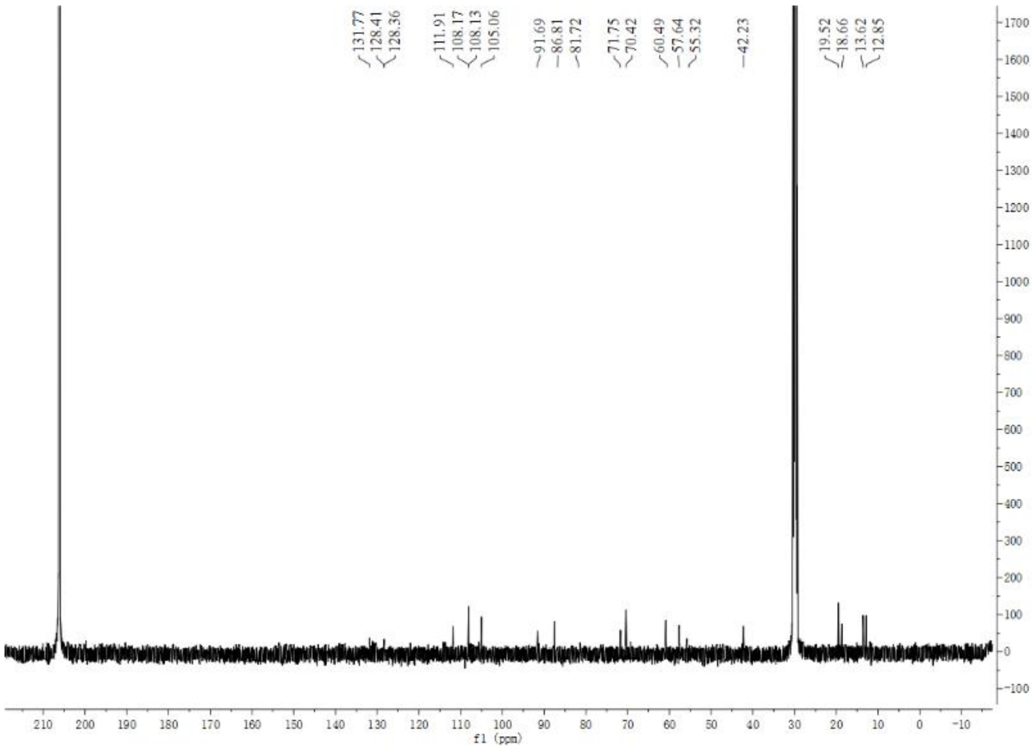

Fig. S80. ^{13}C NMR spectrum of curvamine J (**17**) (125 MHz).

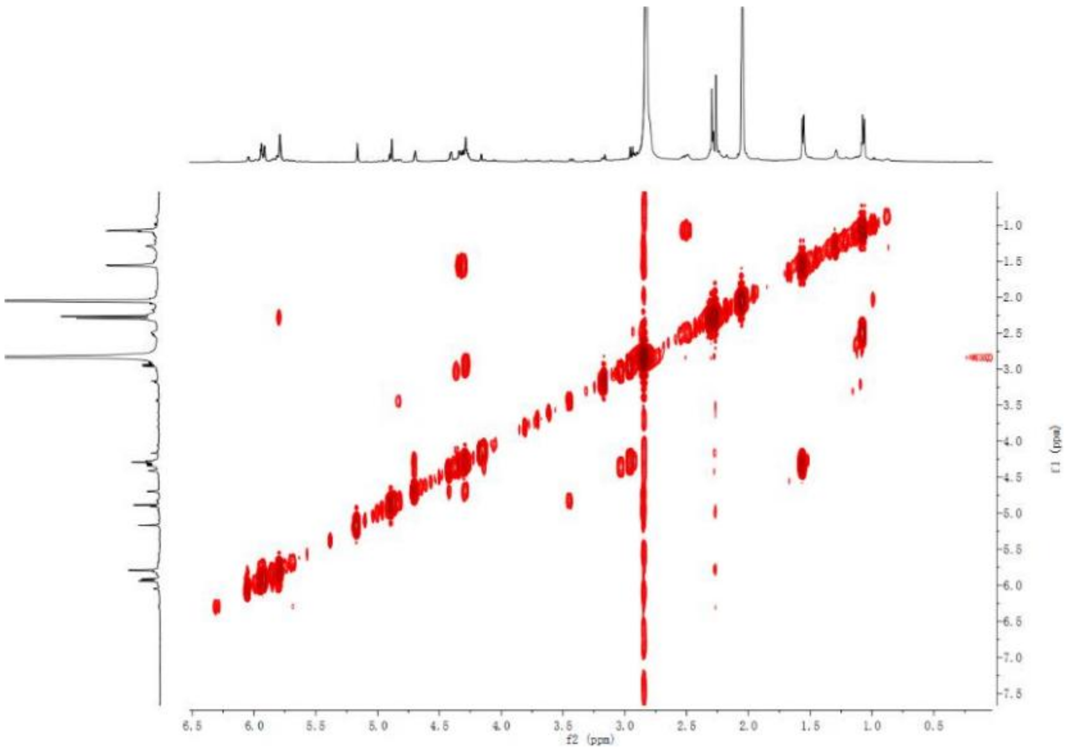

Fig. S81. ^{1}H-^{1}H COSY spectrum of curvamine J (**17**).

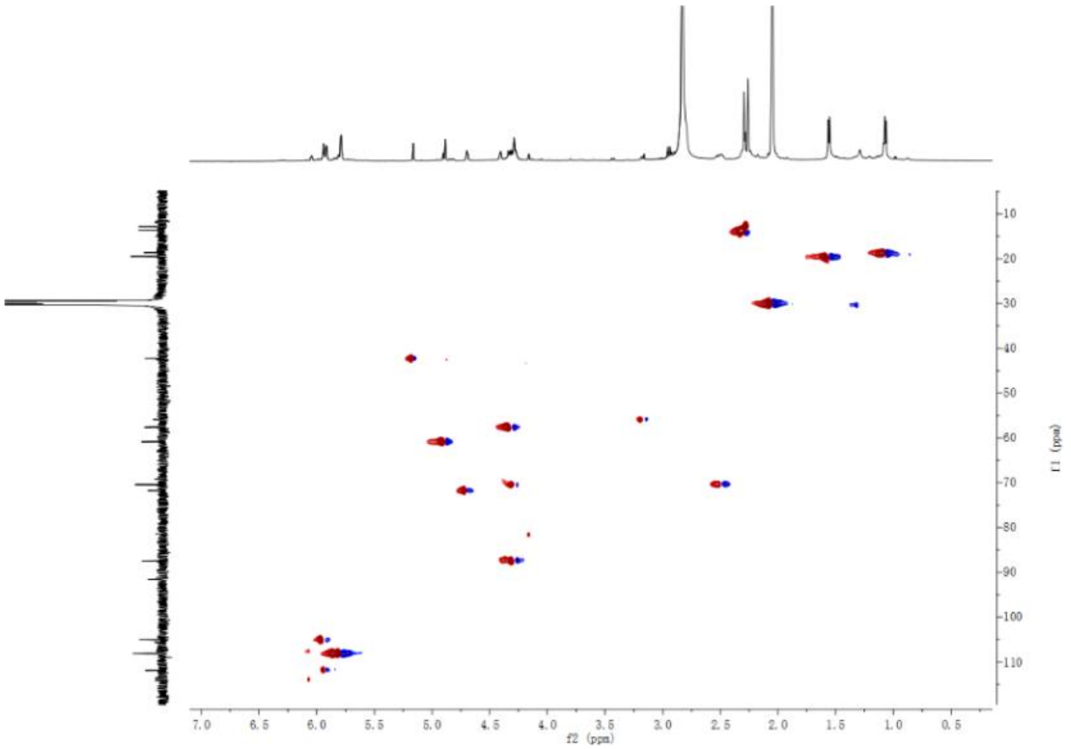

Fig. S82. HSQC spectrum of curvamine J (**17**).

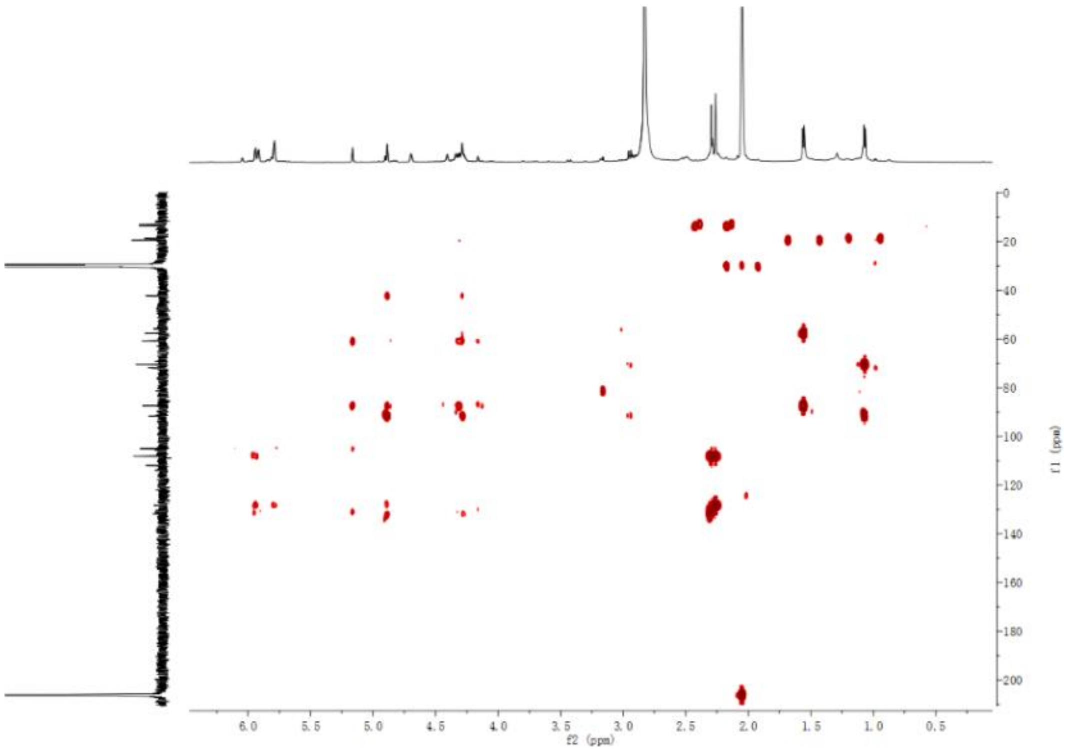

Fig. S83. HMBC spectrum of curvamine J (**17**).

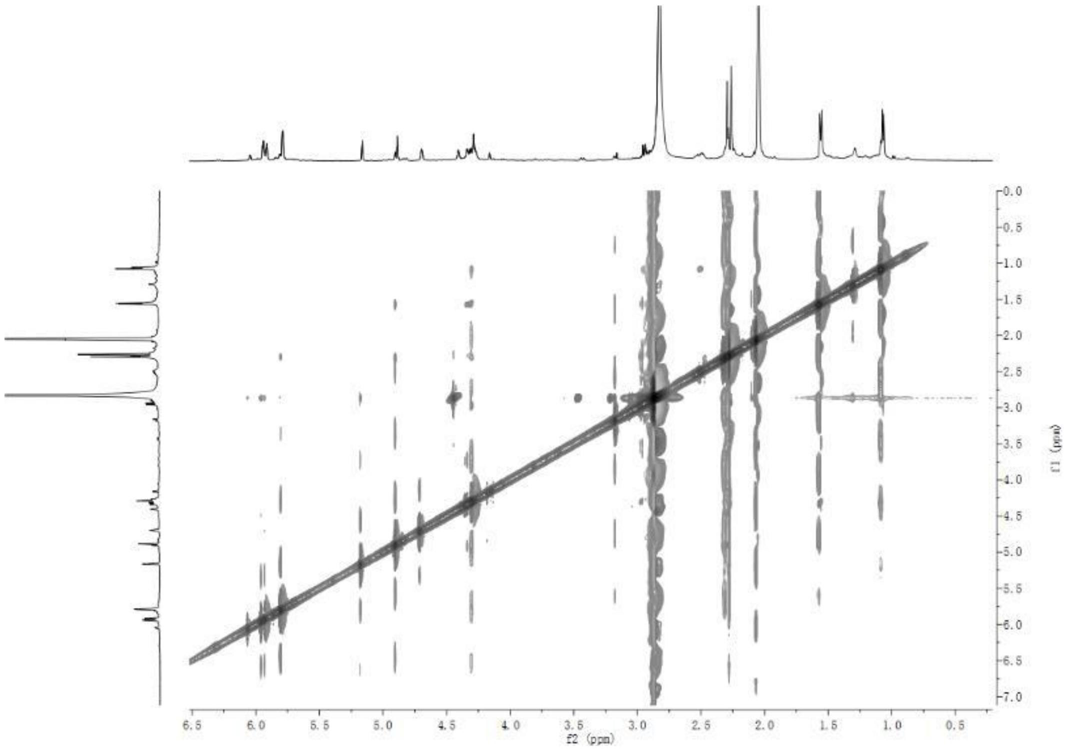

Fig. S84. NOESY spectrum of curvamine J (**17**).

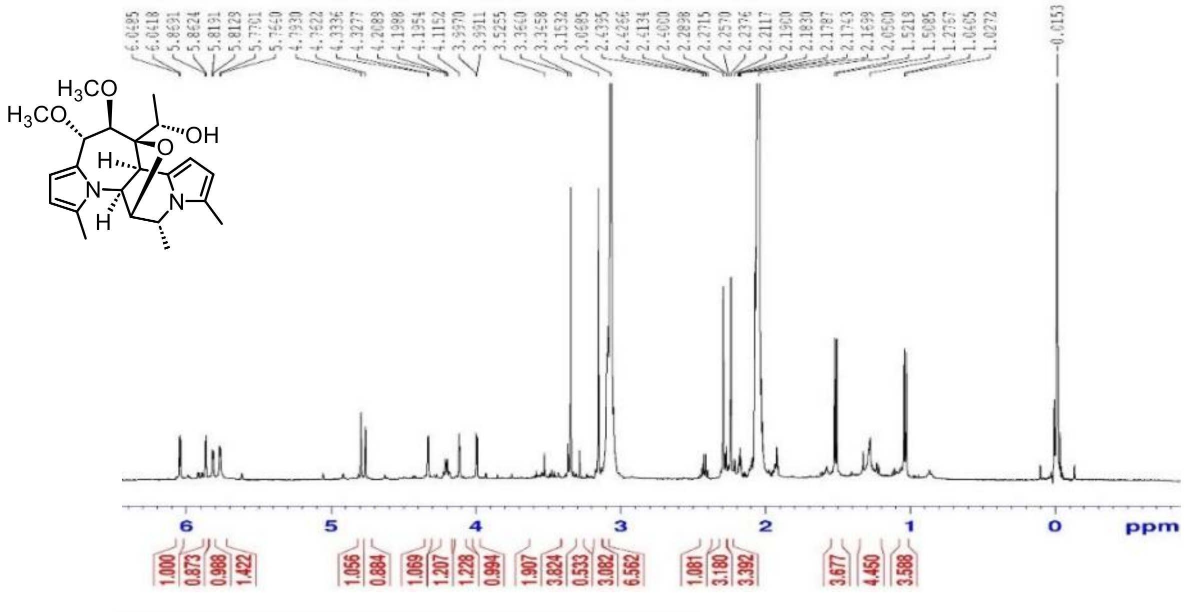

Fig. S85. ¹H NMR spectrum of curvamine K (**18**) (500 MHz).

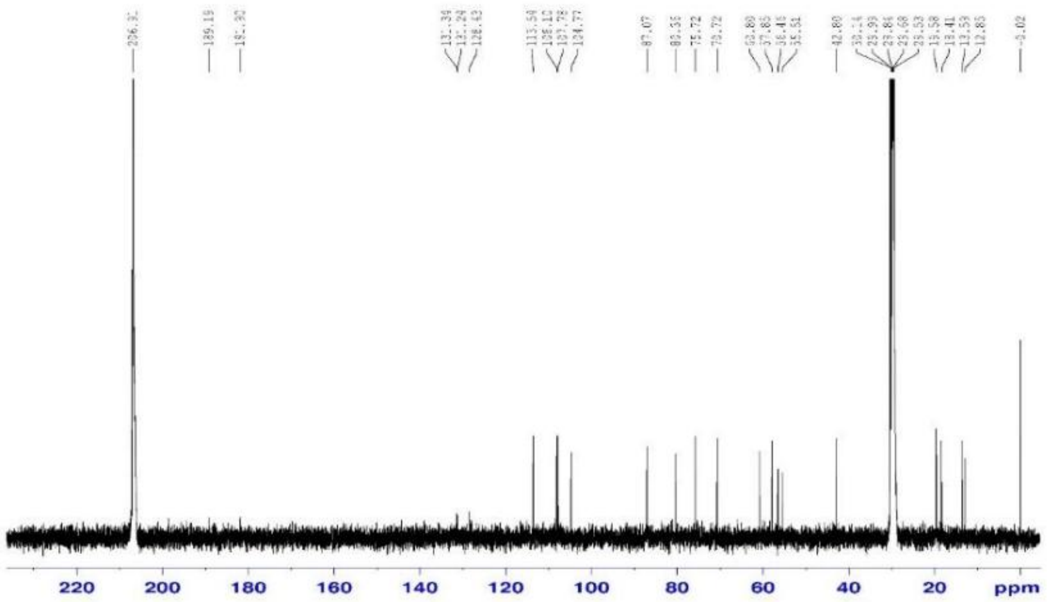

Fig. S86. ^{13}C NMR spectrum of curvamine K (**18**) (125 MHz).

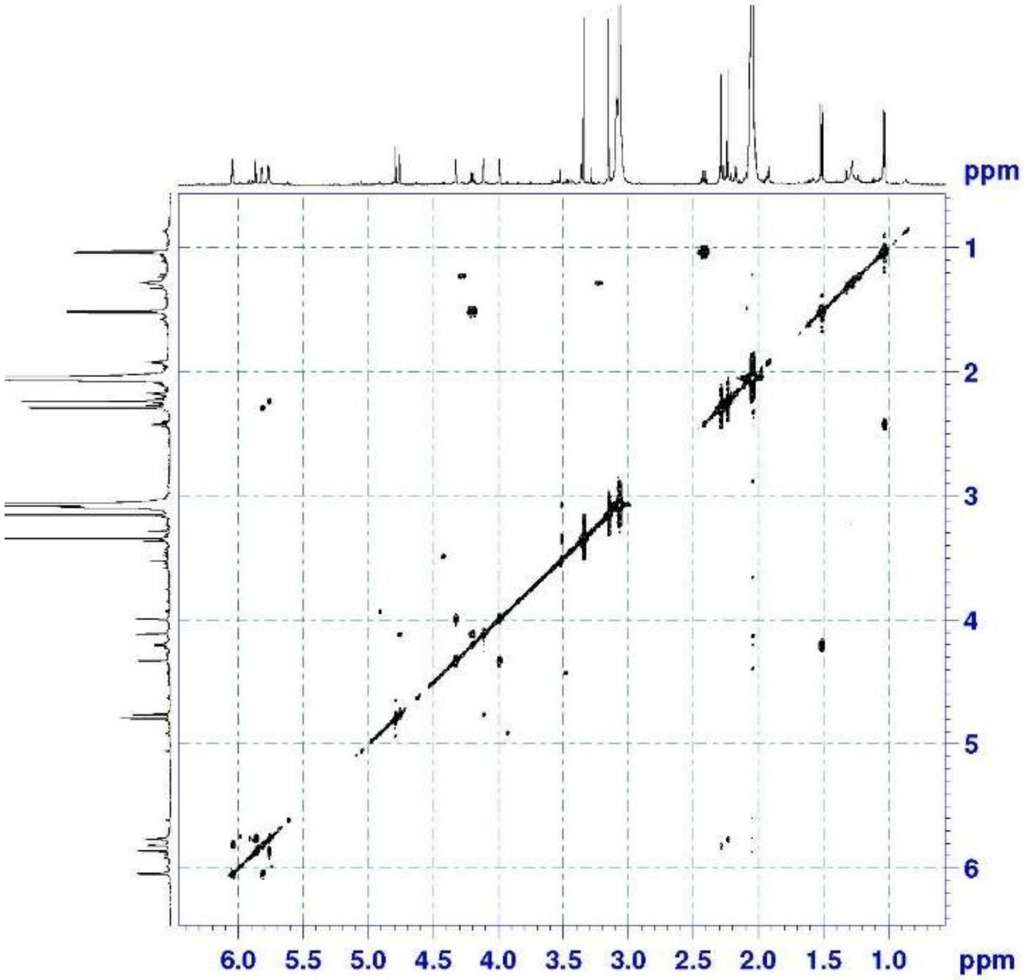

Fig. S87. ^{1}H-^{1}H COSY spectrum of curvamine K (**18**).

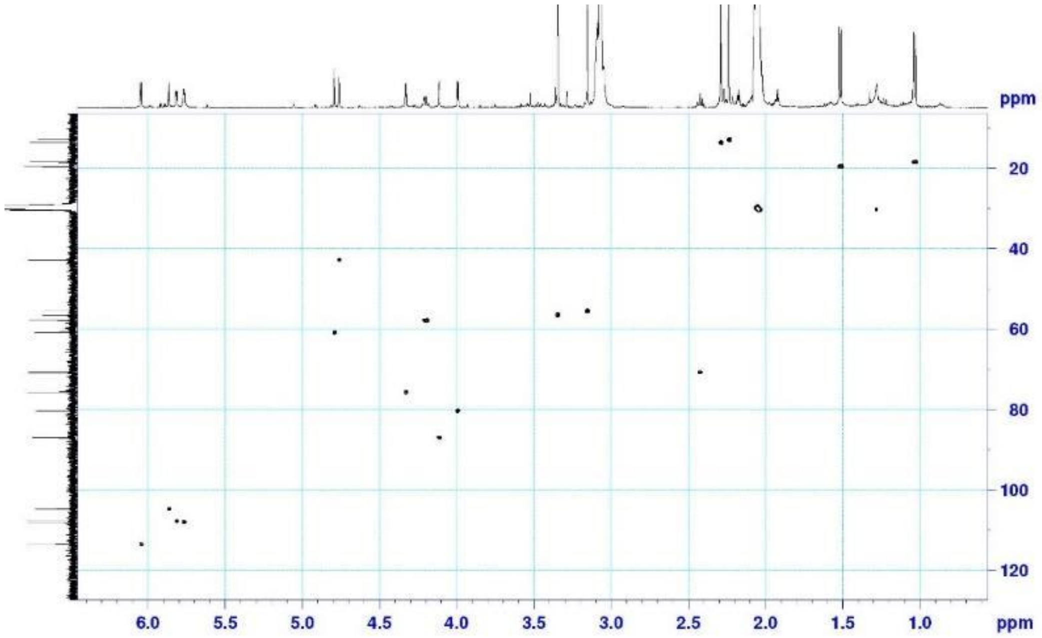

Fig. S88. HSQC spectrum of curvamine K (**18**).

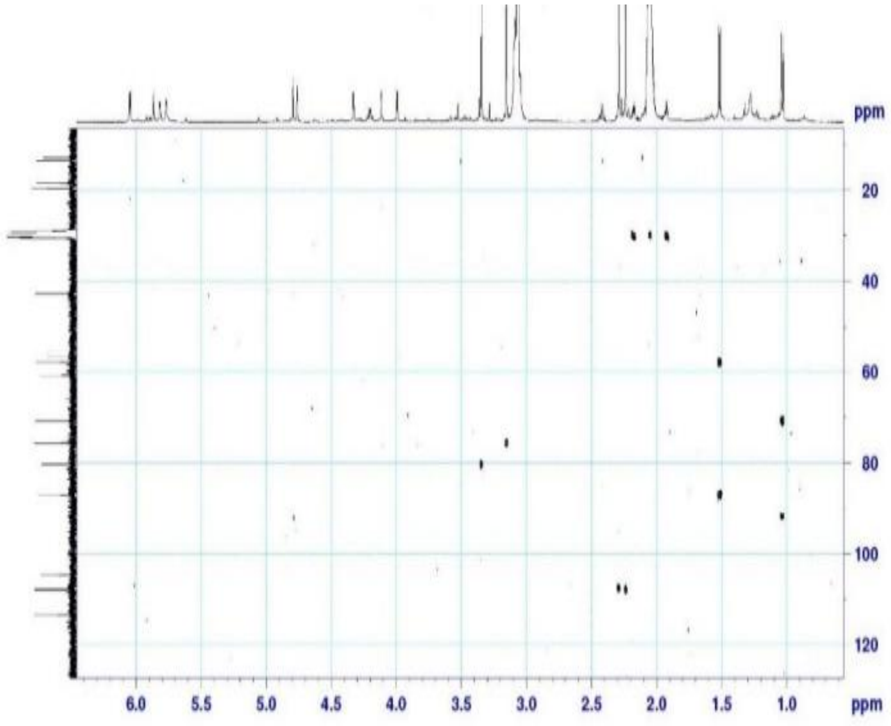

Fig. S89. HMBC spectrum of curvamine K (**18**).

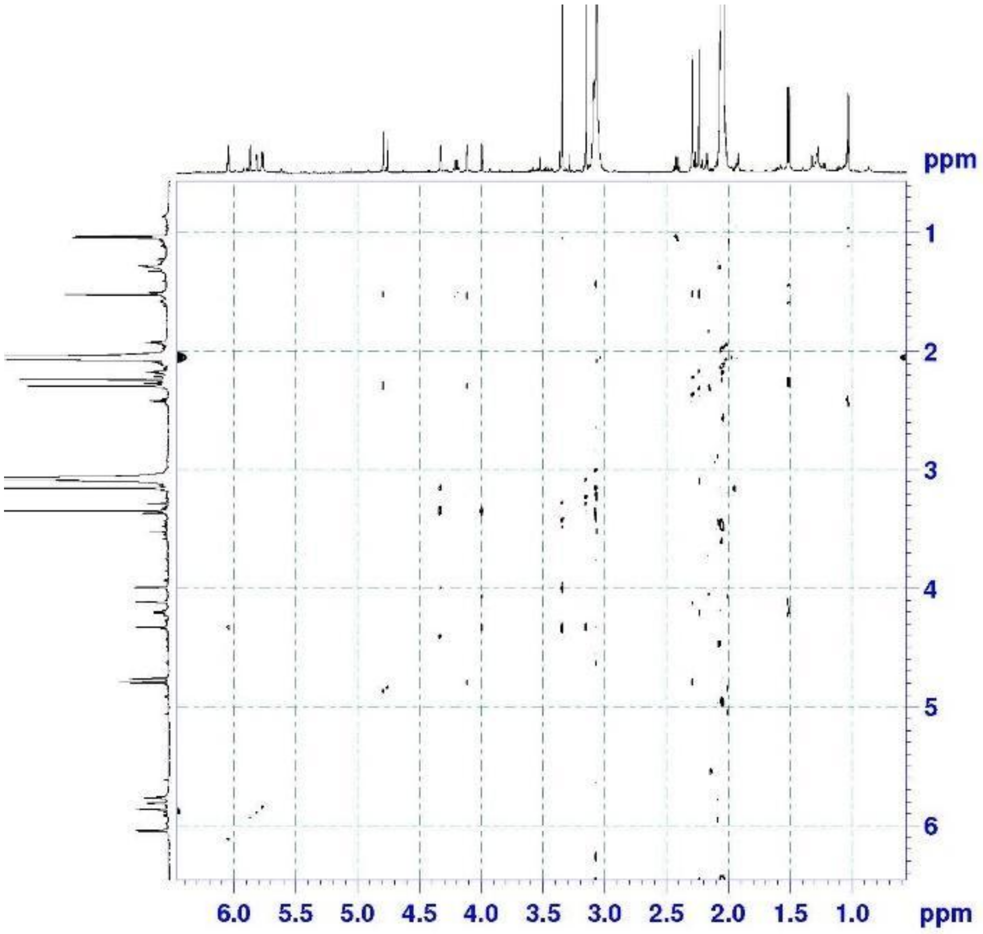

Fig. S90. NOESY spectrum of curvamine K (**18**).

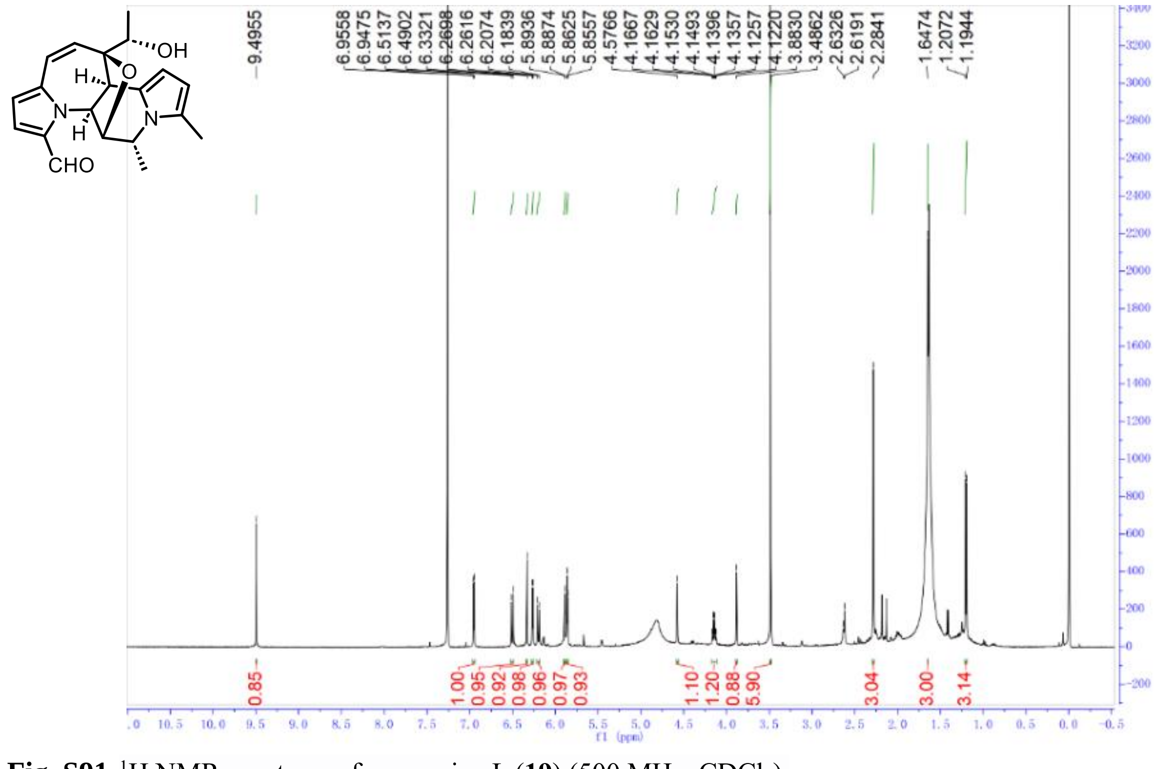

Fig. S91. ^{1}H NMR spectrum of curvamine L (**19**) (500 MHz, $CDCl_3$).

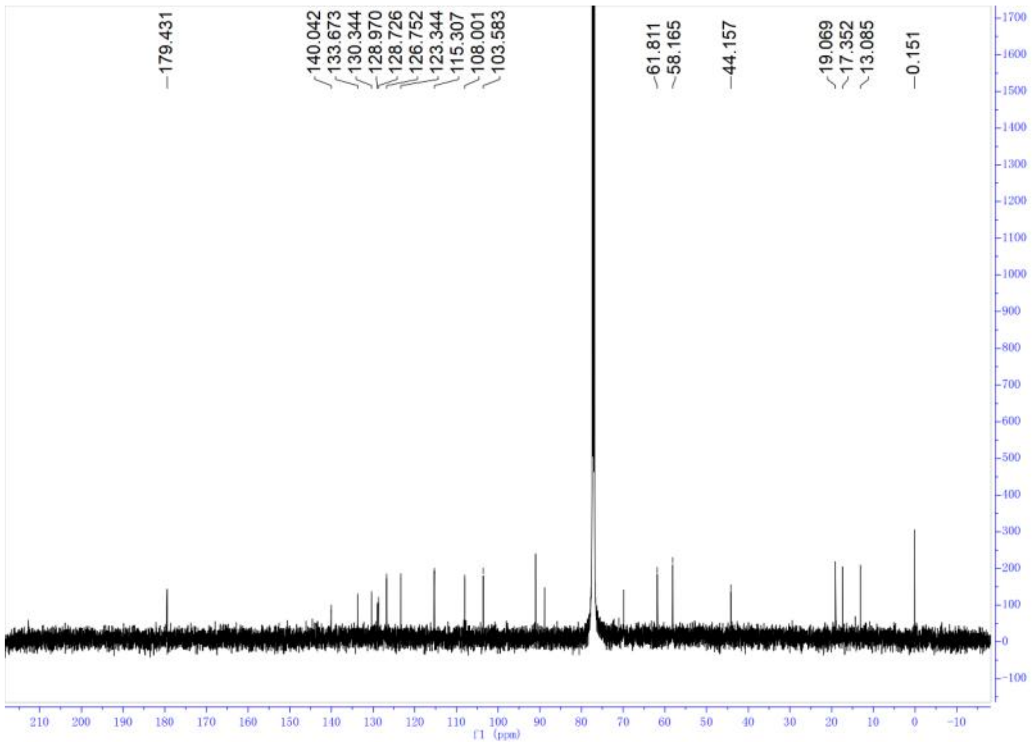

Fig. S92. ^{13}C NMR spectrum of curvamine L (**19**) (125 MHz, $CDCl_3$).

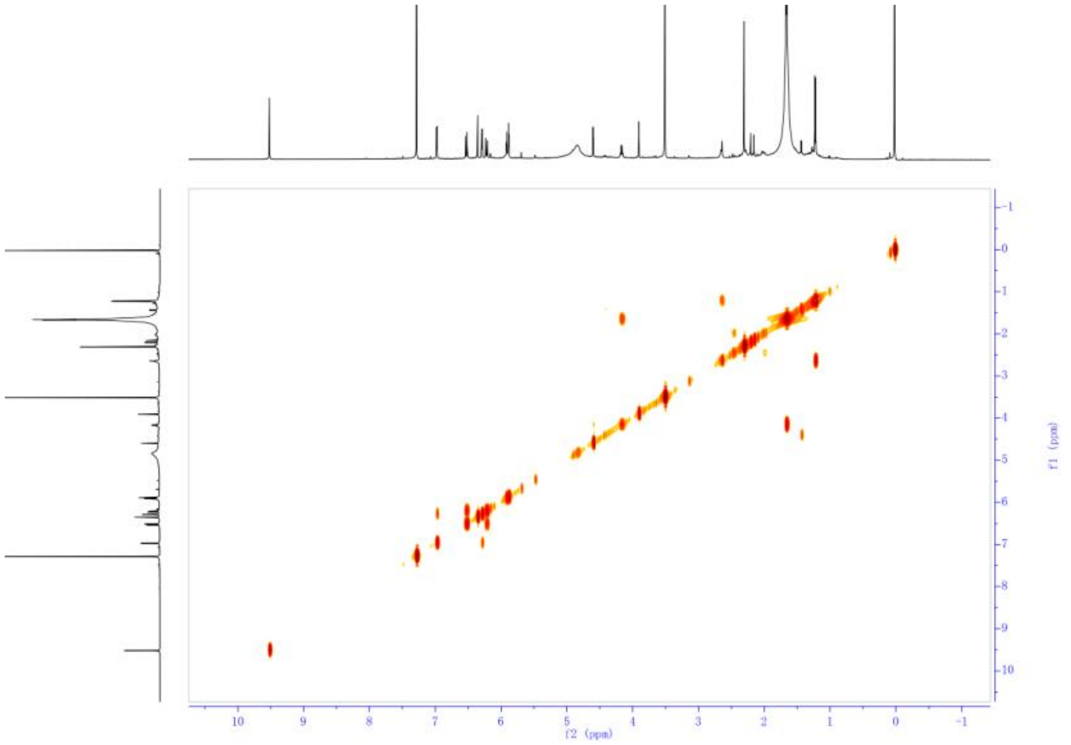

Fig. S93. ^{1}H-^{1}H COSY spectrum of curvamine L (**19**) in $CDCl_3$.

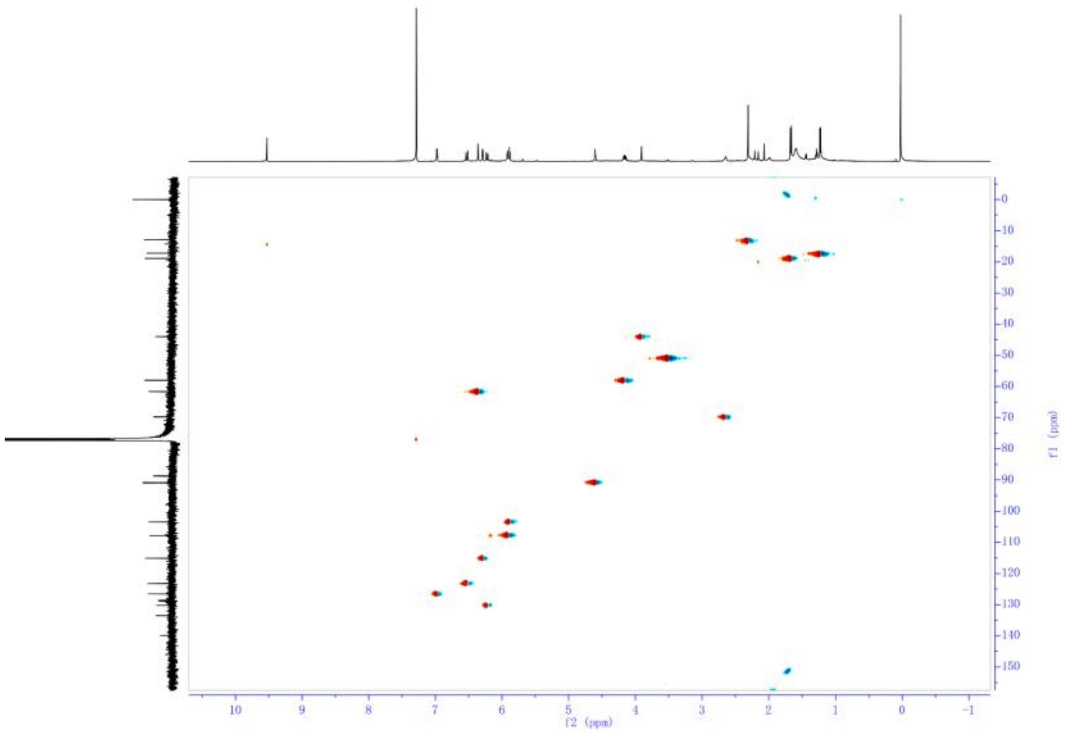

Fig. S94. HSQC spectrum of curvamine L (**19**) in $CDCl_3$.

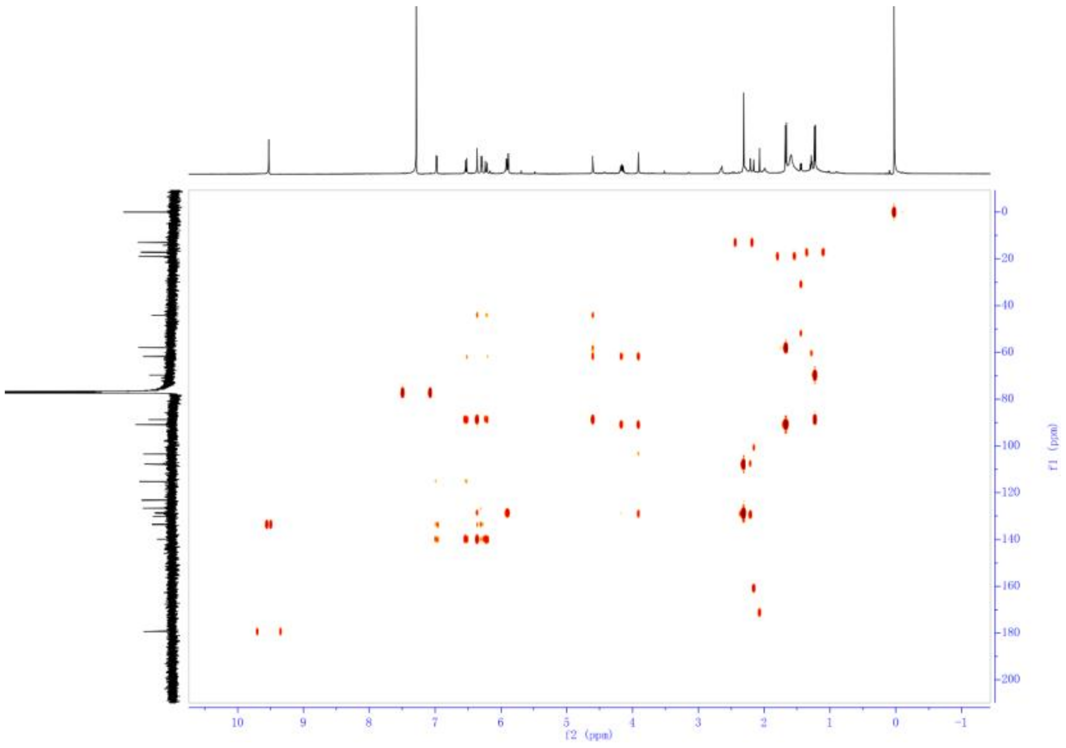

Fig. S95. HMBC spectrum of curvamine L (**19**) in $CDCl_3$.

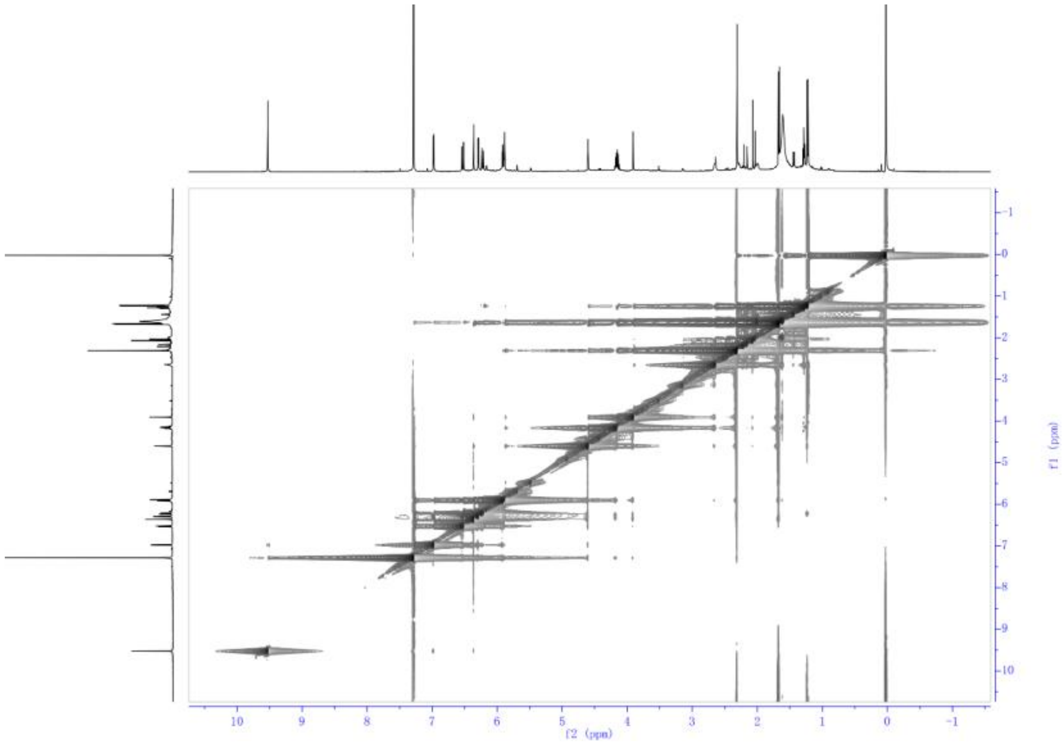

Fig. S96. NOESY spectrum of curvamine L (**19**) in $CDCl_3$.

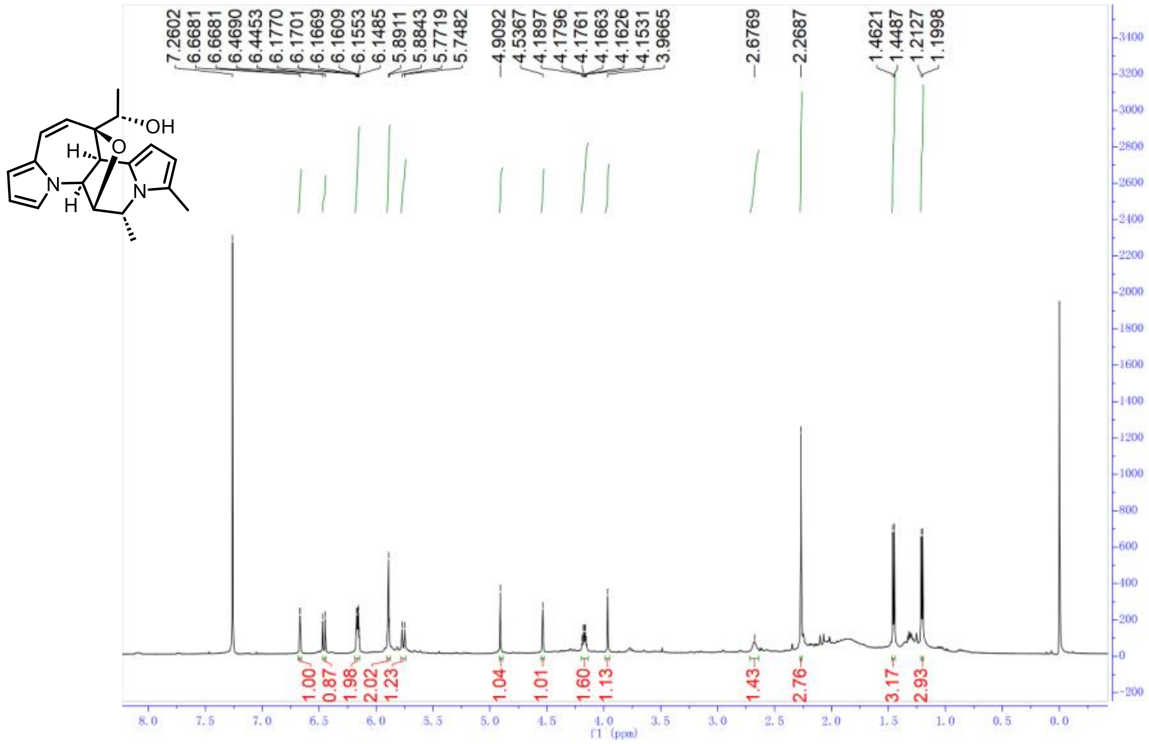

Fig. S97. ^1H NMR spectrum of curvamine M (**20**) (500 MHz, $CDCl_3$).

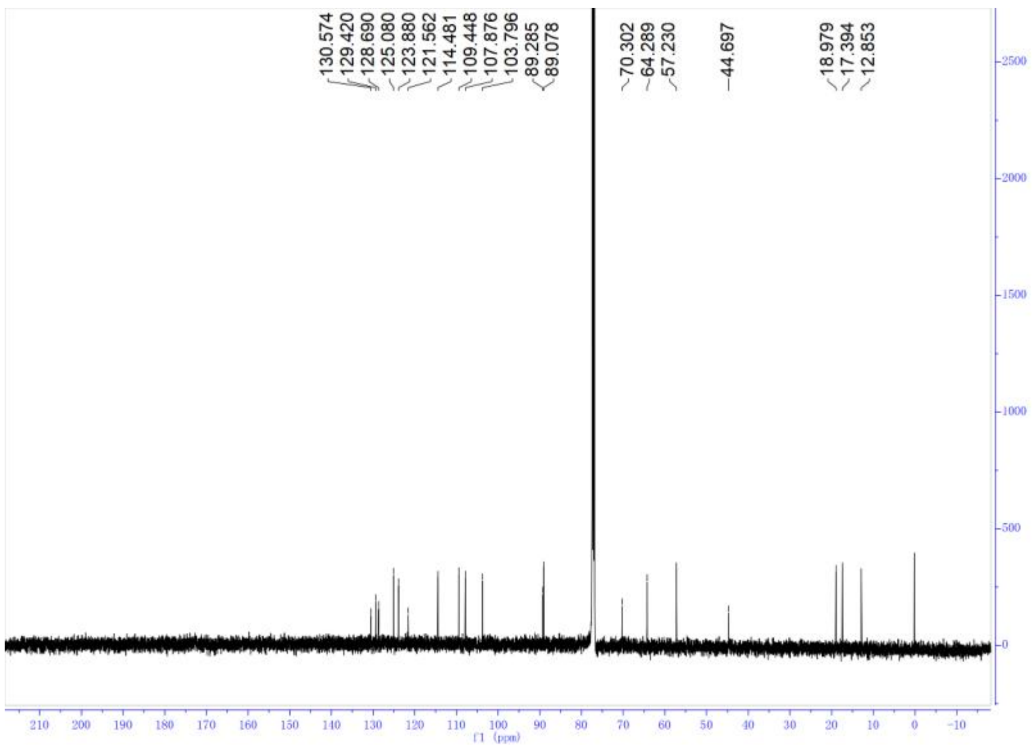

Fig. S98. ^{13}C NMR spectrum of curvamine M (**20**) (125 MHz, $CDCl_3$).

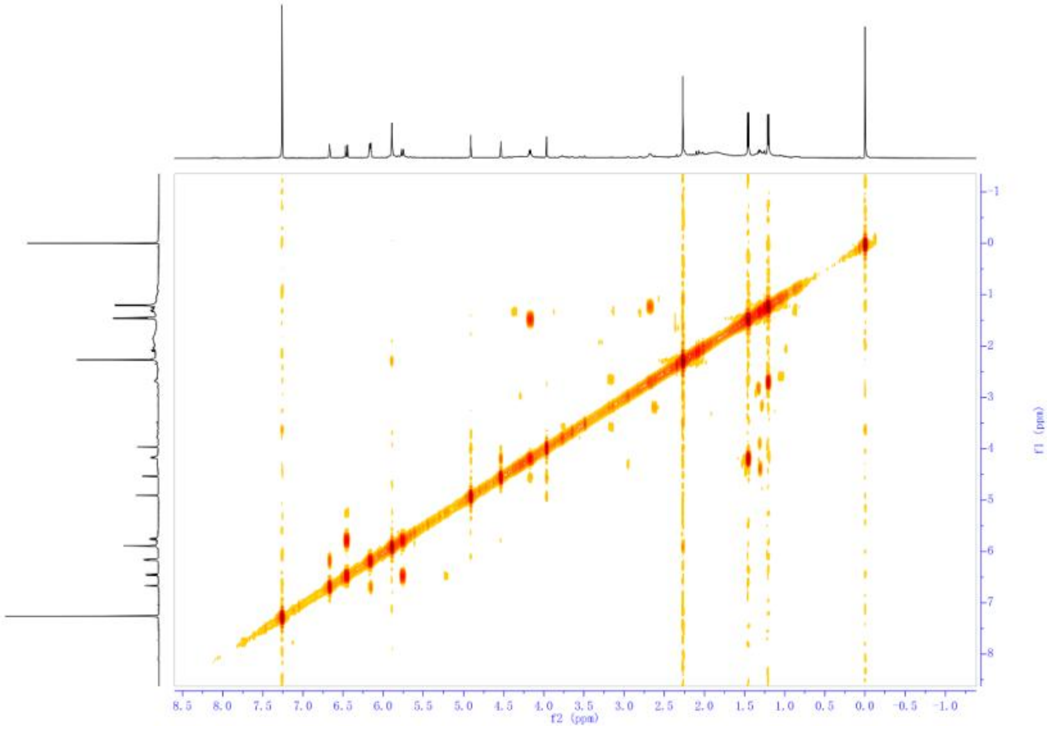

Fig. S99. ^{1}H-^{1}H COSY spectrum of curvamine M (**20**) in $CDCl_3$.

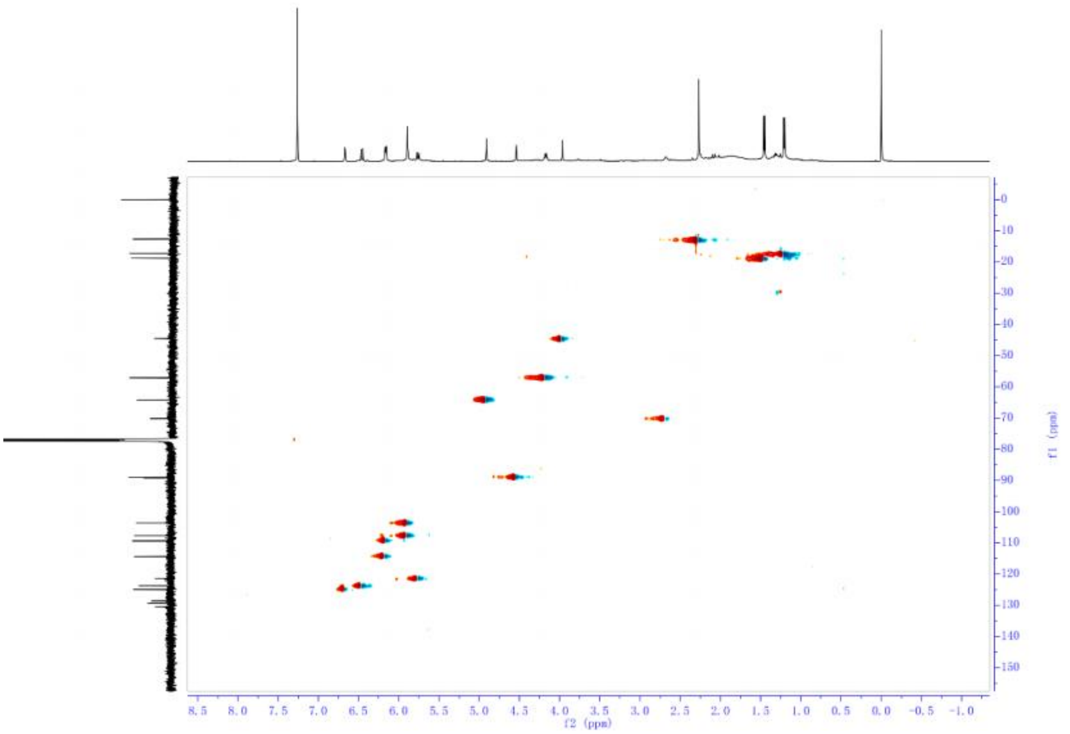

Fig. S100. HSQC spectrum of curvamine M (**20**) in $CDCl_3$.

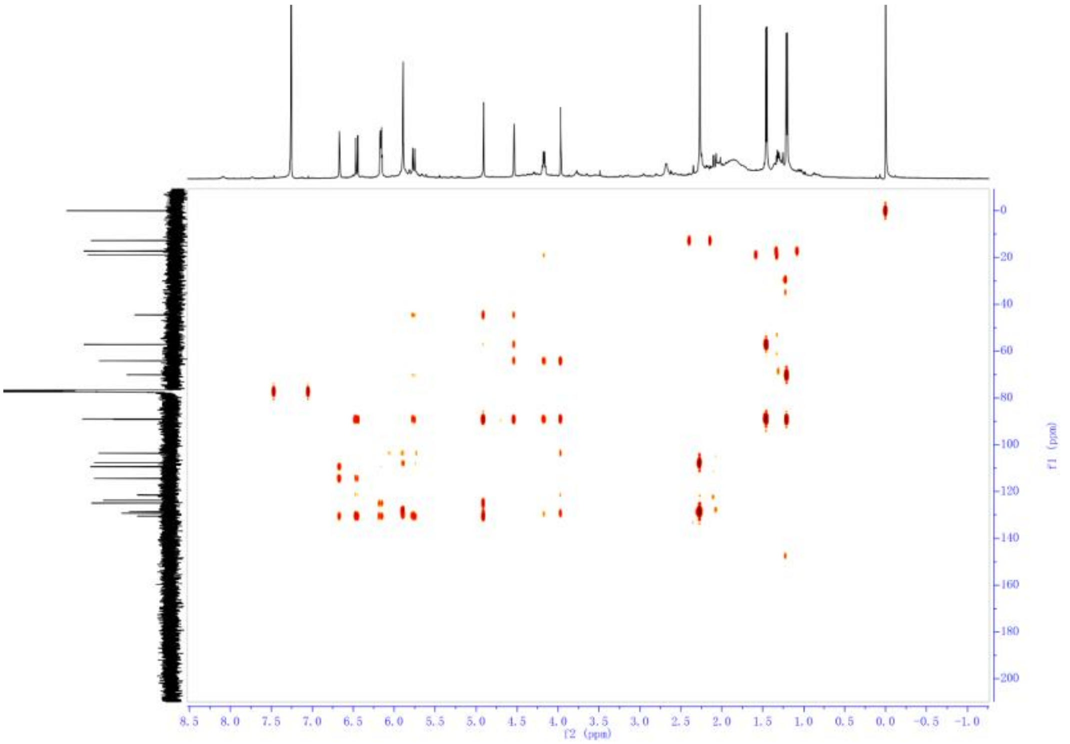

Fig. S101. HMBC spectrum of curvamine M (**20**) in $CDCl_3$.

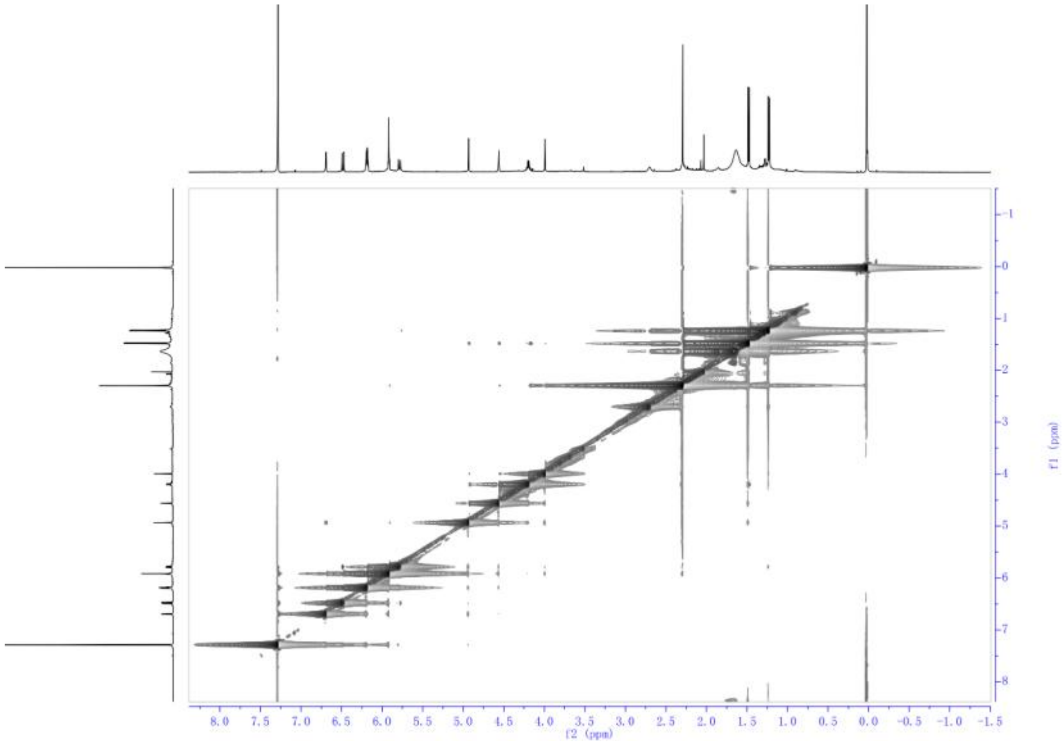

Fig. S102. NOESY spectrum of curvamine M (**20**) in $CDCl_3$.

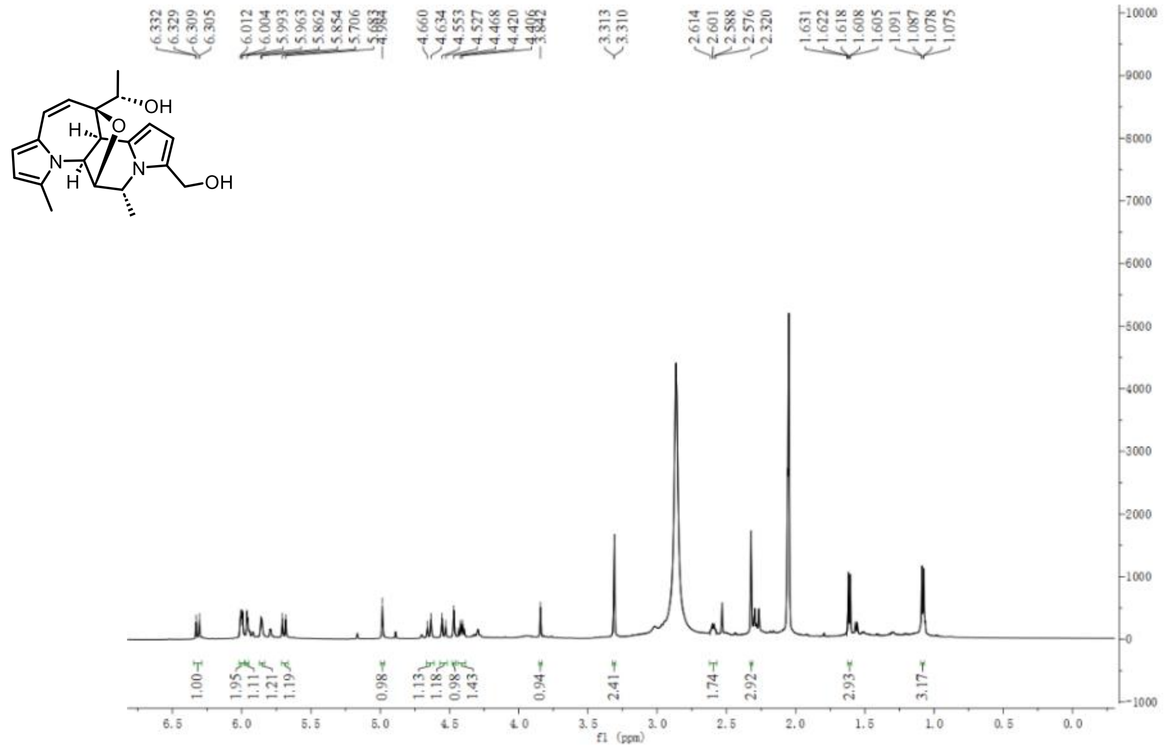

Fig. S103. ^1H NMR spectrum of curvamine N (**21**) (500 MHz).

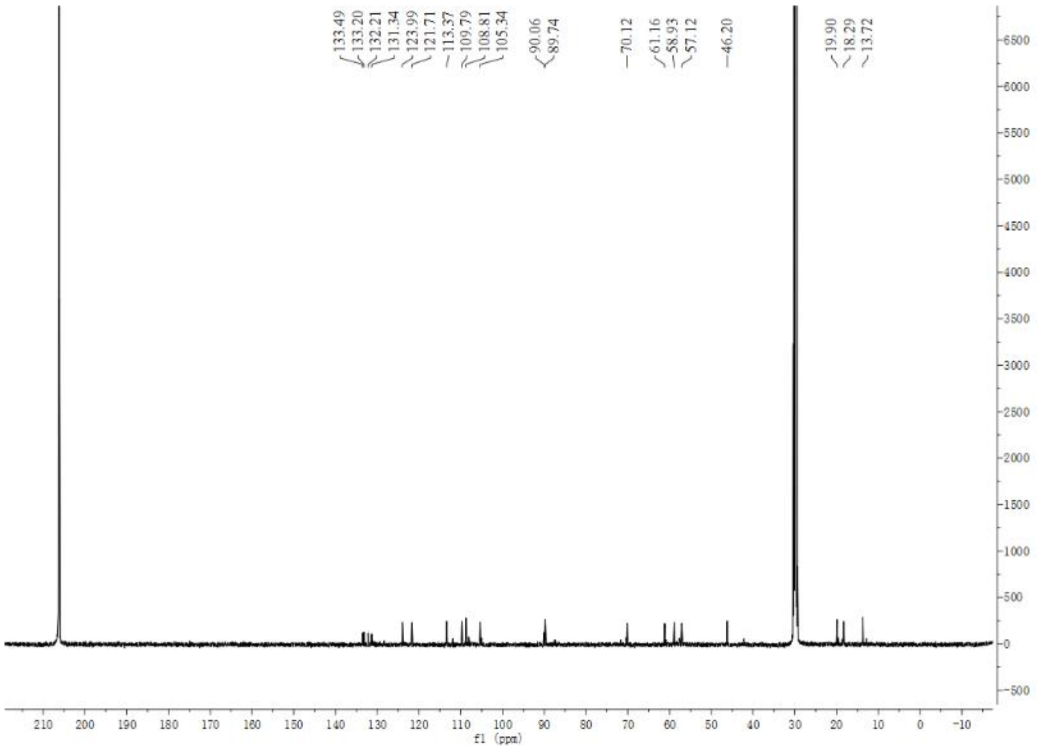

Fig. S104. ^{13}C NMR spectrum of curvamine N (**21**) (125 MHz).

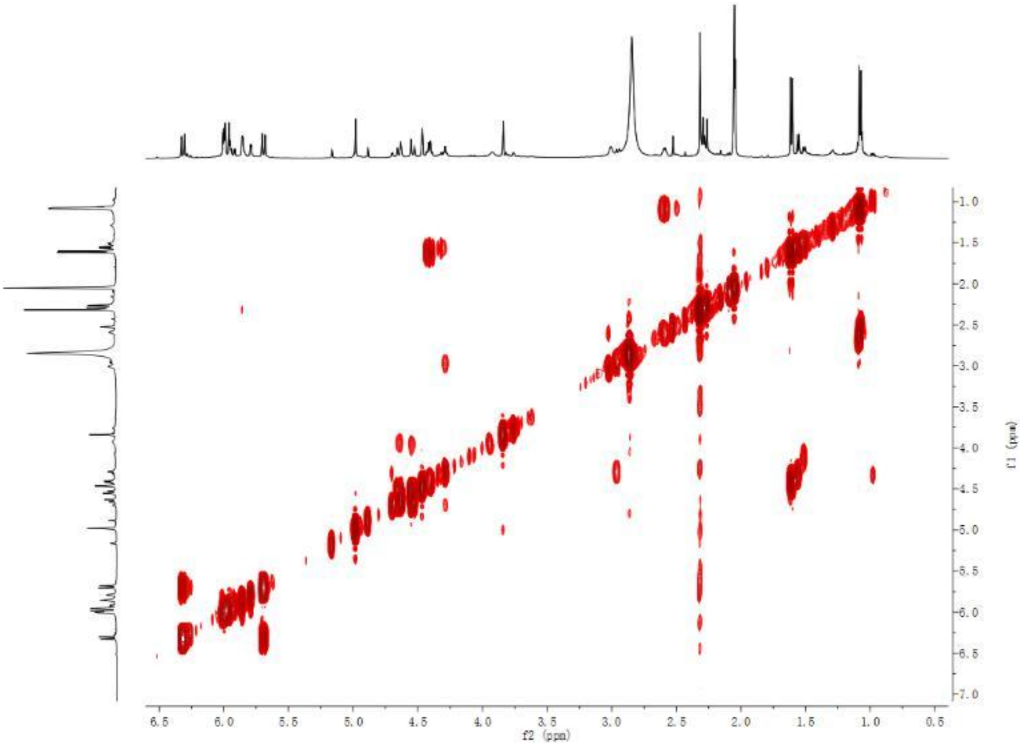

Fig. S105. ^{1}H-^{1}H COSY spectrum of curvamine N (**21**).

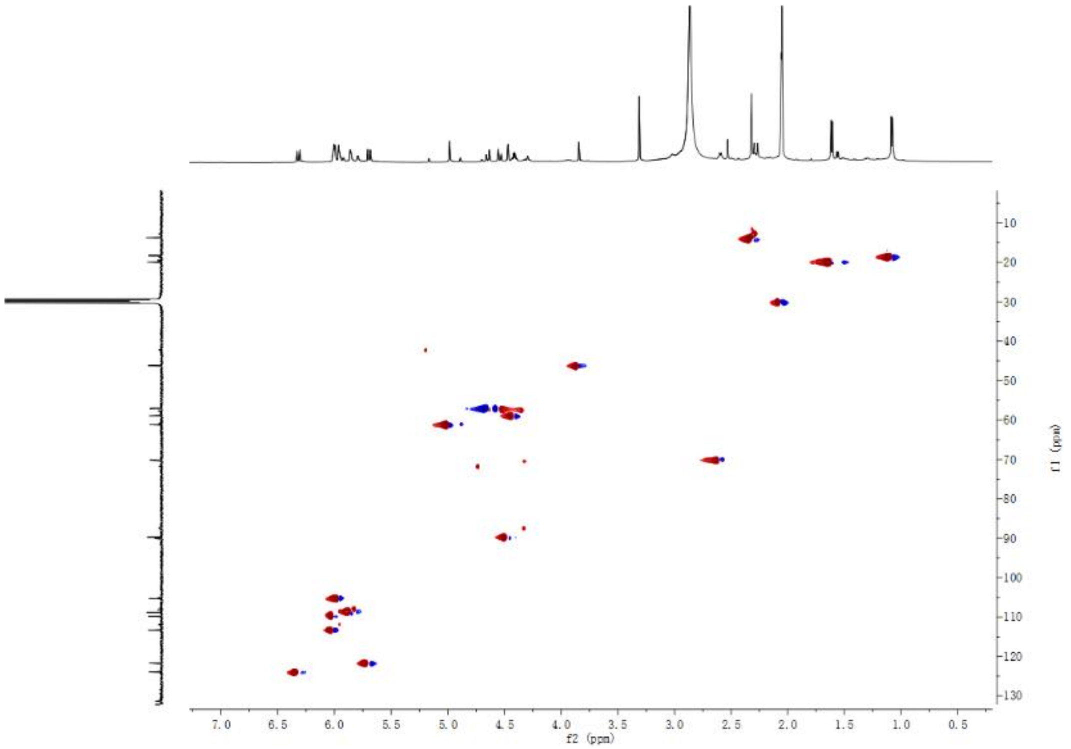

Fig. S106. HSQC spectrum of curvamine N (**21**).

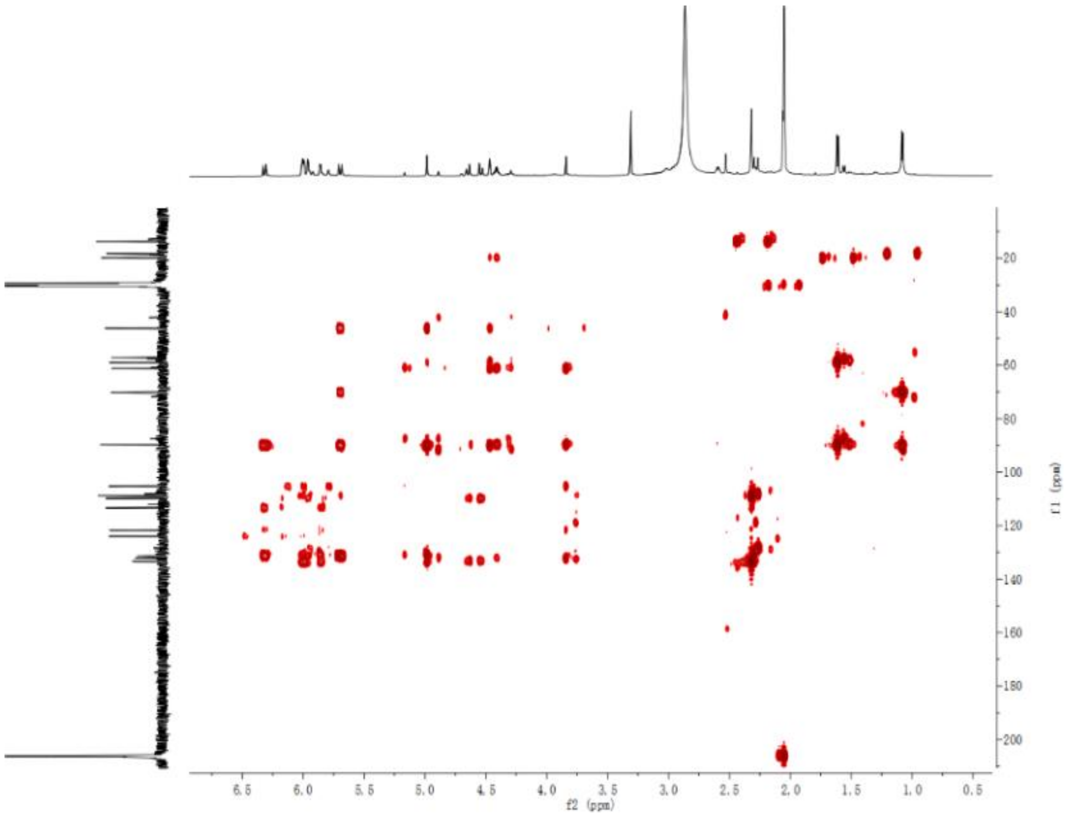

Fig. S107. HMBC spectrum of curvamine N (**21**).

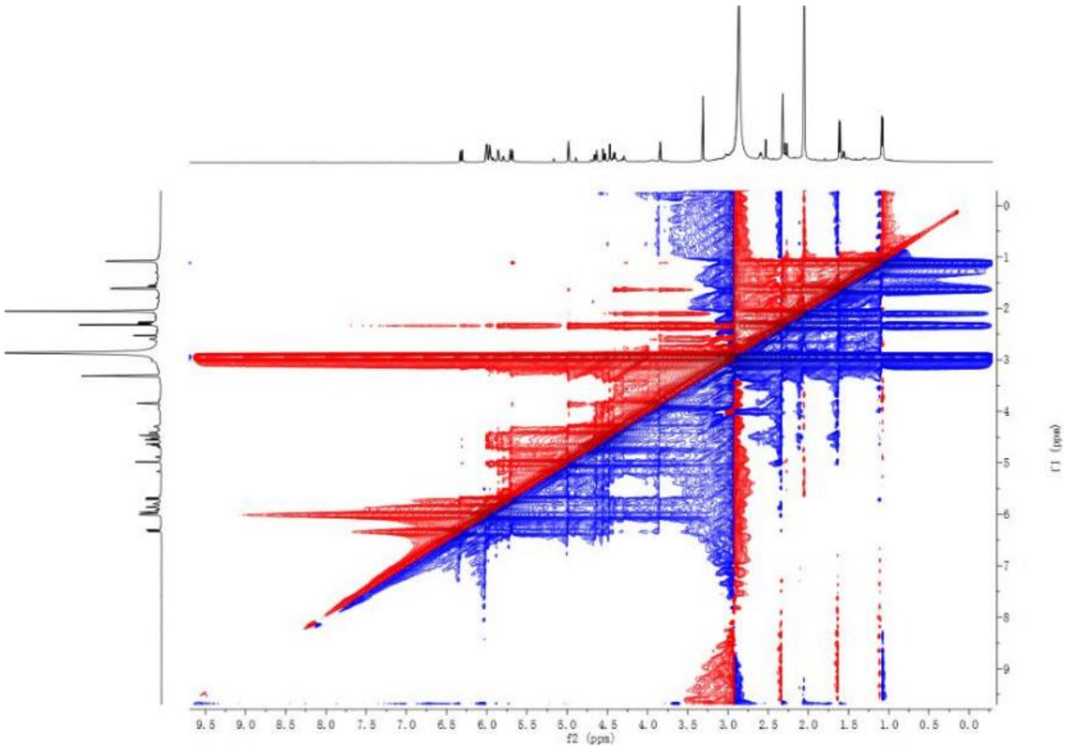

Fig. S108. NOESY spectrum of curvamine N (**21**).

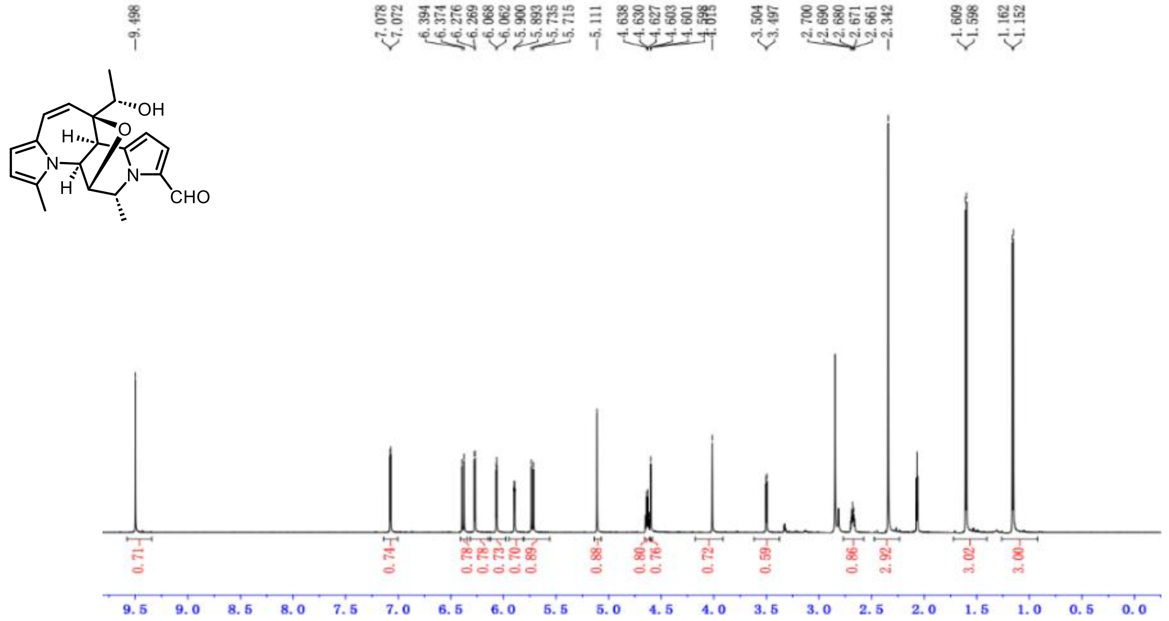

Fig. S109. ^{1}H NMR spectrum of curvamine O (**22**) (500 MHz).

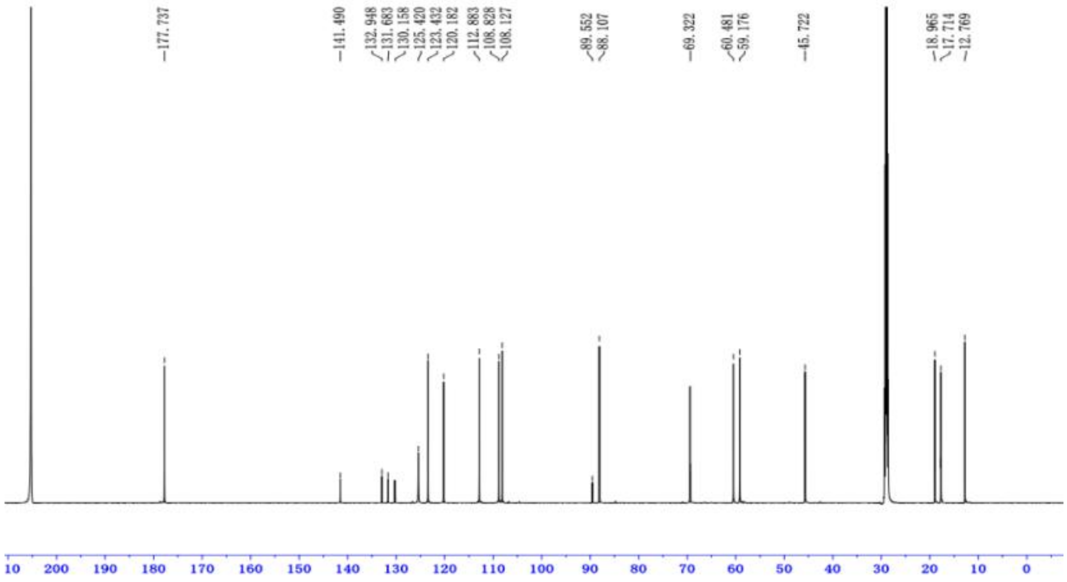

Fig. S110. ^{13}C NMR spectrum of curvamine O (**22**) (125 MHz).

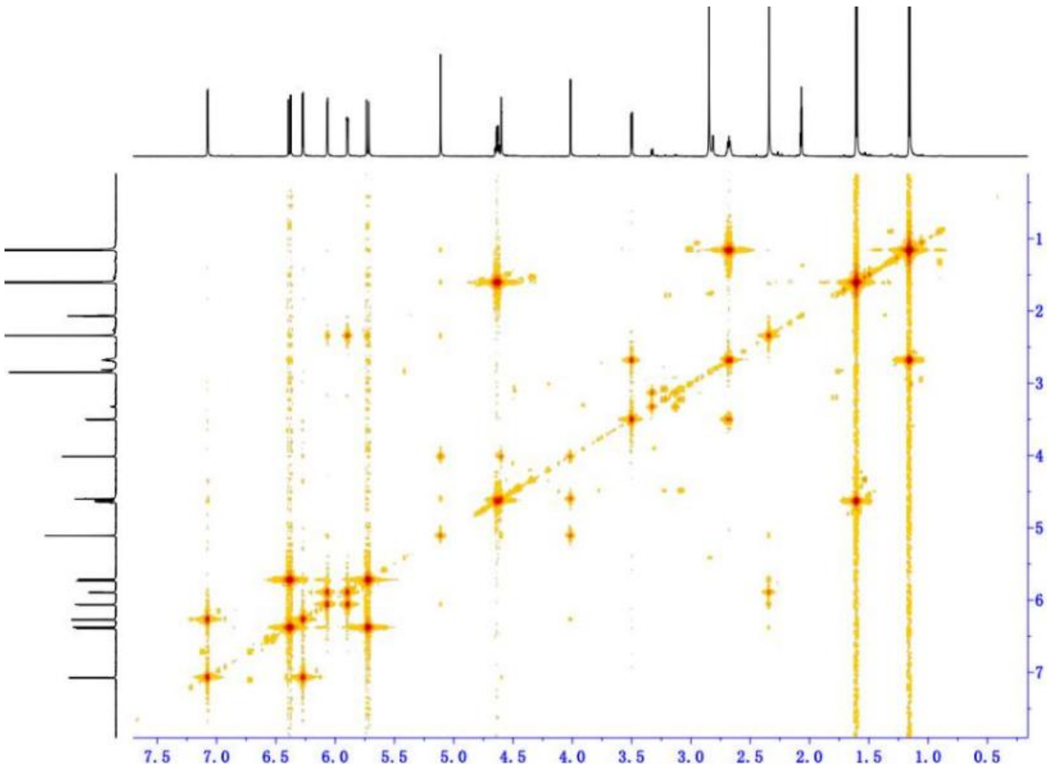

Fig. S111. ^{1}H-^{1}H COSY spectrum of curvamine O (**22**).

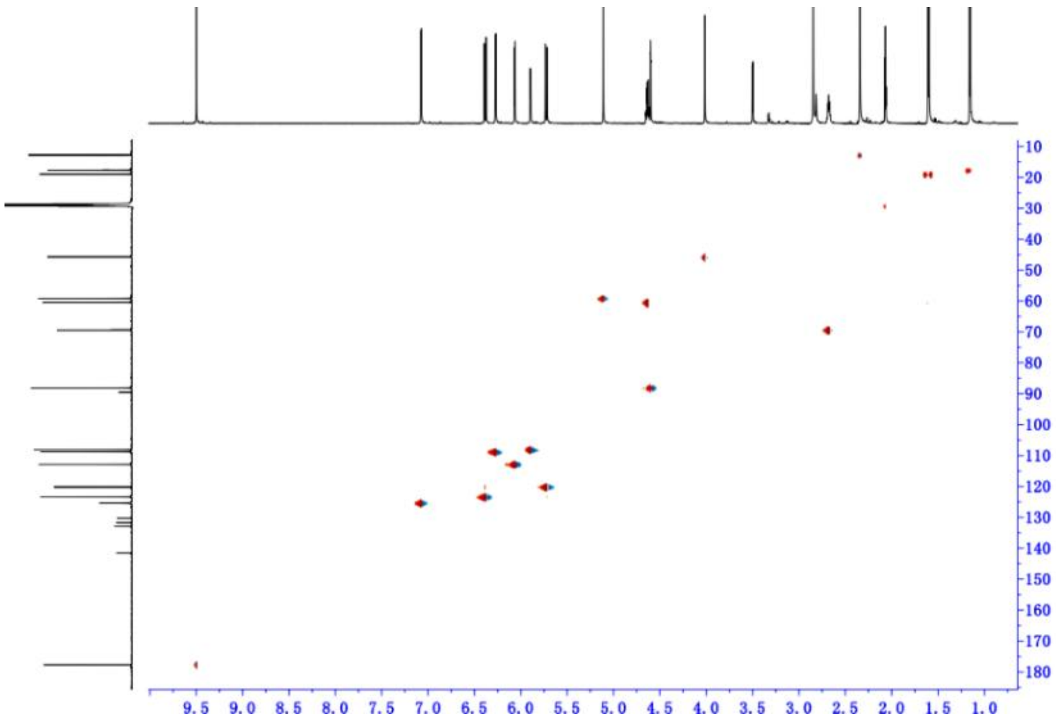

Fig. S112. HSQC spectrum of curvamine O (**22**).

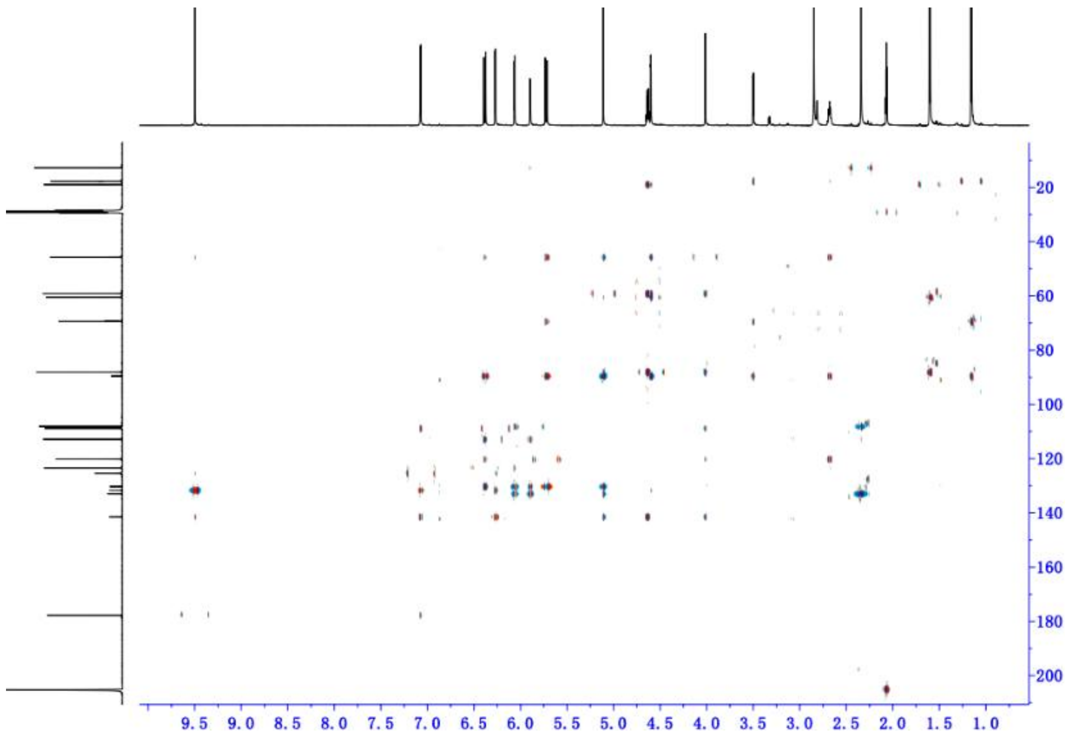

Fig. S113. HMBC spectrum of curvamine O (**22**).

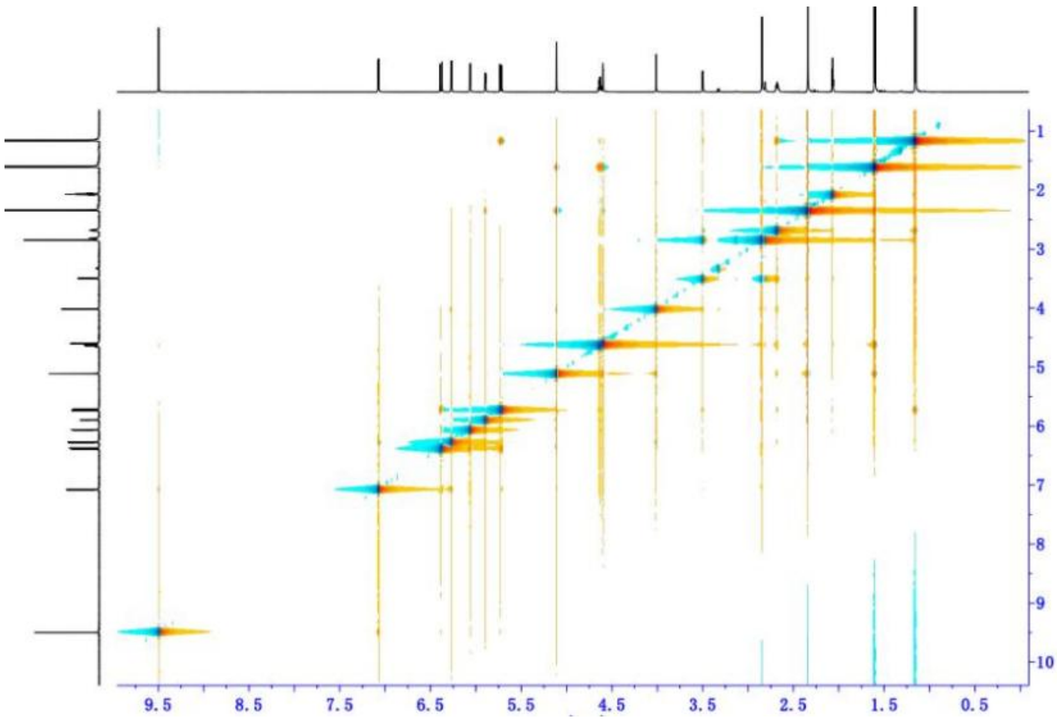

Fig. S114. NOESY spectrum of curvamine O (**22**).

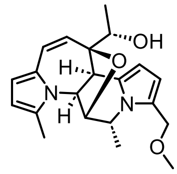

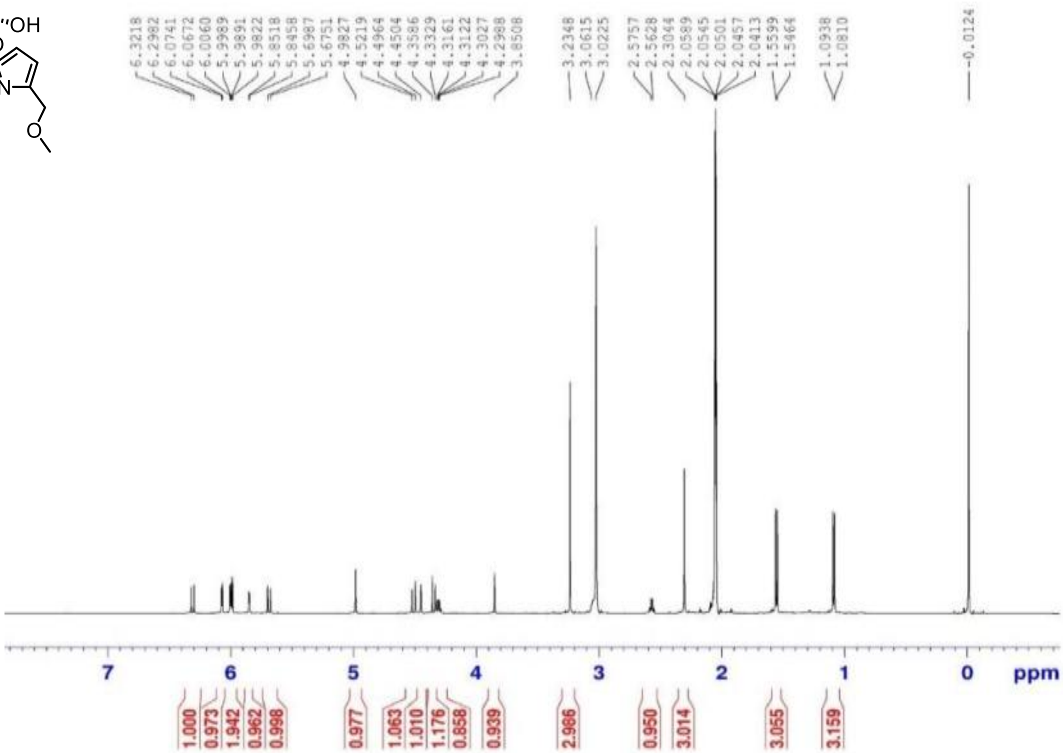

Fig. S115. ^{1}H NMR spectrum of curvamine P (**23**) (500 MHz).

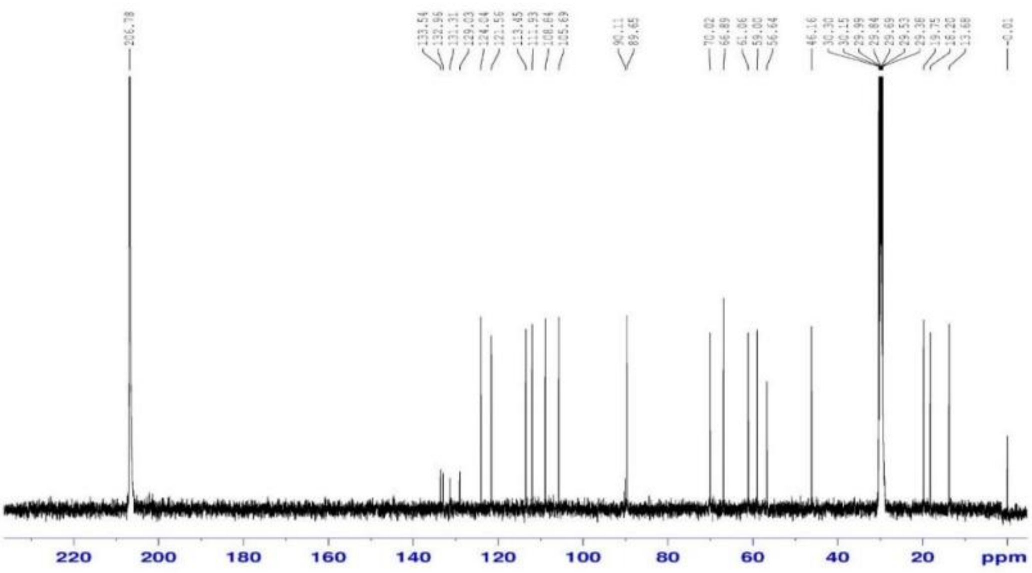

Fig. S116. ^{13}C NMR spectrum of curvamine P (**23**) (125 MHz).

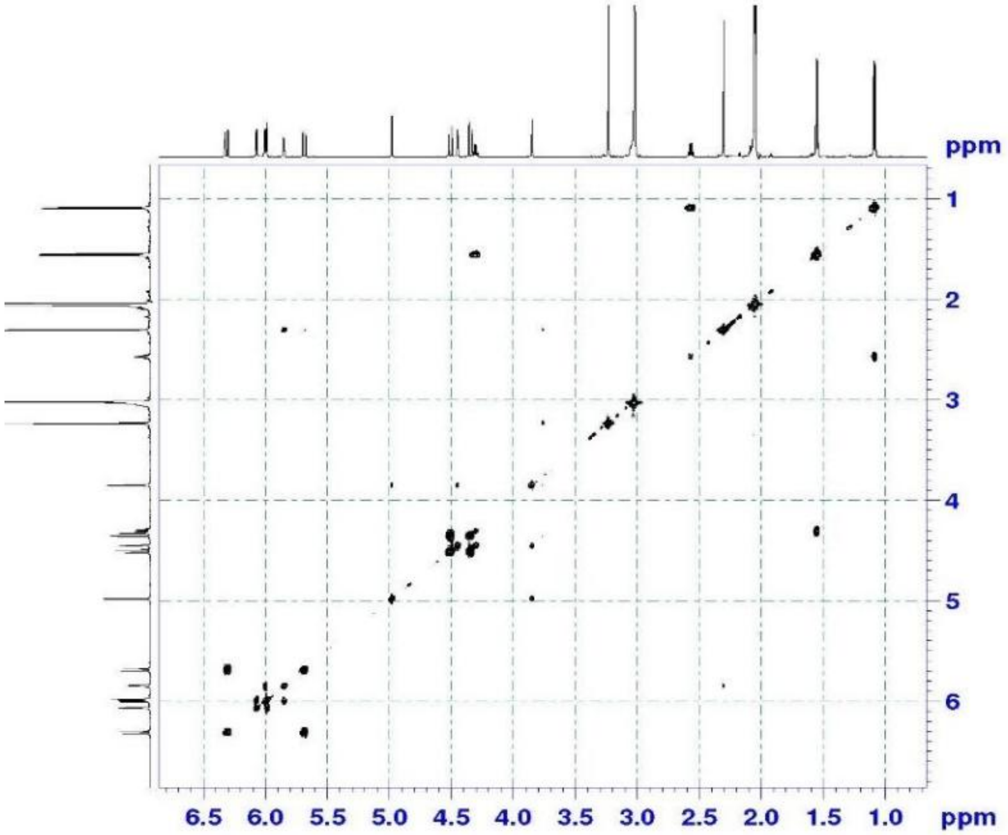

Fig. S117. ^{1}H-^{1}H COSY spectrum of curvamine P (**23**).

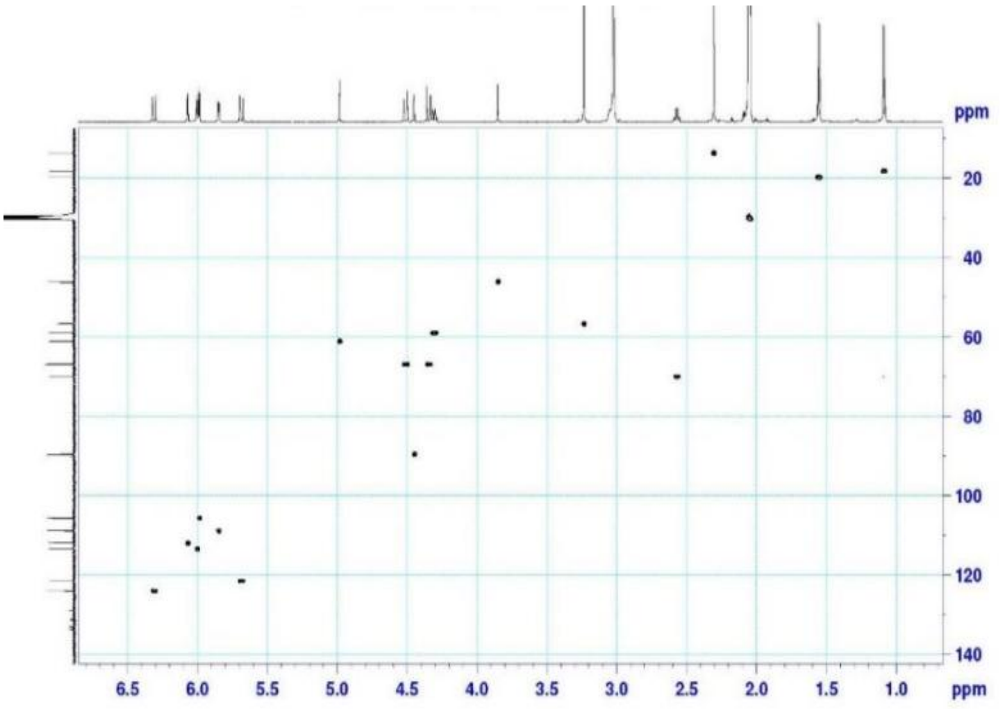

Fig. S118. HSQC spectrum of curvamine P (**23**).

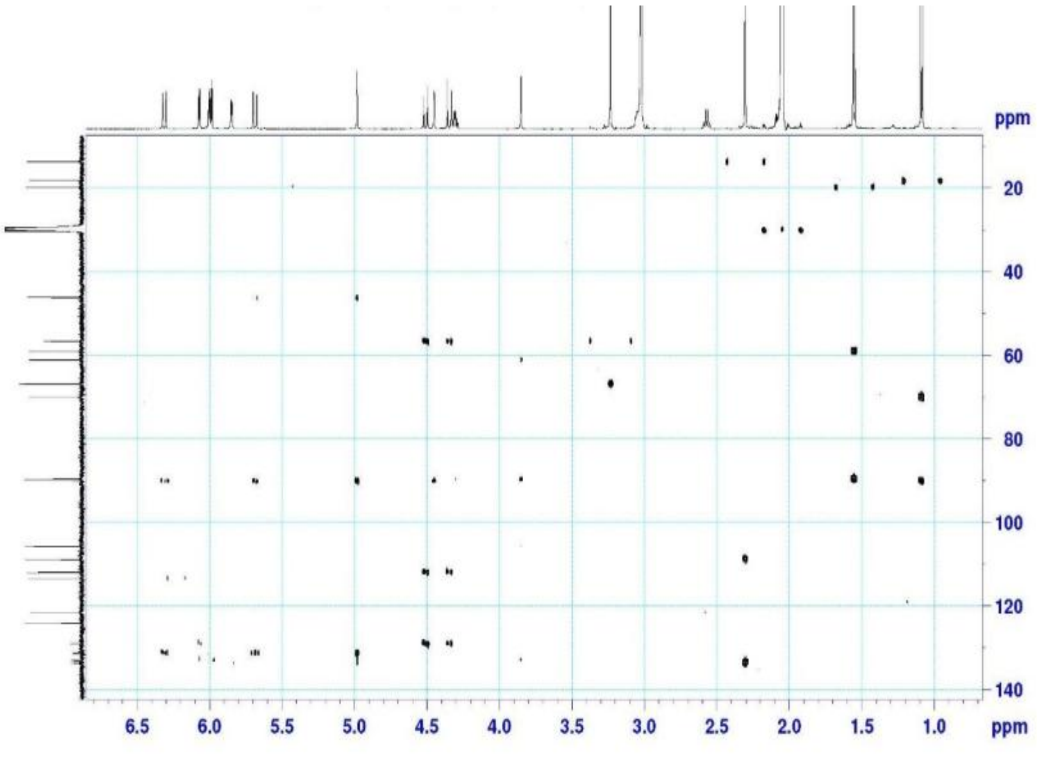

Fig. S119. HMBC spectrum of curvamine P (**23**).

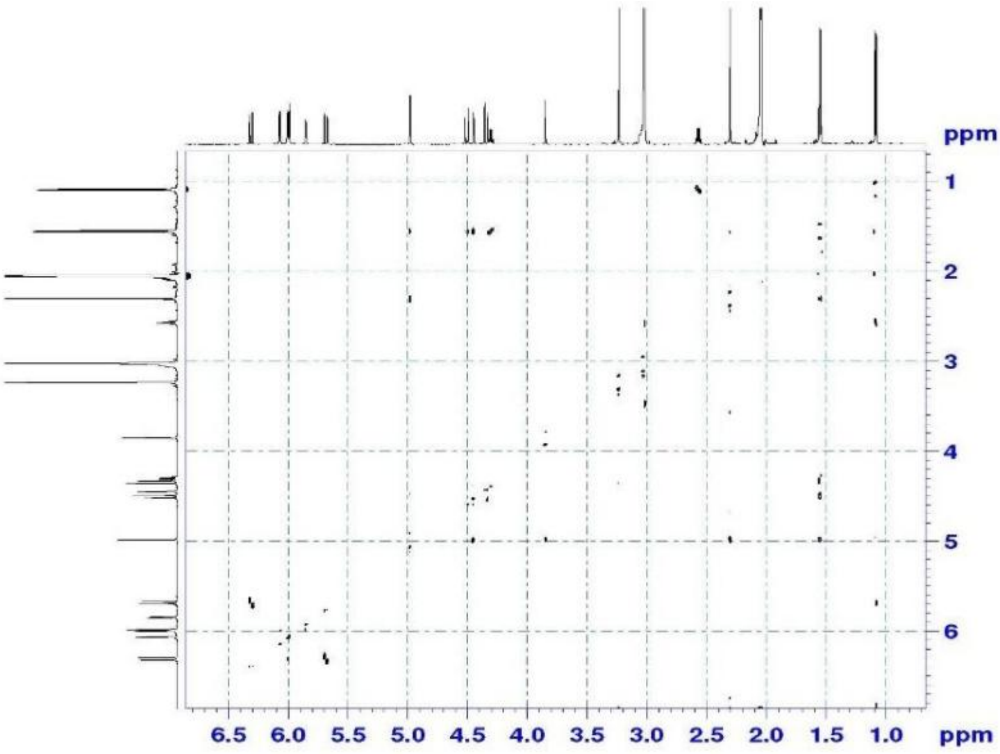

Fig. S120. NOESY spectrum of curvamine P (**23**).

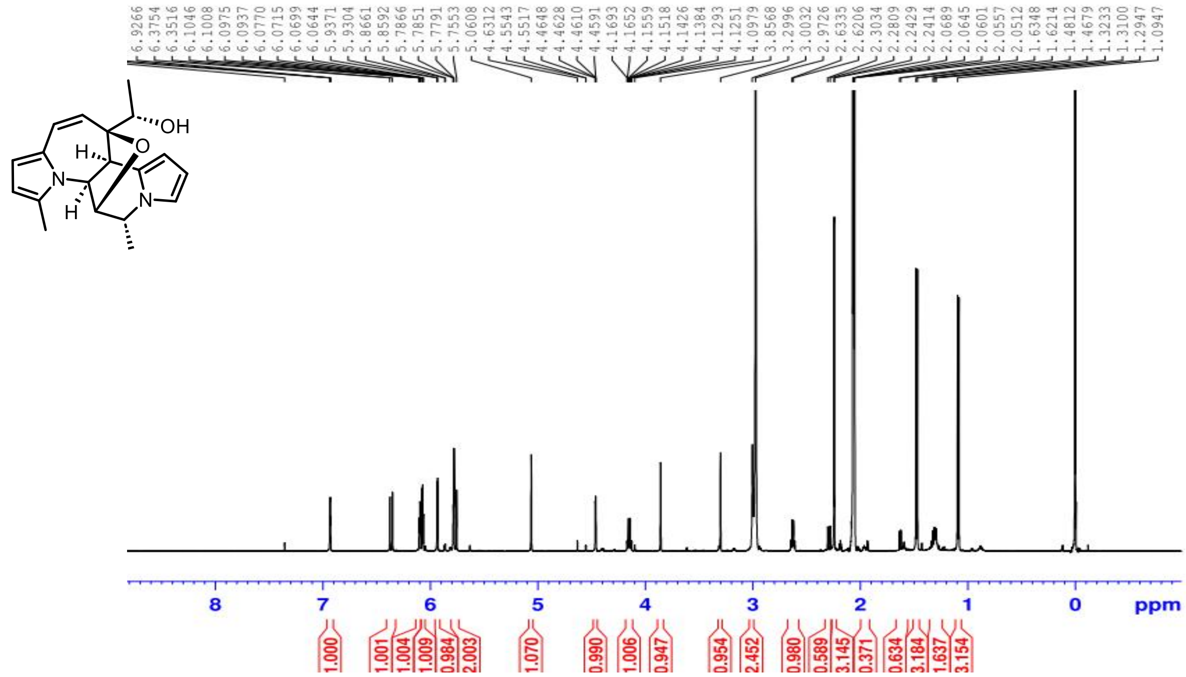

Fig. S121. ^{1}H NMR spectrum of curvamine Q (**24**) (400 MHz).

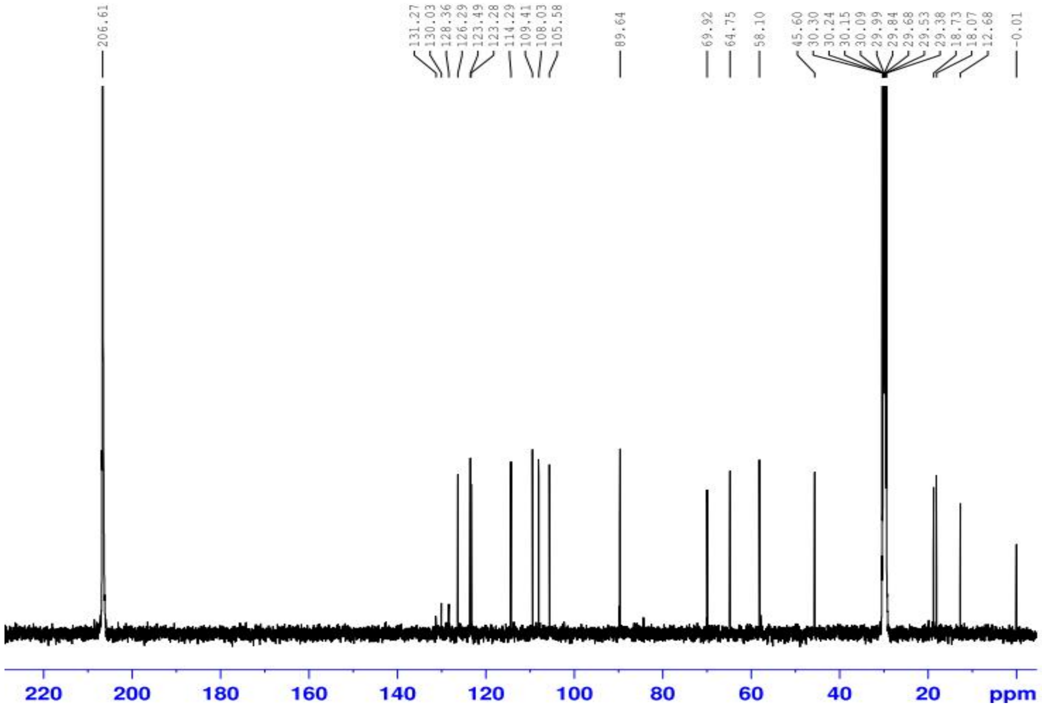

Fig. S122. ^{13}C NMR spectrum of curvamine Q (**24**) (100 MHz).

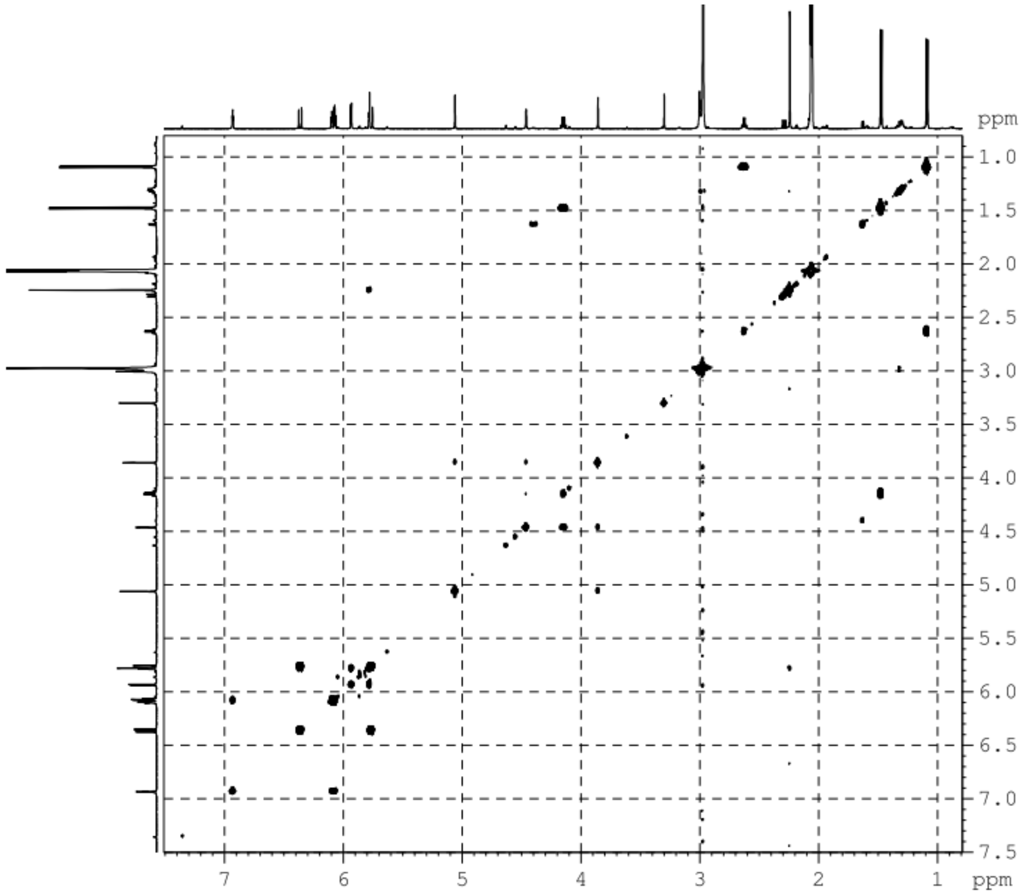

Fig. S123. ^{1}H-^{1}H COSY spectrum of curvamine Q (**24**).

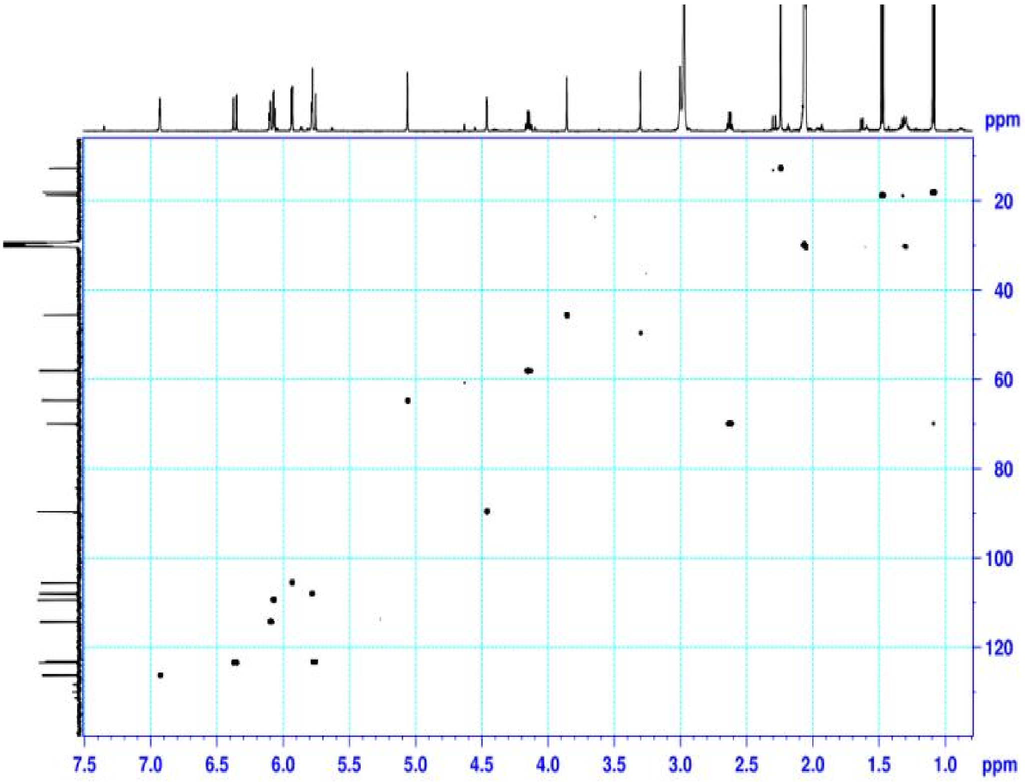

Fig. S124. HSQC spectrum of curvamine Q (**24**).

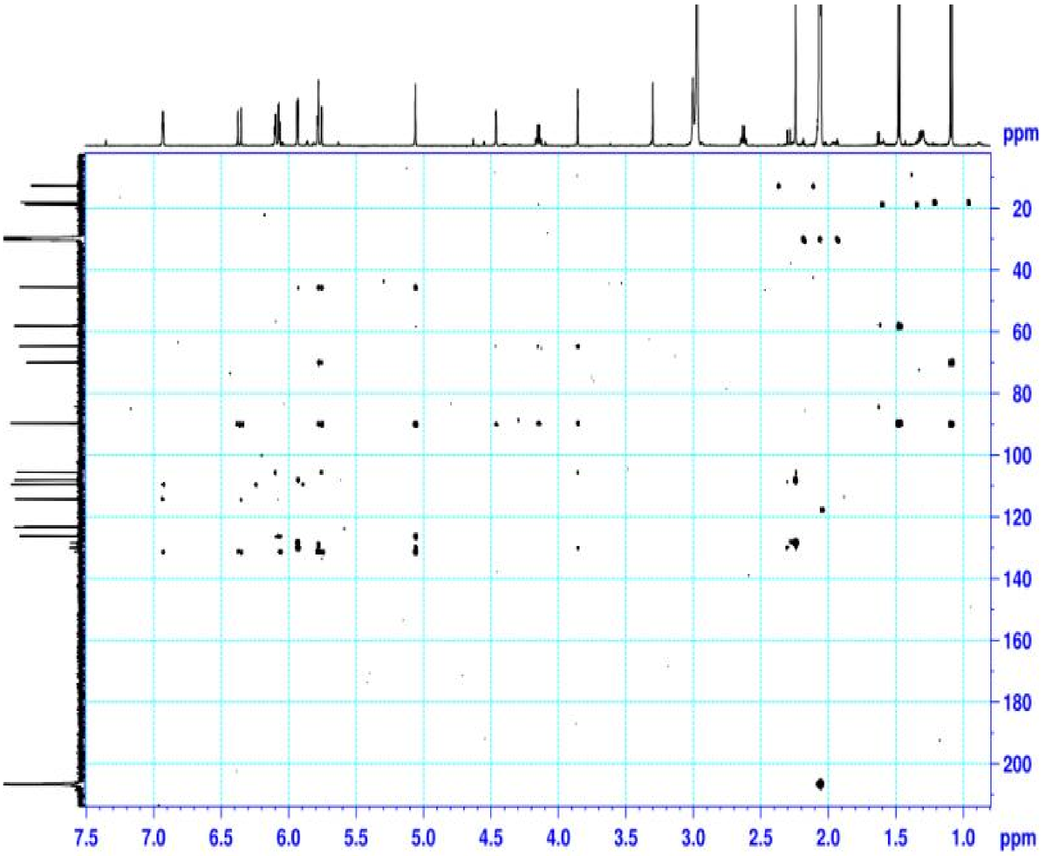

Fig. S125. HMBC spectrum of curvamine Q (**24**).

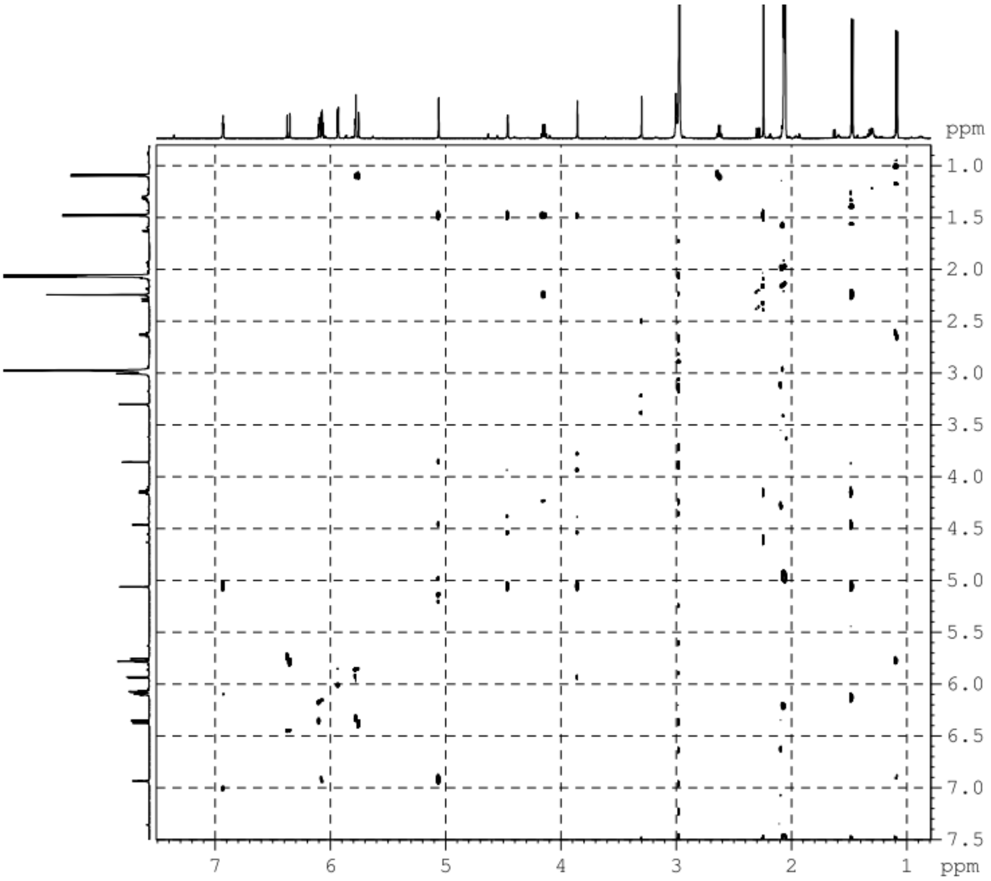

Fig. S126. NOESY spectrum of curvamine Q (**24**).

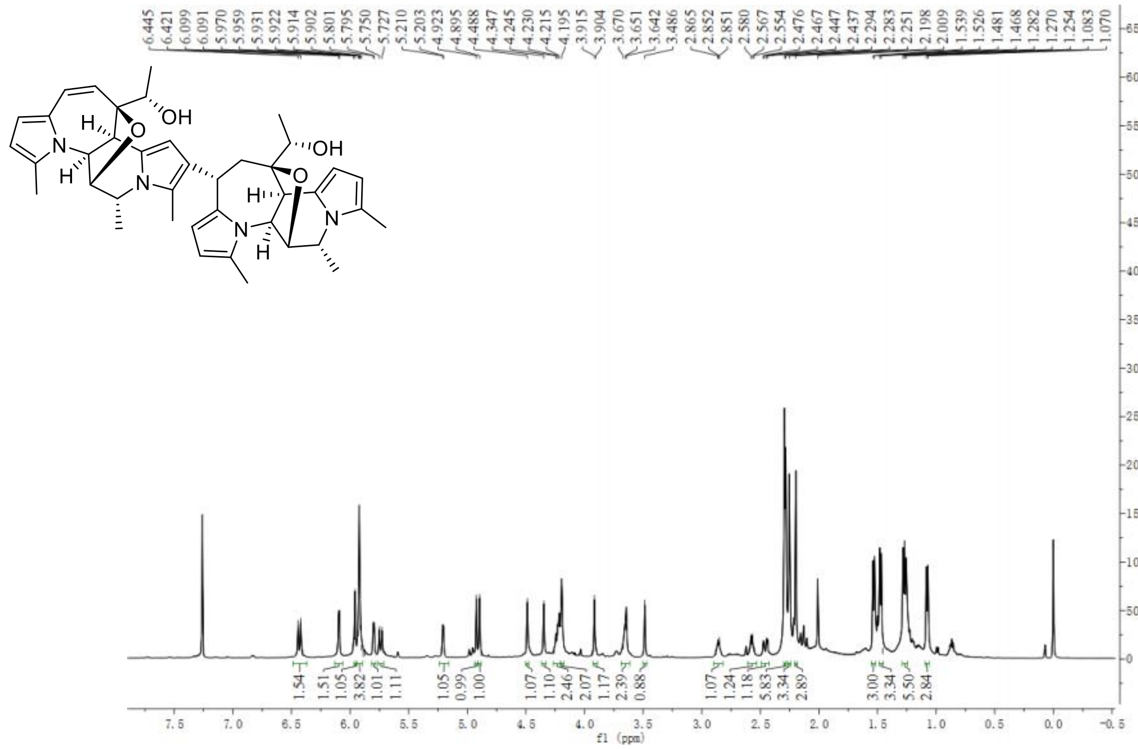

Fig. S127. ^{1}H NMR spectrum of curvamine R (**25**) (500 MHz, $CDCl_3$).

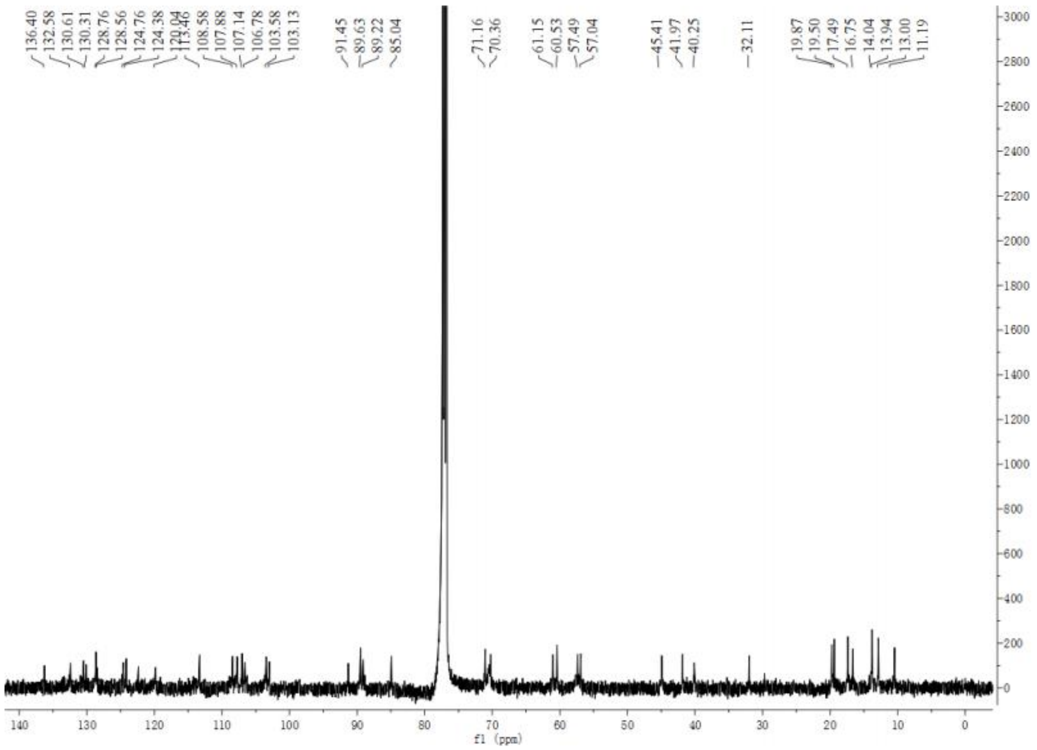

Fig. S128. ^{13}C NMR spectrum of curvamine R (**25**) (125 MHz, $CDCl_3$).

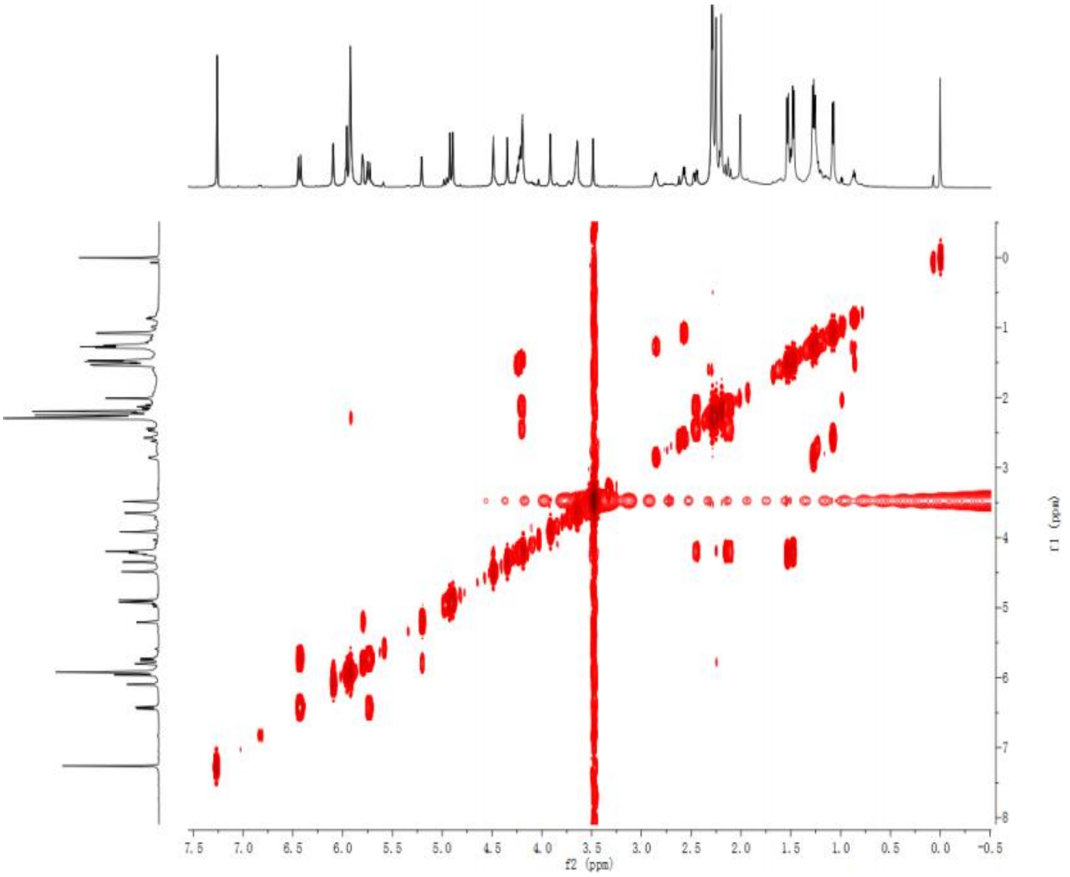

Fig. S129. ^{1}H-^{1}H COSY spectrum of curvamine R (**25**) in $CDCl_3$.

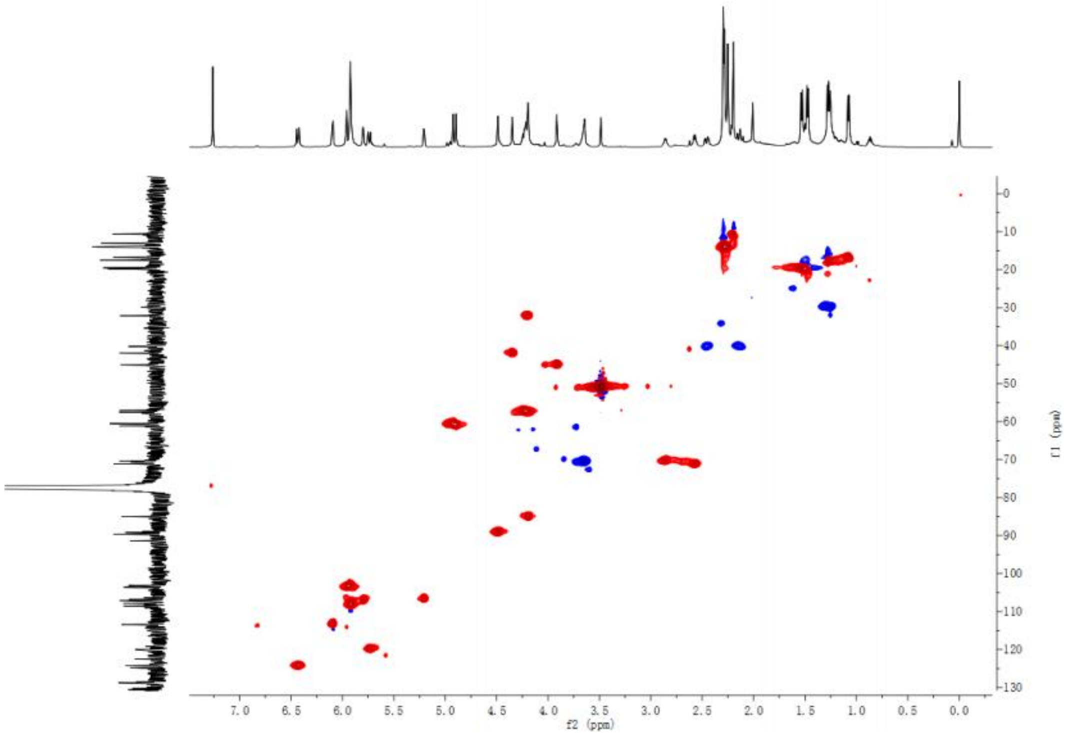

Fig. S130. HSQC spectrum of curvamine R (**25**) in $CDCl_3$.

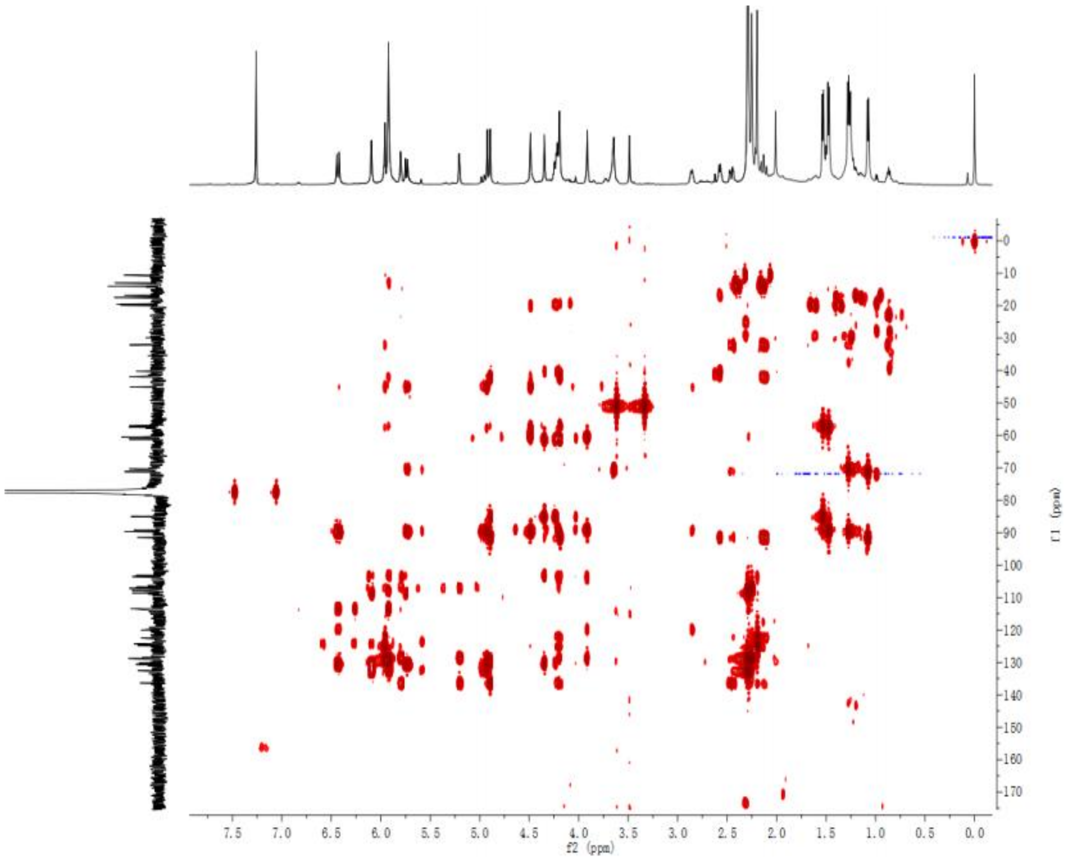

Fig. S131. HMBC spectrum of curvamine R (**25**) in $CDCl_3$.

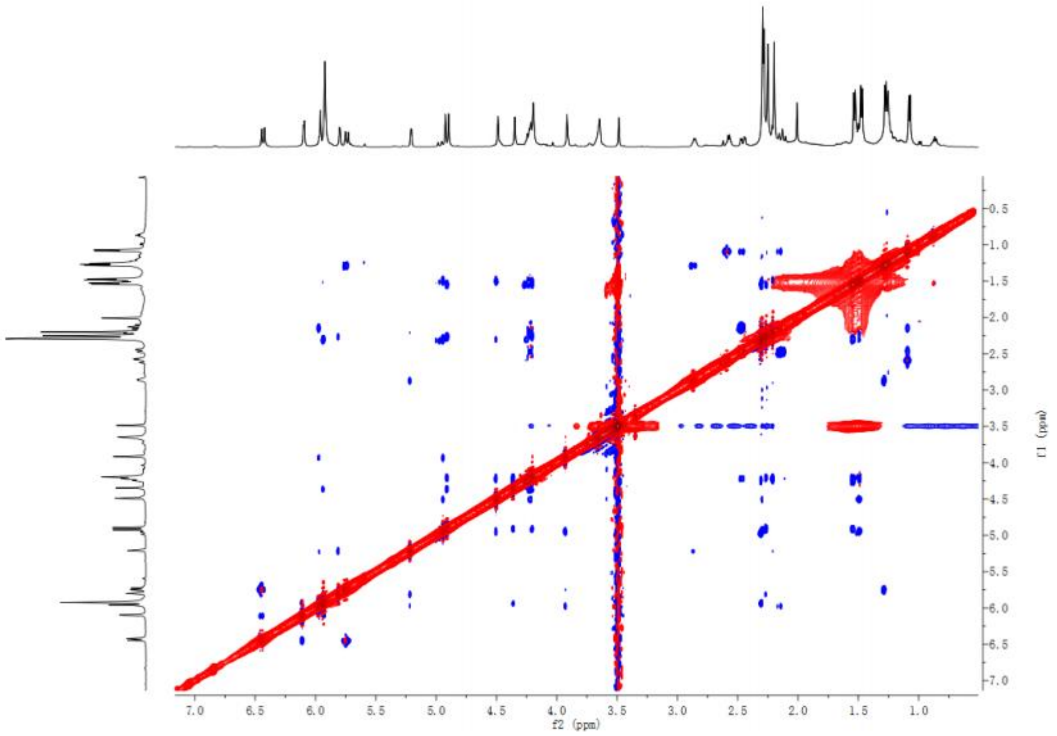

Fig. S132. NOESY spectrum of curvamine R (**25**) in $CDCl_3$.

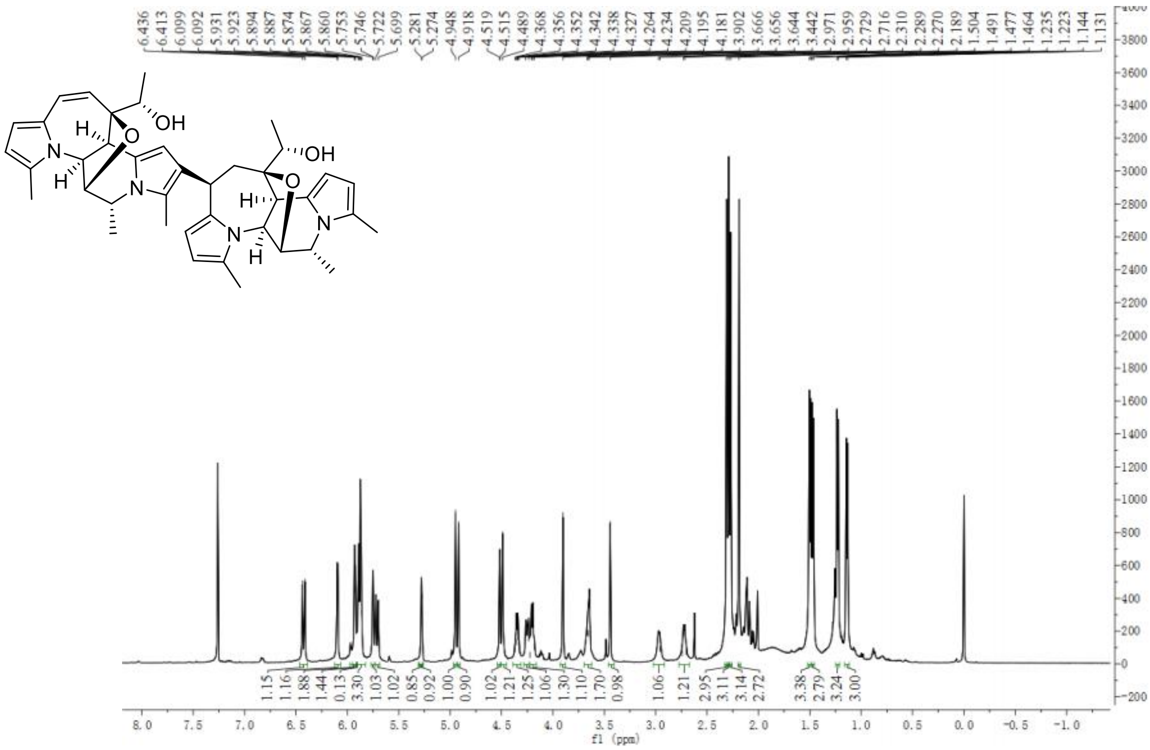

Fig. S133. ^{1}H NMR spectrum of curvamine S (**26**) (500 MHz, $CDCl_3$).

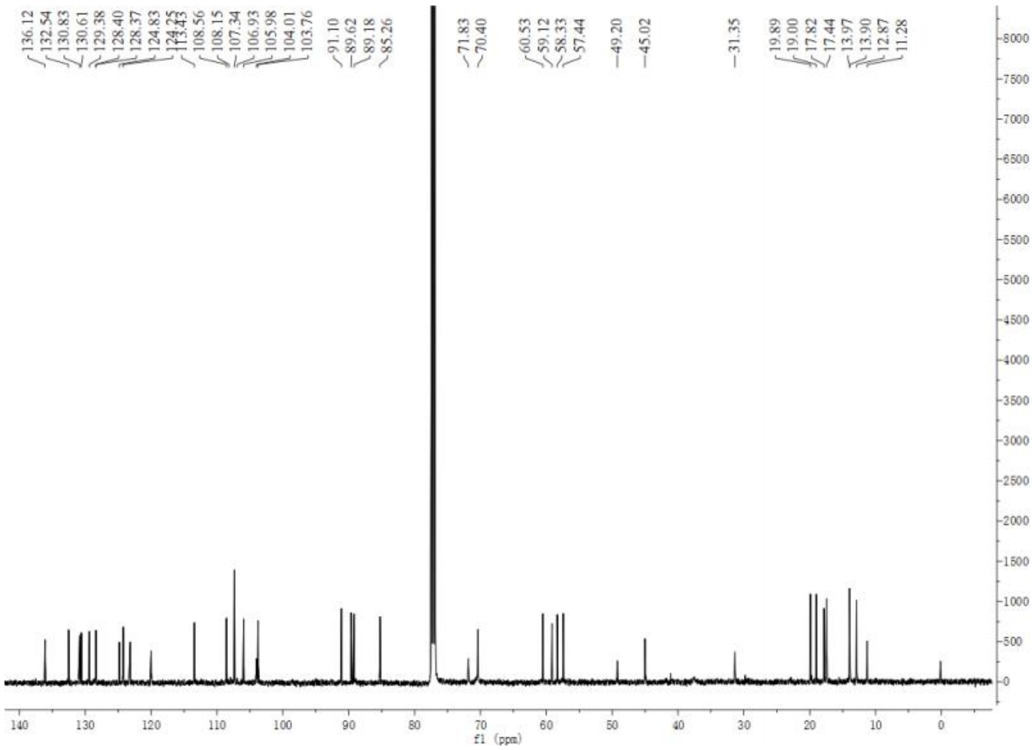

Fig. S134. ^{13}C NMR spectrum of curvamine S (**26**) (125 MHz, $CDCl_3$).

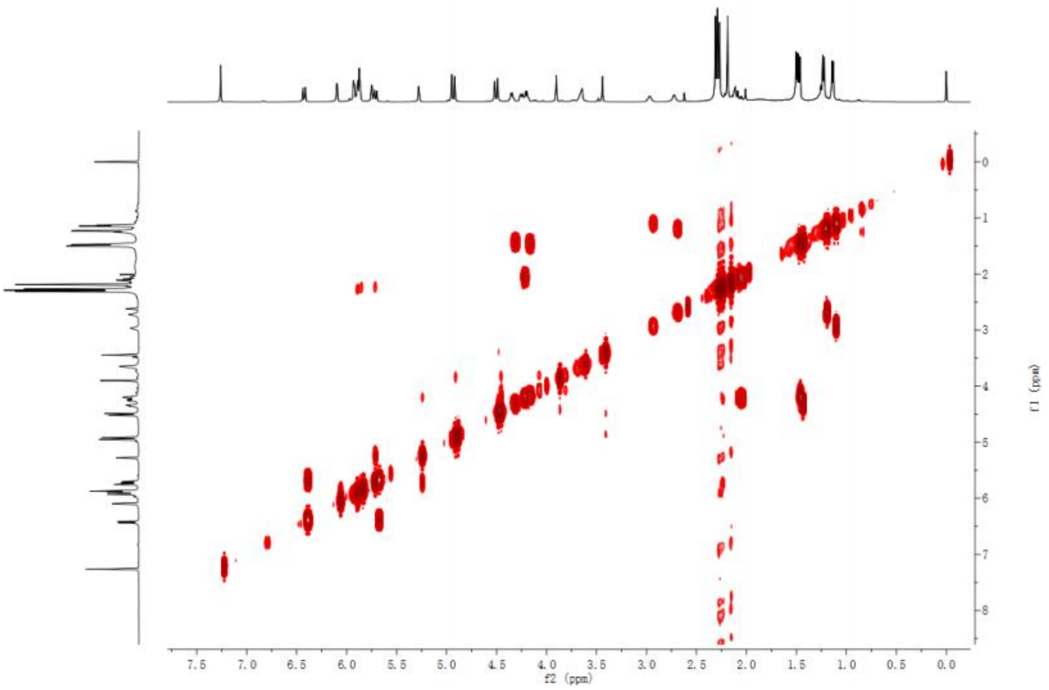

Fig. S135. ^1H-^1H COSY spectrum of curvamine S (**26**) in $CDCl_3$.

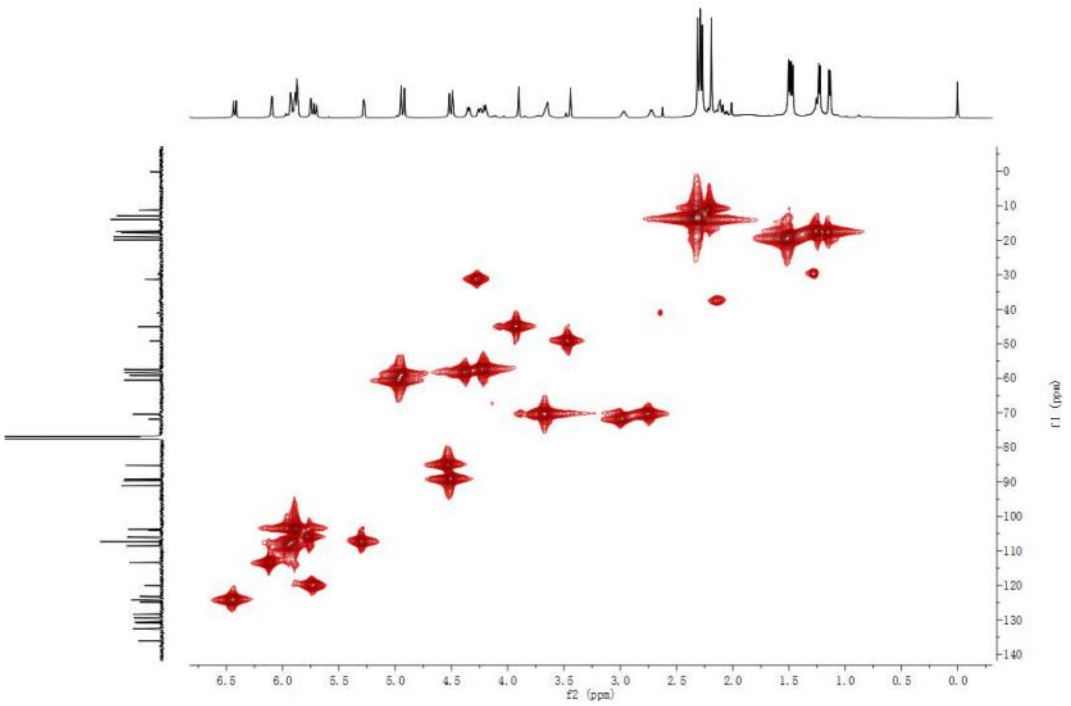

Fig. S136. HSQC spectrum of curvamine S (**26**) in $CDCl_3$.

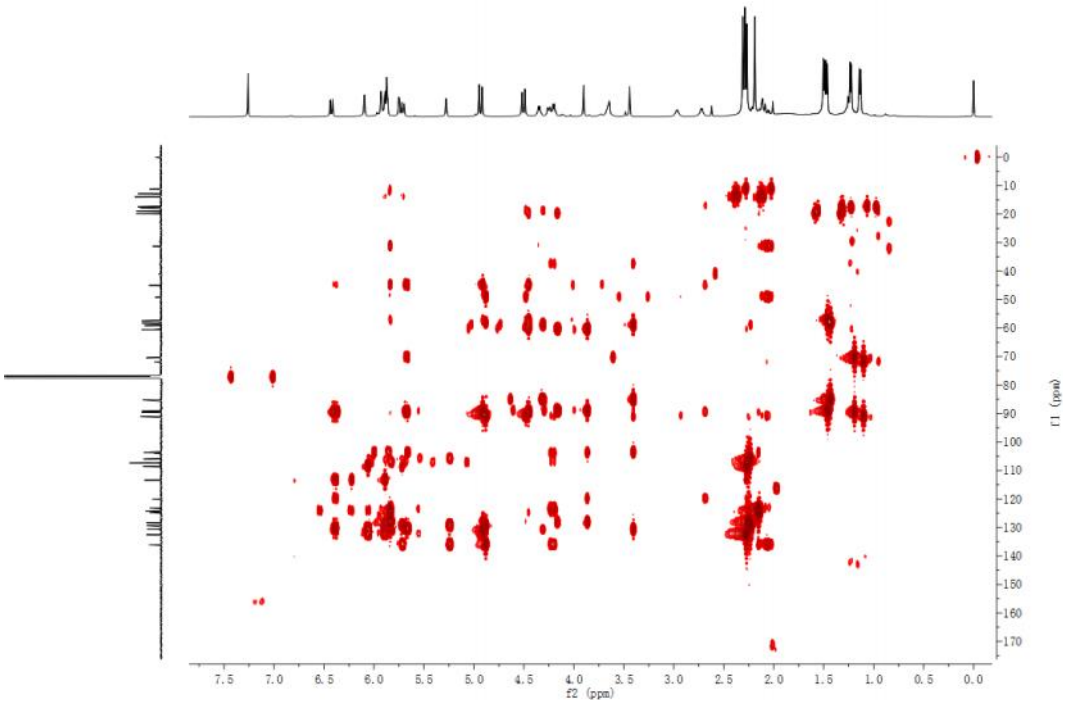

Fig. S137. HMBC spectrum of curvamine S (**26**) in $CDCl_3$.

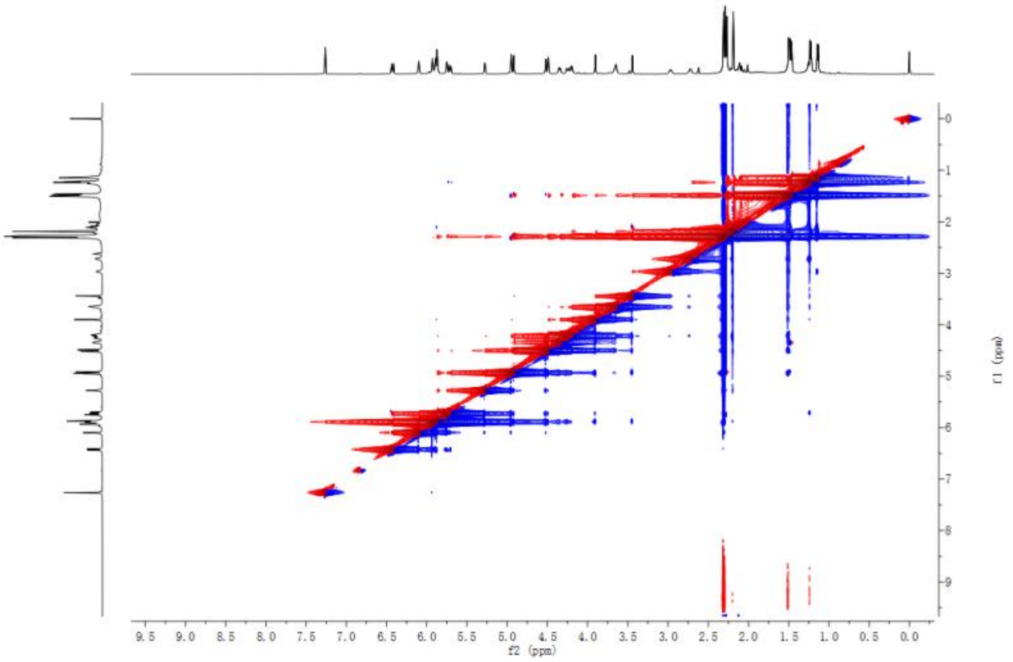

Fig. S138. NOESY spectrum of curvamine S (**26**) in $CDCl_3$.

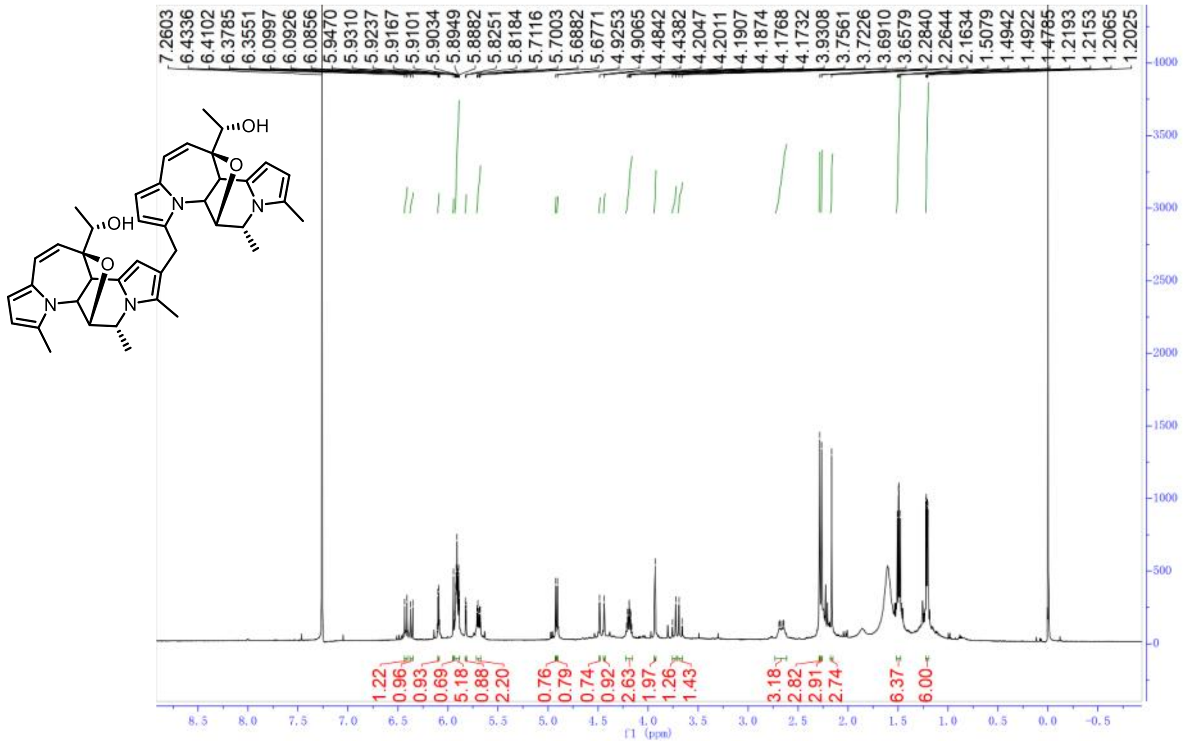

Fig. S139. ^{1}H NMR spectrum of curvamine T (**27**) (500 MHz, $CDCl_3$).

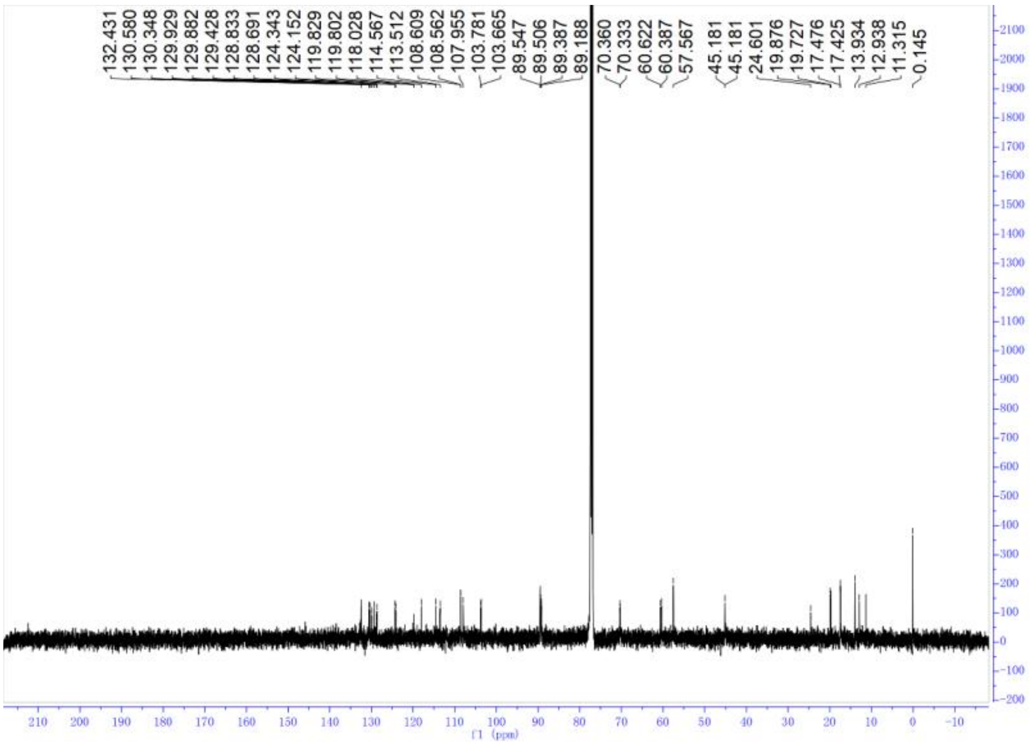

Fig. S140. ^{13}C NMR spectrum of curvamine T (**27**) (125 MHz, $CDCl_3$).
]

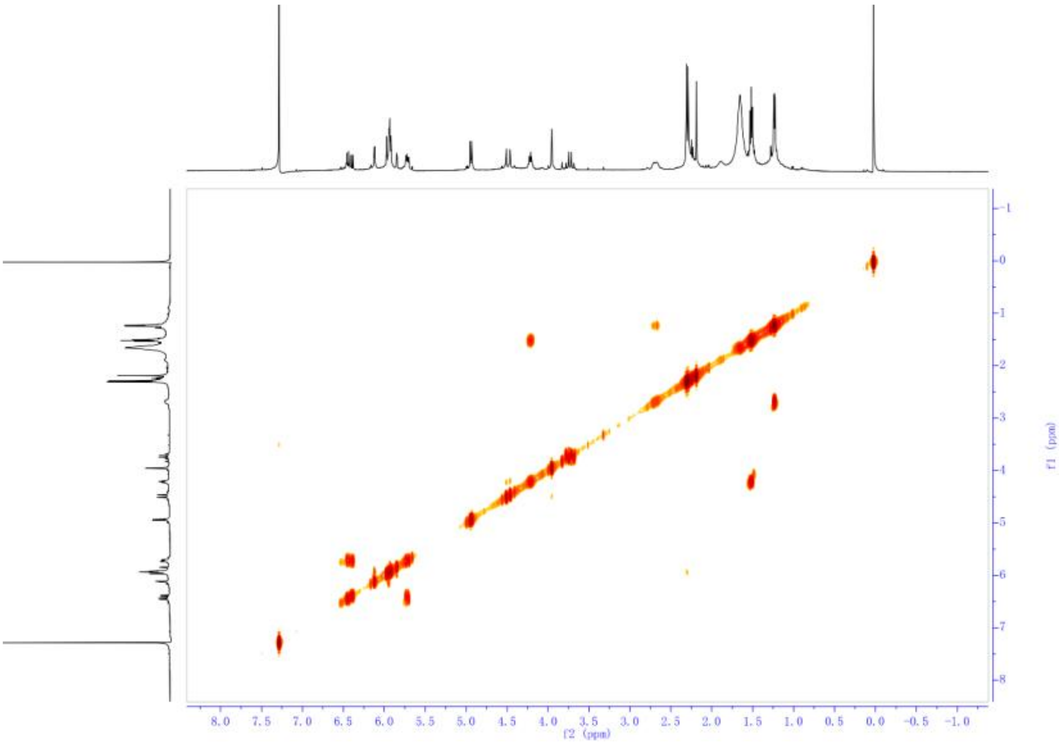

Fig. S141. ^{1}H-^{1}H COSY spectrum of curvamine T (**27**) in $CDCl_3$.

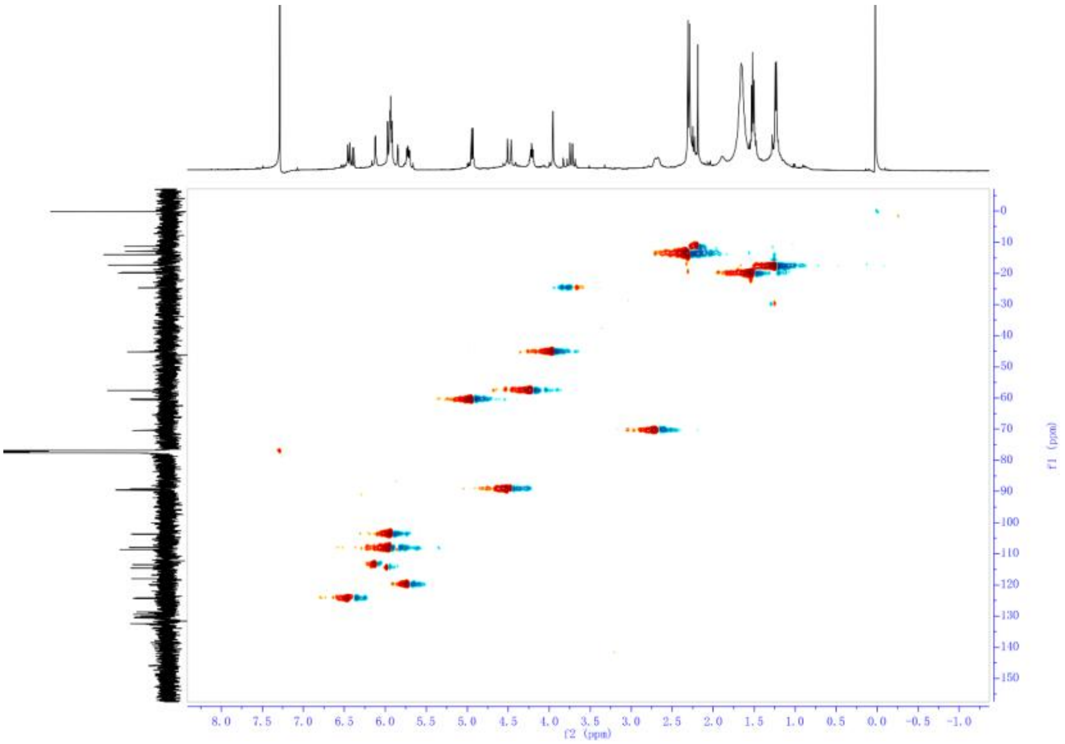

Fig. S142. HSQC spectrum of curvamine T (**27**) in $CDCl_3$.

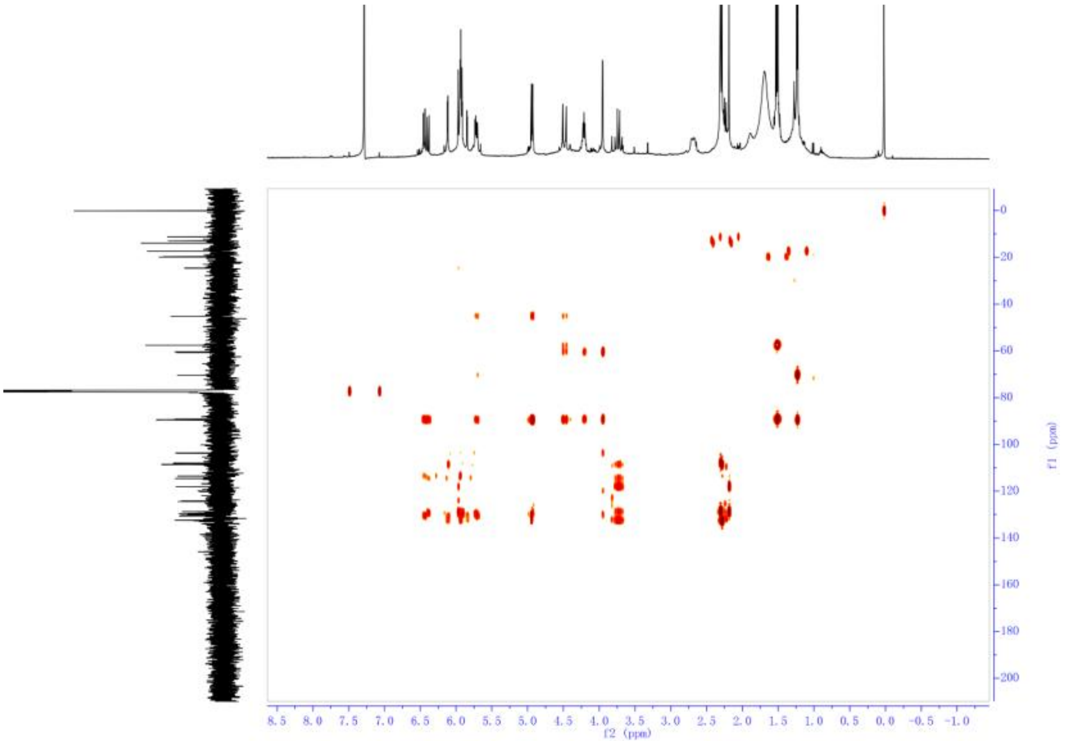

Fig. S143. HMBC spectrum of curvamine T (**27**) in $CDCl_3$.

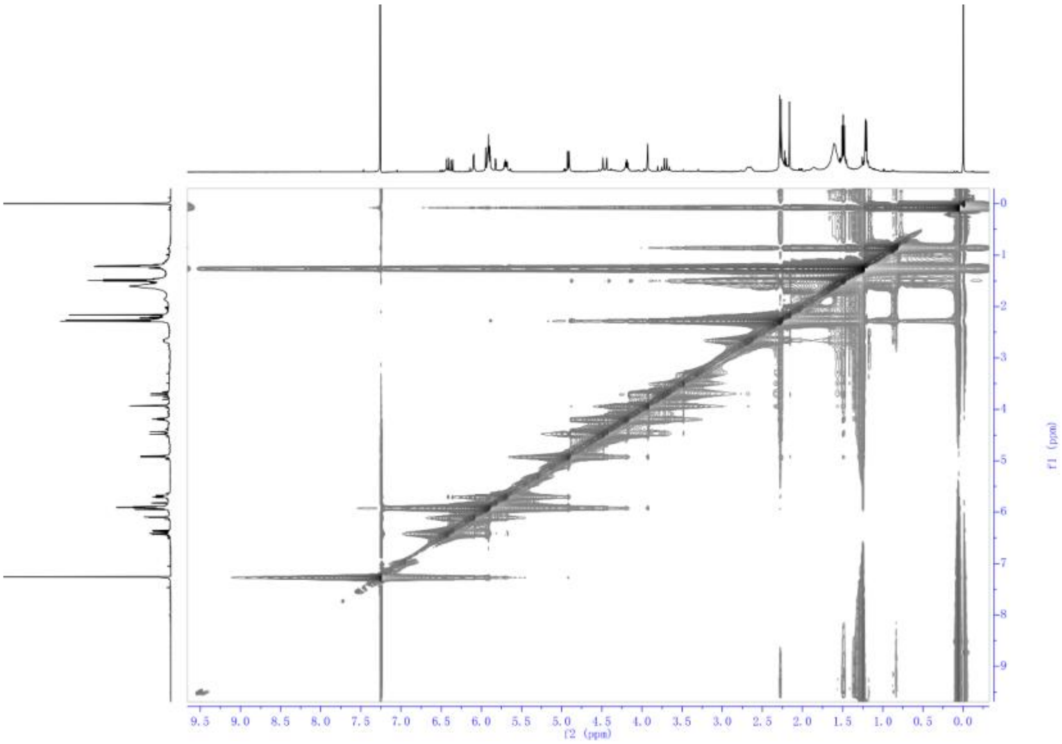

Fig. S144. NOESY spectrum of curvamine T (**27**) in $CDCl_3$.

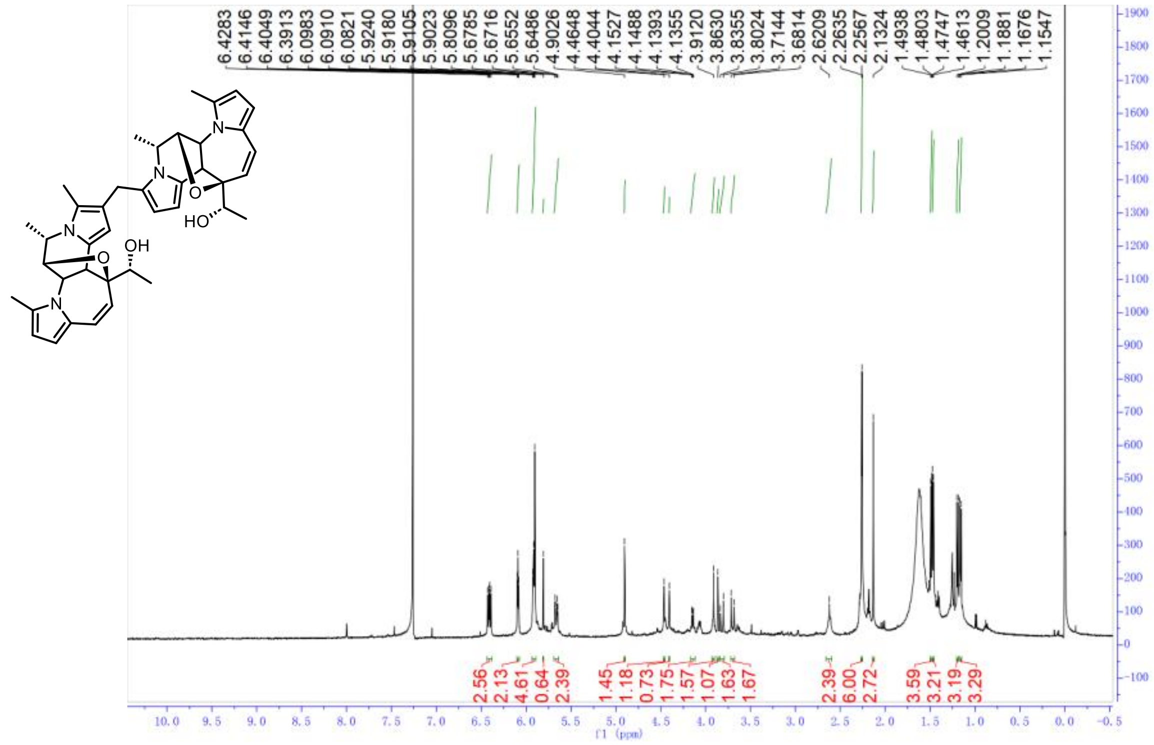

Fig. S145. ^1H NMR spectrum of curvamine U (**28**) (500 MHz, $CDCl_3$).

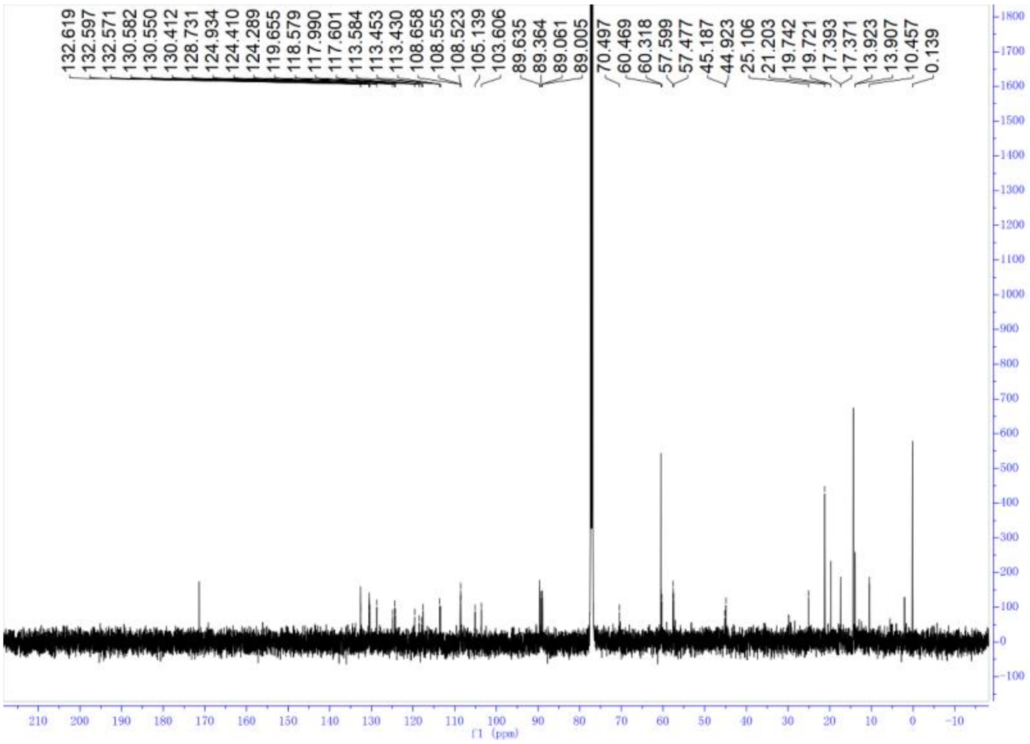

Fig. S146. ^{13}C NMR spectrum of curvamine U (**28**) (125 MHz, $CDCl_3$).

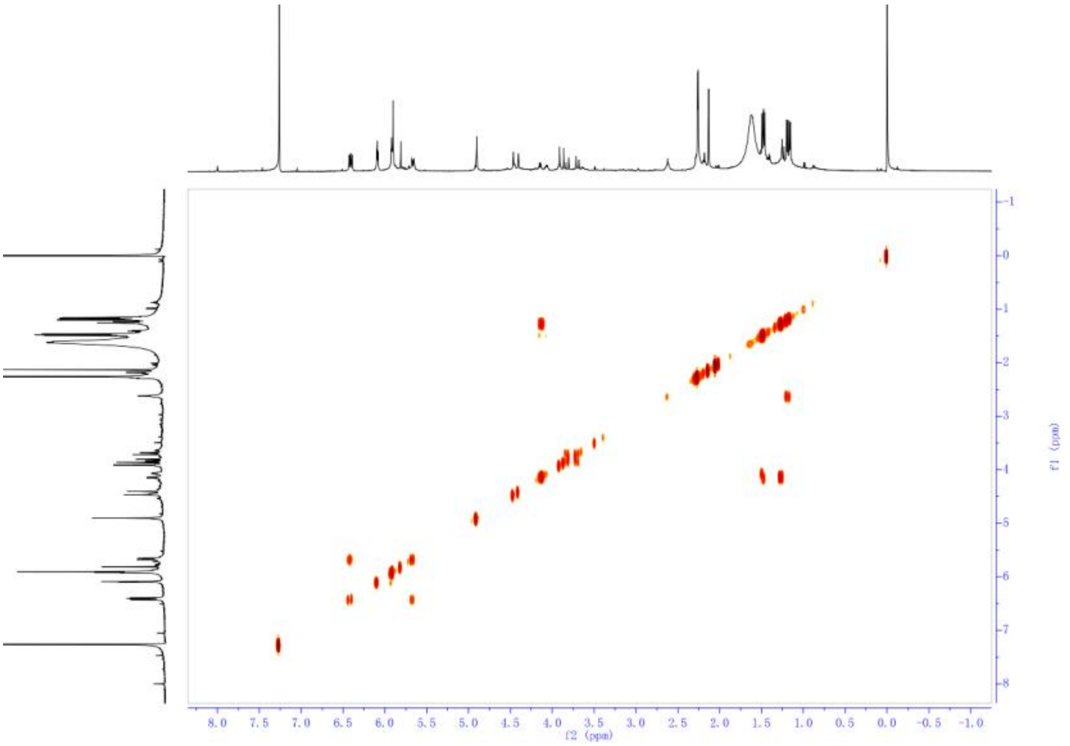

Fig. S147. ^{1}H-^{1}H COSY spectrum of curvamine U (**28**) in $CDCl_3$.

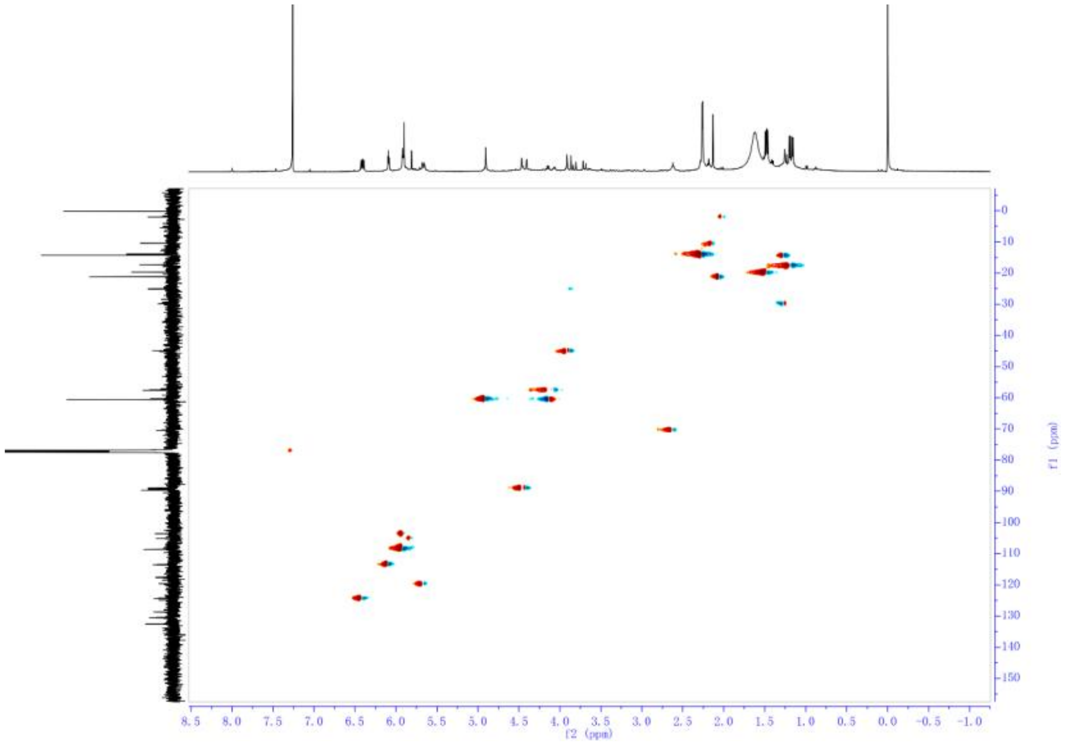

Fig. S148. HSQC spectrum of curvamine U (**28**) in $CDCl_3$.

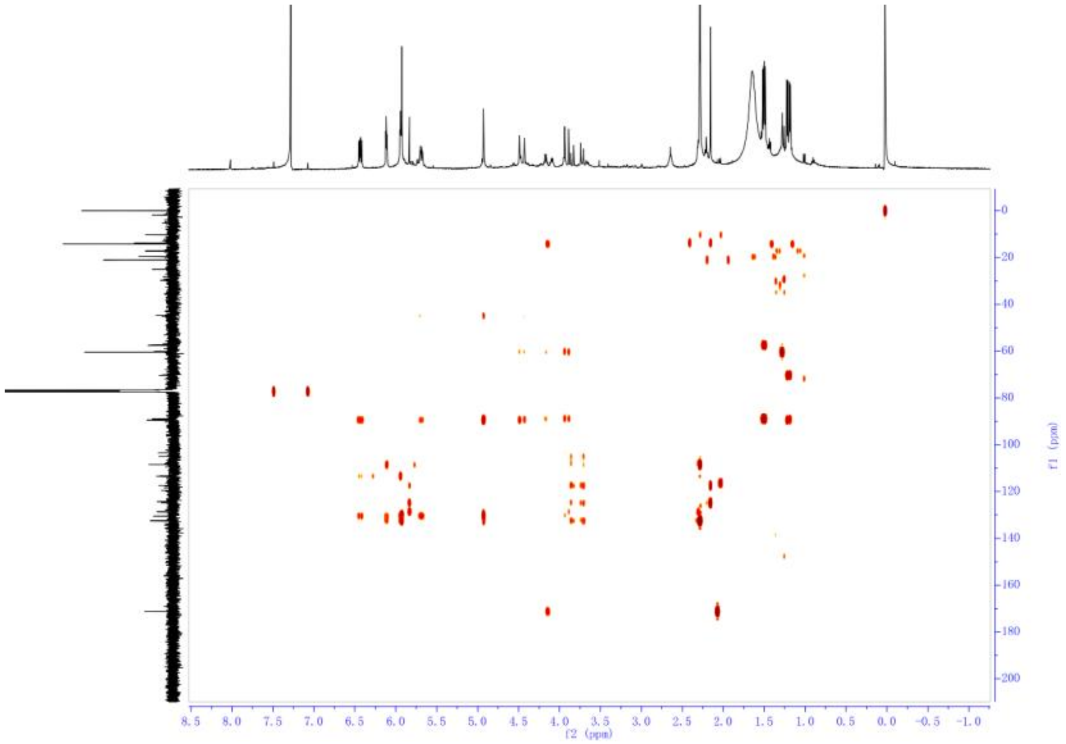

Fig. S149. HMBC spectrum of curvamine U (**28**) in $CDCl_3$.

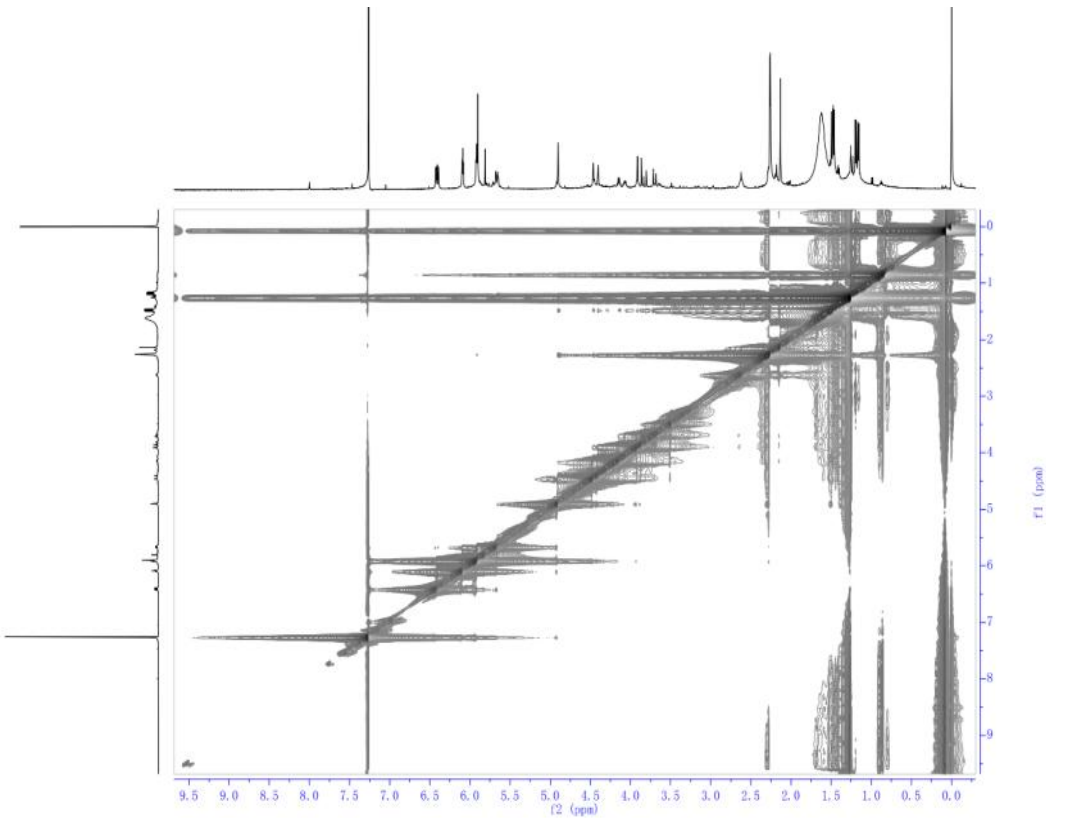

Fig. S150. NOESY spectrum of curvamine U (**28**) in $CDCl_3$.

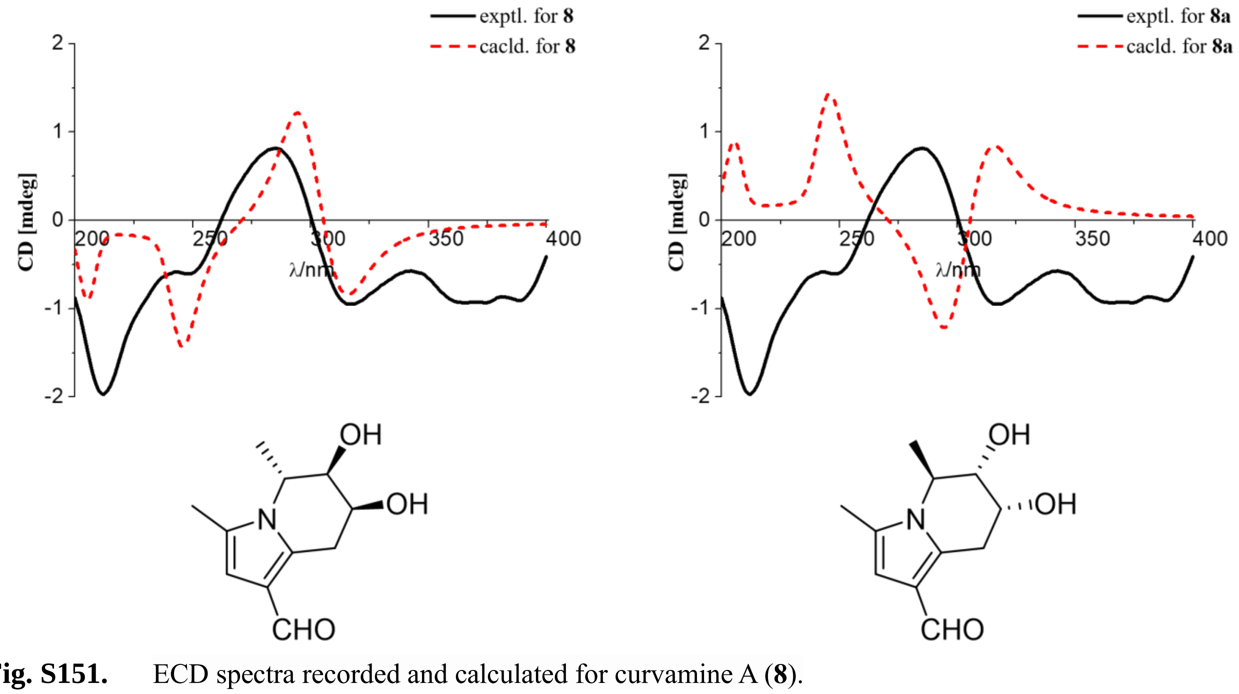

Fig. S151. ECD spectra recorded and calculated for curvamine A (**8**).

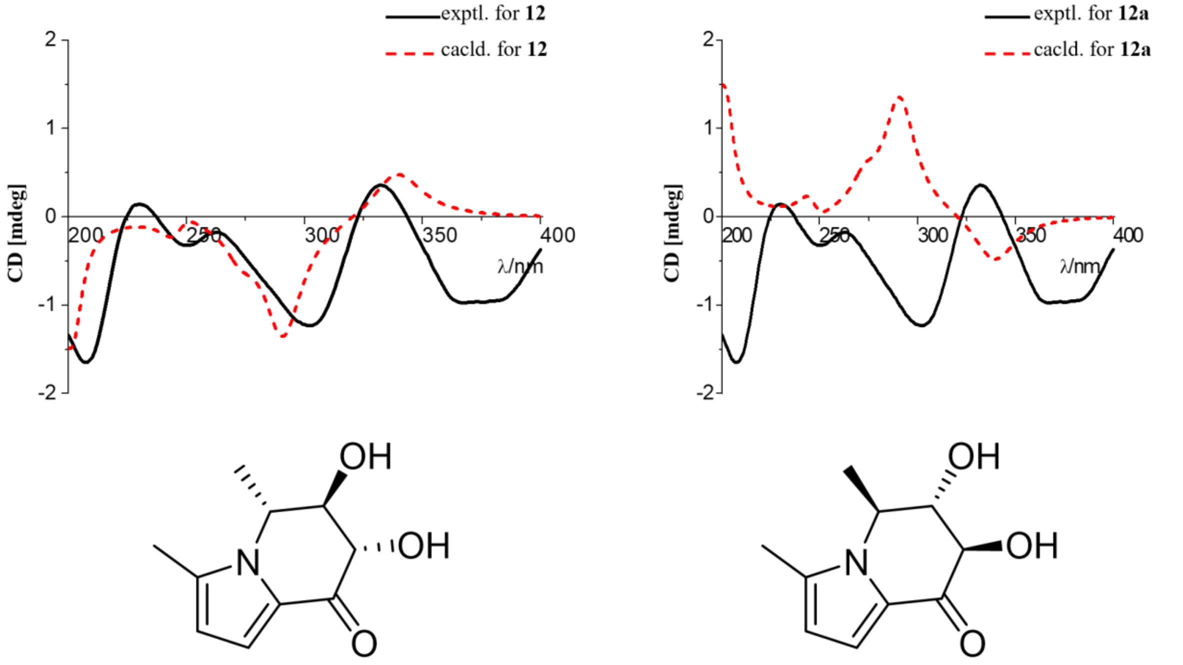

Fig. S152. ECD spectra recorded and calculated for curvamine E (**12**).

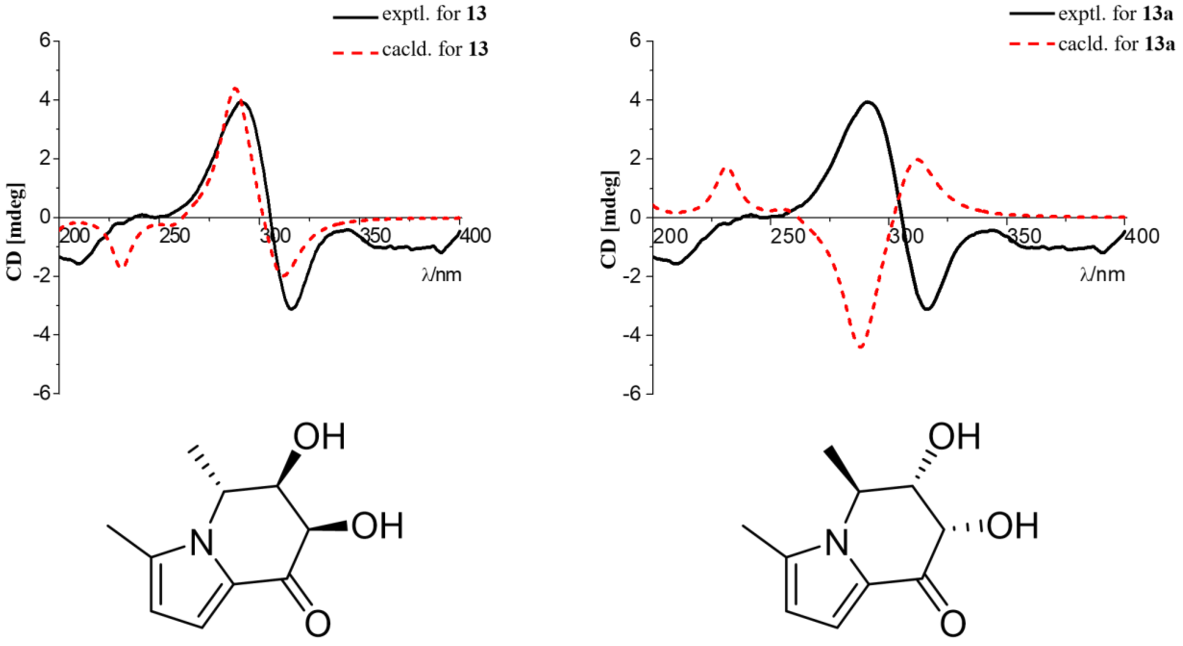

Fig. S153. ECD spectra recorded and calculated for curvamine F (**13**).

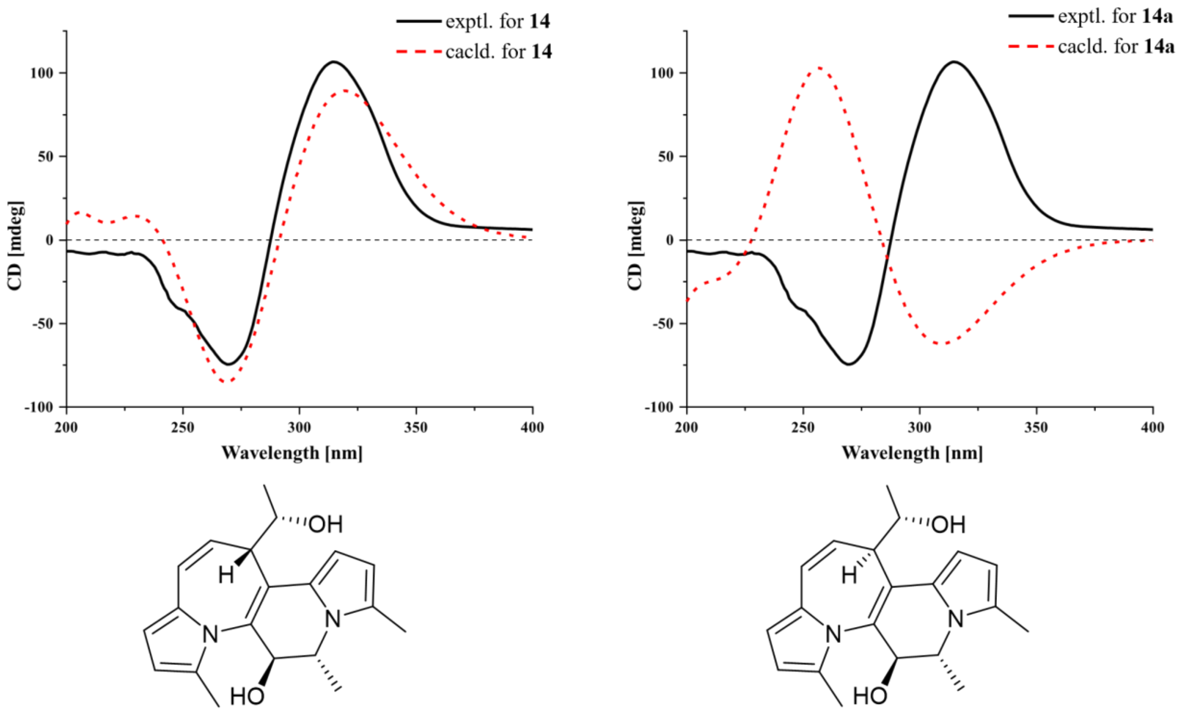

Fig. S154. ECD spectra recorded and calculated for curvamine G (**14**).

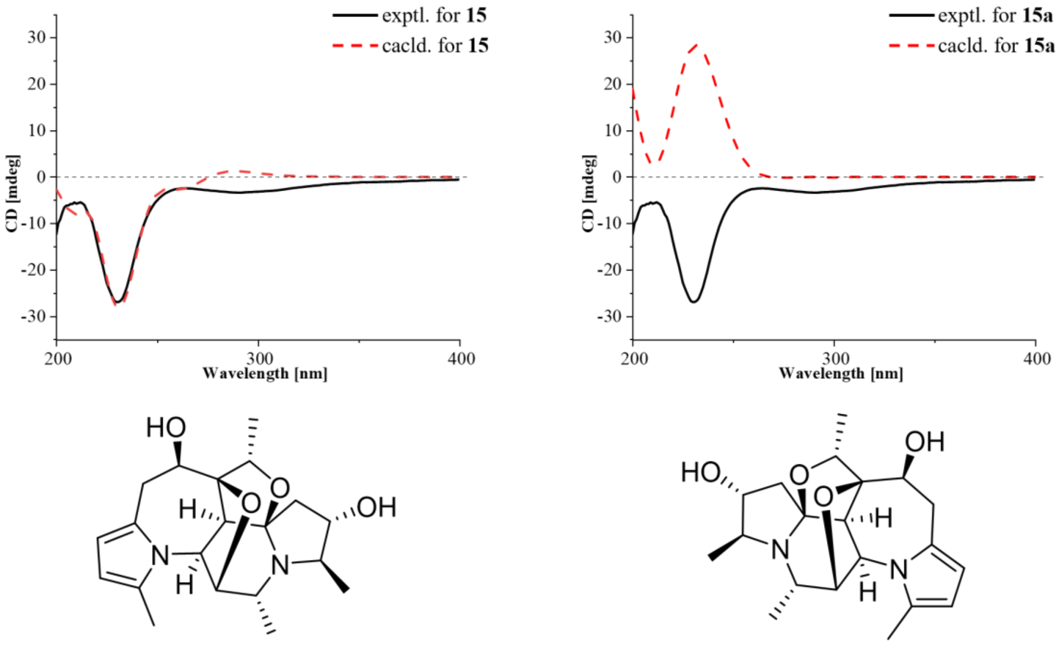

Fig. S155. ECD spectra recorded and calculated for curvamine H (**15**).

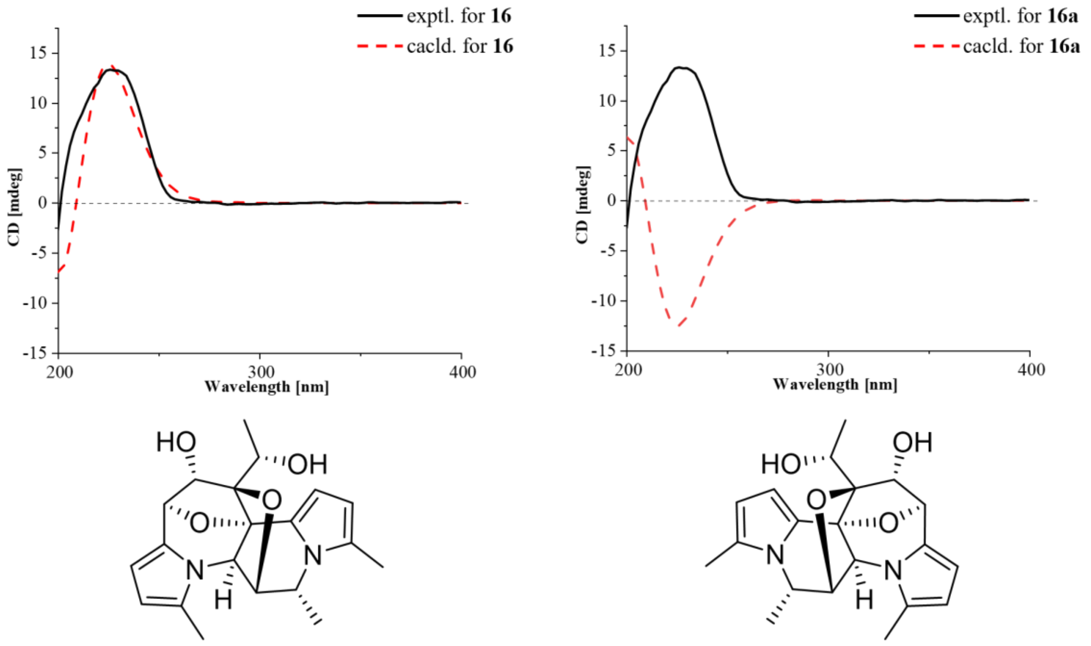

Fig. S156. ECD spectra recorded and calculated for curvamine I (**16**).

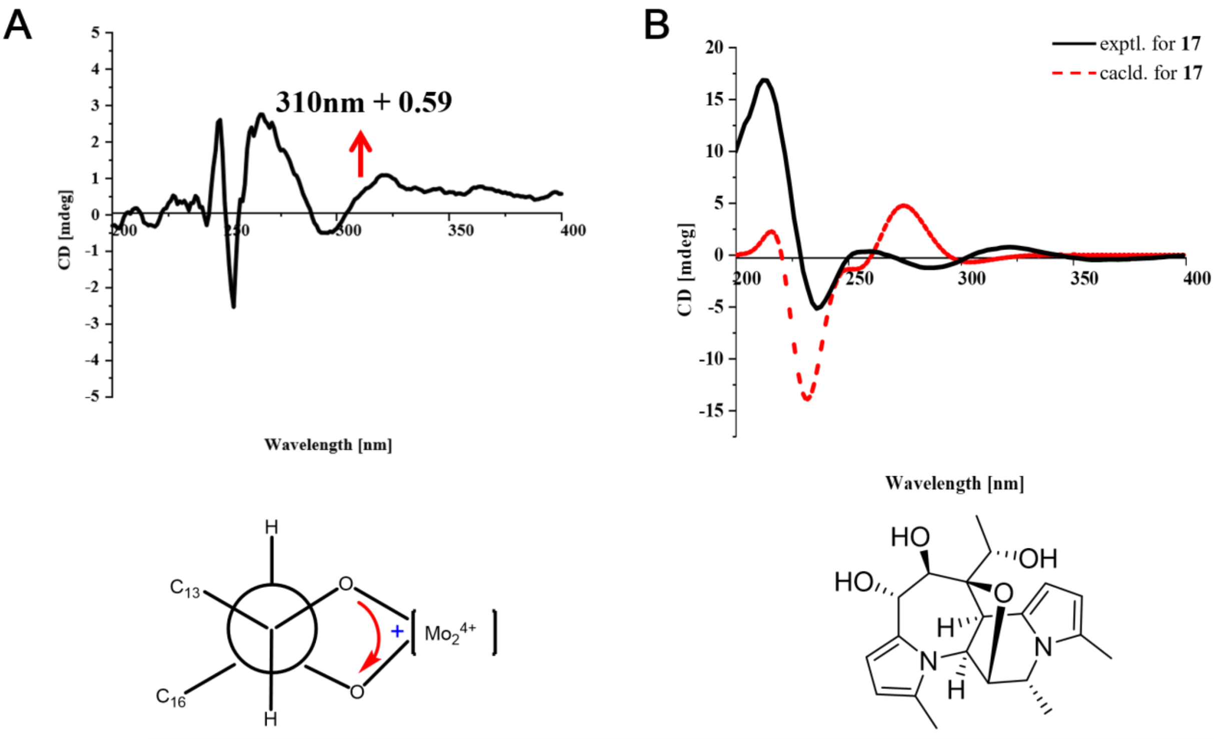

Fig. S157. ECD spectral analysis of curvamine J (**17**). (A) The net induced circular dichroism (ICD) spectrum of **17** after its complexion with Mo_2^{4+}; (B) ECD spectra recorded and calculated for **17**.

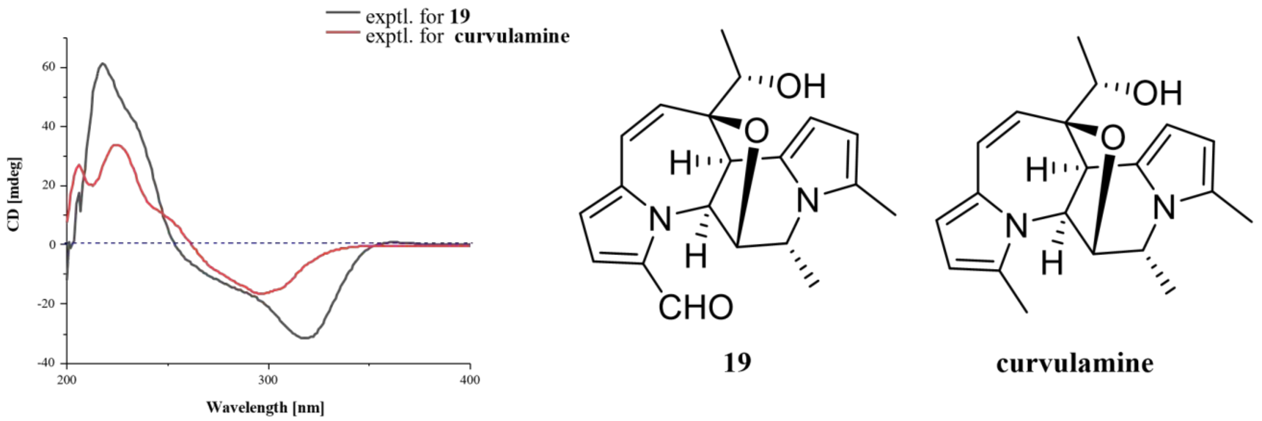

Fig. S158. ECD spectral comparison between curvamine L (**19**) and curvulamine.

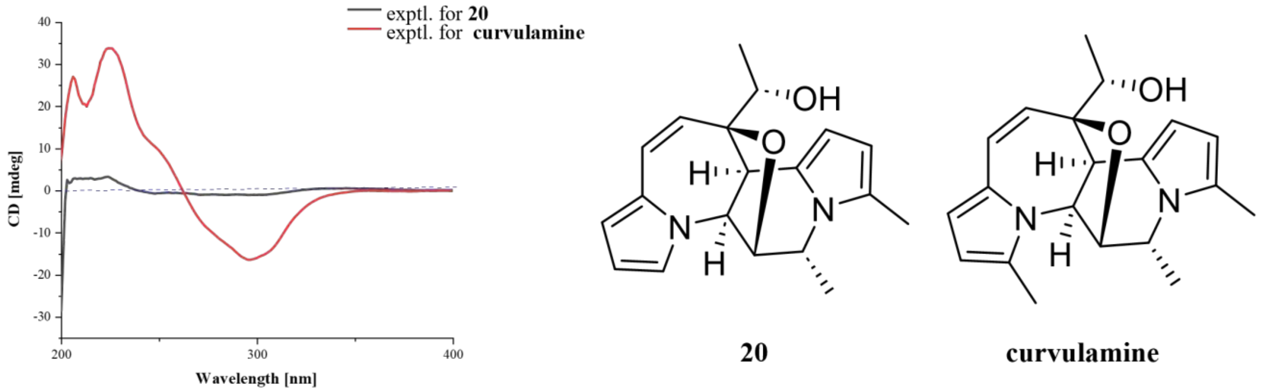

Fig. S159. ECD spectral comparison between curvamine M (**20**) and curvulamine.

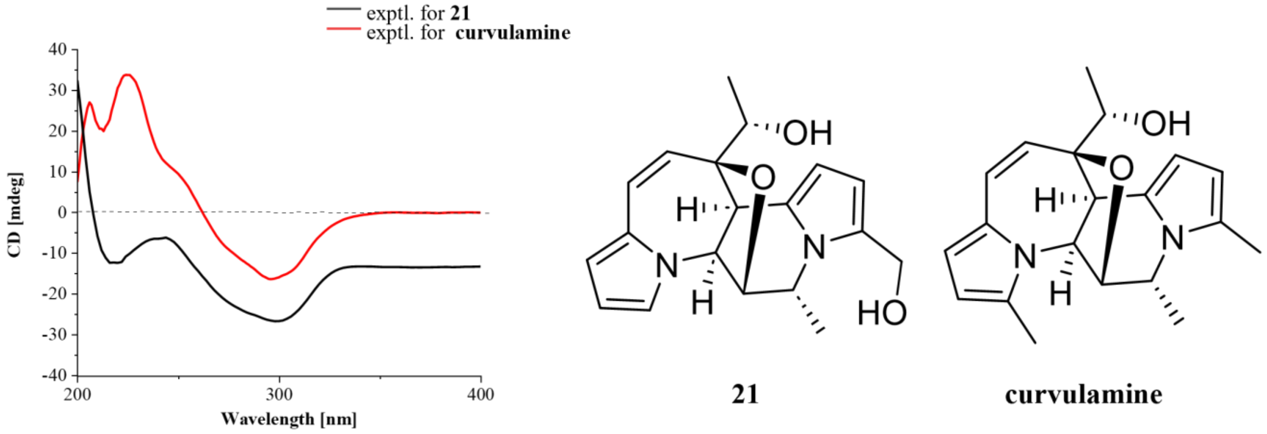

Fig. S160. ECD spectral comparison between curvamine N (**21**) and curvulamine.

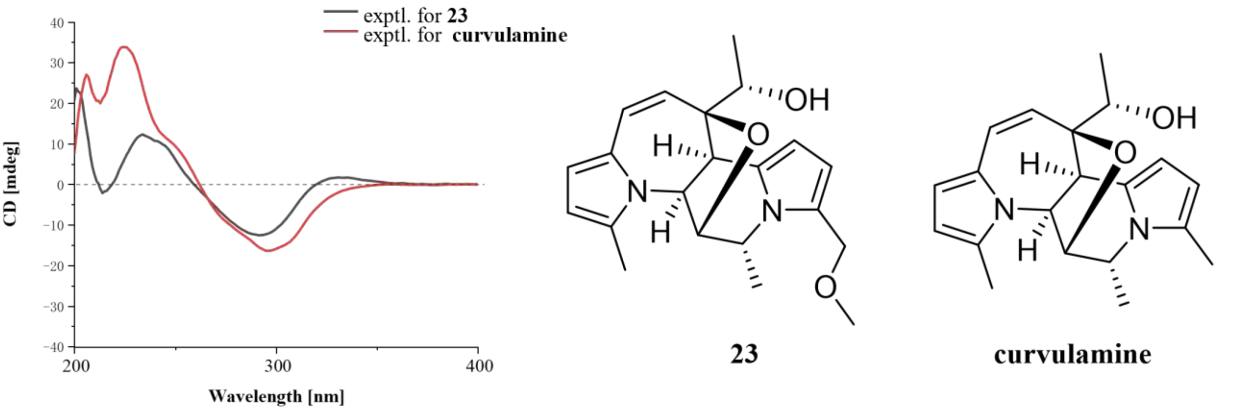

Fig. S161. ECD spectral comparison between curvamine P (**23**) and curvulamine.

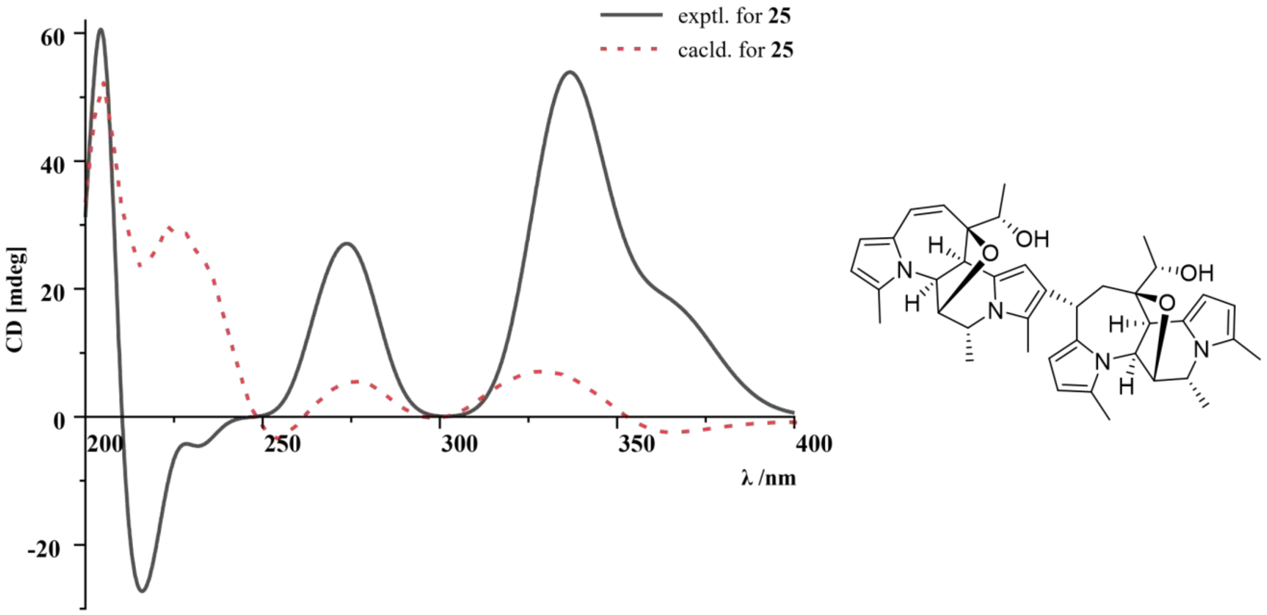

Fig. S162. ECD spectra recorded and calculated for curvamine R (**25**).

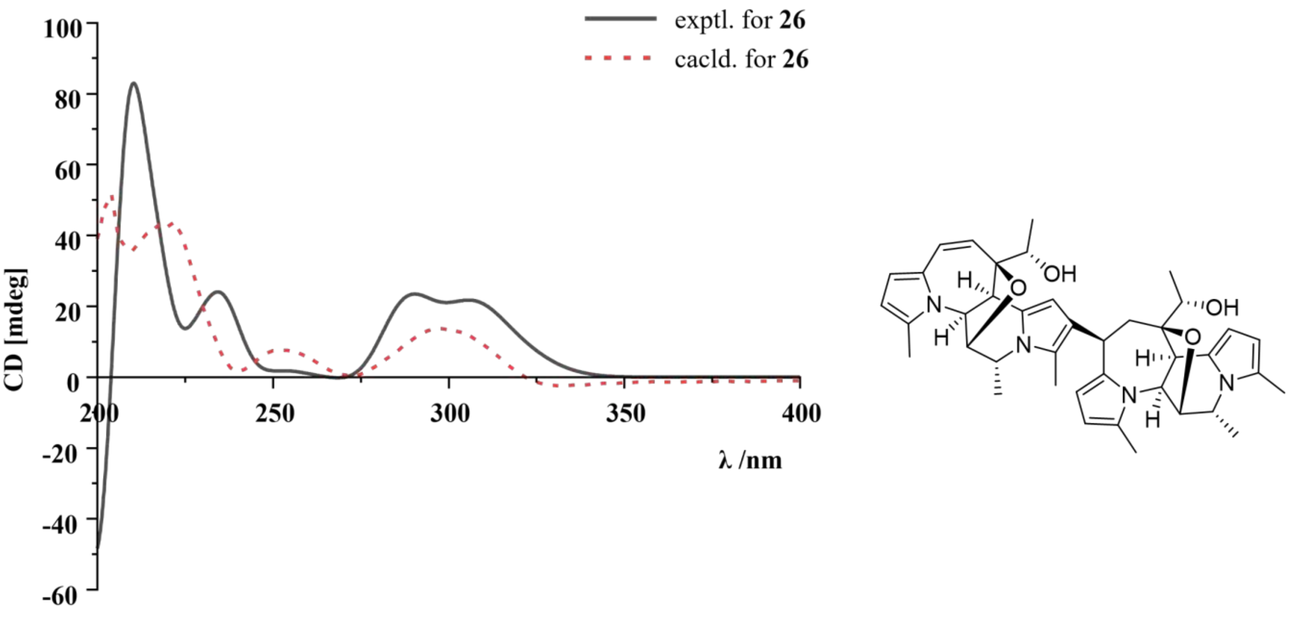

Fig. S163. ECD spectra recorded and calculated for curvamine S (**26**).

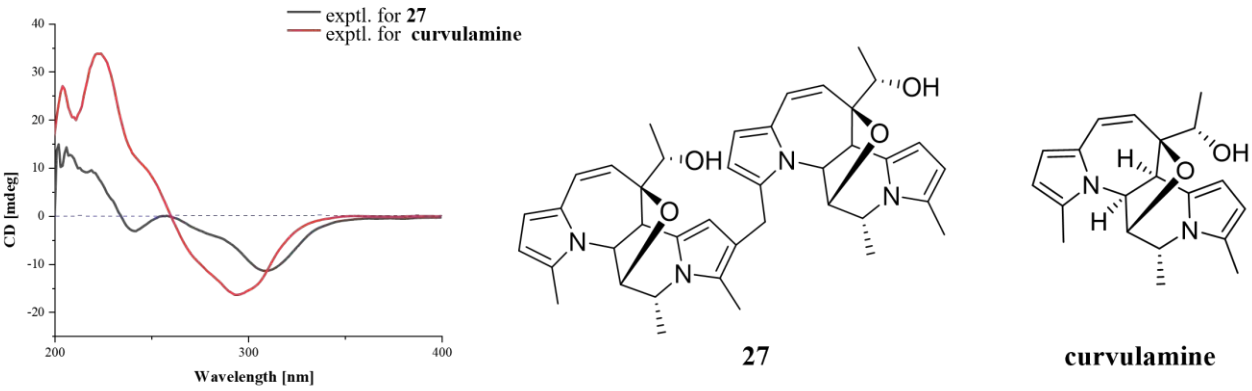

Fig. S164. ECD spectral comparison between curvamine T (**27**) and curvulamine.

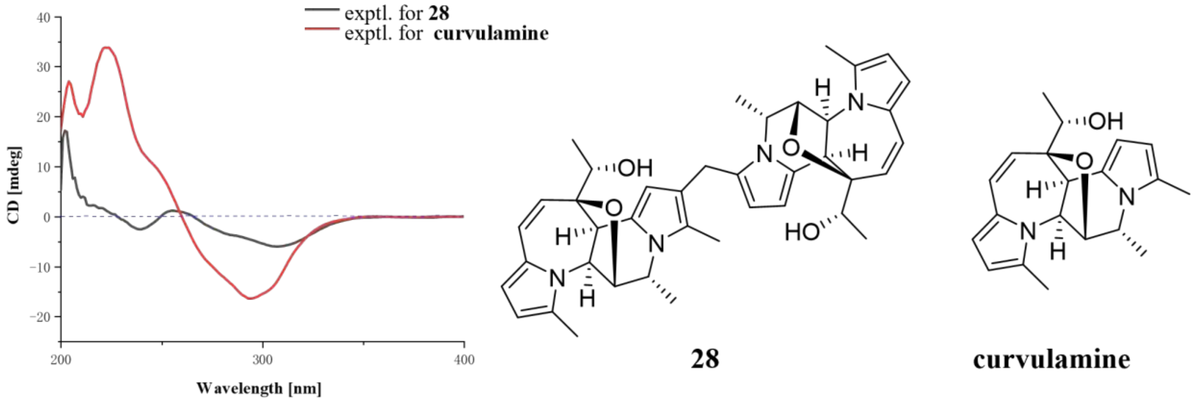

Fig. S165. ECD spectral comparison between curvamine U (**28**) and curvulamine.

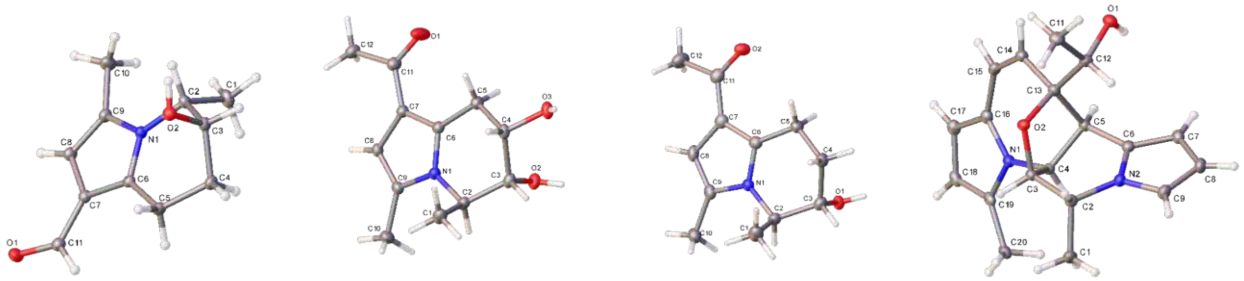

9 **10** **11** **24**

Fig. S166. Single crystal X-ray diffractions of curvamine B (**9**), curvamine C (**10**), curvamine D (**11**), and curvamine Q (**24**).

References

22. Dai GZ, Han WB, Mei YN, Xu K, Jiao RH, Ge HM, Tan RX. Pyridoxal-5′-phosphate–dependent bifunctional enzyme catalyzed biosynthesis of indolizidine alkaloids in fungi. *Proc Natl Acad Sci USA*. 2019;117(2):1174–1180.

39. Miyamoto S, Martinez GR, Medeiros MHG, Di Mascio P. Singlet molecular oxygen generated by biological hydroperoxides. *J Photochem Photobiol B*. 2014;139:24–33.

58. Medema MH, Blin K, Cimermancic P, Jager VD, Zakrzewski P, Fischbach MA, Weber T, Takano E, Breitling R. antiSMASH: rapid identification, annotation and analysis of secondary metabolite biosynthesis gene clusters in bacterial and fungal genome sequences. *Nucleic Acids Res.*2011;39:W339–W346.

59. Han WB, Lu YH, Zhang AH, Zhang GF, Mei YN, Jiang N, Lei X, Song YC, Ng SW, Tan RX. Curvulamine, a new antibacterial alkaloid incorporating two undescribed units from a *Curvularia* species. *Org Lett*. 2014;16(20):5366–5369.

60. Di Bari L, Pescitelli G, Pratelli C, Pini D, Salvadori P. Determination of Absolute Configuration of Acyclic 1,2-Diols with $Mo_2(OAc)_4$. 1. Snatzke's Method Revisited. *J Org Chem*. 2001;66(14):4819–4825.
